# Sensor Arrays: A Comprehensive Systematic Review

**DOI:** 10.3390/s25165089

**Published:** 2025-08-15

**Authors:** Sergio Domínguez-Gimeno, Raúl Igual-Catalán, Inmaculada Plaza-García

**Affiliations:** 1 Electronics Engineering and Communications Department, Escuela Universitaria Politécnica de Teruel, Universidad de Zaragoza, 44003 Teruel, Spain; inmap@unizar.es; 2 Electric Engineering Department, Escuela Universitaria Politécnica de Teruel, Universidad de Zaragoza, 44003 Teruel, Spain; rigual@unizar.es

**Keywords:** sensor arrays, review, sensor array technologies, sensor array applications, sensor array validation, sensor array software, sensor array performance metrics

## Abstract

Sensor arrays are arrangements of sensors that follow a certain pattern, usually in a row–column distribution. This study presents a systematic review on sensor arrays. For this purpose, several systematic searches of recent studies covering a period of 10 years were performed. As a result of these searches, 361 papers have been analyzed in detail. The most relevant aspects for sensor array design have been studied. In relation to sensing technologies, different categories were identified: resistive/piezoresistive, capacitive, inductive, diode-based, transistor-based, triboelectric, fiber optic, Hall effect-based, piezoelectric, and bioimpedance-based. Other aspects of sensor array design have also been analyzed: applications, validation experiments, software used for sensor array data analysis, sensor array characteristics, and performance metrics. For each aspect, the studies were classified into different subcategories. As a result of this analysis, different emerging technologies and future research challenges in sensor arrays were identified.

## 1. Introduction

**Figure 1:**
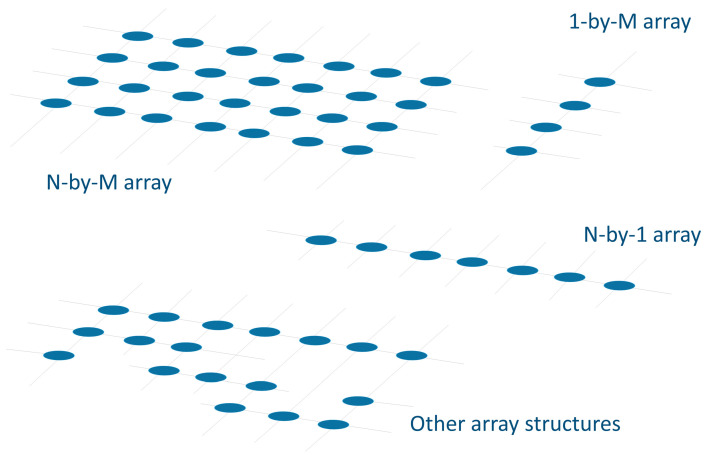
Possible sensor array configurations: each circular shape is a sensor.

Sensor arrays are a group of sensor elements arranged in a certain pattern. There are several possible configurations. The most common way to place these elements is to arrange them in N rows and M columns, obtaining a matrix of N-by-M sensors. Each sensor in the array is called a cell or sensel. It is also possible to find sensor arrays in the form of 1-by-N or M-by-1 [1,2,3], obtaining sensor vectors. In general, any structure that uses several sensors at the same time [4,5,6,7,8] or has several detection points [9,10], regardless of their distribution, can be considered a sensor array (Figure 1).

The design of sensor arrays is challenging, as several aspects have to be taken into account: sensing technology, specific application of the array, validation experiments, data analysis, sensor array characteristics or performance evaluation. Due to the large number of aspects involved in the design of sensor arrays, it is useful to have a comprehensive systematic review that analyzes all of them and identifies the research challenges in this field. To the best of our knowledge, this is the first comprehensive systematic review on sensor arrays.

Some review papers on sensor arrays are already available for the state-of-the-art. In this regard, a paper by Duan et al. [11] presented a review on flexible pressure sensor arrays. The study focused on sensor materials, manufacturing aspects, and applications. The paper analyzed sensor arrays from a materials point of view, identifying key developments in the field. However, other aspects involved in the design of sensor arrays were not reviewed. Another set of review studies on sensor arrays focused on specific applications. In this way, Länge [12] focused on bulk and surface acoustic wave sensor arrays (BAW and SAW, respectively) for use in gas and liquid sensing and biosensing. The study analyzed the components of acoustic sensor arrays, also providing an overview of commercial devices. Similarly, the paper of Nilsson & Skog [13] focused on inertial sensor arrays. The study provided a brief overview of the capabilities and limitations of these arrays, their practical problems, the most common types of array, and their main applications. It did not review the design aspects of inertial sensor arrays. Next, the review of Ozer & Henry [14] focused on arrays for electrochemical sensing. These arrays are suitable for chemical and biological applications. In fact, the review identified applications in the diagnosis of infectious diseases, in health monitoring, in wearable sensors, and in environmental analysis. The review was application-oriented.

In addition, Piron et al. [15] reviewed a specific type of sensor array: single-photon avalanche diode time-of-flight 3D imaging sensors. They were used for distance measurements. The study classified the sensor arrays into two groups and included an analysis of the evolution of the array size over time. The most significant conclusion of this study was that large-scale integration remains a major challenge. In this sense, the study of D’Amico et al. [16] reviewed sensor arrays for exhaled breath analysis. The paper examined the latest technologies in the detection of volatile compounds. It described sensor arrays and interfaces for breath analysis and their applications in the medical field. Therefore, it was a work that focused on a specific health aspect. The review of Yan et al. [17] focused specifically on fluorescent sensor arrays to detect various metal ions simultaneously. The paper reviewed different aspects of these types of sensor array such as sensing materials, data processing techniques, or real-world applications. The authors concluded that the reliability and practicability of these arrays should be improved. Portable devices presented stability problems, which affected their performance. The work of Rath et al. [18] focused on chemiresistive sensor arrays for the monitoring of gas/volatile organic compounds (VOC). The paper analyzed the historical evolution of chemiresistive sensor arrays, their manufacturing methods, algorithms for response extraction, and applications. The paper concluded that these sensor arrays had potential for health assessment and food quality analysis but had not been widely used on a commercial scale. Similarly, Kumar et al. presented a review [19] on arrays for food and beverage quality sensing. The work analyzed detection technologies, sensing approaches, and data processing methods. The review of Chen & Wu [20] focused on sensor arrays made with MXene and their application to electronic skins. The paper concluded that MXene-based electronics was a field that had not yet been explored in depth. Finally, Liu et al. [21] reviewed signal processing methods in sensor arrays used over the past twenty-five years. The paper concluded that this field was entering the era of artificial intelligence (AI), in which sensing is crucial. Other recent reviews go in the same direction [22,23,24,25,26,27].

Unlike all these previous reviews, this paper presents, for the first time to the best of our knowledge, a comprehensive systematic review on sensor arrays. It does not focus on a specific technology or application, but reviews all existing literature on sensor arrays published in recent years. As a result, 361 papers have been located and analyzed in detail. This study is intended to be of use to researchers of sensor arrays, as it considers all key aspects of their design. From this comprehensive analysis, the paper identifies challenges in sensor array research that have not yet been successfully addressed by the scientific community. The paper also includes several comparisons of studies that analyze various elements involved in the design of sensor arrays.

The aim of this work is to help researchers in the field of sensor arrays to identify, in an orderly and structured way, the extent of existing knowledge in this area, which may be of interest when proposing new sensing configurations or improving existing ones. The study reviews, classifies and analyzes the main aspects to be taken into account when designing a new sensor array: sensing technology, application, validation experiment, performance metric, sensor array characteristics, and software to be used for analysis. Therefore, this paper may be useful for researchers or technologists who wish to contribute to the advancement of knowledge in this field, as it provides a comprehensive review of the state of the art in sensor arrays.

The rest of this paper is structured as follows: Section 2 describes the search and selection procedure, Section 3 presents the sensor array technologies found in this review; Section 4 categorizes the applications of sensor array; Section 5 presents the validation experiments performed in the sensor array studies; Section 6 compiles the software used for the analysis of signals or data from sensor arrays; Section 7 summarizes the most important sensor array characteristics; Section 8 presents the metrics used to study the performance of the sensor array; and, finally, Section 9 discusses the results and draws some conclusions.

## 2. Materials and Methods

### 2.1. Search and Selection Procedure

A systematic search on sensor arrays was performed. The Web of Science database was used for this purpose. To cover all possible technologies on sensor arrays, the title keywords used in the searches were the following:

1.   “resistive” plus “sensor” plus “array”;
2.   “piezoresistive” plus “sensor” plus “array”;
3.   “capacitive” plus “sensor” plus “array”;
4.   “inductive” plus “sensor” plus “array”;
5.   “diode” plus “sensor” plus “array”;
6.   “transistor” plus “sensor” plus “array”;
7.   “piezoelectric” plus “sensor” plus “array”;
8.   “triboelectric” plus “sensor” plus “array”;
9.   “fiber” plus “optic” plus “sensor” plus “array”;
10. “hall” plus “effect” plus “sensor” plus “array”;
11. “bioimpedance” plus “sensor” plus “array”;
12. “sensor” plus “array” plus “review”.

**Figure 2:**
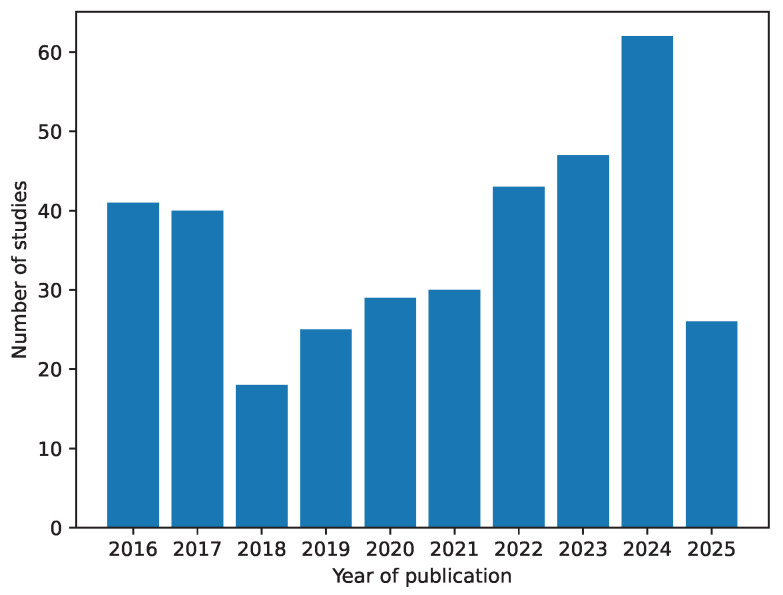
Number of studies found on sensor arrays from January 2016 to June 2025.

The searches were conducted in January 2023 and were updated in September 2023 and June 2025, incorporating in the last two searches the studies published in the time interval between the previous search and the current search. All results from January 2016 to June 2025 were considered for analysis. The studies were analyzed over the years 2023 and 2024. In July 2025, the analysis was updated to include the most recent studies. Figure 2 shows the number of studies found per year.

**Figure 3:**
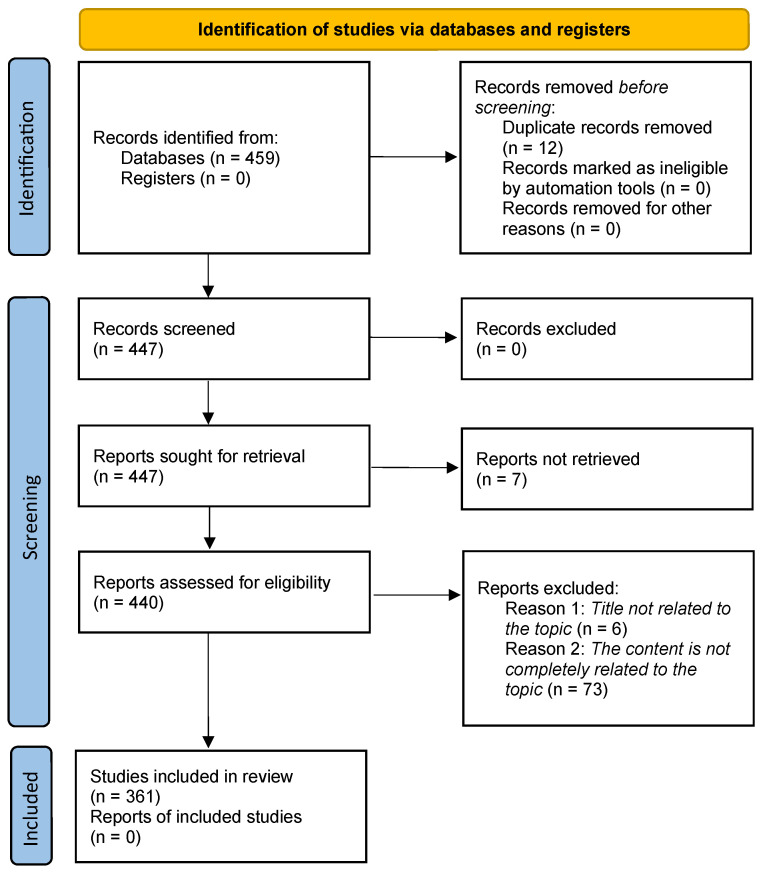
PRISMA search procedure.

A total of 459 studies were found. Overall, 12 of these studies were repeated, so 447 papers were taken. Of the remaining, 7 studies were not analyzed due to paywalls. This resulted in 440 studies. The title and abstract of each study were examined and those with results not related to the topic of this investigation, six studies, were discarded. This task was performed by two of the review authors working independently. The content of the studies was then examined and 73 papers were discarded because they were not fully related to the scope of this work. Finally, a total of 361 related studies were analyzed in depth. Two of the review authors, working independently, extracted the relevant data from these studies. No automatic tool was used to obtain the data. Missing data or non-extractable information from each paper is represented in the different tables of the review with a hyphen. They were excluded from the synthesis. Figure 3 shows the selection procedure according to the PRISMA method [28].

### 2.2. Review Structure

Existing studies on sensor arrays have been analyzed in detail and different items of interest have been extracted. They have been categorized into the following groups:

1. **Sensing technologies** (Section 3): it presents the various state-of-the-art sensing technologies in sensor arrays.
2. **Sensor array applications** (Section 4): this section presents the applications of sensor arrays found in existing studies.
3. **Validation experiments** (Section 5): this section presents the different sensor array validation techniques used in the studies carried out in this field.
4. **Software for analysis** (Section 6): this section presents the software tools used to analyze the sensor array signals or process the derived data.
5. **Sensor array characteristics** (Section 7): this section presents the different physical characteristics of the sensor arrays.
6. **Sensor array performance metrics** (Section 8): this section presents the figures of merit to evaluate the performance of the sensor arrays.

## 3. Sensing Technologies

This section presents an analysis of the different sensing technologies found in the sensor array studies. Several items related to sensing technologies are considered:

- Sensing principle;
- Array size;
- Electrode manufacturing material (electrical connection to the readout circuit);
- Sensing material.

### 3.1. Results of the Analysis

Table 1 presents the analysis of the most recent studies found in the systematic searches. The remaining studies are analyzed in Table A1, Appendix A. The “Technology” column in these tables also distinguishes between homogeneous arrays (composed of identical sensors) and heterogeneous arrays (composed of different sensors that may belong to the same sensing technology or to different technologies) [29]. Studies on heterogeneous arrays are labeled in these tables with the word “heterogeneous” in parentheses, while unlabeled studies present homogeneous arrays (the majority).

**Figure 4:**
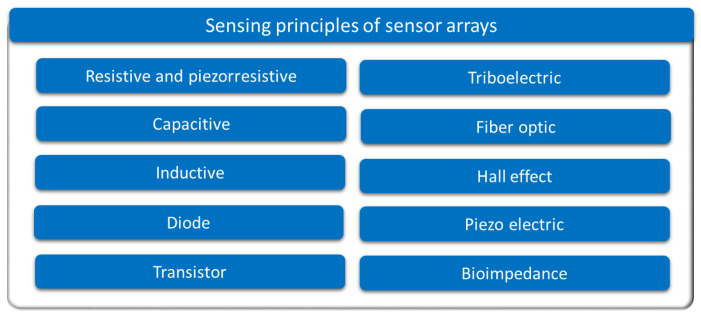
List of the operating principles of sensor arrays found in existing studies.

Focusing on the sensing principle, Figure 4 provides an overview of the different approaches found. The following subsections describe them in more detail. All studies in Table 1 and Table A1 are explicitly cited in the corresponding subsection to which they belong. Studies that implement several sensing technologies are cited in all associated subsections.

#### 3.1.1. Resistive and Piezoresistive Sensor Arrays

**Figure 5:**
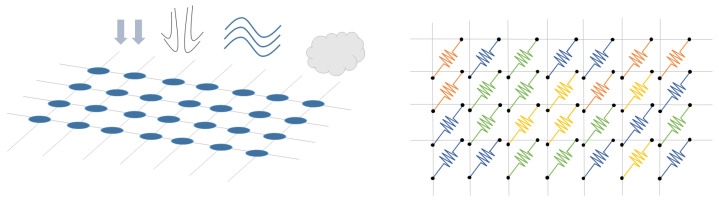
Schematic representation of the resistive technology. Sensor resistance can change depending on several variables, like pressure, gas concentration, etc.

Dozens of studies have considered resistive sensor arrays. Resistance variations in sensor arrays can be due to several phenomena (see Figure 5):

- Resistance changes due to pressure or force: Several studies have designed sensor arrays with materials that experience a change in their electrical conductivity when strained or compressed. This is the piezoresistive effect [8,46,47,50,51,52,53,54,55,56,57,58,59,60,61,62,63,64,65,66,67,68,69,70,71,72,73,74,75,76,77,78,79,80,81,82,83,84,85,86,87]. Gong et al. [88] presented a triaxial force-sensitive mat to recognize the shape, size, and curvature of external objects. A similar approach was followed by Jeon et al. [55], where bending angles were measured by attaching the sensor to a moving part. In this regard, Matsuda et al. [89] developed a deformable sensor to detect wrist flexion. The work of Jain & Bhatia [90] improved the sensitivity of a tactile sensor using a mechanical structure that increased the deformation of the sensing element. Wang et al. [48] optimized force sensing in a micro electro-mechanical system (MEMS) piezoresistive array using novel force transfer structures. In this sense, Islam et al. [58] developed a piezoresistive sensor array for monitoring pressure during sleep. Fluids such as water [3] or air [91,92] can also exert pressure on piezoresistive sensor arrays. Finally, Hailiang et al. [93] developed a force-sensitive sensor array that normally operated on a capacitive principle. However, when the external load exceeded a certain value, the capacitor turned into a short-circuit, activating a resistive sensing principle.
- Resistance changes due to temperature: These sensor arrays are based on thermistors [94,95]. The relationship between resistance and temperature can be expressed by the temperature coefficient of resistance (TCR; see the section titled Effects of Environmental Conditions (ECs)) [94,95]. In the work of Demori et al. [96], the temperature inside a food box was read using a commercial NTC thermistor. Meanwhile, Fan et al. [97] conducted a study on a new reading method using a 5-by-5 thermal resistive sensor array, which was made using Pt100 thermistors.
- Resistance changes due to the presence of chemical compounds: Chemiresistive sensor arrays are sensitive to a target analyte [18]. Their working principle is based on the absorption of the chemical substance or the production of a chemical reaction that caused a change in resistance in the arrays. These properties were used, for example, to classify gases [7,98]. Gong et al. [99] measured particle concentration using a piezoresistive array. In turn, Bassi & Ozev [100] detected changes in the surface resistance of a sensor array due to the release of electrons for conduction. In this regard, Mishra et al. [101] developed an ion-sensitive resistive sensor array consisting of two types of sensors: one sensitive to Zn(II) and the other to Cu(II). Wang et al. [102] proposed a resistive sensor array based on MXene driven by adjacent triboelectric elements.
- Resistance changes due to humidity: MXene sensor arrays can be used to detect humidity. The current increases with increasing humidity [20]. Humidity can be detected with chemiresistive sensors to monitor respiration [18]. It can also be used as a marker of food quality [19].
- Resistance changes due to magnetic field: Magnetic field-sensitive resistive sensor arrays have also been developed. Näf et al. [103] presented a customized biochip consisting of 144 spin-valve magnetoresistive sensors. This sensor array was read using a carrier suppression technique.

A large subset of works focused on improving resistive array readouts [3,7,8,50,53,56,57,60,64,66,69,72,73,74,96,98,99,104,105,106,107,108,109]. One of the most significant problems with resistive sensor arrays is crosstalk. For example, in pressure-sensitive mats (PSMs) designed for posture assessment, crosstalk can cause ghost objects to appear in areas that are not actually pressed [110]. The crosstalk effect can appear in any type of sensor array if no specific measures are taken (see the section titled Crosstalk). This problem has been partially solved in resistive sensor arrays with advanced readout circuits [97,111,112,113,114,115,116,117,118,119,120,121,122,123,124,125,126,127,128,129,130,131,132,133,134,135,136,137,138] or by software techniques [110,139,140,141]. Several authors avoided crosstalk by addressing each sensor with a readout line [3,53,56,60,64,66,67,69,73,74,96,98], but this is inefficient for large sensor mats.

#### 3.1.2. Capacitive Sensor Arrays

The capacitance between two parallel metallic surfaces is given by Equation (1):

(1) C=ϵA/d,

where *C* is the capacitance, ϵ is the dielectric constant, *A* is the area of these surfaces and *d* is the distance between them.

**Figure 6:**
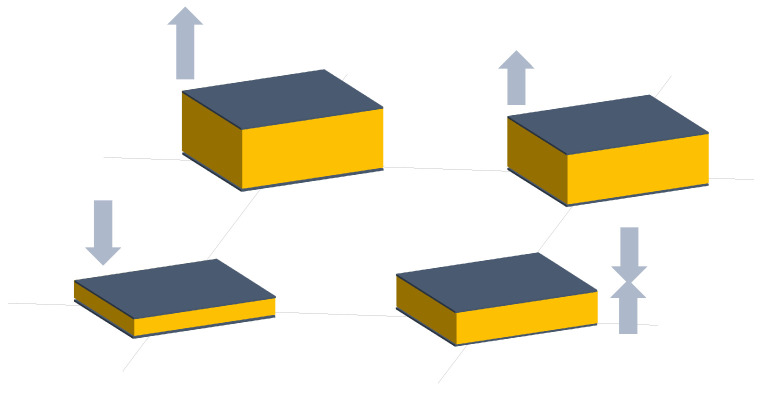
Capacitive sensor array. Sensing principle based on *d* variation.

**Figure 7:**
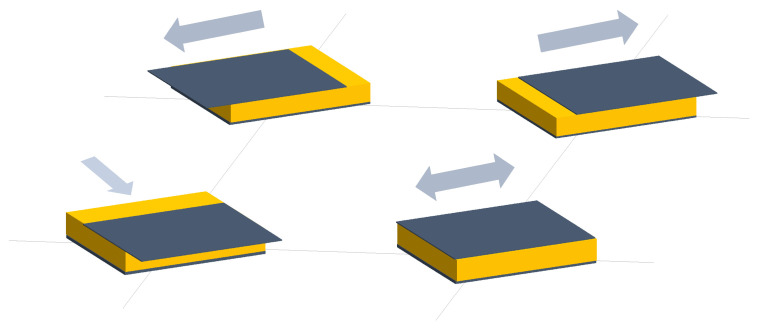
Capacitive sensor array. Sensing principle based on *A* variation.

**Figure 8:**
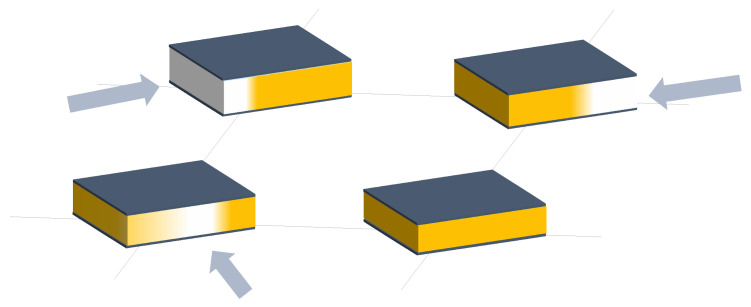
Capacitive sensor array. Sensing principle based on ϵ variation.

Two different sensing trends were found in capacitive arrays. On the one hand, several authors designed a set of capacitive sensor arrays sensitive to changes in any of the parameters of the capacitor model (*d*, *A*, and ϵ). These changes resulted in variation in *C*, which could then be converted into a physical variable. Figure 6, Figure 7 and Figure 8 graphically represent this sensing approach.

On the other hand, another group of studies measured capacitance *C* directly. The array was connected to other elements that produced a capacitance change in the array itself. In this case, the capacitive array was an auxiliary element of the sensing system.

Following these two general trends, existing studies have been grouped into different categories:

- Variation in *d* in capacitive sensor arrays (Figure 6): This is a common operating principle in PSMs, since *d* can be varied simply by applying pressure to the matrix [35,37,38,62,63,142,143,144,145,146,147,148,149,150,151,152,153,154,155,156,157,158,159,160,161,162,163,164]. The capacitance of the tactile arrays [61,165,166,167,168,169] increases with finger touch as *d* decreases. In [92,170,171,172,173], capacitive sensors subjected to strain forces also experience a variation in their dielectric thickness. The strain also produces an increase in *A* [174]. In this sense, bending or stretching the arrays also affected the *d* parameter, leading to capacitance changes. These changes can be used for gesture detection [142,150,175]. Similarly, Weichart et al. [176] proposed a bump-type sensor with spring geometry. Under an external force, the capacitance read between its different lines changed as the bump was displaced. The spring geometry allowed the structure to recover its original shape when the force disappeared. This was equivalent to modifying the parameter *d* between adjacent electrodes. In this sense, Chattopadhyay & Chowdhury [177] developed a capacitive resonator to measure heart rate. The arterial pulse caused the diaphragm to vibrate, resulting in a change in capacitance.
- Variation in *A* in capacitive sensor arrays (Figure 7): Another way to vary the capacitance is to modify the area *A* between the electrodes. This can be obtained by misaligning the two conductive plates of the capacitors, as carried out by Fernandes et al. [178]. An external force displaced the upper plate of a capacitor array, changing the effective area between parallel electrodes. This caused changes in capacitance that depended on the magnitude and direction of the applied force, resulting in a three-axis force reading application. Pu et al. [179] also applied this principle to fabricate a capacitive encoder. In this sense, Fang et al. [10] designed a petal-shaped array for robot fingertip sensing. The structure mimicked human fingertip cells and detected vibrations and the direction of the force. The top layer of the capacitive array was displaced by the action of these external forces, resulting in a change in capacitance. The operating principle was similar to other studies [178,179]. Finally, Kim et al. [61] proposed a capacitive sensor array formed by crossing wires. These wires contained coaxially wrapped conductive and dielectric parts. The contact area between the touching wires determined the capacitance of a cell. This array also measured other magnitudes through resistive and piezoelectric effects.
- Variation in ϵ in capacitive sensor arrays (Figure 8): Changes in ϵ can be used to detect variations in the dielectric material between the two metal plates of each sensor in the array [158]. The studies of Wang et al. [180] and Ye et al. [181] used the variation in the electric field between electrodes to detect an object or substance. In those studies, the object or substance to be detected acted as the “dielectric” of the sensing array. This idea was used to implement tactile capacitive arrays, which detected finger touches through the variation in the electric field [182,183,184,185,186]. A similar approach was presented in [187,188,189,190]. They proposed a contactless imaging system based on changes in the electrical permittivity ϵ of the surrounding medium. By reading the capacitance between different electrodes, the presence of an object could be detected. This technique is called electrical capacitance tomography (ECT). Sun & Sun [36] studied several algorithms to improve the co-planar array capacitive imaging technique (CACT), which is based on ECT. On the other hand, Luo et al. [191] presented a capacitive sensor array focused on detecting variations in both ϵ and *d* due to pressure changes. The sensitivity was increased by using two parameters at the same time. The humidity variation can also be registered as a ϵ variation [96,192]. Tabrizi et al. [193] developed a CMOS capacitive array whose permittivity depended on the concentration of certain solvents in the water droplets. Similarly, Zhu et al. [194] detected particle concentration by this principle in gas–solid flows.
- Variation in charge or voltage (*C* variation): Another subset of studies on capacitive sensor arrays measured charge or voltage changes between capacitor plates. In [195], when pressure is exerted on the capacitive array, the charge is displaced toward the contact point, which generates currents on the sensor surface. The measurement of charge variations is a suitable sensing approach for the detection of molecules or cells. Several studies [196,197,198,199] developed a CMOS-based cell detector. The systems detected charge changes in the sensing electrodes caused by chemical reactions, analytes or cells. In this regard, Poghossian et al. [200] developed an array of field-effect electrolyte–insulator–semiconductor capacitors (EISCAP) comparable to an Ion-Sensitive Field-Effect Transistor (ISFET; see Section 3.1.5), but with a simpler structure that facilitated fabrication processes. The presence of a chemical organic substance, and its biochemical reactions modified the charge (and potential) on one of the capacitor electrodes, changing the capacitance *C* of the sensor array. As a novelty, each capacitor was individually addressable. Similarly, Karschuck et al. [201] proposed a 4-by-4 EISCAP array that allowed the detection of pH and Au nanoparticles. On the other hand, in the field of electrical engineering, Wang et al. [9] presented a capacitive array to detect transients in a transformer. They used the stray capacitances formed in the transformer windings. Equivalent capacitors were considered between the transformer winding and a readout electrode.
- Capacitor as oscillator or part of it: In a set of works, capacitive sensors were part of a resonator whose resonant frequency depended on the variable of interest. In several studies [202,203], these sensors were referred to as Capacitive Micromachined Ultrasonic Transducers (CMUT). Seok et al. [202] developed a gas detection system that changed the resonant frequency of each CMUT depending on the surrounding gas. In other works [203,204,205,206], VOCs modified the resonant frequency of the system. Wang et al. [180] developed a sensing system capable of detecting objects in a four-sided cube. To avoid interference between sensors, an inductive element was added in series with each capacitor so that the resonant frequency of each side of the cube was different. Elzaidi et al. [207] made a distinction between the water and ice layer in marine applications using a capacitive array. A Hartley oscillator was used to measure its capacitance.

#### 3.1.3. Inductive Sensor Arrays

**Figure 9:**
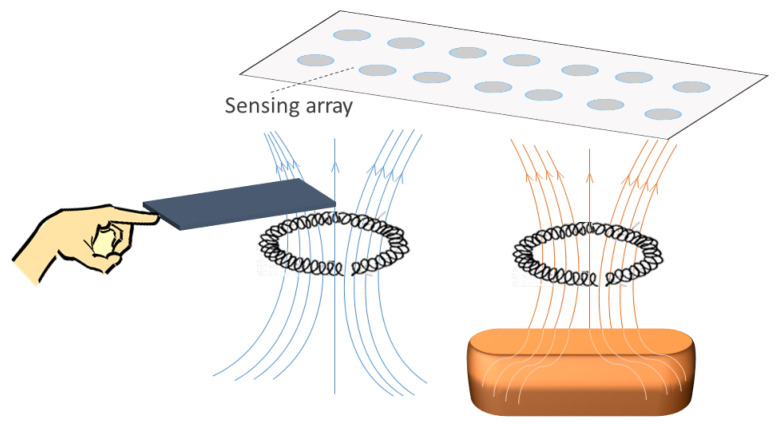
Schematic representation of the inductive technology. The inductance of the sensor array changes when certain types of materials are approached.

Inductive sensor arrays have been considered in several papers. They use the variation in a magnetic field as a transduction of a physical magnitude. The inductance of the coil is affected by the proximity of ferrous materials. Figure 9 represents this operating principle graphically.

Several works have studied inductive sensor arrays. Tang et al. [208] presented a highly sensitive inductive pressure sensor. The array was made up of planar spiral inductors and ferrite films. The permeability around the inductor was influenced by the gap between the ferrite films and the inductor. The inductance value was different depending on the separation distance. Similarly, in [209], the distance from the sensor to a magnetic object was measured using an inductive array.

An inductive triaxial pressure sensor was presented by Yeh & Fang [210]. A CMOS chip with sensor coils was used for force detection. The prototype detected force in all three axes. This CMOS platform was also used to measure the magnitude and distribution of tactile loads [211]. The operating principle was as follows: when a load was applied to the sensing system, the distance between the coil and a stainless steel sensing interface decreased, increasing the coil inductance. Similarly, Johnson et al. [212] developed a 4-by-4 pressure array capable of measuring the trajectory of a pen even when bent.

In [213], hand gestures were recognized by the coils. When the hand approached the sensor, the value of the coil decreased due to eddy currents in the finger. These variations were detected by an LC-tank resonant circuit. A similar approach was adopted by Abbasnia et al. [214]. In this case, elbow gestures were recognized with an inductive array placed around the elbow. Faria et al. [215] presented a quality control system for metal 3D printing.

Finally, a triaxial magneto-inductive sensor array was presented by Liu et al. [216]. It was designed to detect unexploded ordnance (UXO). Its mode of operation was based on two principles: alteration of the electromagnetic field generated by a coil and detection of anomalies in the geomagnetic field. Both are due to the presence of a metallic body.

#### 3.1.4. Diode Sensor Arrays

**Figure 10:**
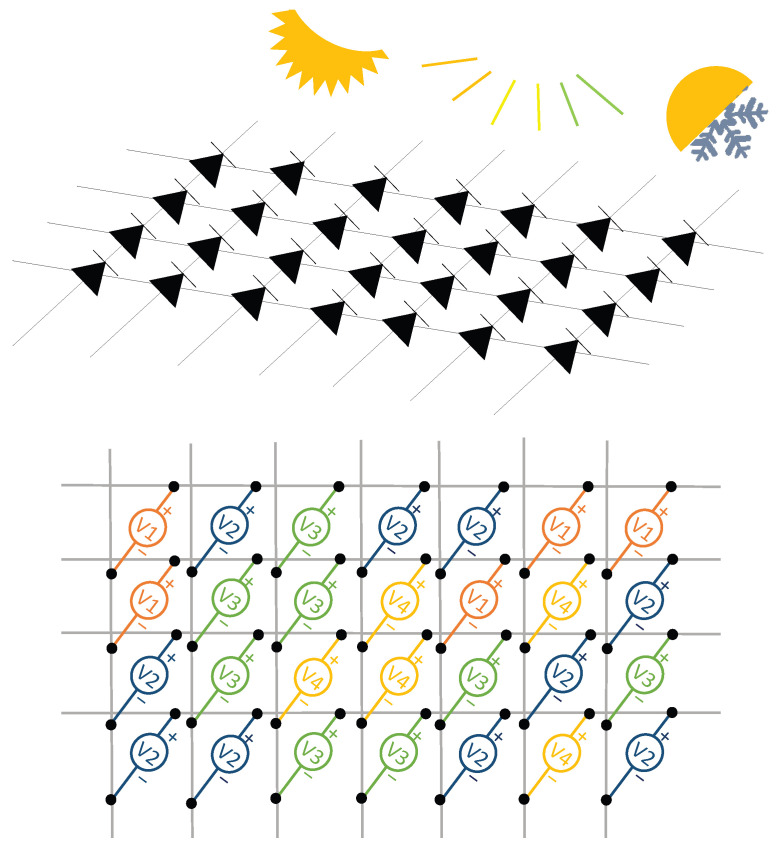
Schematic representation of the diode technology.

This array structure is composed of diodes (see Figure 10). Diodes can be used to read the temperature, as their forward voltage increases with it [52,217]. Photoconductivity is another diode parameter that is frequently used in studies. Several works used diode arrays as image sensors, since the number of free carriers in semiconductors changes when exposed to light (schematized in Figure 10). For example, Choi et al. [218] proposed a 1-transistor/1-photodiode 14-by-14 array. This array was based on photodiodes and was flexible, so it could operate under mechanical deformations. In this regard, Jia et al. [219] presented a diode array for imaging and radiation detection. This array was useful for scientific instrumentation applications.

On the other hand, Lee et al. [62] designed a flexible display device that adhered to human skin. It was composed of an array of quantum-dot light-emitting diodes. The array displayed information about different biosignals registered by other sensors. It was stable to deformations of different types. Similarly, a diode array for biomedical applications was also proposed by Kundu et al. [220]. It focused on the use of optical means for the identification of viruses or antibodies in blood. The study proposed a novel photodiode structure that improved performance in the visible range of optical irradiation.

#### 3.1.5. Transistor Sensor Arrays

**Figure 11:**
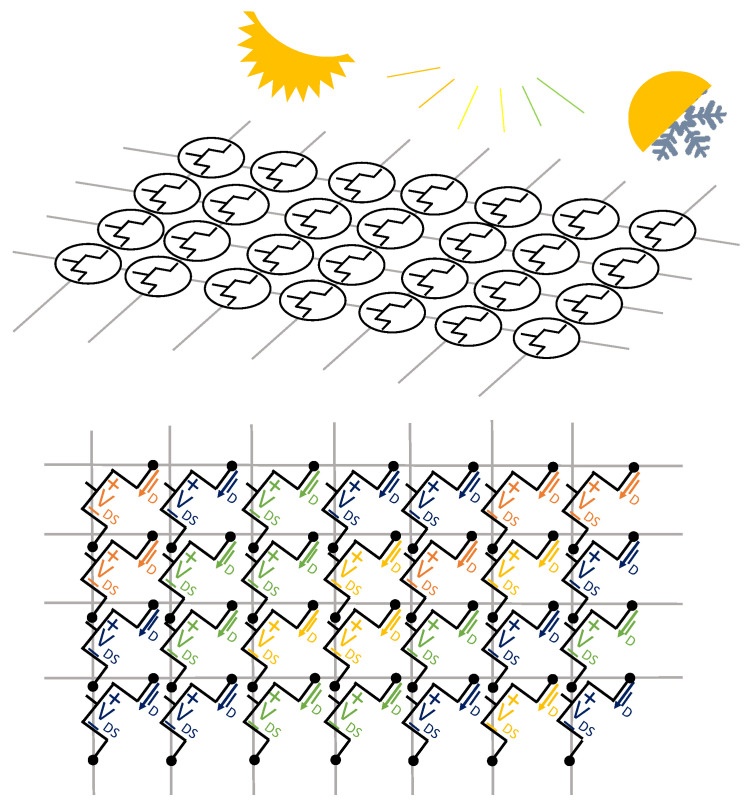
Schematic representation of transistor sensor arrays.

Transistors are active electronic components whose current through varies depending on a physical event. Transistors are used in sensor arrays because of their versatility, high sensitivity, and easy miniaturization. FETs are the most common type of transistor used in sensor arrays. In FETs, the current through their main terminals (drain and source) in active mode $I_{D}$ is given by Equation (2):

(2) $$I_{D}=I_{DSS}{\left(1-V_{GS}/V_{P}\right)}^{2},$$

where $I_{DSS}$ is the current when the transistor gate is connected to its source, $V_{GS}$ is the gate-to-source voltage, and $V_{P}$ is the threshold gate-to-source voltage required to reach the active mode. Another important parameter is the drain-to-source voltage $V_{DS}$, which is the voltage between the two main terminals of the transistor. This parameter is often related to $I_{D}$ and $V_{GS}$ in the characteristic curves of the transistors [58,76]. The sensing principle of transistor sensor arrays is schematically represented in Figure 11. Different detection trends were found for the state-of-the-art depending on the specific parameter of transistor arrays ($I_{D}$ and $V_{GS}$) that is sensitive to the physical variable under sensing.

- $I_{D}$ change detection: A subset of works transduced the variable of interest into a change in the drain current $I_{D}$ of the array transistors. This parameter is usually related to $V_{DS}$ in $I_{D}$-$V_{DS}$ characteristic curves. Depending on the operating principle that produced this change in $I_{D}$, the studies can be classified as follows:
  – Variation in $I_{D}$ due to pressure or stretching: If pressure is applied to the transistor, the distance between the gate and source–drain electrodes decreases and, acting as a parallel plate capacitor, its capacitance increases [221,222]. Due to this increase in capacitance, the charge inside the FET also increases, resulting in a higher drain current $I_{D}$. Unlike piezoresistive sensors, FET-based pressure arrays are active arrays, which can themselves avoid crosstalk [222]. Similarly, moving-gate FETs (MG-FETs) allow movement of their gate as a way of measuring directional force [223]. Moving the gate of an FET also causes a capacitance change, triggering the same chain of effects that was explained above. In turn, Su et al. [76] presented self-healable, printable polymers whose conductivity reacted to stretching or pressure. These materials were used to form organic electrochemical transistors, resulting in stretchable self-healable tactile arrays. In this sense, Ren et al. [224] proposed an electret sensor array. In this type of array, the gate of each transistor was connected to a charge induction electrode. When the sensor was pressed, a charge redistribution occurred between the electrode and the transistor gate. This allowed the transistor to drive a higher $I_{DS}$ than in the relaxed state.
  – Variation in $I_{D}$ due to chemical phenomena: The work of Bhat et al. [225] presented a ZnO nanorod FET array to detect analytes. The drain current $I_{D}$ increased significantly with the presence of these analytes. Hsu et al. [226] proposed high-electron mobility transistor (HEMT) array biosensors that showed an increase in $I_{D}$ when exposed to an exciting buffer solution. This current was controlled through $V_{G}$.
  – Variation in $I_{D}$ due to optical phenomena: Flexible imaging sensors were developed by Chen et al. [227] using Organic Thin-Film Transistors (OTFTs). $I_{D}$ changed depending on the intensity of the incident laser. In the field of imaging, Hu et al. [228] presented an array composed of dual-gate TFT (DG-TFT). In that array, several diodes acted as photodetectors, controlling the conductivity between the two gates and allowing the TFT drive current to flow between the drain and the source. In the work of Kim et al. [229] arrays of organic photomemory transistors contained organic light-sensitive semiconductors, allowing conductivity between their main terminals when exposed to light. This was a new approach that integrated both the photodiode and an additional memory device in the same transistor. Tang et al. [230] implemented radiation strike sensor arrays using a two-transistor structure. The electrical variable that transduced these radiation strikes was the charge accumulated in the transistors. These transistors operated digitally, imitating a random access memory (RAM) array.
  – $I_{D}$ frequency detection: Sensor arrays can also measure the frequency of the variation in $I_{D}$. Hessel et al. [231] presented a cantilever FET (CFET) array that had oscillatory behavior in the presence of certain adsorbates. The electrostatic forces inside the transistor caused self-oscillation, which directly resulted in a variation in $I_{D}$. When the mass on the cantilever changed, there was a shift of the vibration frequency. This work provided the relationship between cantilever mass variation and frequency shift. The circuit proposed by Hessel et al. [231] generated a square signal whose frequency depended on this concentration. This square signal was the result of placing two CFET output signals on AND gate inputs.
- $V_{GS}$ change detection: Several sensor array studies used the gate voltage $V_{GS}$ as the physical variable transducer. Some of them were based on ISFETs. For example, pH sensor arrays featured a sensitive gate, whose voltage varied as a function of the free hydrogen ions in the medium [217,232]. In the work of Yuan et al. [233], chloride ions were detected using these ISFETs. Similarly, electrolyte-gated carbon nanotube (CNT) FETs showed good performance in sensing the enzyme reaction [234]. These transistors did not depend on pH variations to detect enzymatic reactions, which is the typical approach [234]. These transistors were also applied in the work of Zou et al. [235] for gas detection. When gas molecules approached the sensing layer (the sensitive gate), charges were distributed along the oxide layer, so that conductivity was produced. In this sense, Zhai et al. [236] proposed an array for ${\mathrm{NH}}_{3}$ detection. It was observed that $V_{P}$ shifted with its concentration. A similar idea was presented by Tao et al. [237] for antibiotic detection. This principle of operation can be likened to a capacitor model [237]. Liu et al. [238] also developed a gas-sensitive transistor array was based on this principle. Electronic noses (e-noses) are a typical application of transistor arrays [34]. Gao et al. [239] proposed a DNA-sensitive biosensor. It was based on graphene-FETs (GFETs). A specific parameter of GFETs is the Dirac voltage, which is the voltage at which the conductivity of graphene is minimal [239]. In that work, this parameter changes depending on the concentration of complementary target DNA. In this sense, Li et al. [240] proposed a dopamine-sensitive array. The oxidation of dopamine in contact with the electrode generated a potential change.

#### 3.1.6. Piezoelectric Sensor Arrays

**Figure 12:**
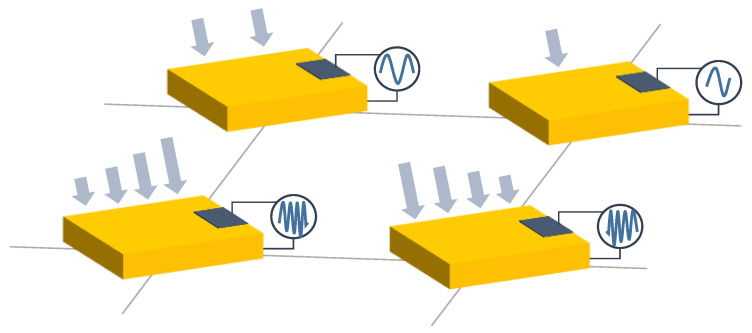
Piezoelectric sensor array. Mechanical piezoelectric arrays.

**Figure 13:**
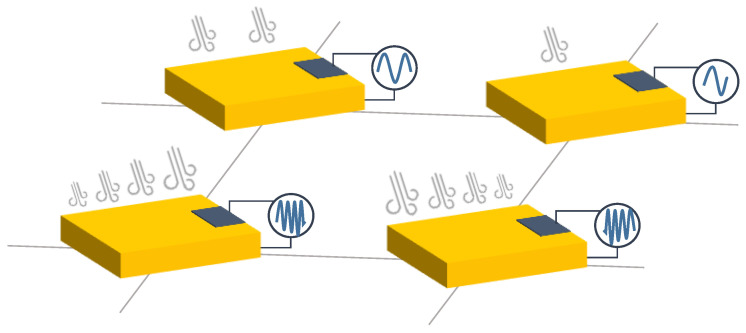
Piezoelectric sensor array. Airflow-based piezoelectric arrays.

**Figure 14:**
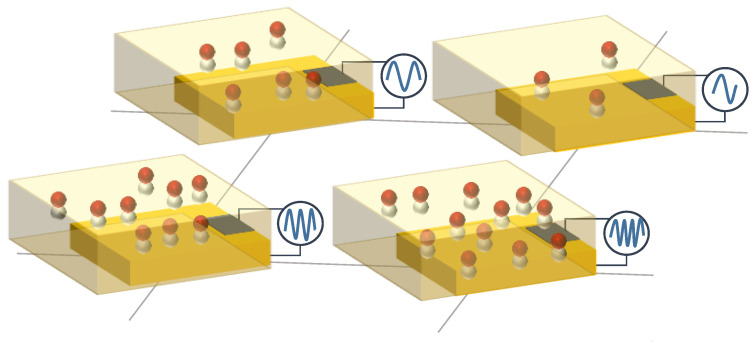
Piezoelectric sensor array. Gas-based piezoelectric arrays.

**Figure 15:**
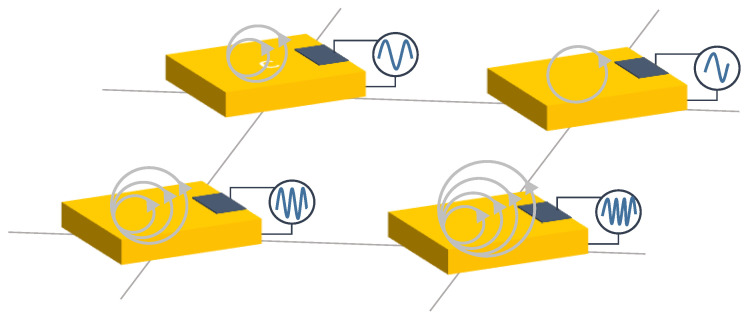
Piezoelectric sensor array. Electromagnetic-based piezoelectric arrays.

Piezoelectric sensor arrays can generate an electrical signal when stressed. This effect can be used to feed low-power devices (see Section 4.1.9). They can be classified according to the external stimuli to which they respond:

- Mechanical piezoelectric arrays: The operating principle is based on the generation of a voltage spike when a sensor of the array is pressed and the generation of another voltage spike but of opposite sign when the sensor is released [63,156,241,242,243,244,245,246,247,248,249,250,251,252,253,254,255,256,257,258,259,260,261,262,263]. Thus, if pressure is periodically applied to the sensor (e.g., through a pressure machine or due to human movement), the generated signal is also periodic. In fact, the output signal would have the same period as the pressure event [264]. In this regard, the measurement of triaxial forces was performed by Yu et al. [265] with an external shaker applying a periodic force of 20 Hz to a piezoelectric array to detect shear and vertical forces. Similarly, Chen et al. [266] designed a piezoelectric array also for the measurement of the triaxial force. To test the sensor, they used a flexible NdFeB-polydimethylsiloxane (NdFeB-PDMS) magnetic bar that was placed on top of the sensor and then moved under an external magnetic field. In the work of Kim et al. [267] a piezoelectric array was used to obtain the strain vs. stress curve but not by monitoring the voltage output of the array, but rather its impedance. In the work of Lei et al. [268], piezo-capacitors were used to develop a pressure-sensitive array. The structure was driven by amorphous In-Tin-ZnO DG-TFTs. Luo et al. [269] developed a piezoelectric resonator whose resonance frequency was strain-dependent.
  The bending of the array also triggers the piezoelectric effect [251,261,270,271]. In this sense, a mass that moves over a piezoelectric surface and bends also acts as a variable force [272,273,274]. For example, a roughness-sensitive robot fingertip was developed by Liu et al. [275]. The operating principle was based on moving the array on a rough surface. Grasping a rough surface generated a variable force on the sensor array. Several commercially available sensors are used in [276,277] to detect gestures by reading muscle deformation in the wrist, as this force generates an upward and downward signal in the sensor.
  Another set of piezoelectric arrays were sensitive to vibrations [261,274,278,279,280,281].
  In the field of biomedical engineering, a pulsating 3D heart model also produced a variable force on the sensor array, so an electrocardiogram signal could be obtained without external feeding circuits [282]. In the work of Tian et al. [283], a new technique was proposed to fabricate strain-sensitive piezoelectric arrays. The fabricated array was used to measure heartbeats in a simulated heart. Similarly, pulse can be measured with piezoelectric arrays [266,284,285]. Iizuka et al. [286] developed a pressure sensor array for the detection of laryngeal movements. A set of works used the pressure exerted on the sensor array when breathing. In this sense, Tamiziniyan & Febina [287] integrated the piezoelectric array into a quilt. In this way, obstructive sleep apneas (OSAs) were detected. The work of Feng & Su [288] developed a piezoelectric sensor array in which respiratory air flow generated a pressure difference on both sides of the sensor and caused deformation of the piezoelectric membrane. These sensing principles are schematized in Figure 12.
- Airflow-based piezoelectric arrays: Bian et al. [6] proposed a piezoelectric array inspired by the structure of cricket airflow detectors. Piezoelectric sensors were cylinders that bent under the force of the wind. This bending also generated a voltage signal in the material. In this sense, Cong & Jing [289] presented a piezoelectric array inside an axial flow compressor to detect the vibrations of the blades and the noise generated by these vibrations. The array was excited by the airflow inside the compressor. This sensing principle is schematized in Figure 13.
- Sound-based piezoelectric arrays: Piezoelectric arrays have been used to measure sound [290] since they are sensitive to vibratory movements [291,292]. Holeczek et al. [293] used piezoelectric sensor–actuator arrays to test composites for ultrasonic applications. The array performed both sound generation and measurement of the associated vibration. Similarly, in the work of Si & Wang [294], PZT actuators vibrated at a given frequency to test laminated composites. Zhen et al. [271] also presented a PZT array for sound detection. In turn, Nagai et al. [295] developed a piezoelectric sensor array capable of extracting Young’s modulus and viscosity of a material by analyzing the peaks of the output voltage signal. In addition, several studies [4,296,297,298,299,300] have used piezoelectric sound arrays based on the generation of ultrasonic waves inside materials to detect faults [291,294,296,297,298,299,300,301], obtain an image of damage [297,299,302,303,304], or perform voice recognition [305].
- Gas-based piezoelectric arrays: Piezoelectric Quartz Resonators (PQRs) are piezoelectric sensors that are sensitive to gas concentration. Shuba et al. [306], and Kuchmenko et al. [307] presented PQR arrays that were modified using thin films of sorbents. This modification allowed them to change their vibration frequency depending on the type of gas. From the frequency value, several types of gases could be distinguished. In the case of [307], *Helicobacter pyroli* was detected by gas concentration. A similar working principle appeared in the work of Li et al. [2], where a cantilever resonator array was used. The cantilever oscillated at different frequencies in the presence of different gases. The cantilever contained five electrodes to maximize the amplitude of the resonance peaks. Finally, Lamb wave resonators (LWR), a type of piezoelectric sensor, were also found to be sensitive to different ambient gas concentrations [308]. This sensing principle is schematized in Figure 14.
- Electromagnetic-based piezoelectric arrays: Ferromagnetic-piezoelectric (FMPE) sensor arrays are systems that generate an output voltage when deformed by a variable external magnetic field [309]. These devices exhibit cross-coupling between electric and magnetic fields, given by the magnetoelectric voltage coefficient [309], according to Equation (3). In that equation, dE/dH is the derivative of the electric field versus the magnetic field, dV/dH is the derivative of the output voltage versus the magnetic field, and *t* is the thickness of the piezoelectric layer.
  (3) $$\alpha_{E}=dE/dH=dV/\left(tdH\right)$$
  This type of piezoelectric array can be used to measure magnetic fields. In the work of Kim et al. [5], piezoelectric sensors are oriented in various positions to sense the direction and magnitude of the field. This sensing principle is schematized in Figure 15.
- Humidity-based piezoelectric arrays: Sensor arrays consisting of piezoelectric membranes change their resonant frequency when absorbing water molecules due to a change in the mass of the membrane [310,311]. These devices are called pMUTs (piezoelectric micromachined ultrasonic transistors), similar to CMUTs. In this way, humidity can be measured. The same principle was followed by Feng & Su et al. [312] to develop a rhinomanometer with an integrated humidity sensor.
- Self-harvesting sensor arrays: Piezoelectric arrays have the potential to be self-harvestable, as they can produce their own voltage [63,292]. In this sense, Kumar et al. [313] presented an Internet of Things (IoT) system based on piezoelectric arrays placed under the roads of a smart city. The proposal was to convert mechanical energy from cars that drive on the roads into electricity. Kim & Yun [63] combined triboelectric and piezoelectric arrays to harvest energy.
- Temperature-based piezoelectric arrays: The pyroelectric effect is used to detect temperature changes [264,314].

#### 3.1.7. Triboelectric Sensor Arrays

Triboelectric sensor arrays are a self-harvestable solution for sensing. These devices are referred to as TEG (triboelectric generator) or TENG (triboelectric nanogenerator) by Yan et al. [315]. They produce a voltage between two materials in contact with different triboelectric polarities. Like piezoelectric arrays, triboelectric arrays can also be used to supply low-power devices [63]. A “positive material” stores a positive charge, while a “negative material” stores a negative charge. When the materials are separated, for example, by sliding, their charge becomes neutral again [316]. This effect appears due to electrostatic induction [61,315,317,318,319] and the magnetic properties of the materials [316].

**Figure 16:**
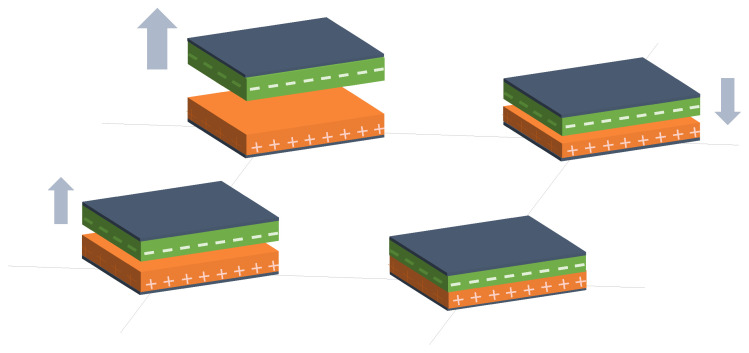
Triboeletric sensor array. Triboelectric effect by separation.

**Figure 17:**
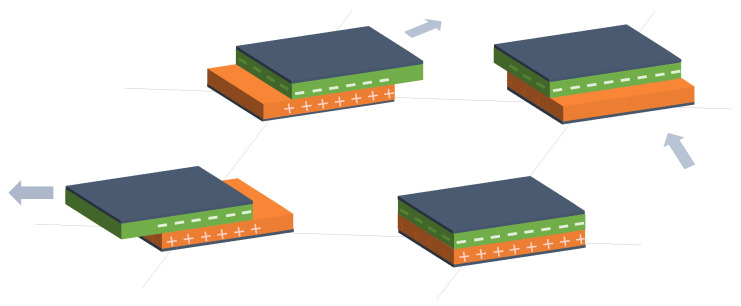
Triboeletric sensor array. Triboelectric effect by sliding.

**Figure 18:**
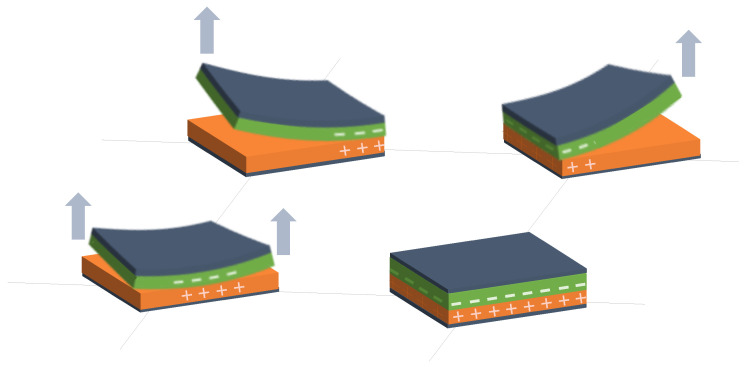
Triboeletric sensor array. Triboelectric effect by flapping.

These material properties have been exploited in different ways to detect various phenomena:

- Triboelectric effect by separation (Figure 16): Gao et al. [320] measured the pressure using a triboelectric array. A sandwich structure composed of polyvinylidene fluoride (PVDF)-PDMS, nylon, and aluminum, in this order, was presented. PVDF-PDMS was a negatively charged triboelectric polymer, nylon was positively charged, and aluminum was also negatively charged. When the PVDF-PDMS layer was pressed against the nylon layer, the positive charges of the nylon layer shifted toward the PVDF-PDMS layer, and the negative charges of the PVDF-PDMS layer did the same toward the nylon layer. The aluminum layer was held with a near neutral charge. When the PVDF-PDMS layer was released, the nylon layer rearranged its positive charges on the contact surface with the aluminum layer. In this way, a potential difference in the aluminum layer was obtained, and from this, the value of the pressure exerted on the triboelectric array could be obtained. Unlike piezoelectric arrays, triboelectric sensor arrays can generate voltage in a static state. Chen et al. [321] followed a similar principle to measure force, but using a glass-based single electrode TEG. Chen et al. [45] implemented a 3-by-3 triboelectric array in which the negative material was PDSM, and the positive materials were Cu and water. Yang et al. [318] developed a smart traffic monitoring system using silver electrodes, PVDF and a polyethylene terephthalate (PET) substrate; all embedded with a Raspberry Pi. Jang et al. [322] performed ultraviolet (UV) patterning to control the sensitivity of a stretchable triboelectric array subjected to touches. This array could detect slippage, pressure, and grip. Yan et al. [315] develop a 16-by-16 dense triboelectric array with laser-induced graphene electrodes that was immune to bending and also followed this principle. This effect was also used by Lee et al. [323] to develop a plantar pressure system that performed equally well in atmosphere and in water. Wang et al. [324] presented a TENG array combined with FETs to amplify the output signal. The TENG powered the FET gate to develop a digital keyboard and analogue pressure systems. Similarly, Li et al. [30] developed a cyber-secure numeric keyboard. Yang et al. [325] presented a cuboid TENG array using silicon rubber to improve the output voltage under pressure. Ahmed et al. [326] and Liu et al. [30] developed computer keyboards using the same principle. The contact separation principle prevails in pressure triboelectric arrays [39,40,41,42,43,44,163,224,327,328,329,330,331]. A similar operation concept was followed by Wang et al. [102] to develop a resistive gas sensor array driven by TENGs. These TENGs generated power from wind motion.
- Triboelectric effect by sliding (Figure 17): Two triboelectric materials generate a voltage signal when they slide. This signal varies according to the amount of material in contact. This operating principle was used by Qin et al. [316] to detect gestures in human-machine interface (HMI) applications.
- Triboelectric effect by flapping (Figure 18): Ko et al. [317] developed a flap-based triboelectric array to measure wind speed and direction. The flaps are thin triangular surfaces of triboelectric material that are attached to the rest of the structure on only one side of the sensor. They are presented in a circular array. The flaps (made of Al and PET) bend as a result of air movement. The flaps are initially rolled up and do not touch the circular base of the array, made of polytetrafluoroethylene (PTFE) and Cu. When the wind blows against the flaps, they unroll and touch the base of the array. The potential difference between the base and the flap is greater with a higher wind speed, as the contact surface is larger.

#### 3.1.8. Fiber-Optic Sensor Arrays

**Figure 19:**
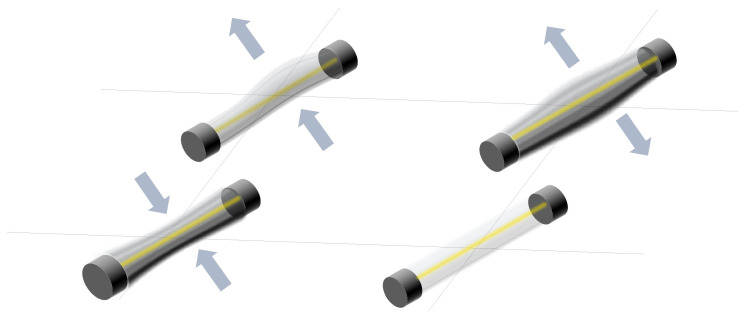
Fiber optic sensor array. Operating principle based on compression, bending or deformation.

**Figure 20:**
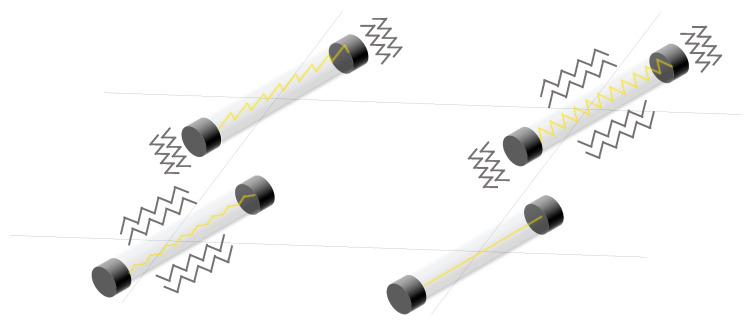
Fiber optic sensor array. Operating principle based on vibration.

**Figure 21:**
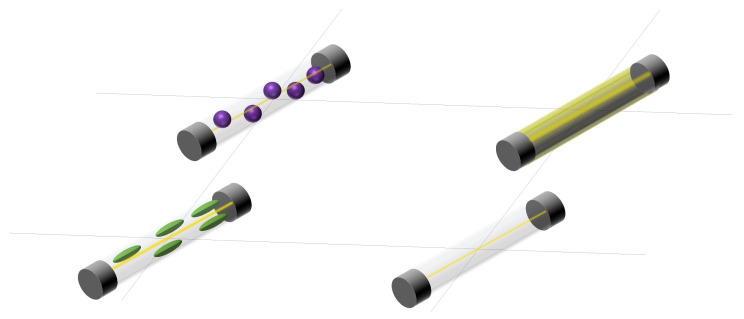
Fiber-optic sensor array. Operating principle based on the modification of internal conditions.

This type of sensor is becoming popular in industrial applications due to their flexibility, high resolution and reliability. Fiber-optic sensors use transparent wires to conduct light. When these wires are compressed, strained [31,332,333], bent [334] (Figure 19), exposed to vibrations [49,335,336] (Figure 20) or modified (Figure 21), light is reflected and refracted inside the material differently from under normal conditions. It can also vary with temperature [31,334,337]. These variations are measured by interrogators. Noise is an important issue in fiber-optic arrays. It is discussed in detail in the work of Liu et al. [338], which includes possible improvement techniques.

Some specific work has been published on high-frequency sound measurement using fiber-optic sensor arrays. Wang et al. [339] presented a fiber-optic sensor array for the detection of high-frequency signals, enabling the location of a real-time sound source. Li et al. [340] also worked on sound source localization with this technology. Pallayil [341] presented a comparative study on underwater acoustic sensing technologies. One of the systems compared was the fiber-optic sensor-based hydrophone array, which featured immunity to electromagnetic interference, lightweight cabling, flexibility, and compact mounting. In this sense, Arbel et al. [342] proposed a fiber-optic system for underwater ultrasound detection. Yang et al. [1] designed an ultrasound sensing system based on a photo-thermal tunable fiber-optic array. The change in the length of the cavity due to its thermal expansion caused a change in the optimum operating wavelength. This improved the detection of ultrasounds. Similarly, Zhang et al. [343] and Liu et al. [344] proposed a fiber-optic ultrasonic sensing array. The system consisted of a multichannel setup for interrogating the fiber-optic array. Shin et al. [345] monitored the position of the proton pencil beam spot using an array of Fiber-Optic Cerenkov Radiation Sensors (AFCRSs). In turn, Baker et al. [346] used fiber-optic technology for ultrasonic needle tracking. A piezoelectric array was also used for this purpose.

In [347], time division multiplexing (TDM) was employed to enable transmission of multiple optical signals over a single fiber thread. In addition, an analytical model was proposed to investigate the effects of noise aliasing in this type of multiplexing. TDM was also performed in [348] with FPGA-based interrogation. In [349], TDM was combined with dense-wavelength division multiplexing to better avoid crosstalk. Zhang et al. presented a new type of TDM [33] that significantly reduced total harmonic distortion (THD) during demodulation. Several fiber-optic studies focused on new interrogation techniques for improve sensing [32,33,347,348,349]. Wave Division Multiplexing [349] and Frequency Division Multiplexing in sensor arrays have also been studied [49].

Ren et al. [350] proposed a fiber-optic sensor array for anti-vandalism detection in pipelines. It was based on vibration detection. The system had an optical part connected to the sensor array attached to the pipeline and an FPGA-based interrogation. In this way, vibrations in the pipeline caused changes in the optical conditions. Similarly, Gutierrez et al. [335] proposed a fiber-optic array for the detection of strain and vibration. It was applied to launch vehicles and aerospace structures. The system was based on fiber Bragg Grating (FBG) and operated as an inertial measurement unit (IMU) and therefore was called FBG-IMU. It was vertically integrated into the body of the launch vehicle to transform vehicle strains and vibrations into optical changes, from which the dynamic characteristics of the aerospace structure could be predicted. Mendoza et al. [351] proposed a woven array that measured the deformation of parachutes during deployment in the air. Liu et al. [352] presented an array of deformation- and stress-sensitive fiber-optic sensors.

On the other hand, inorganic fiber-optic scintillators were proposed by Park et al. [353] for scanning radioactive waste. A gamma-ray generator was used to test the device. The fiber-optic array read the emitted rays in a horizontal line, while moving vertically, resulting in a 2D map of the received radiation. It was demonstrated that the sensor could effectively detect radioactive wavelengths. In turn, Qin et al. [354] proposed a dual-line fiber-optic sensor array to develop a low-cost image system. Kim et al. [355] performed antigen concentration measurements using an array of fiber-optic surface plasmon sensors. Gold films were used to create hotspots and improve antigen detection. In [356], volatile organic liquids (VOLs) were detected by analyzing the transient response of the fiber-optic sensor during their evaporation processes.

#### 3.1.9. Hall Effect Sensor Arrays

**Figure 22:**
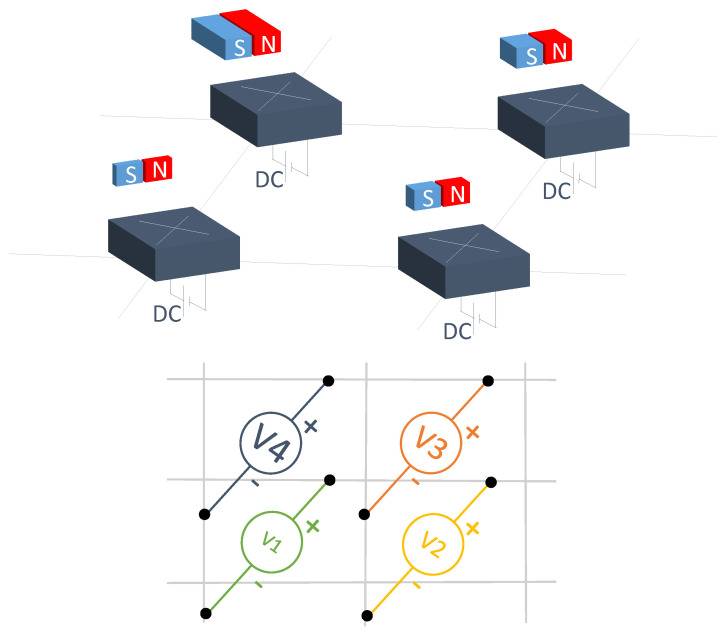
Schematic representation of the Hall effect technology. Magnetic fields generate associated voltages at the sensor output.

Hall effect sensors produce a voltage output proportional to a magnetic field. Thus, they are used to measure current or magnetic fields. These sensors are contactless (Figure 22). Susac et al. [357] used an array of Hall effect sensors to provide contactless information about position of various objects, which had a permanent magnet embedded inside them. This same principle was applied by Pani et al. [358]. Similarly, a Hall effect sensor array can be used to determine the position in microrobotics by magnetic localization [359,360]. Also on this topic, Fischer et al. [361] developed a Hall effect sensor array to track the position of magnetic surgical instruments within the human body. On the other hand, Luca et al. [362] measured the imbalances in the current supplied by various batteries using a Hall effect array. As the output voltage of Hall effect sensors is related to the induced magnetic fields, a common application is current sensing. Hall effect sensors were placed inside the circumference of a ferrite to increase the magnetic flux density. Similarly, Tang et al. [363] measured high currents in rectangular conductors by arranging six sensors in an elliptical shape. Due to its magnetic field measurement capability, Nhalil et al. [364] developed a magnetometric Hall effect sensor array with very low equivalent magnetic noise (EMN). Finally, Vizel et al. [365] mapped the magnetic fields produced by any source. They obtained both the position and orientation of the source coil.

#### 3.1.10. Bioimpedance Sensor Arrays

**Figure 23:**
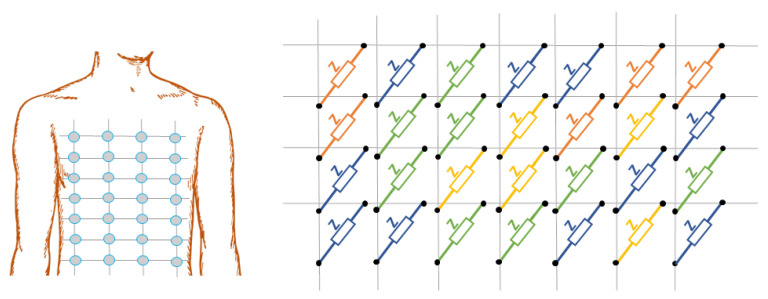
Schematic representation of the bioimpedance technology. Electrical variables are measured around organic tissues.

Bioimpedance sensors measure the internal resistance of organic tissues to infer different physiological variables [366]. Figure 23 shows a schematic representation of this principle. In the studies analyzed in this work, they have been used to monitor the process of skin wound healing [367] and blood pressure [366]. These systems use AC signals to study phenomena of interest that depend on the electrical impedance of the skin and lower tissues [367].

### 3.2. Brief Conclusion of Sensing Technologies

**Figure 24:**
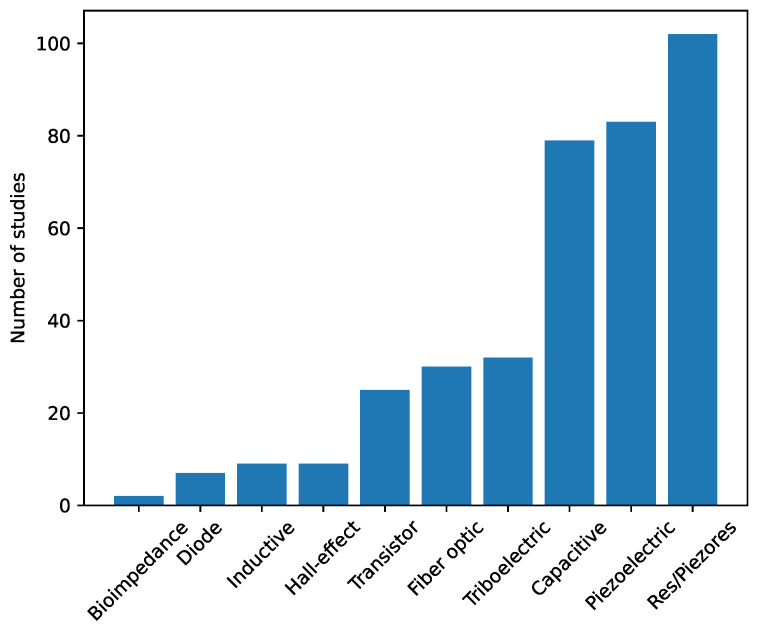
Number of studies found in this research for each sensing technology.

Figure 24 represents the number of papers on sensor arrays found for each sensing principle. Resistive/piezoresistive, piezoelectric, and capacitive technologies are by far the most widely used sensing technologies in sensor arrays. They represent 28.5%, 23.0%, and 21.9% of all studies analyzed, respectively. These technologies can be easily manufactured and integrated and cover a wide range of applications. In addition, readout technologies used to measure the resistance, capacitance, or output voltage values are highly developed and commercially available. However, bioimpedance and diode arrays appear to be the most emerging technologies, accounting for only 0.6% and 1.9% of the analyzed works, respectively.

In relation to the type of array (homogeneous or heterogeneous), only a few studies presented heterogeneous arrays: some of them focused on measuring different physical variables [52,62,68,94,96,114,206,217], while others measured the same magnitude either with different sensing technologies or with different types of sensors within the same sensing technology [61,63,92,98,101,102,225,306,307,346]. Most studies presented homogeneous arrays (95.3% of studies), while only 4.7% of studies focused on heterogeneous arrays.

Finally, Table 2 shows a summary of the advantages and disadvantages of each sensing technology to be considered by researchers in this field.

## 4. Sensor Array Applications

This section presents the different applications of sensor arrays found in existing studies.

### 4.1. Results of the Analysis

Table 3 shows the specific applications of the most recent studies on sensor arrays. Additional columns related to this item are also included: whether the sensor is wearable or environmental, the variable of interest measured by the array, and the experiments carried out for validation. The complete analysis for the rest of the studies found in this review is included in Table A2, Appendix B.

**Figure 25:**
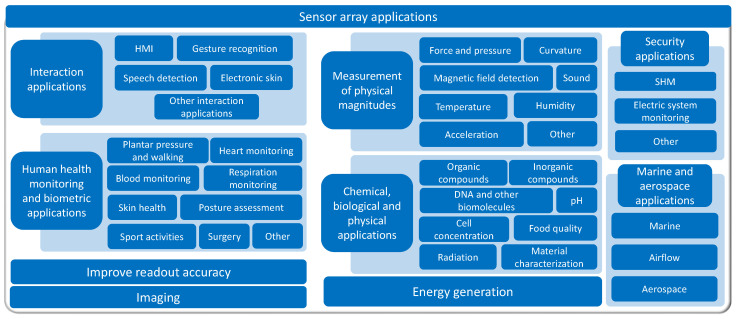
List of the applications of sensor arrays found in existing studies.

Focusing on the sensor array applications, Figure 25 provides an overview of the different approaches found. The following subsections describe them in more detail. All studies in Table 3 and Table A2 are explicitly cited in the corresponding subsection to which they belong. Studies presenting more than one sensor array application are cited in all associated subsections.

The following subsections classify and describe the different applications of sensor arrays.

#### 4.1.1. Interaction Applications

This sections considers studies on sensor arrays intended to facilitate human interaction with technology. The studies have been classified into the following groups: HMI, gesture recognition, electronic skin, and speech detection.

##### Human–Machine Interface (HMI)

**Figure 26:**
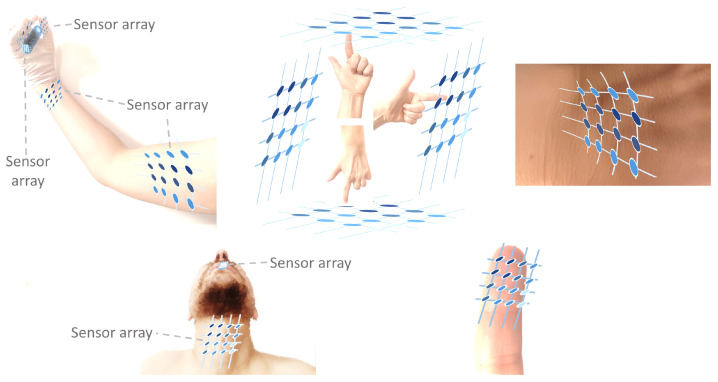
Schemes of interaction applications: human–machine interface (**top left**), gesture recognition (**top center**), electronic skin (**top right**), speech detection (**bottom left**) and other interaction applications (**bottom right**).

HMI sensor arrays allow people to interact with computers [326] or facilitate the display of information [142] (Figure 26). In this sense, Yeom et al. [137] developed a wrist-mountable keyboard composed of an array of pressure-sensitive thin-film diodes. Similarly, Tang et al. [208] developed and tested a wearable inductive keyboard attached to a subject’s arm. Li et al. [30] developed a triboelectric keyboard that, using convolutional NNs (CNNs), was able to identify keystroke signals as a cybersecurity measure. In this sense, Zhang et al. [44] developed a handwriting recognition system based on a 2-by-2 triboelectric array. It was able to distinguish the original writer from several forgers with good accuracy by applying various ML algorithms. Xiang et al. [305] designed a speech detector that allowed for turning lights on and off. Using inductive technology, Yeh & Fang [210] created a joystick based on a triaxial force detector. Xie et al. [163] developed a triboelectric/capacitive sensor array to enhance immersion in mixed reality environments. In turn, capacitive sensor arrays were widely used in HMI applications (tactile devices, keyboards, etc.) [154,155,157,162,169,172,176,182,185,195].

HMI sensor arrays can also be multifunctional. In this sense, Lee et al. [62] developed a wearable wristband consisting of a LED interface and a PVDF speaker. The system also measured temperature and UV radiation, displaying the results on a home-made LED screen. Wu et al. [114] fabricated a force-sensitive glove using a Velcro sandwich structure to measure temperature and illumination. In turn, Booth et al. [276,277] manufactured a piezoelectric wrist array for gesture recognition. Also in the field of gesture recognition, Esposito et al. [53] developed a wearable video game controller using a force-sensitive resistor (FSR) array. Yan et al. [315] presented a multi-touch laser-induced graphene triboelectric device capable of detecting letters drawn on it. In this regard, several studies developed non-contact systems [181,188].

It is also worth noting that studies of sensor arrays for HMI applications can use different sensing technologies such as piezoelectric [271,275], resistive/piezoresistive [46,47,51,54,55,57,59,73,88,90,114], transistor-based [76,221,268], triboelectric [315,316,319,328,331] or inductive [209].

##### Gesture Recognition

Gesture recognition is a basic technique for HMI (Figure 26). This is a novel solution for keyboard-less systems, where the user can control the computer by making hand gestures [316]. A review on gesture recognition and motion detection using transistor arrays can be found in the work of Jang et al. [368].

AI techniques are commonly used in gesture recognition applications [329]. In this sense, Liu et al. [148] presented a capacitive array to detect the direction of movement of the hand. They used a neural network (NN). Similarly, Esposito et al. [53] presented an FSR piezoresistive and transistor-based array for motion sensing. In the same way, Matsuda et al. [89] also performed hand gesture recognition but using pressure-sensitive sensors mounted on the hand. In that case, the sensors were fabricated by themselves. Khatoon et al. [213], performed gesture recognition using inductive stripes that correspond to the five fingers. Different gestures were repeated to train a machine learning (ML) algorithm that classified them. Abbasnia et al. [214] detected gestures made with the elbow. They used a wearable inductive sensor array together with an ML algorithm that allowed gestures to be classified into several classes. Likewise, Wang et al. [314] developed a gesture recognition system with a piezoelectric array based on temperature sensing.

Gesture recognition applications are useful in several fields. For example, Yang et al. [68] presented a smart tennis racket, which distinguished different types of strokes also using ML algorithms; Yan et al. [40] developed a wearable sign language interpreter using triboelectric arrays and ML; and Mclaren et al. [186] presented a capacitive array to analyze affective touch properties.

##### Electronic Skin

Electronic skins (e-skins) mimic human skin functionalities (for example, measurement of pressure or temperature [10,52,60,94,142,145,154]) to provide some machines with these sensing capabilities [260] (Figure 26). In this regard, Shin et al. [222] developed a micro-transistor array device that obtained high-resolution maps for small pressure values. Xue et al. [94] proposed a multifunctional IGZO-TFT transistor–thermistor array for reading pressure and temperature simultaneously. Ren et al. [95] developed a temperature sensor array based on organic FETs that was used as a body temperature sensor. Meanwhile, Golezar et al. [65] presented a 4-by-4 pressure-sensitive resistive sensor array with a wide measurement range. It was suitable for use as an e-skin. Other studies [47,64,65,71,174,176,185,191,246,322] focused on measuring pressure or force on small scales to develop e-skins, while some works investigated self-healable materials suitable for e-skins [20,37,76].

Several works have developed e-skins for a wide range of applications. Sim et al. [264] presented a piezoelectric array to detect pain sensation on human skin. Similarly, Ouyang et al. [85] developed a micro-pressure sensor array and embedded it into an artificial fingertip to provide the robot with enhanced tactile recognition capability compared to that of humans. Also in the field of robotic sensing, Ergun et al. [164] proposed a capacitive array that allowed a robot to grasp an object before grabbing it. It predicted whether the object would fall off in the grab. Similarly, Liu et al. [184] presented a capacitive array to detect types of materials with high accuracy from grasping objects with a robot. Other studies developed sensor arrays to provide robots with tactile sensing [35,146,190].

Luo et al. [142], Ma et al. [175], and Jang et al. [322] proposed a wearable glove that measured how objects were held and the pressure exerted on them. Lee et al. [62] developed a LED array that displayed information about different biosignals registered by other sensors (temperature and UV light) that were also included in the array. Meanwhile, MXene e-skins were reviewed by Chen & Wu [20], concluding that elasticity, flexibility, and self-healability are useful aspects. That work showed that MXene can be mixed with other materials (PVDF, Ag, polyurethane foam, tissue paper, etc.) to achieve different pressure ranges and sensitivities. Sensor arrays made from MXene can measure a wide range of parameters, such as pressure, temperature, force, or humidity.

Another set of studies presented applications derived from e-skins. In this sense, a sensor array was placed on a subject’s neck to detect its movement and deduce speech patterns [159,160,250]. Niu et al. [158] developed a multipurpose array applied to the detection of blinking, speech, and swallowing. Similarly, the work of Jeong et al. [250] detected swallowing with a piezoelectric sensor. The same task was carried out in the work of Xu et al. [162] using a high-resolution capacitive sensor array. Other studies also detected blinking using pressure arrays based on different technologies [87,158,329]. In turn, Verma et al. [84] implemented a CMOS silicon-based e e-skin designed for integration into prostheses. Similarly, Chang et al. [327] also integrated a sensor array into a prosthesis for gait phase detection.

##### Speech Detection

Speech detection is an application of sensor arrays that can be achieved in several ways (Figure 26). Different approaches can be found for the state-of-the-art. First, several studies [42,47,87,145,158,159,160,245,250] placed pressure arrays on the neck, recording the pressure patterns that allowed the distinction between different words. Second, pressure patterns were obtained from air exhaled through the mouth using a capacitive sensor array [92]. Third, since sound is a vibrating signal, it was also measured using piezoelectric sensor arrays [290,305] or high-sensitivity fiber-optic arrays [339].

##### Other Interaction Applications

Other studies also focused on interaction applications (Figure 26). Capacitive sensor arrays were used by Jeon et al. [165] to perform fingerprint recognition, which is a typical task performed on smartphones as part of biometric verification. Another interaction application is robot control. Thus, the works of Son et al. [359], and Géron et al. [360] used a Hall effect array to control the position of a small robot on a surface. The position was extracted using tracking algorithms.

#### 4.1.2. Human Health Monitoring and Biometric Applications

A common application of sensor arrays is human health monitoring. Studies in this category have been classified according to the specific monitoring performed: plantar pressure and walking assessment, heart, blood, and respiration monitoring, skin health, posture assessment, sport activities, surgery, and other health-related applications.

##### Plantar Pressure and Walking Assessment

**Figure 27:**
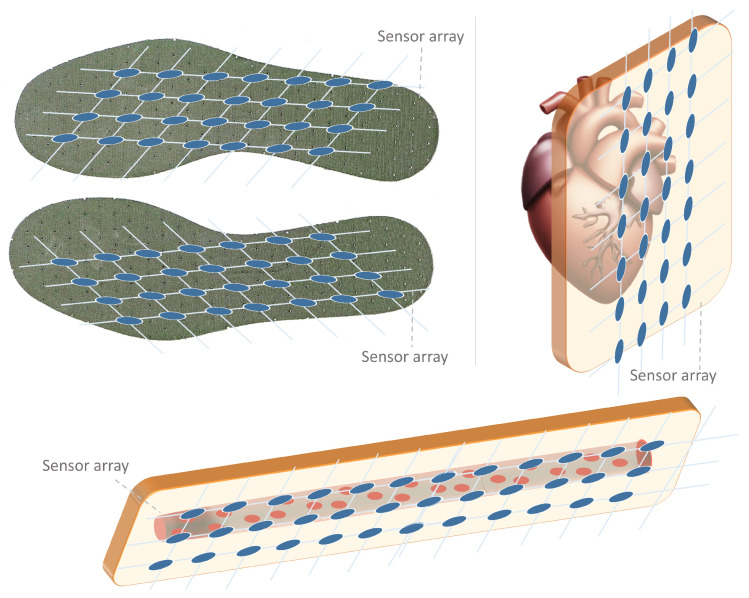
Schemes of plantar pressure and walking assessment (**top left**), heart monitoring (**top right**) and blood monitoring (**bottom**).

Several studies used sensor arrays to measure plantar pressure. This is a common application in health monitoring (Figure 27). Plantar pressure can be used to assess gait [245,248]. For example, Aqueveque et al. [153] developed a shoe-integrated capacitive sensor array to measure gait speed. This array communicated wirelessly via Bluetooth with the processing system. In this sense, Muzaffar & Elfadel [74] developed another portable array to measure the force of the foot on the ground. A similar approach was taken by Lei et al. [87]. In this regard, Fuh et al. [259] proposed a piezoelectric sensor array that can be integrated inside a shoe, while Chen et al. [369] designed a trimmable and customizable resistive sensor array. The originally designed array was trimmed to fit the foot size. Other studies also presented foot-worn sensor arrays [331], and some of them applied AI algorithms for gait analysis [39,45,255]. In this sense, Chang et al. integrated sensor arrays into prostheses for gait monitoring [327], while other studies used long PSMs for that purpose [108,144].

Other works were oriented towards pressure maps. In this regard, Wang et al. [324] performed digital detection of plantar foot pressure with a home-made wireless triboelectric sensor. Yang et al. [325] also used a triboelectric array to monitor plantar pressure in human subjects. Martinez et al. [140] studied the propagation of uncertainty in pressure values in a 16-by-16 PSM. Lee et al. [323] used a 3D printed foot model to record plantar pressure distribution with a triboelectric array. Other studies also used foot models to test new algorithms or electronic designs for measuring plantar pressure [104,105,106,259,325]. Alternatively, Medrano et al. [110] simulated plantar pressure in a 16-by-16 PSM using a mathematical approximation of the pressure distribution.

##### Heart Monitoring

Several studies focused on heart monitoring (Figure 27). On the one hand, Nagayama et al. [282] tested an organic piezoelectric sensor array for monitoring heartbeat phases. For that, they used a 3D heart model. Similarly, Xu et al. [284] presented a piezoelectric sensor array to measure pulse wave velocity (PWV). On the other hand, Qu et al. [309] developed an FMPE sensor array with potential applications in magnetocardiography. Also in the field of piezoelectric arrays, Zalar et al. [244] performed ballistocardiography using an large-area environmental sensor placed on a chair or bed.

Sensor arrays have also been used for heart rate monitoring. In this regard, several studies [92,143,150,159,177] used capacitive sensor arrays for heart rate measurement. Similarly, one of the biosensor arrays proposed by Ibrahim & Jafari [366] measured bioimpedance through blood channels in the wrist to obtain the pulse wave with good accuracy. In this sense, Lin et al. [246] used a piezoelectric array to measure heart rate in the neck artery. Tian et al. [283] attached a piezoelectric array to an imitated heart, allowing its movement to be measured at different points. Similarly, Liu et al. [257] developed a smart wearable piezoelectric sensor array that predicted the heart rate waveform in good agreement using an NN. Likewise, Ren et al. [224] proposed a triboelectric/transistor array to measure arterial pulse. Smart wristbands were also manufactured to monitor heart rate using high-sensitivity sensor arrays of different technologies: piezoresistive [46,47,87], capacitive [37], triboelectric [329], or piezoelectric [285].

##### Blood Monitoring

The study of Kundu et al. [220] presented a medical opto-sensor for the measurement of viruses and antibodies in blood using optical techniques (Figure 27). In turn, Li et al. [32] presented a fiber-optic array for the study of hemodynamics in live mice. Three parameters of vascular dynamics were detected: vessel density, hemoglobin concentration, and vessel width.

##### Respiration Monitoring

**Figure 28:**
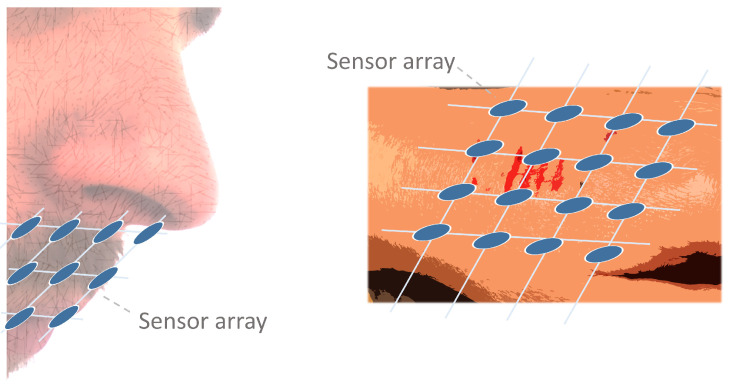
Schemes of respiration monitoring (**left**) and skin health (**right**).

Sensor arrays were also used for respiration monitoring (Figure 28). In this regard, several studies placed the sensing elements under the nose to monitor breathing [159,202]. A detailed review on respiration sensors can be found in the work of D’Amico et al. [16]. Tamiziniyan & Febina [287] presented a sleep monitoring solution. They used a piezoelectric sensor array to measure OSAs. Their system also incorporated vibrating motor disks that could wake up a patient to restore normal breathing. The studies of Feng & Su [288,312] developed a portable respiration monitoring array consisting of several concentric piezoelectric disks made of different metals. They were embedded inside a mask and measured respiration from variations in humidity. In this sense, VOC-sensitive sensors were also used to monitor respiration by measuring gas concentration or humidity [16,18]. In addition, Kuchmenko et al. [307] used commercial piezoelectric sensors to fabricate an array that could detect *Helicobacter pylori* with high sensitivity. For this purpose, the composition of exhaled air was detected. Similarly, Saqib et al. [156] proposed a capacitive sensor capable of detecting air blown over it. Other studies [143,145] presented capacitive arrays for respiration monitoring by placing the sensors on the abdomen.

##### Skin Health

Sensor arrays were also used in skin health applications (Figure 28). In this regard, Cheng et al. [56] developed a sensor system to detect pressure ulcers due to knee, elbow or neck bending. Kim et al. [330] developed a 3-by-2 triboelectric array installed in a bed, also for pressure ulcer prevention. Likewise, Kekonen et al. [367] presented a biosensor array to monitor the progress of wound healing. Its objective was to evaluate the post-surgery and rehabilitation processes. It was based on the generation of a low-intensity direct current to create an antimicrobial area due to the formation of hydrogen peroxide around the wound. In turn, John et al. [161] proposed a biodegradable capacitive pressure sensor made of silk. This could be used in future work to monitor the pressure on wounds.

##### Posture Assessment

**Figure 29:**
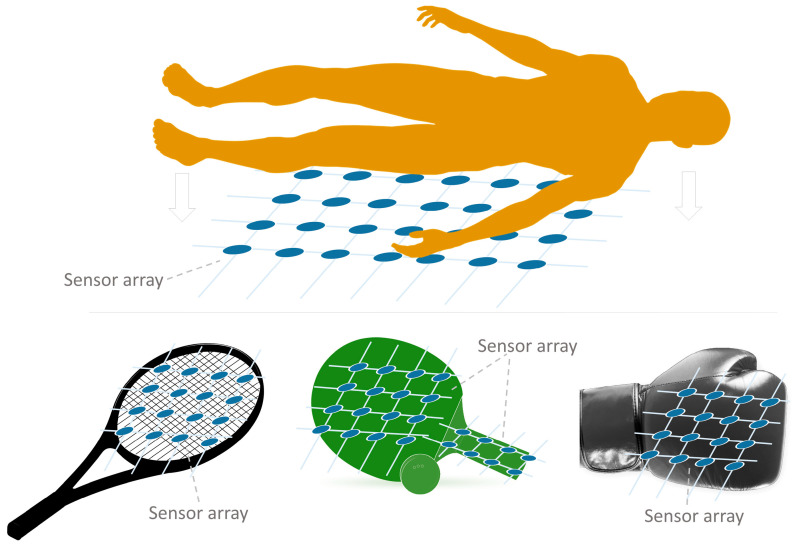
Schemes of posture assessment (**top**) and sport activities (**bottom**).

Some studies were aimed at improving posture when sleeping or sitting in a chair (Figure 29). For example, Gleskova et al. [168] developed a smart chair sensor array made of fabric (see Figure 55). Similarly, Park et al. [82] developed a pressure sensor array embedded in a car seat that allowed measurement of seating position during driving, among other applications. In addition, Islam et al. [58] developed a piezoresistive sensor array driven by a CMOS transistor array. These transistors formed a physical NN to detect whether a patient was correctly positioned while sleeping. If not, an alarm was triggered. In this way, pressure ulcers caused by poor sleeping postures could be prevented. Similarly, Hussain [81] presented an affordable smart mattress made with Velostat to monitor patients lying down. Husak et al. [77] proposed improvements in sleep posture monitoring using a calibration algorithm that reduced the influence of noise. Zhang et al. [78] monitored lumbar and spine movements using a 4-by-4 array of flexible, wearable, strain-sensitive resistive sensors. In this regard, Cen et al. [80] monitored elbow and shoulder joints angles using piezoresistive arrays and a CNN. A similar application was implemented by Zhen et al. [245] with a piezoelectric array. In turn, Tai et al. [86] monitored neck flexion to prevent cervical disc herniation.

##### Sport Activities

Several studies on sensor arrays were applied in the sports domain (Figure 29). In general, they were used to monitor physical activities. For example, Gao et al. [320] fabricated a TENG array for boxing monitoring. The system generated energy when hit. Meanwhile, Yamashita et al. created a piezoelectric sensor array to monitor the health of structures [261,270,274] that was also applied to the study of table tennis techniques. For this purpose, the array was placed on a smart table tennis racket [262,263]. Similarly, Xia et al. [254] also applied piezoelectric sensors for tennis racket stroke monitoring. Likewise, Yang et al. [68] developed a sensor array for a tennis racket located, this time, on the handle. This sensor was integrated with an IMU to read the acceleration curves. In this way, the type of stroke could be identified. Sun et al. [329] monitored table tennis but, in this case, placing a triboelectric array on the player’s fingers. In turn, Susac et al. [357] used an 8-by-8 Hall effect sensor array to monitor chess games. Zhang et al. [150] used a multi-purpose sensor to measure squat depth or arm flexion, and Zhou et al. [159] presented a similar system that also included other applications (HMI, respiration monitoring, voice recognition, etc.). In general, pressure sensor arrays can detect sports-related movements if attached to the human body [87,145].

##### Surgery

**Figure 30:**
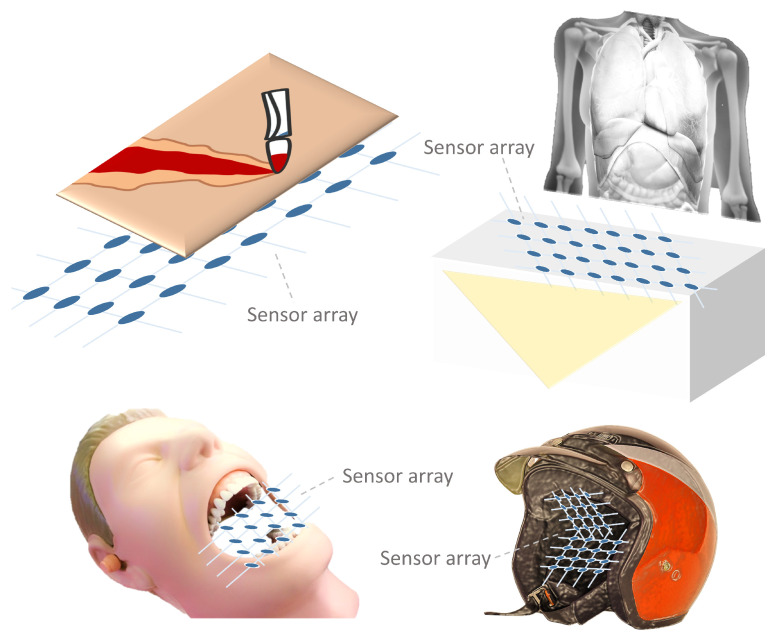
Schemes of surgery (**top left** and **top right**), bite monitoring (**bottom left**), and helmet impact safety (**bottom right**).

Several studies on sensor arrays were applied to surgery (Figure 30). First, Shin et al. [345] developed a tumor scanner using an AFCRS. Similarly, Angeli et al. [66] proposed an inkjet-printed resistive sensor array that could be used for medical instrumentation. Second, Hall effect sensor arrays are a promising solution for position tracking of surgical instruments, as low-frequency magnetic fields are not affected by biological tissues or water [358,359,361]. In this regard, Fischer et al. [361] presented a wearable Hall effect sensor array. It was used to track the position of surgical instruments under human tissues. These instruments had permanent magnets attached to them. In this sense, Baker et al. [346] used a fiber-optic array to track the position of an ultrasonic needle. For this purpose, the fiber-optic beam was embedded with the needle. It was immersed in a tank of water for testing. Third, piezoelectric sensors can be implanted as biomedical devices. Feng & Chen [303] placed an array of invasive 2-by-2 PZT/Ti piezoelectric sensors on a bone for knee surgery. In the field of surgical robots, Ye & Ju [38] proposed a pressure capacitive array capable of providing a pressure map of organ models by palpation.

##### Other Health-Related Applications

Several studies have presented other types of human health monitoring applications (Figure 30). In this sense, Liu et al. [148] presented a modern rehabilitation activity board. Tai et al. [70] manufactured an array of nano-piezoresistive sensors to be placed on human teeth to measure the force of the bite. Kim et al. [355] identified antigens using fiber-optic sensor arrays with nanodome structures. These antigens are representative biomarkers of thyroid cancer.

A sensor array can also be placed on the neck to monitor movements associated with swallowing and prevent choking [158,286]. In fact, swallowing monitoring was performed in several studies [42,158,162,250,286]. Sensor arrays were attached to the Adam’s apple in order to detect the movement of the neck muscles from the pressure exerted on them. Finally, Kim et al. [243] placed a three-sensor piezoelectric array next to the eye for eye tracking. Abnormal eye movements may be related to various neurological disorders.

#### 4.1.3. Measurement of Physical Magnitudes

Several studies do not focus on a specific application but on the measurement of physical magnitudes. Sensor arrays in this category are typically validated against a gold-standard device (see Section 5).

##### Force and Pressure

**Figure 31:**
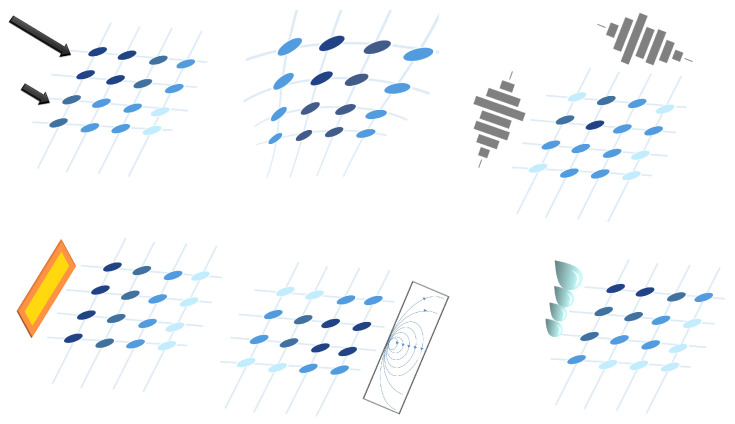
Schemes of measurement of physical magnitudes: force and pressure (**top left**), curvature (**top center**), sound (**top right**), temperature (**bottom left**), magnetic field detection (**bottom center**), and humidity (**bottom right**).

Several studies focused on improving pressure measurement (accuracy, sensitivity, measurement range, etc.) [10,62,64,65,67,70,72,79,83,140,142,147,148,149,151,154,155,157,161,165,167,172,176,183,188,208,212,222,241,246,248,251,252,253,260,264,279,324,325,326] (Figure 31). For example, Yeom et al. [137] presented a diode-based sensor array for low-range pressure measurement. It is also common to consider different types of force (deformation, shear, etc.). In this regard, various studies [88,178,211,265,266,267] used three-axis force sensor arrays for the detection of normal and shear forces. Kim et al. [61] developed a multi-technology (resistive + capacitive) sensor array with a woven structure designed to measure strain. It also explored self-harvesting using triboelectric elements. Strain was also measured in [269] using a piezoelectric oscillator. Meanwhile, Fernandes et al. [178] presented a capacitive array based on capacitance changes between non-parallel plates to measure both normal and shear forces. Gao et al. [223] developed a novel MG-FET transistor array that changed the electrical characteristics of the transistors due to displacement. Omary et al. [247] developed an online pressure map detector using a piezoelectric sensor array based on $MoS_{2}$. It could be read from any device connected to the Internet, such as a smartphone. Su et al. [76] studied the force and pressure response curves of a transistor-based array. Finally, Wang et al. [48] optimized force detection by implementing new structures that transmitted the force to the sensing element more efficiently.

##### Curvature

Several studies focused on the measurement of curvature (Figure 31). In this sense, Zhang et al. [334] presented a fiber-optic array for curvature measurement that was also temperature-sensitive. The array consisted of several bumps connected in series with a fiber beam. Each bump was considered as a different sensor in the array. Liu et al. [352] applied the measurement of curvature in aircraft manufacturing processes (see also Section 4.1.6). Jeon et al. [55] proposed a curvature sensor based on cotton yarns covered with a CNT solution. The array changed its resistance when bent. Curvature measurement is also of interest for damage control in structures (see Structural Health Monitoring (SHM)). In this regard, Wang et al. [297] used a sound-sensitive piezoelectric array to measure the curvature of an aluminum plate.

##### Sound

Several studies on sensor arrays focused on sound-related applications (Figure 31). Piezoelectric sensor arrays were used in BAW and SAW applications. On the one hand, Xu et al. [311] performed a detailed analysis of a BAW resonator. On the other hand, Holeczek et al. [293] used ultrasound to detect defects on a thermoplastic surface. In addition, fiber-optic arrays were also used to measure ultrasound [1] or detection of sound sources [339,340]. Fiber-optic arrays have been frequently used in ultrasound hydrophones [336,341,342].

##### Temperature

A set of sensor array studies focused on temperature-related applications (Figure 31). Fan et al. [97] tested a high-temperature sensor array (from 50 °C to 120 °C) based on standard Pt100 RTDs. Meanwhile, Zhang et al. [334] studied the effects of temperature on fiber bumps connected on the same line, finding a linear decrease in transmission loss as temperature increases. Also in the field of fiber-optic arrays, Wang et al. [337] monitored the temperature inside a semi-enclosed truck under four different conditions, which included sudden temperature perturbations. In turn, Sim et al. [264] developed a piezoelectric array that was capable of generating a pain-like electrical signal when a hot object touched the sensor.

##### Magnetic Field Detection

Several sensor array studies focused on magnetic field-related applications (Figure 31). In this sense, Kim et al. [5] presented an on-board piezoelectric sensor array capable of measuring the magnetic field in all three spatial dimensions. For this purpose, each sensor acts as a coil, so that its relative orientation with respect to the magnetic field enables multiaxis detection. Similarly, Qu et al. [309] developed a magnetic field sensor array applicable to magneto-cardiography. Nhalil et al. [364] presented a Hall effect array with high resolution (pT) and very low EMN. Hall effect sensors are ideal for magnetic field detection [364,365]. Finally, Näf et al. [103] performed magnetoresistive sensor array readout with built-in carrier signal suppression.

##### Humidity

Humidity measurement was performed by Sun et al. [310] and Feng & Su [312] using piezoelectric arrays (Figure 31).

##### Other Applications Related to Physical Magnitudes

Acceleration measurement was performed in a few papers, mainly those using IMUs. Gutiérrez et al. [335] proposed an FBG-IMU to monitor flexible structures. Yang et al. [68] coupled an IMU to a tennis racket to measure acceleration in three axes while detecting the grip force using a piezoresistive array. In the work of Nilsson & Skog [13], a literature review on inertial sensor arrays was developed. The angular position was measured in the work of Pu et al. [179] and in the work of Liu et al. [352] using capacitive and fiber-optic arrays, respectively.

#### 4.1.4. Chemical, Biological and Physical Applications

Sensor arrays that respond to chemical and biochemical compounds are included in this section. They have been categorized into the following groups: organic compounds, inorganic compounds, DNA, and other biomolecules, pH, cell concentration, food quality, radiation, and material characterization.

##### Organic Compounds

**Figure 32:**
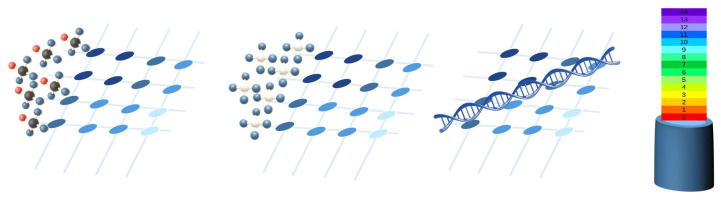
Schemes of chemical and biological applications: organic compound detection (**first**), inorganic compound detection (**second**), DNA and other biomolecules (**third**), and pH measurement (**fourth**).

Several studies focused on the detection of organic gases or biological matter (Figure 32). chemiresistive sensor arrays are an option for this task [7,98,100,102,111,112,118]. In this regard, Park et al. [112] developed a chemiresistive sensor array using commercial components and thin-film resistors. Moreover, Wei et al. [7] printed a flexible resistive sensor array to measure methane and ethanol concentration. Chowdhury et al. [111,118] proposed circuit arrangements using transistors and diodes to improve the accuracy of resistive chemical arrays. In turn, Wijaya et al. [98] detected organic VOCs through a custom fabricated MOS sensor array. The data was sent via Wi-Fi to a PC using the Arduino platform. Finally, the work of Rath et al. [18] reviewed different chemiresistive sensor arrays.

Capacitive sensor arrays are also a common approach for VOC sensing, for example, using CMUTs [203] or EISCAPs [200]. In [206], VOCs are detected with polymer-based capacitors. Tabrizi et al. [193] used a CMOS capacitive array to detect the concentration of ethanol in water droplets.

Other types of sensor array can be used to detect organic gases. One possible approach is to use piezoelectric sensor arrays whose oscillation frequency depends on the gas concentration in the air. Länge [12] presented a list of commercially available piezoelectric e-noses. Li et al. [2] simulated and fabricated a piezoelectric sensor array for VOC detection. The system could identify various types of VOCs by measuring the cantilever oscillation frequency. High detection and classification accuracy was obtained by applying ML algorithms such as decision trees or NNs. In turn, Zou et al. [235] proposed a transistor-based array to detect organic compounds in air. They could identify an unknown gas with high accuracy. Zhai et al. [236] detected ${\mathrm{NH}}_{3}$ in a similar way. In this sense, Liu et al. [238] developed an ${\mathrm{NH}}_{3}$, H_2_S, HCHO gas sensor array with CNT transistors. They applied ML algorithms for classification, which achieved 100% accuracy. Hsu et al. [226] developed a transistor array that detected phosphate buffered saline solutions, and Yuan et al. [233] proposed an ISFET array that was sensitive to chloride ions. Tao et al. [237] detected three different antibiotics (ampicillin, amoxicillin, and kanamycin) with a three-electrode transistor array. They were able to recover them correctly even when mixed in milk, honey and other products. E-noses are a common application of transistor-based arrays [34]. In this sense, Duan et al. [308] detected VOCs using LWRs. VOLs were also detected using fiber-optic arrays and ML classification algorithms [356]. This was applied to detect antigens. In [306], VOCs diluted in water are detected and classified by Principal Component Analysis (PCA).

##### Inorganic Compounds

Several studies also considered non-organic gases [98,235] or inorganic nanoparticles [99] (Figure 32). In this sense, Yan et al. [17] reviewed fluorescent sensor arrays to detect metal ions diluted in water. They showed that fluorescent sensor arrays relied on different types of molecules to distinguish between various cations. ML classification techniques (such as PCA, linear discriminant analysis, etc.) were also used to help identify the particular cation from the array measurements. Similarly, Bhat et al. [225] monitored phosphate, nitrate and potassium ions in water with custom-fabricated transistor-based arrays. The drain current showed linear dependence with the concentration of phosphate ions ($R^{2}>0.991$). Similarly, Mishra et al. [101] distinguished Zn(II) and Cu(II) ions in solution by applying ML techniques. Synergistic effects between ions and sensors were analyzed and explained. Karschuck et al. [201] detected charged gold nanoparticles with a capacitive array. Also, Zhu et al. [194] placed six copper electrodes around an acrylic pipeline to detect particle concentration in two-phase flows. Finally, Park et al. [112] proposed a method to increase the accuracy of electrochemical thin-film resistor arrays.

##### DNA and Other Biomolecules

Detection of DNA and other types of biomolecules (such as proteins, RNA, etc.) can be achieved using different technologies (Figure 32). In the field of biosensors, Gao et al. [239] presented an array of G-FETs to detect DNA. In contrast, Hsu et al. [226] proposed HEMT in the form of micro-SD cards. Lai et al. [199] presented micro-DNA sensors fabricated with CMOS standard manufacturing processes. Likewise, in [200], gate-controlled EISCAPs were proposed for the detection of variations in analyte concentration. DNA hybridization could be detected with these devices. Arrays of BAW and SAW sensors have also been applied to the detection of biological molecules, DNA, or antibodies [12]. Li et al. [240] developed a dopamine-sensitive transistor array.

In turn, Tabrizi et al. [205] mathematically modeled a capacitive biosensor. Equation (4) shows the potential sources of error for a biosensor based on capacitive interdigitated electrodes.

(4) $$C_{IDE}=C_{C}+\Delta C_{NS}\left(t\right)+\sigma C\left(t\right),$$

where $C_{IDE}$ is the capacitance of the interdigitated electrode, $C_{C}$ is the parasitic capacitance inside the chip, $\Delta C_{NS}\left(t\right)$ is the variation in capacitance due to environmental conditions (ECs), such as temperature, pH, etc., and σC(t) is the time-variant capacitance sensed in the target [205].

##### pH

Another application of sensor arrays is pH measurement (Figure 32). Transistor-based arrays are common in industrial applications for acidity measurement. In this sense, Li et al. [232] fabricated a custom ISFET transistor array with MOS technology, showing good repeatability and stability. Ha et al. [217] also integrated several ISFET transistors with a temperature-dependent diode and FET for multiparameter readout. Likewise, Karschuck et al. [201] used EISCAPs to measure pH. Finally, Melzer et al. [234] developed a CNT transistor array for enzyme detection by measuring acidity.

##### Cell Concentration

**Figure 33:**
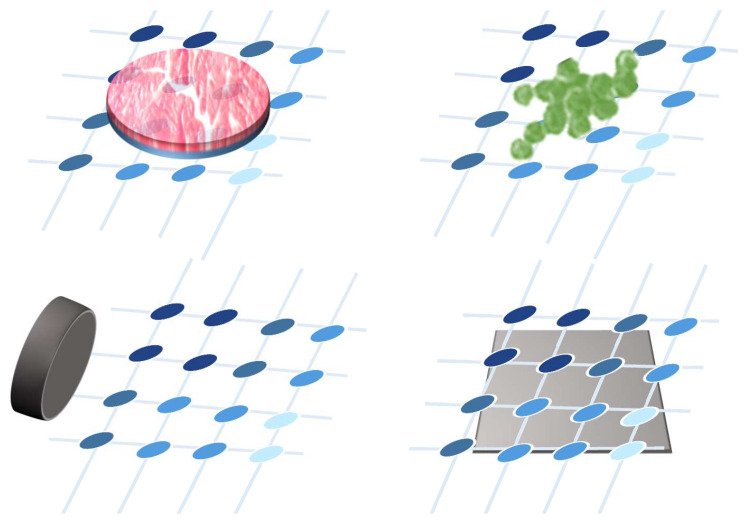
Schemes of chemical and biological applications: food quality (**top left**), cell concentration (**top right**), radioactivity (**bottom left**), and material characterization (**bottom right**).

Measurement of cell concentration is necessary to monitor the growth of cell populations and is useful for disease diagnosis (Figure 33). In capacitive sensors, the process is as follows: changes in cell concentration result in different ionized cell membranes, leading to charge changes on the surface of sensor arrays when placed on it. Thus, Nabovati et al. [196,197] fabricated a customized CMOS chip to count the number of cells in a given area. They presented a capacitive array that measured the capacitance between interdigital electrodes when cells were placed on them. The surface charge of the cell became polarized due to the existence of an electric field, resulting in a change in capacitance: the more cells there were in the electrode, the higher the capacitance was. A similar approach was presented by Lai et al. [198]. Wijaya et al. [98] measured bacterial growth indirectly by quantifying gas concentration.

##### Food Quality

Kumar et al. reviewed food quality sensor arrays [19]. They analyzed technologies such as e-noses and techniques such as neural networks applied to food quality (Figure 33). Most of the studies presented in the section entitled Organic Compounds can also be applied to food quality assessment, as they are based on the same principles [12,18,19,98,200,233]. In addition, the detection of metallic ions is also common in food quality assessment due to the associated health risk [17]. In this regard, Demori et al. [96] developed a dual resistive-capacitive sensor array that can be used for this purpose.

##### Radiation

Wavelengths emitted by radioactive materials can also be detected by sensor arrays (Figure 33). In this way, Jia et al. [219] provided a diode-based radiation sensor array. The array was intended for high-energy physics research centers, such as the European Organization for Nuclear Research (CERN), or for astrophysical applications. The study also provided a custom application-specific integrated circuit for the array. Meanwhile, Park et al. [353] used a fiber-optic sensor array to locate the source of radiation. A horizontal sensor array was moved on a vertical guide to generate a two-dimensional thermal map of the intensity of the radiation. This map was filtered to provide a more accurate position of the radiation source. A gamma-ray source was used for the experiments.

##### Material Characterization

Some studies presented sensor arrays to measure material properties (Figure 33). In this sense, Liu et al. [275] developed an e-skin to measure roughness as a way to mimic human perception of textures. In turn, Holeczek et al. [293] used a microphone array to generate custom ultrasonic waves propagating in thermoplastic materials. The objective was to detect defects on the surface of the composite. Meanwhile, Kim et al. [267] presented a piezoelectric sensor array to measure both elasticity (Young’s modulus) and force. Similarly, Sun et al. [333] presented a fiber-optic sensor array to study the response of sandstone to strain forces. Finally, Nagai et al. [295] developed a piezoelectric tactile sensor to measure viscosity and hardness in materials.

#### 4.1.5. Security

Sensor arrays can provide multipoint measurements. This can be useful in different fields. One set of sensor array studies focused on structural health monitoring and safety of electric systems.

##### Structural Health Monitoring (SHM)

**Figure 34:**
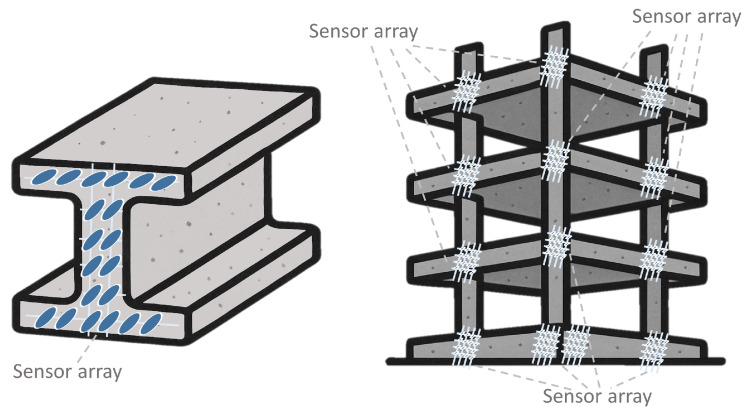
Scheme of structural health monitoring.

Structural health monitoring deals with damage to various types of structures, typically buildings, bridges, beams, etc. (Figure 34). On the one hand, piezoelectric sensor arrays can be used, which can function both as microphones and loudspeakers. Sound propagation within materials can help quantify and locate damages, so BAWs and SAWs may be suitable arrays. Several studies adopted this approach [4,292,294,296,297,298,299,300,301,302]. The MUSIC (MUltiple SIgnal Classification) algorithm is a typical damage detection method [297,299,301,302]. Various studies [261,270,272,273,274] measured the deformation in stainless steel plates in the cantilever position, so that the amplitude of the piezoelectric signal depended on the bending of the plates. In addition, Bae & Lee [291] performed surface damage detection using multiwave propagation imaging. Similarly, Barzegar et al. [304] used PZT sensors to map damage in composite lap joint, while Wang & Zong [301] studied impact locations in composites with this type of arrays. However, capacitive sensor arrays were also used in SHM. In this sense, Yan et al. [170] performed crack pattern detection in concrete. For that, a row of capacitive sensors was placed along a concrete beam. Kong et al. [171] detected cracks in steel bridges using a home-made capacitive array. Cracks were also detected by Shi et al. [280] around aircraft bolts. Yao et al. [152] fabricated several capacitive sensors with different curing conditions to locate both the position and the value of a point charge. CACT can also be applied to locate damage in composite materials [36]. Meanwhile, Santamato et al. [31] monitored strain inside train rails using FBGs. Finally, in the field of inductive arrays, Faria et al. [215] proposed an inductive array that detected imperfections in 3D-printed metal parts.

##### Electric System Monitoring

**Figure 35:**
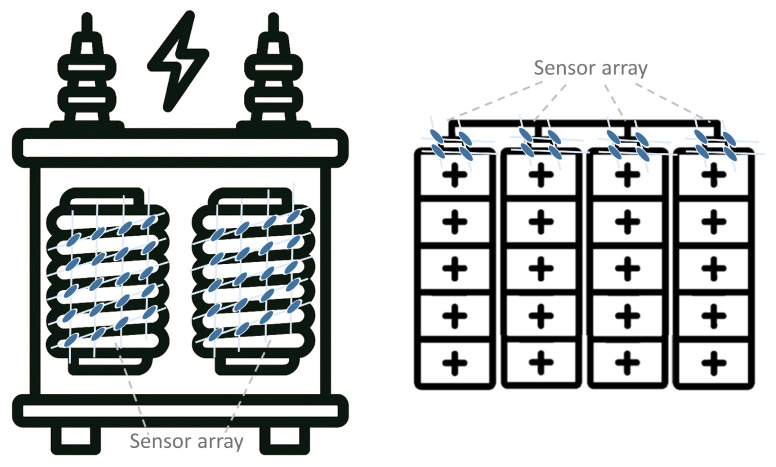
Scheme of power system monitoring.

Safety in energy distribution and storage systems is a topic of interest in research on power systems (Figure 35). In this sense, Wang et al. [9] simulated an array of capacitive voltage sensors to detect and quantify transient voltage characteristics in transformer windings. Transients were detected at various points on the windings by placing several capacitive sensor arrays. In this sense, Shu et al. [281] measured vibrations inside power transformers. In turn, Luca et al. [362] used a Hall effect array to measure the imbalance of battery discharge. A similar application was carried out by Tang et al. [363], who, as a novelty, arranged the sensor array in an ellipse, which reduced the space required for installation. In this sense, Hu et al. [249] also measured the health of lithium-ion batteries. Meanwhile, Zhang et al. [343] used a Fabry–Perot fiber-optic sensor array to measure the insulation efficiency in power transformers. Insulation failures caused the fiber-optic sensor array to detect ultrasonic waves. Liu et al. [344] implemented a similar application, also with fiber-optic arrays. They applied a MUSIC algorithm for fault detection. In a different field, Veske et al. [173] proposed a capacitive pressure array to control stress in MEMS packaging due to temperature or external forces.

##### Other Security Applications

Other security applications were addressed by Li et al. [258] and Ren et al. [350]. The first study developed a system to measure the impact on a safety helmet, while the second study presented a fiber-optic anti-vandalism surveillance system.

#### 4.1.6. Marine and Aerospace Applications

This subsection includes marine, airflow, and aerospace applications of sensor arrays.

##### Marine Applications

**Figure 36:**
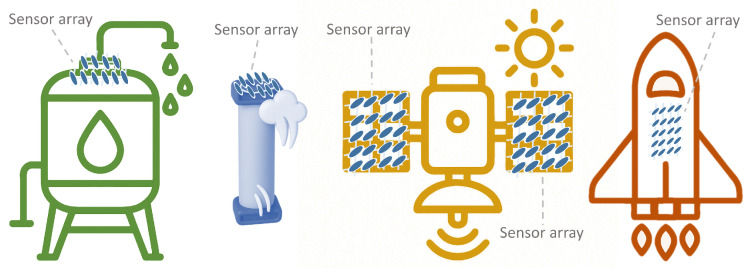
Schemes of marine (**left**), airflow (**center-left**) and aerospace (**center-right** and **right**) applications.

Several studies focused on marine or fluid applications (Figure 36). In this regard, Dusek et al. [3] developed a piezoresistive sensor array to measure near-field water flow around a marine vehicle. The objective was to improve vehicle performance. Similarly, Liu et al. [41] measured the velocity of an unmanned underwater vehicle with a triboelectric array. It was based on seal whiskers. At the same time, they could detect obstacles with ML algorithms. In this regard, Wei et al. [242] developed a piezoelectric array to study the pressure distribution around a model vehicle. Riccioli et al. [256] proposed a piezoelectric sensor array to measure transient pressure stresses. A pressure pulse generator was placed at the bottom of a water tank. The array was placed at the top. The sensor array could measure the pulse that propagated through the water. Finally, Elzaidi et al. [207] detected several underwater ice layers using a NN. The research was applied toward the formation of sea ice.

##### Airflow Applications

The measurement of ECs, such as wind speed and direction or atmospheric pressure, is also useful for various applications. In this sense, Ko et al. [317] developed an array of self-powered curved-flap triboelectric sensors. As this array was circular, it could measure the wind speed and direction omnidirectionally. Zhang et al. [43] also proposed a wind-sensitive triboelectric sensor array, which used a permanent neodymium magnet to enhance its sensitivity. Also in the field of triboelectric arrays, Wang et al. [102] used wind-sensitive TENGs to supply power to a chemiresistive array. In turn, Bian et al. [6] proposed a three-dimensional wind sensor array mimicking the airflow receiver of a cricket to detect the wind direction through piezoelectric technology. In addition, piezoresistive sensor arrays were also used to monitor air pressure. In this sense, Chen et al. [91] presented a fin-type piece with a piezoresistive element attached to it to measure the airflow velocity. Using four of these fins at the same time, the components *x* and *y* of the air velocity vector were obtained. Meanwhile, Su et al. [47] developed a piezoresistive sensor array that was sensitive to very low airflow speeds. Capacitive arrays have also been applied to monitor wind speed [37].

##### Aerospace Applications

Several studies on sensor arrays focused on aerospace applications (Figure 36). In this regard, Gutierrez et al. [335] performed structure monitoring in aerospace vehicles using a fiber-optic sensor array applied as an IMU. In the same way, Nilsson & Skog [13] highlighted several applications of IMUs for navigation, ballistic platform guidance, and platform control. In turn, Liu et al. [352] used an FBG array to monitor and correct aerospace component assembly processes. Similarly, Fonseca et al. [278] performed automatic regulation using piezoelectric sensor arrays. The objective was to control the vibrations of the satellite solar panels. Meanwhile, Zhang et al. [69] designed and integrated a piezoresistive sensor array to measure pressure in radiosonde systems. Mendoza et al. [351] proposed an instrumented broadcloth canopy to monitor the strain stresses suffered by parachutes during deceleration in the reentry of space vehicles. Finally, Cong & Jing [289] monitored the axial flow in a real aircraft compressor. For this, piezoelectric PVDF sensors were developed.

#### 4.1.7. Improve Readout Accuracy

A subset of studies focused on improving the readout accuracy of sensor arrays. This was achieved mainly by designing new readout circuits or developing new readout techniques. Improving readout accuracy has been an active topic of research, especially in resistive and piezoresistive sensor arrays [8,50,51,72,75,79,93,96,97,99,104,105,106,107,109,110,111,112,113,114,115,117,118,119,120,121,122,123,124,125,126,127,128,129,130,131,132,133,134,135,136,138,139,140,141,370]. To a lesser extent, studies in this field can also be found in capacitive arrays [160,166,176,178,179,192,200,202,203,206], fiber-optic arrays [32,33,49,338,347,348,349], inductive arrays [210,211], transistor-based arrays [116,231], diode-based arrays [137], and piezoelectric arrays [290].

#### 4.1.8. Imaging

**Figure 37:**
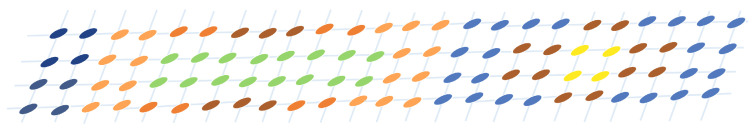
Scheme of imaging.

This section includes studies on sensor arrays capable of providing 2D or 3D maps of a given object or phenomenon (Figure 37). In this sense, Ma & Soleimani [188] developed a 4D capacitive image sensor array that contactlessly provided the position of an object on the sensor. A similar application can be found in [180]. Liu et al. [216] proposed an inductive array for non-contact UXO detection. Similarly, Tholin & Soleimani [187] used a planar capacitive array to detect objects non-invasively. This approach enabled multiple dielectric sensing with a single copper plate. Cao et al. [189] applied ECT to detect two-phase flows inside metallic pipes. ECT has been applied in several works [36,147,180,187,188,190]. In turn, Piron et al. [15] reviewed time-of-flight diode arrays, which could function as 3D imagers. In the same way, Jia et al. [219] also applied diodes for imaging, using core shell diode pixels. Those diodes allowed for high 2D resolution and a lower charge sharing effect. In this regard, Choi et al. [218] developed a photodiode array that was driven by transistors. As a novelty, the array operated without loss of properties under several bending cycles.

Transistor-based diodes were also used in the imaging field. For example, Tang et al. [230] presented a 2D sensor array based on light transistors, comparing its density and performance with that of a RAM. Chen et al. [227] used OTFTs to detect an image projected onto its surface using a light source. Similarly, Hu et al. [228] implemented a drawing surface, which acted as a display, using photoconductive transistors. Kim et al. [229] proposed a new type of photomemory transistor, improving the previous state-of-the-art.

In addition, fiber-optic arrays were applied to the development of handheld imaging devices [354]. They were also used for skin-mirroring applications. Finally, Yang et al. [1] performed sound imaging using a fiber-optic array sensitive to acoustic waves.

#### 4.1.9. Energy Generation

TENGs (see Section 3.1.7) and piezoelectric nanogenerator arrays can be used in low-power [63] or self-harvesting systems [253], due to their electrical generation properties. In this regard, Kim et al. [61] fabricated a triboelectric–capacitive–piezoresistive sensor array that generated power to measure forces. Similarly, Chen et al. [321] presented a micro 3 × 3 triboelectric array for force measurement. The output voltage of this array was fed directly to the analog-to-digital converter (ADC), via a multiplexer, so no power supply was needed for the main array. Meanwhile, Demori et al. [96] developed a mixed resistive and capacitive sensor array. The array sent information to the reader unit, as well as the power needed to operate, both via radio-frequency identification. Thus, these types of array are useful for IoT applications [313,318]. In this sense, Yang et al. [318] presented a self-powered triboelectric array targeting IoT applications. They proposed a smart traffic monitoring system for city streets. Finally, Ko et al. [317] studied the power generation capability of a wind speed detector in depth, demonstrating its self-powering capability.

### 4.2. Brief Conclusion of Sensor Array Applications

**Figure 38:**
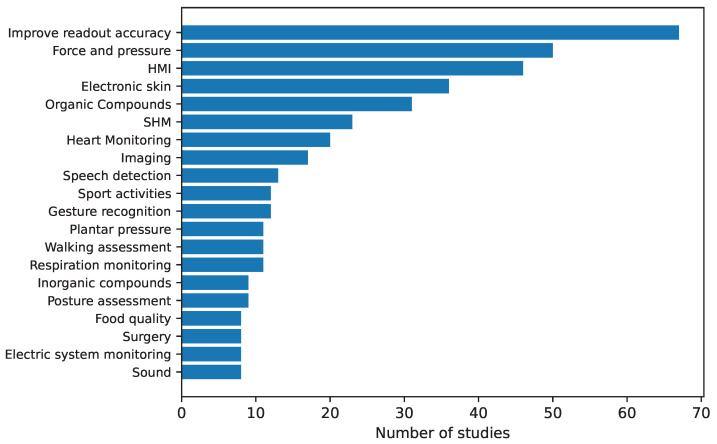
Number of sensor array studies by applications. Only the most frequent applications are shown.

Figure 38 shows the number of existing studies for each application. It can be seen that improving the readout accuracy is a major problem in sensor arrays, as most studies focused on this issue (18.6% of all works). In addition, pressure and force were the physical parameters most frequently measured by sensor arrays (13.6% of all works). Within interaction applications, most studies of sensor arrays investigated in the HMI domain (12.7% of all works). Regarding the human health group, heart monitoring and monitoring of sport activities were prevalent (5.5%, and 3.3% of all works, respectively). In turn, most of the chemical, biological, and physical sensor arrays were in the field of organic compound detection (8.6%), followed by inorganic compound detection (2.5%). It is also worth noting that a considerable number of studies addressed imaging and energy generation applications. Other applications of sensor arrays, such as blood or bite monitoring, appeared in isolated studies.

Table 4 represents the percentage of studies within each application that use the different sensing technologies. It can be observed that resistive and piezoresistive sensor arrays are applicable to a wide variety of fields, as they appear in a high percentage in almost all types of applications. Capacitive sensor arrays are also common and especially useful for interaction and imaging applications. Transistor-based arrays are of great relevance for chemical, biological and physical applications and in the field of imaging. In this regard, triboelectric sensor arrays are used in greater proportion for power generation and in marine and aerospace applications. Finally, piezoelectric sensor arrays are particularly relevant in human health monitoring, measurement of physical magnitudes, security, and marine and aerospace applications.

## 5. Validation Experiments

One required step in the design of sensor arrays is validation. The purpose of validation is to provide an objective metric of system performance.

### 5.1. Results of the Analysis

Several types of validation experiments have been proposed by researchers in this field. They can be broadly categorized into four groups: simulation experiments, tests with external machines or elements dependent on the specific array application, experiments that involve humans interacting with the sensor arrays, and laboratory experiments.

Table 3 analyzes the validation experiments performed by the most recent studies on sensor arrays. The analysis of the rest of the works can be found in Table A2, Appendix B.

**Figure 39:**
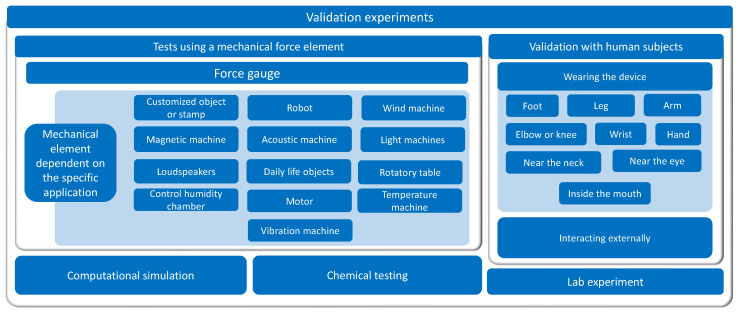
List of validation experiments contained in existing studies.

Focusing on the sensor array validation experiments, Figure 39 provides an overview of the different approaches found. The following subsections describe them in more detail. All studies in Table 3 and Table A2 are explicitly cited in the corresponding subsection to which they belong. Studies that use more than one type of validation experiment are cited in all associated subsections.

#### 5.1.1. Computational Simulation

This type of sensor array validation consists of simulated testing to obtain a measure of system performance. Several studies have taken this approach. A set of studies conducted simulations that focused on the electronics and engineering of sensor arrays. These were technical simulations. Liu et al. [195], Karbari et al. [290], Hasan et al. [105], Rashidi et al. [106], Warnakulasuriya et al. [75], Yang et al. [68], Yue & Moussa et al. [73], Zhao et al. [8], and Guorong et al. [4] performed circuit simulations to test the validity of their proposed circuit configurations. Moreover, many studies on sensor arrays used simulations to test the validity of new approaches to reduce the crosstalk problem in resistive sensor arrays [8,79,97,98,107,108,109,110,111,115,117,118,119,120,121,122,123,124,125,126,127,132,133,134,135,136,138,139,140,141]. This specific aspect of the literature has been previously reviewed until 2016 in the work of Wu et al. [370].

Chattopadhyay & Chowdhury et al. [177] simulated pressure on the sensor array to validate the proposed capacitive measurement circuit. Poghossian et al. [200] simulated the capacitance–voltage curves of a novel capacitive sensor array in Python. Shen et al. [92] simulated the wrist pulse in COMSOL to test the behavior of their capacitive array under this stimulus. Liu et al. [352] and Chong et al. [60] performed mechanical simulations based on finite element analysis (FEA) to evaluate the deformation of a given mechanical element attached to the sensor or in the sensor array itself. Jain & Bhatia [90] simulated the deformation in a cantilever to increase the bending of a coupled tactile sensor array. In the work of Hessel et al. [231] simulations were performed to test different geometries and sizes of cantilever array sensors. Arndt et al. [279] performed a FEA simulation to optimize the sensor layout and decrease coupling between neighboring columns. Similarly, Wang et al. [296] used the same technique to determine the most appropriate configuration of a piezoelectric sensor array to assess the severity of damage in structures. Lin et al. [246] applied FEA simulation to analyze the operating mechanism of a tactile sensor array under different types of loads. The same technique was used by Xu et al. [311] to simulate the behavior of different array electrode spacings and by Gao et al. [223] to design the structure of a transistor array.

Another set of simulations does not depend on the circuit configuration but on the specific application of the sensor array. A large range of approaches can be found for the state-of-the-art. Géron et al. [360] simulated both the magnetic field measurement of its sensor and the position of the robot in the plane. Kim et al. [229] simulated elastic materials. In the field of fiber-optic sensor arrays, several studies have chosen to perform simulations. Liu et al. [347] carried out numerical simulations to verify a proposed noise analytical model. Islam et al. [58] presented a large-area sensing system using transistors. Simulations were performed to test different aspects of the complete system. In this regard, Yan et al. [315] proposed a high-resolution triboelectric array. The simulations were used to better understand the operating mechanism of the proposed sensor array design. Arbel et al. [342] also performed a numerical simulation. The goal was to test two different types of fiber-optic interrogation methods and analyze the signal-to-noise ratio (SNR) in each case.

Other studies based on capacitive [36,38,143,145,147,160,165,172,173,177,181,183,185,189,194,199,205], fiber optic [33,49,334,335,338,347], piezoelectric [242,245,248,251,256,269,271,278,281,288,292,301,305,314], piezoresistive [48,84], Hall effect [363,364,365], and triboelectric [41,43,45,102,163,315,316,317,323,325,326,331] technologies evaluated their sensors by computational simulation.

#### 5.1.2. Tests Using a Mechanical Force Element

Sensor arrays are tested using a mechanical element that exerts force on the array. The type of mechanical element used depends on the specific application for which the array is used.

##### Force Gauge

**Figure 40:**
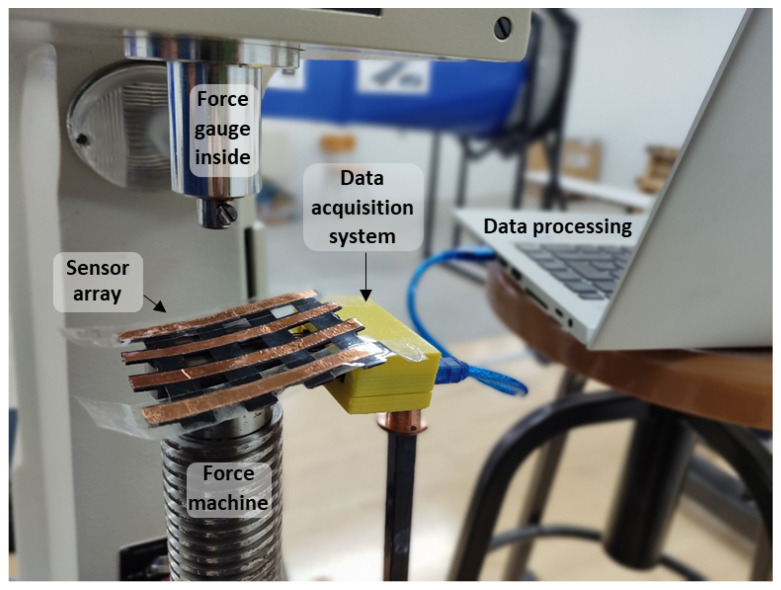
Strain machine for testing sensor arrays.

A common test method is to use a strain machine or a force gauge to test the response of the array to different force/pressure values (Figure 40). A large body of studies has used this validation method with certain differences between them [10,38,40,48,50,51,54,56,59,61,63,64,65,66,72,80,81,82,83,93,113,116,137,142,151,152,154,157,160,161,162,163,164,167,168,169,170,171,172,176,178,179,185,191,204,206,208,209,210,211,221,224,241,244,245,247,248,253,254,259,261,265,267,272,274,275,323,326,328,329,333,334,352]. In general, the testing process consists of different steps. First, a strain machine is programmed to exert forces of different values in the array. This machine usually incorporates a force gauge to measure the magnitude of the applied force. Second, an instrumentation system is implemented to record the response of the array to a given pressure value. Third, several tests are performed consisting of applying different forces with the strain machine and recording the response of the array with the instrumentation system. In this way, the sensor array is characterized. It is common to perform tests by applying pressure in both an upward and downward direction in order to evaluate the hysteresis of the array.

##### Mechanical Element Dependent on the Specific Application

**Figure 41:**
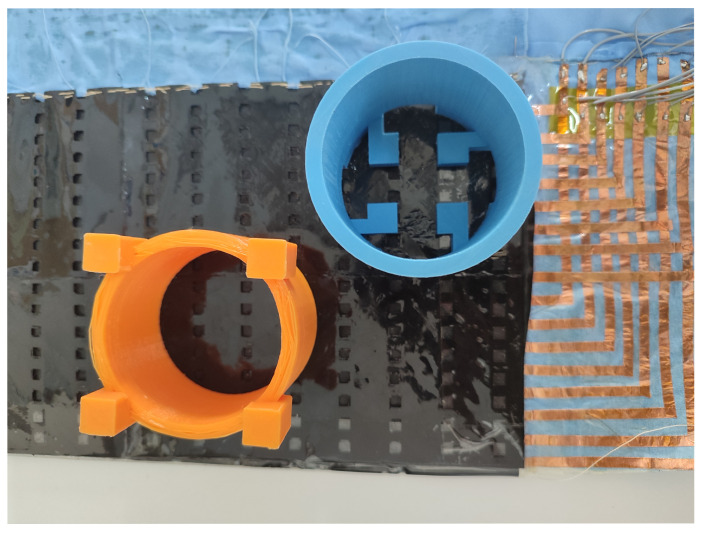
Sensor array validation using a customized object.

**Customized object or stamp:** These works use custom shapes and objects to exert pressure on the sensor array (Figure 41). This is a common technique for validating these systems [38,46,47,75,81,83,89,143,145,147,148,149,163,164,183,185,191,227,251,268,323,325].

In this regard, Huang et al. [151] pressed the array with a C-shaped stamp with different pressure values. Sotgiu et al. [154] used a stamp with parallel bars and different orientations, while Kang et al. [72] considered an L-shaped form with different weights. In this regard, Gong et al. [88] used N-shaped, straight bar, and fish-shaped objects. They also considered different pressure values. Several studies used plastic letters to press on the sensor arrays [52,85,87,93,109,163,224,245,327,328]. On the other hand, Hoang et al. [54] pressed the array with fingers, while Shin et al. [222] used a force and building blocks. Afsar et al. [67], placed a star-shaped acrylic sheet on the pressure sensor array. In turn, Tai et al. [86] tested a pressure sensor array for neck flexion monitoring. They used a cervical spine model loaded with a 5 kg dumbbell. Finally, a rubber 3D stomach model was used by Ye & Ju [38] to test a pressure sensor array. They also tested it on a real pig liver.

**Figure 42:**
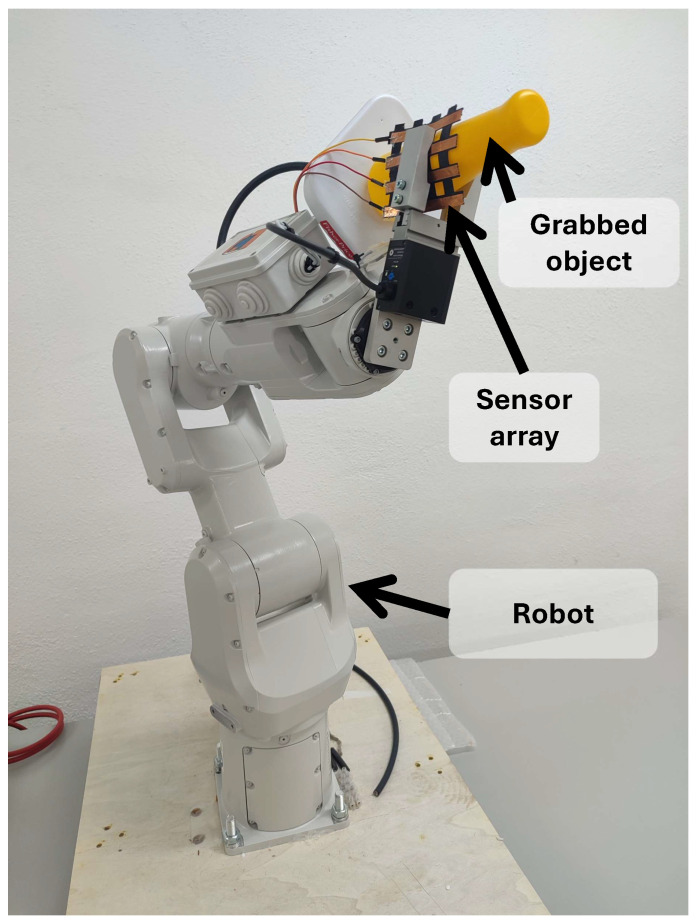
Sensor array validation using a robot.

**Robot:** Robots have been used to validate sensor arrays (see Figure 42). These studies focused on e-skin sensor arrays. A set of studies did not use robots as a means of validation but as a central part of the application.

Testing tactile sensor arrays by attaching them to a robotic hand is a common method [35,38,85,146,164,184,190,251,328]. In this sense, Liu et al. [275] designed a piezoelectric tactile fingerprint sensor array that focused on the recognition of roughness for use in a robotic hand. Lin et al. [246], mounted their piezoelectric array on a robotic hand so that it interrupted the grasping process when the sensor output exceeded 4V. Similarly, Fang et al. [10] proposed a capacitive robotic fingerprint to capture object properties in manipulations. Validation consisted of grasping with the fingers of a robotic hand (with the sensor array on them) a foam board placed in the palm of that robotic hand. Kim et al. [260] attached the sensor to a finger of the robot hand that grasps various types of objects. The test consisted of recognizing the contact area. In the work of Su et al. [76], an array of piezoresistive transistors was attached to the hand of a robot. This makes the robot aware of the mass of the grasped objects. Qin et al. [316] presented a magnetic array-assisted sliding triboelectric sensor for real-time gesture interaction between a human hand and a robotic hand. Both a robotic hand and a human hand were involved in the tests. In the work of Fischer et al. [361], a parallel kinematics robot was used to move the target to be detected by a Hall effect sensor array. Several works presented a Hall effect sensor array for locating magnetic robots [359,360].

**Figure 43:**
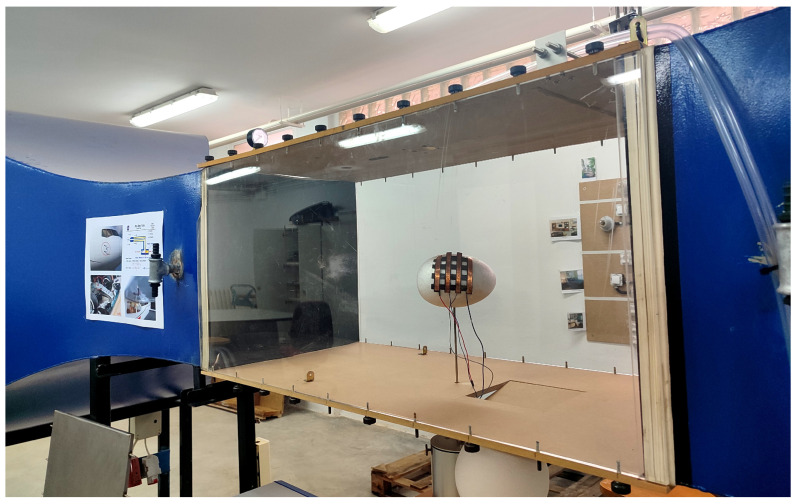
Sensor array validation using a wind machine.

**Wind machine:** There are a number of studies focused on airflow sensor arrays. To test the validity of these systems, a common approach is to use a wind tunnel, insert the sensing system inside, and see its response under different wind speed values (Figure 43).

Fonseca et al. [278] performed a study on elastic vibration control of a satellite containing flexible solar arrays. They placed the sensors on a plate and performed the test inside a wind tunnel. In this sense, Bian et al. [6] presented a biomimetic 3D airflow sensor array. To test the prototype, the array was attached to a rotatory worktable that was placed inside a miniature wind tunnel with adjustable wind speed. On the other hand, Cong & Jing [289] presented a work in the field of compressors. A pressure sensor array was designed to measure the pressure field distribution over the compressor rotor blade tip clearance. The test involved a flow compressor with the sensor array attached to the housing wall of a tip of the rotor blade. In addition, Chen et al. [91] designed a piezoresistive array of airflow sensors for airflow velocity and direction sensing. To test the sensor, it was placed inside a wind tunnel. Similarly, Ko et al. [317] presented a triboelectric array for the omnidirectional measurement of wind speed and direction. A small wind tunnel was used to test the sensor array. A high-speed camera recorded the oscillatory motion of the flap array at different wind speeds. A similar approach was taken by Zhang et al. [43] and Zhao et al. [37] Finally, Wang et al. [102] studied how much power a TENG array could deliver to a chemiresistive array, using a fan for testing.

**Figure 44:**
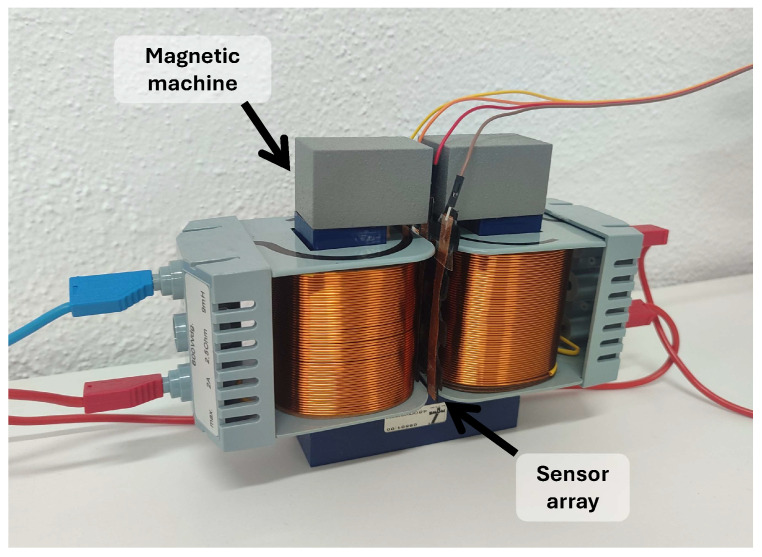
Sensor array validation using a magnetic machine.

**Magnetic Machine.** Several papers on sensor arrays used a magnetic machine for validation (see Figure 44). In this regard, Qu et al. [309] developed a customized magnetic machine that generated a magnetic field controlled through an AC current source. In addition, Duan et al. [308] used a vector network analyzer that produced frequency continuous wave signals to test a piezoelectric array. Similarly, Kim et al. [5] used a network analyzer to measure the admittance of a sensor under a magnetic field generated by a Helmholtz coil. In this sense, Näf et al. [103] used a two-coil Helmholtz setup, exposing a magnetoresistive array to a controlled magnetic field. Finally, Vizel et al. [365] used a coil consisting of several segments. The coil had 2783 turns and carried a sinusoidal current of 10 Hz and 50 mA.

**Acoustic machine:** Several studies used acoustic machines for sensor array validation. On the one hand, Kim et al. [267] presented a piezoelectric sensor array to measure elasticity and force based on acoustic load impedance sensing. To test the performance of the array, a stress-strain measurement and a force-sensing test were conducted. On the other hand, Yamashita et al. [261] mounted an array of piezoelectric strain sensors in the center of a stainless steel plate. The edges were fixed to tables and the strain was generated by applying a load in the center of the plate. In addition, Wang et al. [339] detected speech signals with a fiber-optic array installed in an anechoic room. In this sense, other work in the field of fiber-optic sensor arrays also used acoustic machines [1,340].

**Loudspeakers:** Several works used loudspeakers to test the sensor arrays. Nagai et al. [295] presented a piezoelectric array to measure Young’s modulus and viscosity. The tests were performed with loudspeakers, studying the response of the array to sound. Holeczek et al. [293] investigated sound wave generation using piezoelectric sensor–actuator arrays. System tests included measurements with microphones and a scanning laser Doppler vibrometer. Several studies on piezoelectric arrays used this type of validation, as this technology is suitable for sound detection [271,293,295,305].

**Figure 45:**
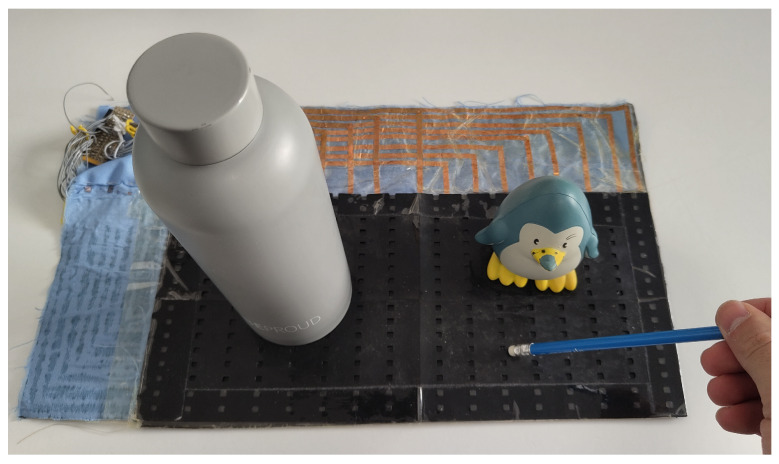
Sensor array validation using daily life objects.

**Daily life objects:** Another group of studies directly used everyday objects to test the performance of the sensor array (see Figure 45). Most of the studies that used this validation method were of pressure sensors. The tests consisted of evaluating the ability of the arrays to identify a specific object or accurately obtain its weight distribution. Objects were often placed in various orientations to evaluate the performance of the arrays when different sensors on the mats were activated. Among the objects used in the experiments, a large proportion can be found to be state-of-the-art.

In the healthcare field, Nagayama et al. [282] and Tian et al. [283] used a 3D heart model. The sensor array was attached to the heart model to identify its movements and enable real-time detection of irregular movements or heart disease.

On the one hand, Li et al. [258] designed a helmet impact resistance test system using a sensor array. They used a headform with the sensor array attached to the surface. The headform was inserted into the helmet and impacts of different magnitudes were applied. The sensor array measured the magnitude and severity of the impact.

On the other hand, Yamashita & Kobayashi [262,263] developed a smart ping-pong racket. The piezoelectric sensor array was attached to the racket for testing. The objective of the tests was to identify the location of ball impact and the type of shot. In this regard, the detection of strikes during tennis practice was performed by Yang et al. [68] using an IMU and a flexible pressure-sensitive sensor mounted on the racket.

Other authors used objects such as a pencil, a coil, a bottle, or toys to evaluate the performance of sensor arrays. In this sense, Sim et al. [264] used a pencil to press the sensor array attached to a human hand. The purpose of the test was to reproduce an electrical ’pain’ signal on the sensor array. Chong et al. [60] used a coin to exert forces on the array in different directions. The objective of the test was to see the dynamic pressure response. Similarly, Jeon et al. [55] obtained various pressure maps of everyday objects such as a bottle of water or a Lego figure, while Kinjo et al. [116] obtained the surface pattern of different soft toy balls. Touching the arrays with everyday objects is a common technique for preliminary validation of different types of sensor arrays [143,145,185,245].

**Rotatory table:** Rotatory tables were used to validate sensor arrays in different orientations. For example, Pu et al. [179] used a rotatory table to test an array of angular measurement sensors. Bian et al. [6] presented a fluid mechanics application. They used a mini-rotatory table to orient the sensor array into the wind. Finally, Chang et al. [327] attached prostheses with triboelectric sensor arrays to a rotatory motor, which was used to simulate gait movement.

**Figure 46:**
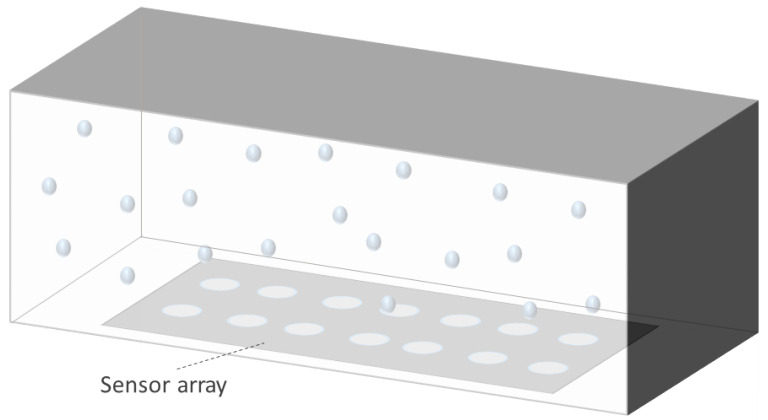
Representation of sensor array validation in a wet environment.

**Control humidity chamber:** A humidity chamber is a device that allows different relative humidity values to be set in a given environment (inside the chamber). If a sensor array is placed inside the humidity chamber, measurements can be obtained at different relative humidity values (Figure 46).

Two approaches can be found to be state-of-the-art: on the one hand, the humidity chamber is used only to test the performance of the array under different relative humidity conditions, while on the other hand, the humidity chamber is used to test the performance of the sensor itself, as the array is intended to measure humidity. The work of Pyo et al. [172] falls under the first approach, while the work of Sun et al. [310] and Demori et al. [96] falls under the second.

In this regard, Pyo et al. [172] tested an array of capacitive tactile sensors under various relative humidity conditions, using a customized humidity control chamber. However, Sun et al. [310] designed a humidity sensor based on a piezoelectric ultrasonic array. A humidity chamber was used to obtain the sensor sensitivity and hysteresis characteristics under different humidity levels. Similarly, Demori et al. [96] used a food box to monitor different levels of temperature and humidity. In this case, they tested a mixed array of resistive and capacitive sensors.

**Figure 47:**
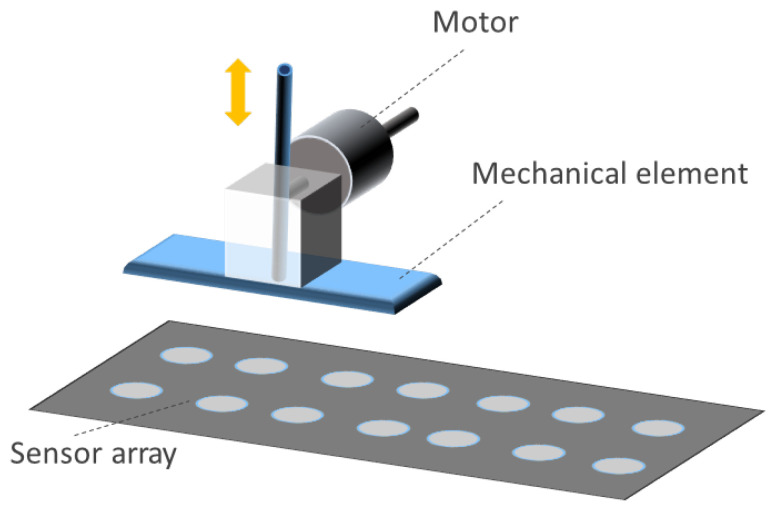
Representation of sensor array validation using linear motors.

**Motor.** The most common application of linear motors is to test the dynamic response of a sensor array [30,31,35,38,39,40,42,43,44,46,47,52,85,86,87,144,146,147,149,156,183,186,218,224,243,244,248,251,259,285,319,327,328,329,358]. Figure 47 represents this validation approach. In the work of Qin et al. [316], the motor was configured to rotate at different angles, representing finger flexion/extension. Similarly, the TENG array proposed by Gao et al. [320] used a programmable linear motor to control a moving end impacting the array installed on a fixed part. In this regard, Yang et al. [318] tested another triboelectric array using a linear motor applying a controlled force to characterize the electrical response of the sensor. Linear motors can be used to apply controlled pressure to sensors and measure their hysteresis [175].

Meanwhile, in the work of Yeo et al. [241], a linear motor was used to periodically bend and release a tactile sensor. The objective was to test its mechanical durability. The results showed that no cracks or delamination occurred in the array after 1000 bending/unbending cycles. The linear motor was also used to test the crosstalk effects of the array by applying a localized constant pressure.

In a different field, Hu et al. [249] presented an array to monitor the health of ion-lithium batteries. They used a linear motor to check for mechanical damage to ion-lithium batteries that were monitored by a sensor array. The puncture damage of the batteries was mimicked by using a linear motor with a sharp needle. The array was able to correctly identify the degree of battery damage.

Several studies used motor-based stages for validation. Dusek et al. [3] used a linear stage based on a stepper motor to plunge a piezoresistive sensor array inside a tank. This sensor was designed to act in marine applications. Similarly, Naku et al. [356] tested a fiber-optic array by immersing it in a target VOL using this type of actuator. In this sense, Yue & Moussa [73] validated a piezoelectric force sensor array using a three-axis linear stage.

Finally, in underwater/marine applications, motors were used to generate waves inside water tanks for testing purposes [1,41,242,256,342].In turn, Baker et al. [346] used a three-axis motion system to control the position of a needle inside a water tank.

**Figure 48:**
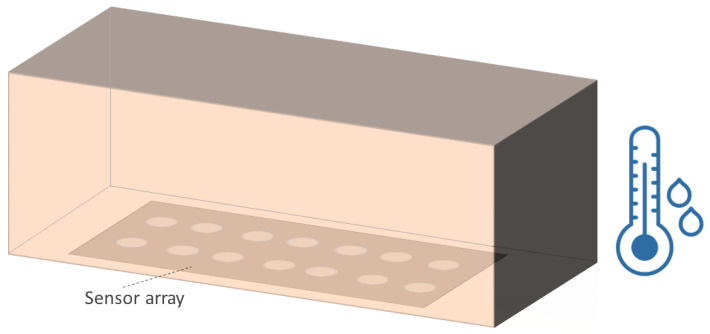
Representation of temperature influence on sensor array validation.

**Temperature machine:** Several works have tested the influence of temperature on sensor arrays using specific machines (Figure 48). In this sense, Zhang et al. [69] used a dedicated temperature machine to calibrate a pressure sensor array for different temperature values. Fan et al. [97] tested a readout algorithm for a resistive sensor array. The algorithm compensated for measurement errors in high temperature environments (500 °C). Finally, Veske et al. [173] placed a heater on an integrated circuit to test its resistance to thermal stress. In [297], the effects of temperature variations on Lamb wave propagations are studied. For this purpose, an epoxy plate is placed inside a thermostat, where these waves are obtained with a piezoelectric array formed by PZT sensors. Finally, Zhang et al. [52] tested a pressure–temperature e-skin using a heater.

**Figure 49:**
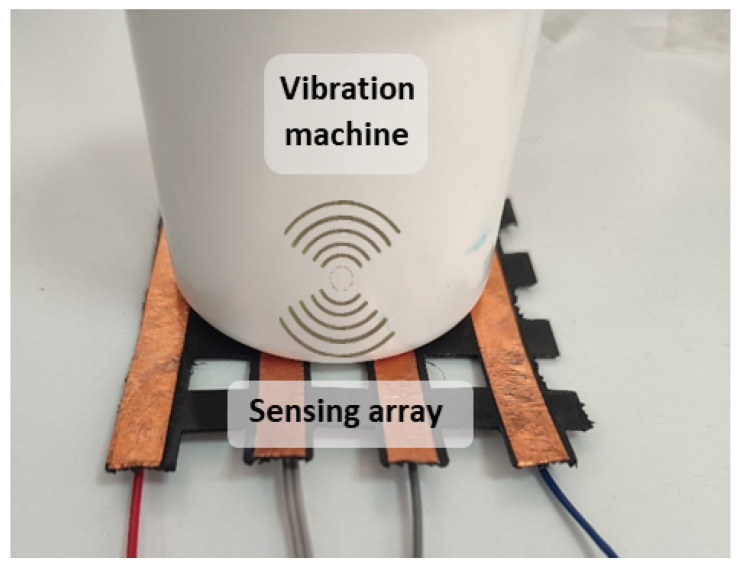
Sensor array validation using a vibration machine.

**Vibration machine:** Vibration machines have been used to test sensor arrays. Experiments consists of impacting the array with the vibration machine (or a suitable piece attached to it) to characterize the sensors. Various parameters can be set, such as vibration frequency, vibration force, or amplitude of movement (Figure 49). In this way, the performance of the array can be tested under different conditions.

In this regard, Chen et al. [266] presented a triaxial piezoelectric tactile sensor for three-axial force measurement. Tests were performed to obtain sensitivities for the X-, Y-, and Z-axis force components. For the vertical force test, an electromechanical vibrator was used to apply a compressive force on the sensor. The sensor was placed under a cylindrical probe driven by the electromechanical vibrator. Similarly, Kim et al. [71] used an oscillator placed on a tactile pressure sensor array to obtain the sensor response to vibrational stimulation. The frequencies analyzed ranged from 1 to 400 Hz. The time delay between sensor response and vibration amplitude was also obtained. An electrodynamic shaker was used by Kim et al. [330] to test a sensor array under different input forces and frequencies. Other studies with vibration machines tested fiber-optic sensor arrays [33,49,332,348,349], or piezoelectric sensor arrays [245].

**Light machine:** Light machines are ideal for light-based imaging sensor arrays [15,218,227,228,229].

#### 5.1.3. Chemical Testing

**Figure 50:**
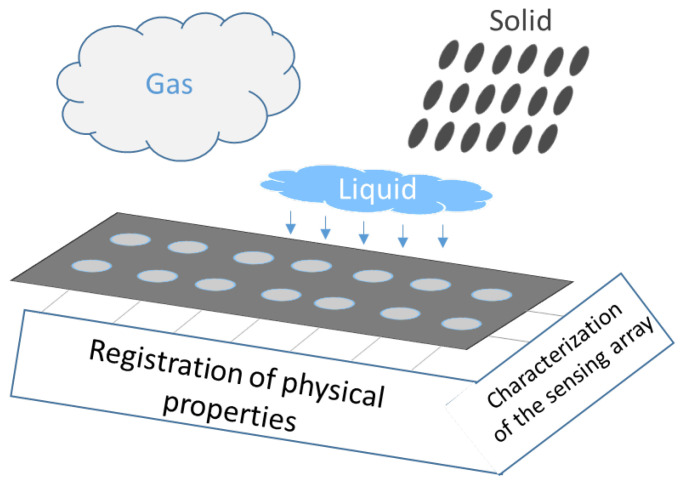
Representation of chemical testing for sensor array validation.

Several studies presented sensor arrays that were validated by exposing them to specific elements or compounds [2,7,18,19,34,99,100,101,193,197,198,199,201,203,205,206,217,220,225,226,232,233,235,236,237,238,240,306,307,308,355,356]. Most of these types of arrays belong to the category of chemical sensors. The studies were carried out in a controlled laboratory environment and focused on characterizing the physical response of the array when the sensing material was exposed to a certain element (Figure 50). Gaseous and liquid compounds were the most frequent exposure elements. The tests were usually repeated several times under different conditions (temperature, humidity, irradiance, etc.). Sensor hysteresis was often characterized. In these types of studies, experiments focused entirely on the physical properties of the sensor array and not so much on the application of the system.

#### 5.1.4. Validation with Human Subjects

A number of studies used humans to validate sensor arrays. Those studies focused on applications of the arrays that involve real people. Validation experiments can be classified according to whether real users wear the systems or interact with them externally.

Studies involving human subjects must be approved in advance by a competent ethics committee. In addition, written informed consent must be obtained from all participants prior to testing.

##### Subjects Wearing the Device

**Figure 51:**
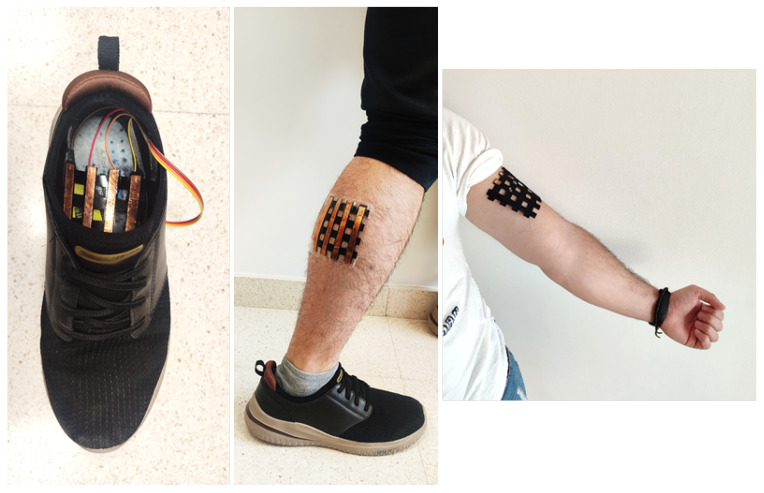
Representation of the validation technique with human subjects wearing the device. Shoe/foot (**left**), leg (**center**) and arm (**right**).

Studies in this category can be grouped according to the part of the body where the device is worn:

**Sensor arrays worn on the foot** (Figure 51): Within this category, Aqueveque et al. [153] integrated a capacitive sensor array inside a footwear to detect the start and end of the swing phase of gait. Thirteen subjects (eight men and five women) participated in the tests. Each subject walked with the sensorized footwear for 10 m, repeating the experiment twice (the first time at a normal speed and the second time at a faster speed). For comparison, the subjects wore an inertial sensor located on the lower back. The sensor array showed a lower detection error. Similarly, Muzaffar et al. [74] presented a shoe-integrated sensor array to measure body weight and gait. One type of test performed consisted of wearing the instrumented shoes while walking and standing. Other wearable arrays for gait assessment also applied this validation approach [39,45,87,255,259].

**Sensor arrays worn on the leg** (Figure 51): Kekonen et al. [367] tested a bioimpedance sensor array for long-term monitoring of wound healing in the leg. The test consisted of monitoring intact skin and different wounds. Two experiments were conducted, placing the sensor array on both shins. A total of 142 h were monitored in each experiment, finding differences in bioimpedance measured on intact skin and wounds. In this regard, Zhang et al. [150] attached the sensor array to different leg muscles, such as the gastrocnemius or quadriceps, to monitor muscle contraction/relaxation.

**Sensor arrays worn on the arm** (Figure 51): Zhang et al. [150] also attached the capacitive sensor array to the biceps to assist people and receive feedback and guidance during exercise. In the tests, the array was attached to different body positions. In this sense, the Hall effect sensor array proposed by Fischer et al. [361] was placed on a subject’s arm to study whether bending the array affected its performance. Chen et al. [248] tested a tactile piezoelectric sensor by pressing it while attached to the inner forearm.

**Figure 52:**
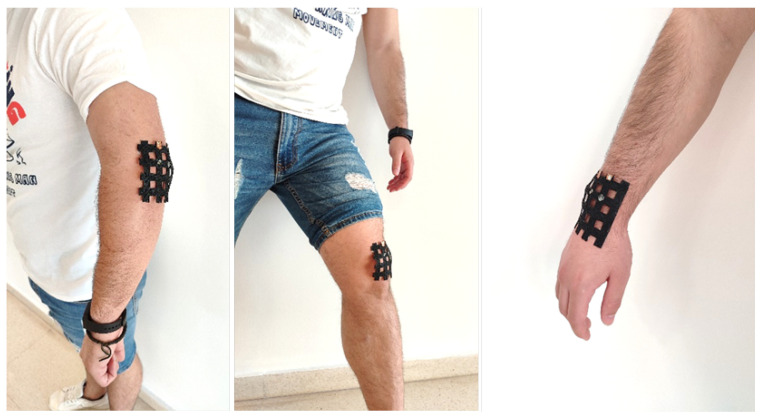
Representation of the validation technique with human subjects wearing the device. Elbow (**left**), knee (**center**) and wrist (**right**).

**Sensor array worn on the elbow or knee** (Figure 52): Several studies placed the sensor array on joints of the human body. In this sense, Li et al. [204] tracked knee and elbow motion. This type of motion was also measured by Cheng et al. [56], and Lei et al. [87]. Abbasnia et al., [214], Lei et al. [87]. Zhen et al. [245], also tracked elbow motion. In [303], a piezoelectric sensor array is attached to a porcine leg to monitor knee osteoarthritis.

**Sensor arrays worn on the wrist** (Figure 52): All studies in the bioimpedance category, due to the specificity of these types of sensor, have used humans wearing the arrays for testing. In this sense, Ibrahim and Jafari [366] involved 4 subjects in the tests of a blood pressure monitoring wristband based on a bioimpedance sensor array. A total of 6000 heartbeats were collected from each participant who wore the wristband.

On the other hand, Niu et al. [158] obtained the pulse before and after exercise using a sensor placed on the wrist. Other studies also measured the pulse on the wrist [37,46,47,57,87,92,150,257,329].

Furthermore, Booth et al. [276,277] involved ten subjects in different tests with a wristband sensor array, while Matsuda et al. [89] and Xu et al. [162] tested wrist flexion. A similar approach was taken by Zhen et al. [245,271].

Meanwhile, the sensing system designed by Lee et al. [62] fixed a visualization diode array to the wrist. Different sensing units were attached to the skin to detect body temperature, pressure, and UV intensity. In this regard, Tang et al. [208] placed a smart keyboard based on an inductive array on the wrist. The experiments consisted of the use of the smart keyboard by a volunteer. Similarly, Yeom et al. [137] developed another wrist-mountable keyboard composed of thin-film diodes.

**Figure 53:**
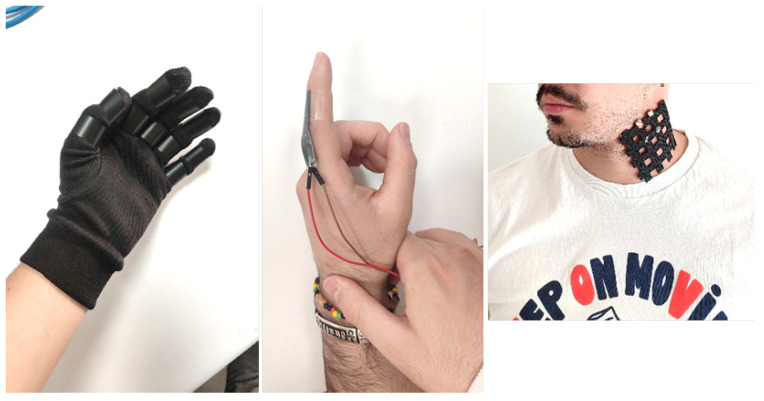
Representation of the validation technique with human subjects wearing the device. Hand (**left** and **center**) and neck (**right**).

**Sensor arrays worn on the hand** (Figure 53): Several studies have placed sensor arrays in the hands of volunteers for validation. For example, the test performed by Luo et al. [142] involved placing the sensors in a glove and examining the change in capacitance when grasping objects. Different hand postures were tested. The tests were carried out on an array with and without damage. Several studies also monitored finger bending with a smart capacitive glove [158,159,160,174]. Similar experiments were carried out with a triboelectric array [40,316,322,329] and piezoresistive arrays [47,57,87]. Similarly, Jang et al. [322] examined variations in the charge density of a triboelectric sensor when touching different shapes. Sim et al. [264] performed a test consisting of attaching the array to a finger and pressing with different sharp or hot objects, trying to reproduce the “pain” of humans. In this regard, Esposito et al. [53] conducted a test with ten subjects to detect eight hand movements with an armband form by a piezoresistive array. Meanwhile, Wu et al. [114] proposed a Velcro structure that was embeddable inside a glove. Various types of sensors were placed in the glove: thermistors, photoresistors, etc.

**Sensor arrays near the neck** (Figure 53): Several studies have adopted this approach for validation. Niu et al. [158] attached the sensor array to the throat to monitor swallowing, water intake, and movement and vibration of the throat muscle. Subjects who performed swallowing action with the neck sensor were also found in the work of Li et al. [57]. A similar approach was adopted by Parashar et al. [42], and Lei et al. [87]. In addition, Jeong et al. [250] placed a piezoelectric array in Adam’s apple for speech and swallowing detection. A similar experiment was performed by Xu et al. [162], and Su et al. [47]. In this sense, Lin et al. [246] focused on distinguishing various stimuli in real time. The tests consisted of wearing the array on parts of the body, such as the neck. Zhao et al. [37] placed a capacitive array on the carotid artery.

**Sensor arrays near the eye**: Niu et al. [158] placed a capacitive sensor array near the eye to detect blinking in the eyes, while Sun et al. [329] tested a triboelectric array. In turn, Kim et al. [243] placed a piezoelectric array on the side of the eye to test eye tracking.

**Sensor arrays inside the mouth**: In the work of Tai et al. [70], a piezoresistive pressure array was placed on a molar tooth and its pressure distribution was measured. In this way, the function of the tooth after surgery could be analyzed.

**Sensor arrays worn on the back**: Zhang et al. [78] asked subjects to wear a 4-by-4 resistive sensor array on the lumbar region to track spinal postures. Cen et al. [80] developed an smart t-shirt which included all the necessary hardware for elbow and shoulder angle monitoring. It had several sensors distributed on the back.

**Sensor arrays worn on the abdomen**: In some works [143,145], the test consisted of placing the sensor on the abdomen of the subjects to detect respiratory patterns.

##### Subjects Interacting Externally with the Device

In this category, sensor arrays are not worn by the subjects. In contrast, the validation method is based on external interaction with the sensor arrays. Several studies have adopted this approach.

**Figure 54:**
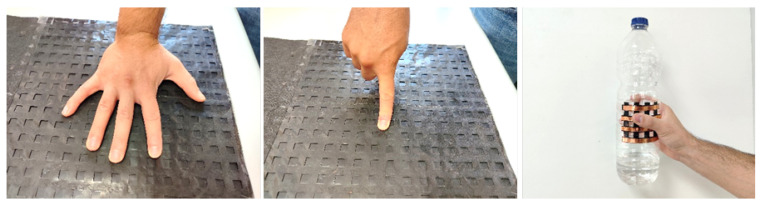
Representation of the validation technique with human subjects interacting externally with the device. Mat pressed by a hand (**left** and **center**) and mat integrated in bottle (**right**).

On the one hand, Liu et al. [148] presented a capacitive sensor array for gesture recognition. The tests involved six subjects performing four hand gestures on a table, repeating 50 times each (see Figure 54). In this sense, Ye et al. [181] designed a capacitive proximity sensor array. The tests consisted of moving the hand at different distances from the array. Several sizes and speeds of the hands were tested. Similarly, Khatoon et al. [213] performed several gestures on an inductive array to train an ML algorithm. In addition, Ma et al. [188] presented a capacitive imaging array and performed non-contact obstacle positioning and tracking. They also performed pressure mapping by pressing their finger on different points of a deformable foam placed on a capacitive plate (see Figure 54). Zhang et al. [44] trained a ML-based handwriting recognition system with real-world handwriting. In fact, having subjects press the sensor array is the most common validation method in studies that test their prototypes by interacting externally with them [30,47,52,54,57,68,70,79,82,87,89,92,94,143,144,145,146,147,149,155,174,182,185,186,212,214,224,245,246,250,260,261,270,271,285,305,314,315,319,322,325,327,328,330,331].

On the other hand, Niu et al. [158] performed tests consisting of grasping a glass, clicking a mouse button, and pressing a flexible object. Similar tests were also conducted by Li et al. [204]. In [326], a triboelectric array embedded in a keyboard was subjected to continuous typing. It was able to power a wristwatch and a calculator. In other studies, participants also stretched [8] and bent [160] the sensor array during the testing. In this regard, Xue et al. [94] fixed the array on the surface of a beaker and held it by hand. In contrast, Tamiziniyan & Febina [287] embedded the array in a quilt. The tests consisted of monitoring five different sleeping positions. Ten subjects participated in the experiments. Husak et al. [77], and Hussain [81] also tested the sensors with subjects lying on the array.

**Figure 55:**
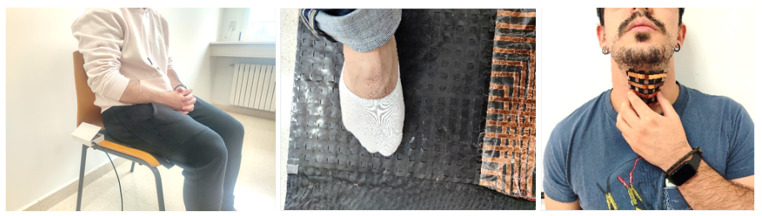
Representation of the validation technique with human subjects interacting externally with the device. Mat embedded in a chair (**left**), plantar pressure assessment (**center**) and mat externally attached to the neck (**right**).

Another series of studies was more health-oriented. In this sense, Ghamsari et al. [104] performed plantar pressure scanning. A user stepped on the mat for testing (see Figure 55). A similar test was also carried out by Wang et al. [324], and Ghouchani et al. [108]. In contrast, Xu et al. [284] involved 33 subjects in an experiment to validate a piezoelectric sensor array for the measurement of carotid-femoral PWV. The experiments were repeated three times for each subject. A gold-standard device was used for comparison. Zalar et al. [244] monitored 20 volunteers using a large-area piezoelectric cardiographic sensor array. In addition, in [92,156,202], mouth-blown air detection was performed and tested. Similarly, Iizuka et al. [286] performed tests consisting of detection of laryngeal movement during swallowing. The array was attached to the ventral surface of the neck of the subjects (see Figure 55), near the laryngeal prominence, and held by the hand of a researcher. Twelve subjects participated in the tests repeating the protocol between 10 and 20 times.

Meanwhile, Kuchmenko et al. [307] presented a chemical array to diagnose a specific human infection. Three groups participated in the tests: 10 patients with previous positive and negative diagnoses, 38 patients suffering from respiratory and digestive disease, and 27 patients who had undergone previous therapy. The saliva of the patients was used in the arrays for diagnosis. In this regard, Tai et al. [70] presented an application for dentists. The tests consisted of monitoring the pressure of a molar before and after a dental filling process. The array was placed between the upper and lower molars of a model.

#### 5.1.5. Lab Experiment

Several studies performed a variety of customized laboratory experiments to validate sensor arrays. There is no uniformity or pattern to categorize this set of studies, as they depend entirely on the type of sensor or application.

In the group of capacitive sensor arrays, Tholin & Soleimani [187] presented a capacitive sensing system in which the dielectric of the capacitor was the object to be detected. The experiments consisted of detecting a bottle buried in sand or a block of wood suspended in air. Thus, they performed ECT. In this sense, Elzaidi et al. [207] applied water to the sensor surface with a paint roller. As in the previous study, the material to be detected was the dielectric of the capacitor. They performed water or ice detection. Similarly, Cao et al. [189] used sand and air to simulate various two-phase flow distributions inside a metallic pipe. Zhu et al. [194] designed a six-electrode capacitive array wrapped around an acrylic pipe. The test consisted of measuring the particle concentration in the two-phase flow inside the pipe. Sun & Sun [36] applied CACT to find defects in several composite films. The defects were hand-made square holes in the material. Meanwhile, Wang et al. [180] presented an object proximity detector. The tests consisted of varying the capacitance in a program and observing the reaction of the sensor system to proximity. Wang et al. [9] performed transient voltage measurements on real transformer windings, using a pulse generator to mimic real lightning. Shu et al. [281] tested the energy harvesting capability of a piezoelectric array with a customized prototype.

In the field of fiber-optic sensor arrays, several authors have set up laboratory experiments specific to this technology. In this regard, Liu et al. [352] conducted a laboratory experiment of a FBG sensor array applied to the aerospace industry. They designed a custom test bench. Similarly, Gutierrez et al. [335] monitored the acceleration, rotation, and velocity of aerospace structures and launch vehicles using an FBG-IMU. A custom lab setup was implemented to test the system. Liu et al. [347] designed an experiment to measure the level of input noise. Ren et al. [350] installed their antivandal fiber-optic sensor array in a real outdoor pipe. The pipe was then tapped with a hammer and an electric drill. Finally, Liu et al. [338] also implemented a custom setup to test a new approach that improved the dynamic range of a fiber-optic sensor array. In [349], a custom experimental setup was built combining optical fiber launch modules and receiver/recombination modules. They were controlled by an FPGA. Liu et al. [32] tested a multichannel detection strategy for fiber-optic arrays under similar conditions. They also applied a new approach to monitor cerebral and intestinal hemodynamics in in-vivo experiments.In turn, Zhao et al. [336] assessed a Sagnac hydrophone array by sending certain light signals into the beams and recording their responses.

Another group of studies also presented validation experiments that were heavily dependent on the specific sensing technology of the arrays. Several validation experiments were performed for transistor-based sensor arrays [95,227,228,229,230,234,239,268]. The tests consisted of custom lab experiments that focused on characterizing the physical properties of the devices. Similarly, for triboelectric technology, Chen et al. [321] and Jang et al. [315] conducted laboratory experiments to evaluate the voltage response of a triboelectric array to different input stimuli.

Several studies focused on validation experiments to improve the circuit-related aspects of sensor arrays [78,79,104,109,111,112,118,120,124,125,126,127,129,130,131,132,133,138,140,166,192,196]. In general, most studies developed physical lab prototypes to test a specific improvement in the electronics of sensor arrays in real life. Some of them first simulated the circuit improvements and then validated the physical prototype with a customized laboratory experiment involving various sensing scenarios.

Other studies used various application-dependent laboratory experiments. In the field of radiation detection, Park et al. [353] carried out experiments with radioactive materials. The sensor array was moved along a vertical guide to obtain a map of the intensity of radioactive emission in an area. For the experiment, they used a gamma-ray source. In this sense, Jia et al. [219] presented an imaging sensor for radiation detection.

Piezoelectric sensor arrays for structural damage detection were frequently tested in laboratory environments. In this regard, Khan et al. [292] validated an acoustic sensor array for damage detection in solids with an ultrasonic transmitter. The detection of damage to materials was usually tested in laboratory experiments [4,256,273,274,280,291,294,298,299,300,301,302,304]. In the field of marine applications, Wei et al. [242] developed a piezoelectric pressure array to study the pressure field around an airfoil. The airfoil was immersed in a water tank. In this way, laminar, transition, and turbulent flows were tested.

In the field of health, Tian et al. [283] used a model of a heart for testing. Feng & Su [288,312] developed a piezoelectric airflow sensor array. It was placed on a mobile platform with several syringes attached. The movement of the platform mimicked the human breathing rate, so the syringes exhaled and inhaled air at that rate. Feng & Chen [303] studied knee conditions with a piezoelectric array, which was attached to a porcine leg. Meanwhile, a mobile stage mimicked an exercise routine.

Other validation experiments focused on specific objects. In this sense, Susac et al. [357] played chess on a sensor array based on the Hall effect. Liu et al. [216] placed metallic objects under an inductive array at a certain depth to detect UXOs. Lin et al. [246] placed a spider on the sensor array in a laboratory. Faria et al. [215] mounted an inductive array on a mechanized scanner to analyze some defects in a 3D-printed metal part. The output voltage was simultaneously plotted on a regular PC, providing a visual verification of system performance.

A set of studies implemented laboratory experiments for applications in the field of power systems [362]. In this sense, Luca et al. [362] designed a lab test to monitor battery strings. A similar experiment was conducted by Tang et al. [363]. Meanwhile, Zhang et al. [344], and Liu et al. [344] reproduced in a laboratory the partial discharges that occurred in oil-immersed transformers due to poor isolation. Finally, Tuukkanen et al. [252] used a mechanical tester to measure the stretching force exerted on a piezoelectric array as well as a charge amplifier to measure the generated piezoelectric charge.

### 5.2. Brief Conclusion of Sensor Array Validation

**Figure 56:**
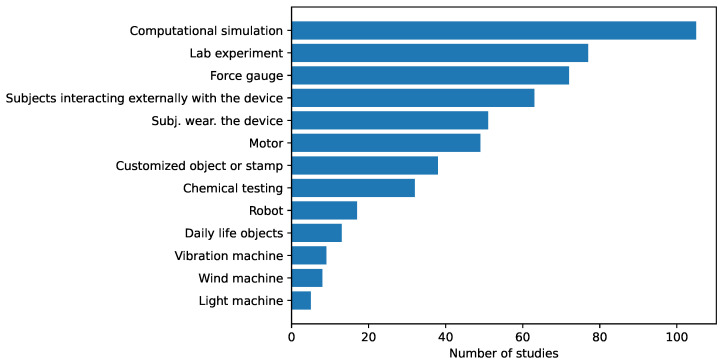
Number of sensor array validation studies classified by validation method. Only the most frequent types of validation experiments are represented.

Figure 56 shows the number of studies that applied the different validation methods. Computational simulation is the most frequent validation approach. When designing a new sensor array or improving existing state-of-the-art technology, the performance of previous simulated tests is very useful. Computational simulation was adopted by 29.1% of the existing studies. In addition, laboratory experiments (lab experiments) have also been frequently used as a validation technique. This includes a wide range of laboratory settings. This validation method was adopted by 21.3% of the studies. In relation to test instruments, force gauges are widely used, appearing in 19.9% of the studies. This may be because pressure sensor arrays are the most common technology. In contrast, validation experiments focused on specific applications or technologies and using elements such as magnetic, temperature, or vibration machines were found in isolated cases.

Finally, Table 5 shows a summary of the advantages and disadvantages of each validation experiment to be considered by researchers in this field.

## 6. Software for Analysis

This section includes a review of the software tools used in the experiments to evaluate the performance of the sensor arrays.

### 6.1. Results of the Analysis

Table 6 presents the results of the analysis of the software tools for the most recent studies. The rest of the studies are analyzed in Table A3.

**Figure 57:**
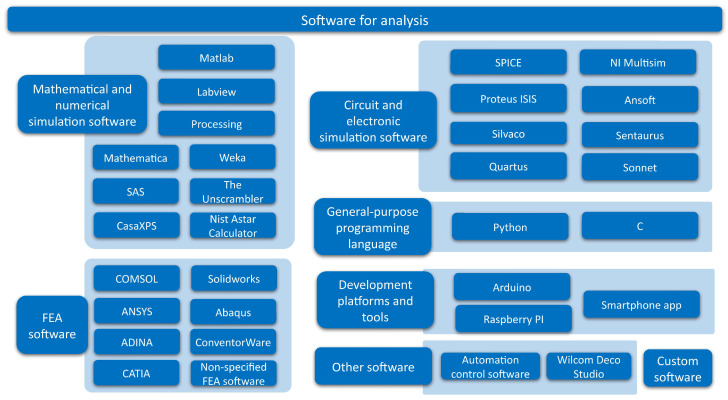
Results of the software tools used in sensor array studies.

Figure 57 categorizes the different items found in the state-of-the-art of sensor arrays in relation to the software tools used for analysis. They are briefly described in the following subsections. All studies in Table 6 and Table A3 are explicitly cited in the corresponding subsection to which they belong. Studies that use more than one software tool are cited in all associated subsections.

#### 6.1.1. Mathematical or Numerical Simulation Software

##### MatLab

MatLab is a popular mathematical software that is applicable to a wide variety of fields. It is the most widely used software in state-of-the-art studies. MatLab has been applied in sensor array studies for different tasks:

- Performing complex matrix operations [105,106,136].
- Simulation of resistive arrays with their corresponding readout circuits [78,111,118,120,122,127,132,133,134].
- Digital filter design [3,30,205,207].
- Modeling of systems with classical techniques such as state-space models [273,335]. The Matlab Simulinkpackage was also used for system design and modeling [132,133,134].
- Fitting data to mathematical models [98,363].
- Data processing of results [252].
- Serialization and data storage on a general PC [168,178,204,233,284].
- Training of AI models [69,148,214].
- FEA for the study of deformations in pieces [352] or vibration tests on piezoelectric sensors [278]. MatLab can be connected to COMSOL to this end [243,363].
- Solving complex equations [31,296].
- Calculation of characteristic parameters of sensors such as the piezoelectric resonant frequency [99,308] or the spindle outline in fiber-optic arrays [334].
- Graphical tools to represent results [30,43,54,164,168,178] or visual interfaces for HMI systems [30,208].
- IoT development [325].
- Simulate the response of a sensor structure under basic inputs, such as unit step [248].

##### LabView

LabView is a visual environment for system design developed by National Instruments. It has been used in a large number of sensor array studies for different tasks:

- Signal processing [55,196,291,339,359,360] and data display [215,222,241,265,268,275,359].
- Control of readout electronics or data acquisition [3,7,42,43,95,152,170,183,196,197,215,243,266,288,316,329,363] and actuator operation [64,157,161,360].
- Algorithm implementation [196,316,359].
- Transduction of voltage signals to other physical variables such as current [362] or displacement [288,312].
- HMI visualization [315,321].
- IoT applications [318].

##### Processing

Processing is, according to its webpage, “a flexible software sketchbook and a language for learning how to code” [371]. Esposito et al. [53] used Processing for real-time human–computer interaction and calibration of sensor arrays.

##### Mathematica

Mathematica is a technical computing software. It was used by Kim et al. [71] to visualize the pressure profile of a piezoresistive sensor array. The sensor array was pressed and wrapped around a finger. It was also used by Wang et al. [314] to simulate the pyroelectric voltage in the proposed piezoelectric array.

##### Weka

Weka is a ML software for data mining that uses Java as its base language. Weka was used by Esposito et al. [53] to implement a wide variety of ML methods on sensor arrays.

##### SAS

SAS is an statistical software. Xu et al. [284] used it to manage the data and compare a proposed PWV sensor array with a state-of-the-art device.

##### The Unscrambler

The Unscrambler is a multivariate data analysis software. It has been used in several studies of sensor arrays [306,307].

##### CasaXPS

CasaXPS is a software that allows for spectral analysis. In the field of sensor arrays, it was used in the study of Gao et al. [239].

##### Nist Astar Calculator

The Nist Astar Calculator was publicly provided by the NIST (National Institute of Standards and Technology of the United States). In sensor array studies, it was used to calculate the linear energy transfer of a new radiation transistor [230].

#### 6.1.2. Finite-Element Analysis (FEA) Software

Different software tools for FEA have been used in sensor array studies.

##### COMSOL

COMSOL Multiphysics is a simulation software based on numerical methods. In sensor array studies, it has been used for different types of simulations:

- In triboelectric arrays, it is the standard for simulations of the electrical characteristics of the arrays [41,43,45,102,163,292,315,316,317,323,325,330,331,363].
- Simulation of the mechanical behavior of the sensor arrays [281,305,310,311] and their response to external stimuli [60,84,90,92,147,154,159,167,185,195,243,245,266,271,292].
- Simulations of changes in ECs [84,180,181,326].
- Testing ECT or CACT in capacitive sensor arrays [36].
- Solving fluid fields using Finite Volume Method [194].

##### SolidWorks

SolidWorks is a computer-aided design software widely used in various engineering fields, mainly for solid modeling. In the sensor array field, SolidWorks was used by Feng & Su [288] to design the sensing PDMS composite of a piezoelectric sensor array. In this regard, Mu et al. [160] used this software to design a special PDMS dielectric layer for pressure sensor arrays. Similarly, Kim et al. [260] used SolidWorks to design some parts of a piezoelectric array that were printed on a stereolithographic 3D printer. Wei et al. [7] also used it to perform flow field analysis for a chemiresistive sensor array. This test was performed to ensure that the target gases passed through the sensor.

##### ANSYS

ANSYS is a simulation software that includes FEA and modeling for a wide variety of applications. It has been used in several sensor array studies:

1. Simulation of complete sensor arrays [41,69,73,251,293].
2. Simulation of the distribution of piezoelectric sensors [278].
3. Calculation of Young’s moduli and Poison’s ratios of several materials [229].
4. Simulation of the distribution of mechanical stress on pieces [296,335,352] or sensors [160,223,296].
5. Optimization of the structure of the sensor array to reduce inter-element crosstalk [160].

##### ABAQUS

Abaqus is a unified suite of FEA products to solve engineering and industrial problems. This FEA simulation software was used in several works focused on the monitoring of structures using piezoelectric sensor arrays [4,300,301]. It is also a suitable option for FEA in the field of guided ultrasound waves [300]. The software allows simulation of the sensor arrays and structures to which they are attached at the same time.

##### ADINA

ADINA is a computer program for advanced analysis that includes FEA. It was used by Choi et al. [155] to analyze the characteristics of a capacitor as a function of its thickness and width. With this software, a tensile stress was applied, and a non-linear analysis was performed.

##### CATIA

CATIA is a computer-aided design software that is widely applied in several industries (automotive, aerospace, etc.) [337].

##### ConventorWare

This software is suitable for MEMS design and simulation with pre- and post-processing tools [199].

##### Non-Specified FEA Software

One set of studies used FEA tools, although the specific type was not mentioned:

- Several papers on capacitive sensor arrays studied the response of a sensor array to external forces or pressures [38,48,143,145,150,151,158,269]. The FEA allowed visualizing the distribution of the loads on the sensor array or on one of them.
- The work of Chattopadhyay & Chowdhury et al. [177] simulated the virtual fabrication steps of a MEMS capacitive sensor array in the FEA software.
- The work of Li et al. [2] simulated the reaction of a piezoelectric sensor array to exposure to VOC using FEA software.
- Finite-element simulation is common in fiber-optic studies [31,49]

#### 6.1.3. Electronic and Circuit Simulation Software

Several studies on sensor arrays have also used electronic and circuit simulation software.

##### SPICE

SPICE (“Simulation Program with Integrated Circuit Emphasis”) is a circuit simulation software. This software has been mainly applied to test new techniques to increase the readout accuracy of sensor arrays, as it can deal with real-life components with their corresponding non-idealities. It is possible to simulate the entire array, but also its readout circuit [69,75,107,117,119,121,128,129,135,231,370]. SPICE was also used to implement the equivalent electrical model of a sensor array [111,118,183,203]. Tang et al. [230] linked SPICE to Spectre and VerilogA to model the drain–source current behavior in a new transistor array. VerilogA was also used by Verma et al. [84] to implement a customized stress model.

##### NI Multisim

NI Multisim, which belongs to National Instruments, was also used for circuit simulation. Wu et al. incorporated NI Multisim in several studies focused on increasing the readout accuracy of resistive sensor arrays [97,120,122,124,125,126,127,138,370]. In this regard, Wang et al. [115] also used NI Multisim for the simulation of an improved version of a fast readout Zero Potential Circuit.

##### Proteus

Proteus ISIS is useful for the simulation of digital and analog circuits. Rashidi et al. [106], and Hasan et al. [105] presented a Nodal Array Approach that adopted Proteus for the implementation of the proposed circuit. Similarly, Zhang et al. [109] performed simulations of a readout circuit for resistive sensor arrays that was capable of compensating the internal resistance of analog multiplexers.

##### Ansoft

Ansoft is useful in the design stage of electronic, electrical, or RF devices, among others. Wang et al. [9] used it to calculate the coupling capacitances that exist inside the windings of a transformer. These coupling capacitances form the capacitive sensor array.

##### Silvaco

Silvaco can be used for electronic design automation and computer-aided design, including semiconductor technology. It was adopted in the work of Kundu et al. [220], who presented an array of light-dependent lattice-structure diodes.

##### Sentaurus

Sentaurus is a semiconductor device simulation tool. It was used by Hessel et al. [231] to simulate transistor arrays with different gate-oxide thicknesses. The study presented the relationship between the current of the drain source and the voltage of the cantilever.

##### Quartus

Quartus is a multi-platform environment suitable for FPGA design. This software was used in the work of Cui et al. [348], where an FPGA was incorporated to optimize an interrogation method for fiber-optic sensor arrays.

##### Sonnet

Sonnet software is suitable for high-frequency electromagnetic analysis, among other purposes. In the work of Nabovati et al. [197], it was used to evaluate the linearity of a capacitive sensor array on cell concentration.

#### 6.1.4. General-Purpose Programming Languages

This subsection presents the programming environments commonly used in sensor array studies.

##### Python

Python is a general-purpose, high-level programming language. In the context of sensor array studies, it has been used for several tasks:

1. Running control software for prototypes and experiments [176,198,202].
2. Signal processing tasks, such as denoising [35,198].
3. Training of AI models with Keras [40,141,366], scikit-learn [35], or Pytorch [85,182].
4. Implementation of numerical methods to mitigate crosstalk in sensor arrays [110,139,140]. These three studies presented optimization approaches. Python optimization packages were also used to develop tracking algorithms [361].
5. Implementation of user interfaces for data representation [35,75,153,154,161,185,198,200,247,346], and sensor operation visualization [330].
6. Automatic sensor characterization [201].

##### C Programming Language

In studies on sensor arrays, the C programming language is commonly used for embedded system applications. Several studies adopted it for the acquisition phase [69,206]. It was also used in the analysis and visualization phases via LabWindows/CVI software [50]. Several works have developed C++/C# custom applications to simultaneously configure and read data from the data acquisition card (DAQ). [35,82,103,109].

#### 6.1.5. Development Platforms and Tools

##### Arduino

Arduino is an open-source electronic prototyping platform. It has been used in several sensor array studies for the sake of simplicity in programming. Arduino is suitable for a variety of tasks: sensor reading [7,8,51,80,93,98,100,161,163,166,190,247,254,330,357,361], control [62,93,138,156,178,190,313], on-board data analysis [254], input system reading [53,326], data transmission by various means (Bluetooth [202], Wi-Fi [55], transmission from a dedicated capacitance-to-digital converter to a PC [168]), information display [220], IoT platform [55,313], etc.

##### Raspberry PI

The Raspberry PI is a type of single-board computer. It can also be considered an IoT platform [55,318]. It has been adopted in several sensor array studies. Yang et al. used a Raspberry PI to perform smart traffic monitoring [318]. Jeon et al. [55] proposed an IoT-oriented bending sensor array. In turn, Zhao et al. [8] used a Raspberry PI to perform switch control, readout processing, and reconstruction algorithms. It was used by Pani et al. [358] to read a Hall effect sensor array, control the position of a permanent magnet, and obtain the distance to the magnet in real time.

##### Smartphone Apps

Sensor array studies used smartphone apps, in most cases, to visualize the data. Yoon et al. [203] developed an Android application for the continuous monitoring of VOCs with a portable CMUT system. The data were sent via Bluetooth to a smartphone, which plotted the received data. Cheng et al. [56] implemented a similar application for a piezoresistive sensor array. Other studies [14,368] also used smartphone apps for visualization of the results. In this regard, Omary et al. [247] presented a piezoelectric sensor array for biomedical applications that interfaced with a smartphone using Arduino. Finally, Phyphox was used by Zhang et al. [44] to represent the output signals of a triboelectric array on an smartphone.

#### 6.1.6. Custom Software

This subsection includes customized tools developed by researchers:

- DeTECT (Demining Technology ECT): it was a new software presented in the work of Tholin & Soleimani [187]. It used a simulation method called the Finite Difference Method (FDM) to obtain the spatial permittivity distribution of a capacitive sensor array.
- In the work of Zafeirakis et al. [206] a customized software for visualization and frequency calculation was presented. It allowed measurement of the capacitance of a sensor array, sending the data to a remote computer via SSH, since it was running on an embedded Linux system.

#### 6.1.7. Other Software

This subsection includes other software found in studies on sensor arrays that could not be grouped into a single category:

- Automation control software: In the humidity sensor array system for the CERN’s high-energy detector [192], a WinCC Open Architecture SCADA (Supervisory Control And Data Acquisition) software was used to command the PLC involved in the control process.
- Wilcom Deco Studio: This software was used by Gleskova et al. [168] for the design of the electrode embroidery of an all-textile capacitive sensor array.

### 6.2. Brief Conclusion of Software for Analysis

**Figure 58:**
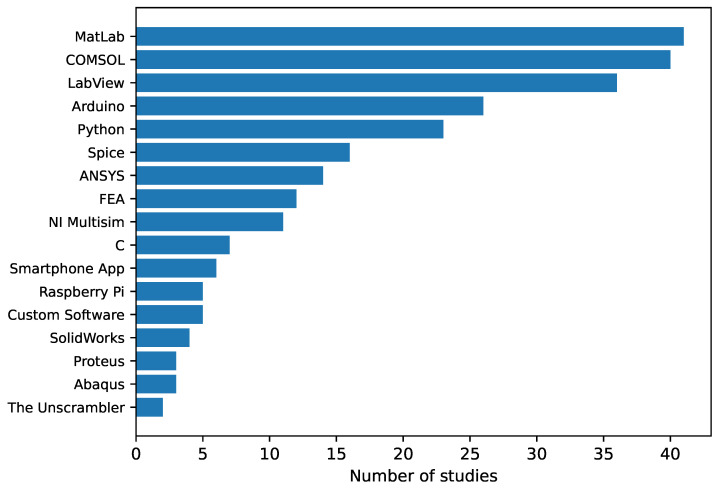
Number of sensor array studies classified by software tool. Only the most commonly used tools are represented.

Figure 58 represents the number of sensor array studies using the different software tools. MatLab, COMSOL, and LabView are preponderant, appearing in 11.4%, 11.1%, and 10.0% of the studies, respectively. Open-source solutions, such as Python, Spice, or Arduino platform, are also widely used in sensor array studies. Other software tools such as Abaqus or The Unscrabler are very dependent on the specific application, so they are used in isolated cases.

## 7. Sensor Array Characteristics

**Figure 59:**
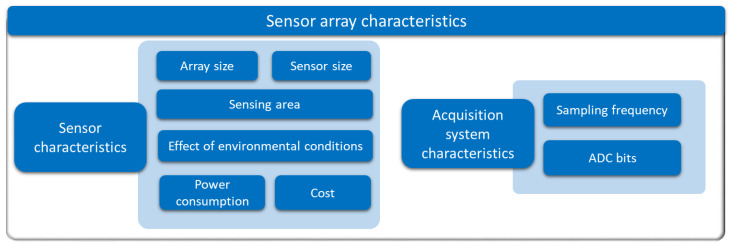
Sensor array characteristics.

This section analyzes the design characteristics of sensor arrays, which have been categorized into the following groups (Figure 59):

- Sensor characteristics. This group comprises different items: array size, sensor size, sensing area, effect of ECs, power consumption, and cost.
- Characteristics of the acquisition system. This includes the sampling frequency and the number of bits of the ADC.

### 7.1. Results of the Analysis

The sensor characteristics included in the most recent studies are presented in Table 6. The characteristics of the rest of the studies can be found in Table A3. The following subsections describe them in detail. All studies in Table 6 and Table A3 are explicitly cited in the corresponding subsections.

#### 7.1.1. Sensor Characteristics

This section presents the physical characteristics of the sensor arrays that were compared most frequently in existing studies:

##### Sensor Dimension

The analysis of this characteristic is provided in Table 1 and Table A1. Sensor dimension can be given with the following characteristics:

- **Array size**: An important comparison feature is the number of rows and columns in the sensor array. This parameter is almost always indicated in sensor array studies [8,12,15,20,30,31,32,33,34,35,36,37,38,39,40,42,43,45,46,47,48,49,50,52,53,55,56,57,58,60,61,64,65,67,69,70,71,73,75,76,77,78,79,81,82,84,85,87,88,93,94,96,97,98,100,101,104,105,106,107,108,109,111,112,113,114,115,117,119,120,121,122,123,124,125,126,127,128,129,132,133,134,135,136,138,139,140,143,144,145,146,147,149,150,151,152,155,156,158,159,160,161,162,163,164,165,167,169,172,173,174,180,181,182,183,185,186,188,189,190,193,194,196,197,198,200,201,204,205,210,211,212,213,214,215,216,218,221,223,224,225,229,230,233,236,237,238,240,242,243,244,245,248,251,252,255,256,259,260,261,262,263,268,269,270,271,272,274,280,281,283,285,287,288,297,304,305,306,314,319,320,323,324,325,327,328,330,332,334,336,340,342,344,346,348,349,353,357,358,359,361,363,365,368,370]. It is used specially for comparison in studies focused on improving readout accuracies. Studies that present new methods or techniques often test them on arrays of different sizes [8,12,15,20,50,53,55,56,57,58,60,61,64,65,67,69,70,71,73,75,76,88,94,97,98,104,105,106,111,112,113,114,115,117,119,120,121,122,123,124,125,126,127,128,129,132,133,134,135,136,139,140,150,151,152,155,156,158,159,160,161,162,165,167,169,172,173,174,180,181,188,196,197,198,200,204,205,210,211,213,216,221,223,225,229,230,233,252,256,259,260,261,262,263,268,270,272,274,283,287,288,297,306,320,323,324,325,332,334,348,349,353,357,359,361,368,370]
- **Sensor size**: The size of a single sensor in the array (a single cell) is also an important parameter. It is given as an area or as the length of the cell sides (length of the beam in the case of a fiber-optic array) [30,33,36,37,41,43,45,48,50,52,56,58,70,71,76,81,86,88,92,94,114,116,146,147,150,152,155,158,160,161,162,163,166,167,170,171,175,177,178,180,181,182,183,186,187,196,198,201,205,209,212,214,215,216,217,221,222,223,235,240,242,244,245,248,251,252,256,257,258,260,268,271,281,286,287,288,291,311,319,323,332,341,342,349,353,357,360]. This parameter affects the effective sensing area.
- **Sensing area**: It is the area covered by the whole array. A larger area does not necessarily means that the array has more sensors, as the sensor size must also be taken into account [7,8,10,12,36,42,56,57,58,61,63,65,66,70,71,75,81,82,88,92,94,112,116,142,144,150,151,155,156,160,161,162,167,168,171,173,181,182,186,188,209,210,215,216,222,223,224,225,226,230,232,233,240,243,244,246,248,253,254,255,256,258,259,260,261,268,269,282,287,291,292,298,305,314,320,321,324,327,328,330,331,340,349,353,357,359,360,361,366,367,370]. This parameter, together with the sensor size, determines the effective sensing area, which can be obtained from Equation (5).
  (5) $$EA=\frac{N\cdot M\cdot A_{S}}{A_{A}}$$
  where EA is the effective sensing area, *N* and *M* are the number of rows and columns of the sensor array, respectively, $A_{S}$ is the area of one sensor, and $A_{A}$ is the sensing area (the area covered by the entire array).

Another related parameter is the **spatial resolution**. It has been provided as the number of sensors per unit area [32,52,58,73,75,85,94,154,162,163,176,182,184,187,188,219,221,222,227,228,245,248,271,286,353,365,366], as the area covered by one sensor [38,304], or as the minimum distance between sensors [215,244,249]. It has been expressed in pixels per inch [228], dots per inch [191,368], or units per one-inch diagonal square [227]. This parameter can also be provided as the distance between pixels [85,154]. Sensors with high spatial resolution are found in submillimeter applications, such as cell detection (Section 4) or gas detection (Section Organic Compounds), among others.

Equation (6) shows one way to calculate the spatial resolution SR.

(6) $$SR=\frac{N\cdot M}{A_{A}}$$

Modular systems allow the dimensions of the sensor arrays to be expanded by connecting several sensing modules to the same DAQ [103].

##### Effects of Environmental Conditions (ECs)

ECs can influence the measurements of sensor arrays. The most studied environmental parameter in sensor array studies is temperature. Temperature may affect the performance of certain microelectronic devices. In this sense, temperature sensors were used in the work of Veske et al. [173] to study the effect of this parameter on the packaging of a device. Länge [12] studied the stability of piezoelectric crystals under temperature variations. Temperature can also affect fiber-optic arrays [337,350,352]. In this sense, Santamato et al. [31] compensated for the effects of temperature. They used an additional FBG for that purpose. Huang et al. [151] advised performance changes in their CMOS capacitive array under different temperatures. Park et al. [82] conducted a study of how temperature affected a pressure array, which was embedded in a car seat.

In some cases, temperature independence is desirable, as the sensor array must operate in different ECs and should show similar responses in all of them [1,34,44,52,59,64,71,79,84,86,92,99,111,118,156,166,172,176,177,192,193,203,204,205,208,217,220,235,245,254,279,287,311,325,333,340,350,352]. Several papers analyzed variations in the properties of sensor arrays as a function of temperature and/or humidity [163,208,238,251,297,327,334]. Pu et al. [179] and Wang et al. [297] used chambers to test their arrays in different temperature and humidity conditions. Li et al. [204] developed temperature- and humidity-independent sensors. Chattopadhyay & Chowdhury [177] used a differential circuit to eliminate the effects of temperature on the output signal. In the work of Hsu et al. [226], the thermal expansion coefficient was calculated and considered during the fabrication of a transistor sensor array. Husak et al. [77] analyzed the variation of each sensel model in the array in relation to temperature. They also performed an analysis of the effect of time.

Temperature also affects the ancillary elements of sensor arrays. In this sense, temperature influences some characteristic parameters of operational amplifiers (OAs) [113]. Similarly, electronic materials can undergo thermal expansion, which influences, for example, the packaging of products [173].

In some studies on sensor arrays, four types of temperature coefficients have been considered to evaluate the influence of this parameter on measurements:

- Temperature Coefficient of Frequency(TCF): The resonant frequency of certain sensor arrays can be sensitive to temperature variations according to Equation (7) [2,249,308,310], where $f_{0}$ is the fundamental resonant frequency of a pMUT and Δf is the frequency variation associated with a temperature variation ΔT.
  (7) $$TCF=\frac{1}{f_{0}}\frac{\Delta f}{\Delta T}$$
- Temperature Coefficient of Resistance (TCR): This coefficient is especially useful in studies of resistive sensor arrays [60,94,97]. It can be modeled by Equation (8) [94], where *R* is the sensor resistance at a reference temperature and $\frac{dR}{dT}$ is the derivative of the resistance versus temperature.
  (8) $$TCR=\frac{1}{R}\frac{dR}{dT}$$
- Temperature Coefficient of Sensitivity (TCS): The sensitivity of a sensor may also depend on temperature [151,167]. Chen et al. [332] conducted a study on the influence of temperature on strain measurements using fiber-optic arrays. Similarly, Zhang et al. [69] observed that their pressure piezoresistive array changed its sensitivity for different temperatures.
- Temperature Coefficient of Offset (TCO): In the work of Hsieh et al. [167], the TCO was provided for a capacitive pressure array. The TCO indicated the base capacity value in the absence of pressure for a range of temperatures. In this case, the coefficient was −3.79 fF/°C.

Other environmental parameters that have been studied are humidity and magnetic field. On the one hand, Li et al. [319] submerged a triboelectric sensor array under water to test whether its conductivity was affected. In fact, humidity is a relevant factor in triboelectric sensor arrays [42,327,328,329]. On the other hand, Zhang et al. [43] quantified the effect of the surrounding magnetic field (including that of the Earth) on a triboelectric sensor array.

##### Power Consumption

Power consumption is usually provided in sensor array studies and is essential in portable devices. Several papers highlighted the need for low-power sensor arrays [7,10,35,53,62,69,73,75,80,88,92,113,117,128,131,137,143,146,150,179,184,186,202,215,269,329]. In this sense, sensor arrays manufactured in CMOS technology are usually of low power [58,84,151,193,196,197,233,235]. In resistive array studies focused on increasing acquisition accuracy, there is a trade-off between power consumption and system performance [96,108,109,125,127].

Power generation is studied especially in triboelectric arrays [39,40,42,44,45,61,63,102,317,318,319,320,321,322,323,324,325,326,330,331] and piezoelectric arrays [63,156,242,243,246,247,248,250,251,253,259,261,264,269,281,282,285,305,313,314], as these technologies have the potential to self-harvest. Table 6 and Table A3 include the generated power values (labeled as *generated* in those tables).

##### Cost

Reducing the cost of sensor arrays is a priority for several state-of-the-art studies.

Depending on the sensor array technology, there are different approaches. In piezoresistive sensor arrays, it is common to use commercially available materials for mat implementation [3,55,57,59,68]. In fiber-optic technology, several authors have incorporated low-cost fiber-optic options [1,332,334,339,343,346,348,354]. In [347], TDM is shown to be both a cost-effective and efficient technique for the readout of fiber-optic sensors. However, some fiber-optic reading methods can be costly and complex [49]. In capacitive sensor arrays, Weichart et al. [176] focused on the fabrication process to limit the cost of the system. In fact, several studies from all technologies developed low-cost sensors [357], some of them using cheap materials that did not require specialized machinery [18,40,51,66,77,81,87,94,102,133,143,144,148,155,158,159,160,164,168,170,171,179,181,183,188,200,204,246,284,291,292,307,328,362] or cost-effective manufacturing options [43,47,65,85,93,101,108,185,201,213,214,271,293,319,327,344,353,355,360]. The use of well-established manufacturing techniques has a positive impact on the cost of the system [146]. This is the case for CMOS [19,58,151,193,196,205,231] or MEMS manufacturing processes [48]. Other alternatives for manufacturing low-cost transistor-based sensor arrays can be found in [58,76,94,226,229,231,234,237,239]. Complex DAQs significantly increase cost, especially in resistive arrays. There is often a trade-off between cost and accuracy [128,135,136].

Other existing studies have achieved low-cost prototypes by including commercial sensors [78,98,100,114,215,362] or low-cost commercially available microcontrollers for the processing part of the prototypes (resistive examples in [8,75,80,103,106,127]; piezoresistive examples in [64,140]; capacitive examples in [168]; and piezoelectric examples in [257,300]).

#### 7.1.2. Acquisition System Characteristics

The part of the acquisition system that is typically compared in sensor array studies is the digital-to-analogue converter. It is responsible for converting analogue electrical signals from sensor arrays into digital signals for the processing module. The most common characteristic parameters of an ADC are the number of bits and the sampling frequency.

##### Number of ADC Bits

Several sensor array studies provided the number of ADC bits [4,33,35,75,78,81,84,103,108,124,126,138,140,153,154,184,214,215,244,252,276,277,337,342,363].

Some studies indicated the effective number of bits (ENOB) instead [84,130,178]. This number is the resolution actually used in the system. It is always less than the number of ADC bits due to the presence of noise, crosstalk, or other types of uncertainty [130].

Several studies that provided the number of ADC bits used low-cost commercial microcontrollers [8,64,75,82,93,106,109,127,140,168,206,257], such as Arduino, STM32, ESP32, or Raspberry Pi platforms. Other studies used FPGA-based DAQs, such as Xilinx XC6SLX9 [181] or Spartan 3 FPGA (XC3S50AN-4TQG144C) [130,131]. In addition, several studies used NI PXI-series converters [147,151,198,199,224,312,330,364] and USB-6 DAQs [1,53,147,243,268,328].

In studies focusing on readout systems, the number of ADC bits is a relevant parameter, since the maximum allowable noise level is 1/2 LSB (least significant bit; see Equation (9)).

(9) $$1LSB=\frac{V_{MAX}-V_{MIN}}{2^{N}-1}$$

where $V_{MAX}$ is the maximum ADC voltage, $V_{MIN}$ is the minimum ADC voltage, and *N* is the number of ADC bits. The higher the supply voltages, the better the noise immunity [8,75,105,106,112,120,130,132,134,139].

##### Sampling Frequency

The sampling frequency determines the rate at which the acquisition system converts analog signals to digital values [2,4,9,30,32,53,75,78,80,81,84,85,88,92,93,99,100,104,105,106,110,116,121,129,140,147,148,149,151,153,154,155,159,168,170,181,182,183,184,186,187,188,196,199,202,205,207,212,215,228,240,255,256,257,258,260,263,266,268,273,276,277,280,286,289,291,293,298,299,301,305,306,321,329,335,337,339,349,359,361,362,363]. In studies focused on analyzing the effects of array size on sampling frequency, it can be provided as a function of the number of elements in the array [103,138,140]. In fiber-optic arrays, it depends on the interrogator used [1,33,49,332,333,336,338,341,342,348]. Ghamsari et al. [104] proposed a method to significantly reduce the sampling rate of a resistive sensor array. Ghouchani et al. [108] presented an adaptative sampling algorithm based on regions of interest that improved on the one proposed by Ghamsari et al. [104].

### 7.2. Brief Conclusion of Sensor Array Characteristics

**Figure 60:**
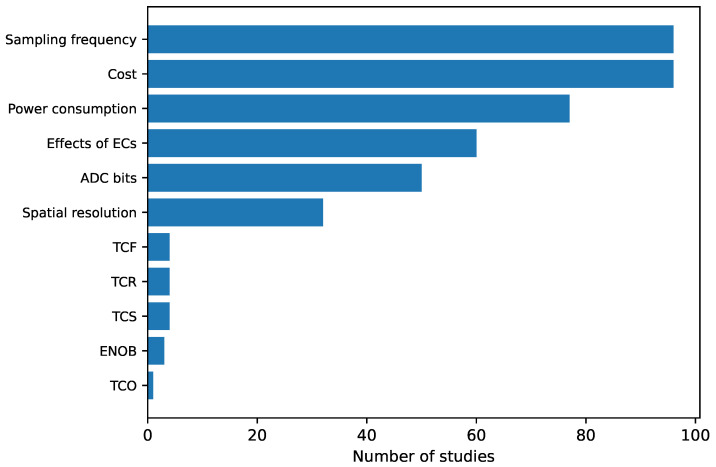
Number of sensor array studies that provide the different sensor array characteristics. Sensor dimension appears in almost 100% of the studies, so this feature has not been represented in the graph.

Figure 60 represents the number of studies that provide the most common sensor array characteristics. The sensor dimension (array size, sensor size, or sensing area) is provided in almost all studies. Cost, sampling frequency, and power consumption are also concerning aspects in this area, since they appear in 26.6%, 26.6%, and 21.3% of the studies, respectively. Another important characteristic in the design of sensor arrays is the number of ADC bits, as they represent the overall resolution of the system. The effects of environmental conditions on measurement performance have also been widely studied.

Finally, Table 7 shows some aspects related to sensor array characteristics to be considered by researchers in this field.

## 8. Sensor Array Performance Metrics

**Figure 61:**
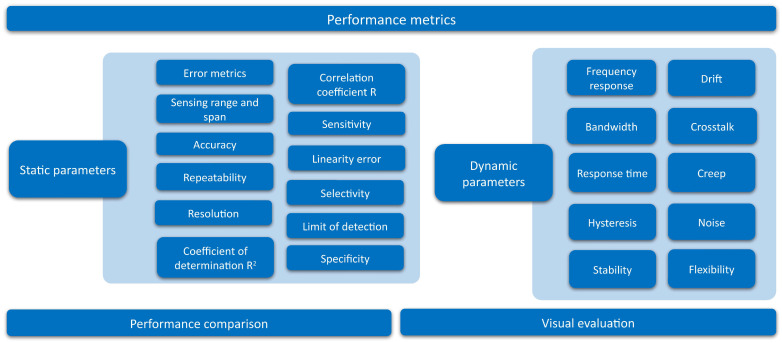
Sensor array performance metrics.

This section analyzes the performance metrics of sensor arrays. They have been classified into the following groups (see Figure 61):

- Static performance metrics: These include error metrics, sensing range and span, accuracy, repeatability, sensitivity, resolution, coefficient of determination, correlation coefficient, linearity error, selectivity, limit of detection, and specificity.
- Dynamic performance metrics: This group considers frequency response, bandwidth, response time, hysteresis, stability, drift, crosstalk, creep, noise, and flexibility.

### 8.1. Results of the Analysis

Table 6 presents the sensor characteristics and performance metrics for the most recent state-of-the-art studies. The analysis of the rest of the studies can be found in Table A3. The following subsections describe them in detail. All studies in Table 6 and Table A3 are explicitly cited in the corresponding subsections. Studies that use more than one performance metric are cited in all associated subsections.

#### 8.1.1. Performance Metrics: Static Parameters

This section presents the results of the analysis of the static performance metrics used in sensor array studies.

##### Error Metrics

Different metrics were used to provide the difference between the measured value *y* and the true value $y^{\prime }$ in sensing arrays. Some studies differentiated between the error of a single element of the array and the error of the entire array [31,63,66,69,105,106,166,178,183,252,265]. These cases are explicitly indicated in Table 6 and Table A3. For the rest of the studies (which are the majority), the error values shown values refer to the entire array.

**Single Error:** This is the difference between the measurement *y* and the real value $y^{\prime }$ [91,195,301,339,358,361] (Equation (10)). Single errors are not usually employed, as an average value is normally preferred.

(10) $$E=y-y^{\prime }$$

**Absolute Error (AE):** This is calculated according to Equation (11). Since it is not a metric that reports average values, it is not frequently used in sensor array studies [3,37,50,56,79,97,120,127,130,131,133,152,179,280,297,298,299,352,361,365]. This variable is used to provide the maximum error of a sensor array system.

(11) $$AE=|y-y^{\prime }|$$

**Relative Error (RE):** The relative error is expressed in Equation (12). Several studies have incorporated this metric [31,69,91,101,103,113,115,120,121,122,123,124,125,126,127,136,189,192,193,257,273,317]. This variable is used to provide the maximum error of a sensor array system [103].

(12) $$RE=\frac{y-y^{\prime }}{y^{\prime }}$$

**Absolute Relative Error (ARE):** Equation (13) represents the ARE calculation included in several sensor array studies [9,63,66,67,75,88,96,97,102,109,110,112,114,117,119,128,129,130,131,134,135,136,139,140,166,181,223,272,273,288,298,332,338,344,352,363]:

(13) $$ARE=\frac{|y-y^{\prime }|}{y^{\prime }}$$

This variable is used to provide the maximum error of a sensor array system in the input range considered [79,107,138].

**Mean Relative Error (MRE):** Since this is the averaged relative error for the *N* measurements (Equation (14)), it is usually given together with the associated standard deviation in the following form: MRE±σ. This metric has only been provided in two studies [12,102].

(14) $$MRE=\frac{1}{N}\sum_{i}^{N}\frac{y_{i}-y_{i}^{\prime }}{y_{i}^{\prime }}$$

**Mean Absolute Error (MAE):** MAE is calculated in Equation (15) as the absolute error averaged over *N* measurements [102,153,178,183,246,284,302,337,340,359,360,361,363,366].

(15) $$MAE=\frac{1}{N}\sum_{i}^{N}\left|y_{i}-y_{i}^{\prime }\right|$$

**Mean Absolute Relative Error (MARE):** The MARE is calculated in Equation (16) as the ARE is averaged over *N* measurements. It is one of the most widely used metrics [4,78,105,106,110,118,139,141,152,183,194,235,265,294,302,340,363,365].

(16) $$MARE=\frac{1}{N}\sum_{i}^{N}\frac{|y_{i}-y_{i}^{\prime }|}{y_{i}^{\prime }}$$

**Mean Squared Error (MSE) and Root Mean Squared Error (RMSE):** Equations (17) and (18) show the calculation of MSE and RMSE, respectively.

(17) $$MSE=\frac{\sum_{i}{\left(y_{i}-y_{i}^{\prime }\right)}^{2}}{N}$$

(18) $$RMSE=\sqrt{MSE}=\sqrt{\frac{\sum_{i}{\left(y_{i}-y_{i}^{\prime }\right)}^{2}}{N}}$$

RMSE is a common metric in sensor array studies [3,8,33,36,41,51,74,98,100,132,133,134,139,144,252,255,294,300,337,366], while MSE [35,36,97,111,363,365] and the normalized RMSE (NRMSE) [3,80] are used in isolated cases.

##### Sensing Range and Span

The sensing range is the interval of values that the sensor array can measure (Equation (19)). The span can be obtained as the difference between the extreme values of the interval, $X_{0}$ and $X_{1}$ (Equation (20)). Sensor array studies commonly provide the range or span to indicate the operating values of the parameter of interest [2,3,5,8,9,10,35,37,39,42,48,50,52,56,57,59,60,61,63,64,65,66,67,68,69,71,72,73,74,75,77,84,86,87,88,91,92,94,95,97,99,111,112,113,114,115,116,118,119,120,121,122,123,124,125,126,127,128,129,130,131,132,133,134,135,136,137,139,140,143,144,145,149,150,151,152,154,155,156,158,159,160,161,163,164,166,167,168,170,172,173,174,175,176,177,178,179,180,181,183,184,185,186,187,191,192,196,199,205,208,216,219,220,221,222,224,225,228,229,232,233,234,236,237,240,245,247,250,251,252,256,257,265,273,286,288,292,296,307,310,314,316,318,324,325,327,329,330,332,334,336,337,338,339,343,348,352,358,359,360,361,364]. Zhang et al. [78] developed a method to extend the measurement range of a resistance array up to 1.5 times that of the reference method.

(19) $$Range=[X_{0},X_{1}]$$

(20) $$Span=X_{1}-X_{0}$$

##### Accuracy

Accuracy is a key metric in sensor array studies [10,56,120,148,164,178,203,235,257,272,276,277,300,350,357,358]. Different definitions of the accuracy metric have been found in sensor array studies. On the one hand, in classification/detection studies using ML or AI techniques [41,42,44,83,85,184,236,238,255,306], it is given as the ratio of correctly classified items to the total number of items (Equation (21)). In these cases, the most common way to provide information on accuracy is through a confusion matrix [2,39,40,42,45,53,68,85,149,163,182,183,185,186,213,214,245,275,328,329,356].

(21) $$acc\left(\%\right)=\frac{i_{cc}}{i_{cc}+i_{ic}},$$

where $i_{cc}$ and $i_{ic}$ are the number of correctly and incorrectly classified items, respectively.

On the other hand, other works refer to the accuracy of measurements from the instrumentation point of view [179,184,190,210,341,352]. In the work of Ha et al. [217], accuracy refers to the number of decimal units that the sensor array system can measure. In other contexts, accuracy is the complementary value of the ARE, according to Equation (22).

(22) acc(%)=1−ARE(%)

##### Repeatability

Repeatability in sensor arrays measures the ability of systems to behave similarly when performing the same experiments over different time periods. It is closely related to the uncertainty of the measurements [332]. Repeatability has been expressed in sensor array studies in several ways.

Firstly, it can be provided as a standard deviation σ (or a variance $\sigma^{2}$). Equation (23) is used to calculate the standard deviation of a measurement, where $y_{i}$ is each measured value and $\bar{y}$ is the mean of these values.

(23) $$\sigma =\sqrt{\frac{\sum_{i}{\left(y_{i}-\bar{y}\right)}^{2}}{N-1}}$$

On the one hand, this metric shows the variability between measurements from individual sensors in the same array, which, ideally, should be the same for the same input stimulus. On the other hand, this metric can be calculated from several measurements of the same sensing element taken under the same conditions but at different times [66,80,88,142,201,237,242,252,255]. These deviations can lead to what is called fixed-pattern-noise (see Section “*Noise*”). A high value of the standard deviation indicates that the sensor array system has low repeatability. Several works have quantified repeatability in this way [10,53,56,61,64,66,71,76,78,81,96,110,118,152,153,156,161,174,175,176,186,201,213,214,221,242,248,252,256,284,310,329,336,337,346,359,360,366,367]. It can also be expressed as a relative standard deviation (in percent) [3,69,76,82,88,98,101,103,142,144,163,186,225,237,265,267,275]. It can be given as a percentage in classification problems [307]. The work of Oballe-Peinado et al. [130] studied the uncertainty of the measurement in resistive sensor arrays expressed as a variance value. Uncertainty analysis of the measurements in a PSM is also performed by Martinez et al. [140].

Secondly, several studies on sensor arrays represented repeatability in box-and-whisker plots (labeled as “visually” in Table 6 and Table A3) [2,30,31,38,40,43,50,59,91,92,94,99,102,146,159,167,168,193,199,205,210,211,212,222,229,234,235,239,240,241,243,246,251,254,256,257,262,263,271,286,294,318,323,360,361]. These bars can represent standard deviation or confidence intervals.

Finally, cyclically testing the sensor is a common way to assess repeatability [6,54,61,62,63,68,80,93,137,145,150,158,162,169,171,172,178,185,204,208,227,232,261,273,316,350,355,366]. Thus, in some works, repeatability and stability are studied with the same experiments [56,174,185] (see the section entitled Stability). A possible alternative is to simulate different scenarios several times [117,130,131,139,140], or manufacture several of the same devices and test them separately [95,222,239]. It can also be determined by testing with several subjects [244,286].

##### Sensitivity

This metric is widely used in sensor array studies [1,2,3,5,6,7,10,31,32,37,38,39,41,42,43,46,47,48,49,50,52,55,56,57,59,60,61,62,63,64,65,66,68,69,70,71,72,73,76,82,83,85,86,87,88,89,90,91,92,93,94,95,96,97,99,103,111,116,118,137,142,143,144,145,146,147,149,150,151,152,153,154,155,156,157,158,159,160,161,162,163,166,167,169,172,174,175,176,178,180,181,185,187,188,191,192,194,196,197,198,199,200,201,203,204,206,208,209,210,211,212,213,215,216,218,220,221,222,223,224,225,226,228,229,230,231,232,233,234,235,239,240,241,242,243,244,245,246,247,248,249,250,251,252,253,254,255,256,257,258,261,262,263,264,265,266,267,268,269,270,271,274,283,285,288,305,307,308,309,310,312,315,318,319,324,325,326,327,330,331,332,333,334,336,339,342,343,344,348,349,356,359,360,364]. It shows the influence of the parameter of interest *X* on the output variable of the sensing array *Y* (voltage, current, etc.). Many applications of the sensor array require high sensitivity. The higher the sensitivity, the greater the change in the sensor array output variable *Y* for small changes in the parameter of interest *X*. If the response of the sensor array is not linear, the sensitivity can be obtained with Equation (24) [16]. However, if the response is linear within a range, Equation (25) can be used. This is the slope of the linear curve. Even if the relationship is not linear, it is usual to perform a linear fit for different ranges of the parameter of interest. For example, Wang et al. [324] presented a triboelectric array with three different pressure ranges. For each pressure range, the pressure–voltage relationship was different.

(24) $$S=\frac{\partial Y}{\partial X}$$

(25) $$S=\frac{\Delta Y}{\Delta X}$$

The sensitivity of a classification system can be obtained using Equation (26) [17,18,19,77,214,307]. In this equation, TP are the true positives (number of items that the system has correctly classified as the item of interest), and FN are the false negatives (number of items that the system does not classify as the item of interest when they really are). An example of the “item of interest” is *Helicobacter pylori* in [307].

(26) $$SE_{C}=\frac{TP}{TP+FN}$$

Alternatively, other sensor array studies provided sensitivity-related metrics. On the one hand, several studies focusing on chemical sensor arrays [2,102,203,307] mention cross-sensitivity as a metric to evaluate the effects of non-target chemicals on sensor array measurements. On the other hand, Chowdhury et al. [111] focused on the sensitivity of the readout circuit. The sensitivity of the output voltage of the operating amplifier was analyzed.

Some studies differentiated between the sensitivity values of a single element of the array and those of the entire array [1,3,6,7,31,56,59,61,63,66,69,73,88,91,99,147,161,162,166,173,178,201,203,206,210,211,218,223,225,228,242,243,244,250,251,252,255,256,266,267,271,283,305,306,318,324,336,343,344,364]. A common way to represent the results is to plot the response of several individual sensors to the same stimulus on the same graph (labeled “visually for each individual sensor” in Table 6 and Table A3). Studies then usually provide an average value. This differentiation is good practice, as some array-related effects, such as crosstalk (Section Crosstalk), parasitic effects of non-sensitive elements, [147,250] or non-idealities due to the manufacturing process, [31,61,63,95,271,324] can cause the array to exhibit sensitivity values different when assembled in a matrix configuration. The studies that made this distinction are indicated in Table 6 and Table A3. For the rest of the studies, the sensitivity values refer to the entire array (since they do not analyze the sensing elements individually).

##### Resolution

The resolution determines the minimum change in the parameter of interest that has an effect on the output of the sensor array. High resolution is desirable for many applications. It is normally related to the number of bits in the ADC and its range (see Section Number of ADC Bits) [64,75,84,105,130,132,151,168,181].

The resolution is usually expressed in units of the parameter of interest [3,10,54,58,71,73,85,92,93,94,95,96,99,114,127,133,147,154,162,166,170,173,174,176,178,180,187,188,191,196,204,208,216,217,219,221,222,227,228,231,246,267,269,279,286,294,299,309,310,314,315,329,332,333,334,337,349,353,360,362]. It can also be provided as a percentage of the nominal value to be measured [103] or as the result of dividing the sensing range by the number of bits of the ADC [84]. Some studies differentiated between the resolution of a single element of the array and the resolution of the entire array [166,353]. These cases are indicated in Table 6 and Table A3.

##### Coefficient of Determination (CD, $R^{2}$)

The coefficient of determination ($R^{2}$) determines the degree of fit of a model to experimental data [300]. It has been used in sensor array studies that perform linear [2,7,35,39,43,48,60,68,75,80,82,86,144,145,152,159,160,163,174,183,186,199,205,212,218,224,225,233,237,248,255,257,258,267,275,300,317,324,326,327,329,331,334,348,353,355,362], polynomial [3,83,193,257,307,317], and exponential fitting [74,98,310]. The coefficient of determination ranges from 0 to 1: $R^{2}=0$ means that there is no correlation between the model and the experimental data, while $R^{2}=1$ corresponds to a perfect fit. It can be calculated according to Equation (27) [98]. In this equation, $f_{i}$ is the value provided by the fit, $y_{i}$ is the measured value, and $\bar{y}$ is the mean of all measured values.

(27) $$R^{2}=1-\frac{\sum_{i}{\left(y_{i}-f_{i}\right)}^{2}}{\sum_{i}{\left(y_{i}-\bar{y}\right)}^{2}}$$

This metric can also be used to evaluate classification systems [101].

##### Correlation Coefficient (*R*)

The correlation coefficient *R* can be calculated as the square root of the coefficient of determination $R^{2}$ [98,257,284,355,366]. It is also called Pearson’s coefficient [7,337]. It indicates the degree of relationship between two variables, and ranges from −1 to 1. The closer it is to 1, the stronger the positive (direct) relationship; the closer it is to −1, the stronger the negative (inverse) relationship. R=0 would indicate no linear relationship between the variables. The correlation coefficient between two vectors of variables $\vec{a}$ and $\vec{b}$ can be calculated according to the Equation (28) [98], where $a_{i}$ and $b_{i}$ are the values of each vector and $\bar{a}$ and $\bar{b}$ are the mean values of each vector.

(28) $$r=\frac{\sum_{i}\left(a_{i}-\bar{a}\right)\left(b_{i}-\bar{b}\right)}{\sqrt{\sum_{i}{\left(a_{i}-\bar{a}\right)}^{2}}\sqrt{\sum_{i}{\left(b_{i}-\bar{b}\right)}^{2}}}$$

This metric can also be provided as a percentage [77]. The correlation coefficient can be used to compare the performance of a sensor array with other measurement systems [336,355], or a theoretical approach with real-life implementations [31,36,189,337]. It was also used to find a relationship between the measurements of different taxels in the same array [34,194]. Barzegar et al. [304] used the 2D correlation coefficient as a visual metric to detect defects in materials.

##### Linearity Error

The linearity error indicates the degree of proportionality between the sensor measurement and the physical parameter of interest [69,223]. It can be provided as a percentage [50,167,170,181,216,242,265,279,314], or as an RMSE [255]. For some applications, it may be interesting to have an indication of how differently the sensor array system behaves with respect to a linear system. This can help determine whether a linear approximation is acceptable or not. Si & Wang [294] assessed the quality of their linear fit using RMSE and the *p*-value of Student’s *t*-test. Li et al. [79] quantified the nonlinearities of a digital-to-analog converter in less than 1LSB.

##### Selectivity

In the context of sensor arrays for classification applications, selectivity can be understood as the ability of the system to correctly classify one specific item among all possible items [2,12,14,16,98,203,225,232,233,234,240,307,355].

Alternatively, in the context of studies focused on solving the problem of crosstalk in sensor arrays [112,326], a system can be considered selective if crosstalk is low. This means that the excitation of one sensor has no significant effect on the adjacent sensors of the array [94,326]. The work of Ahmed et al. [326] provided an expression (Equation (29)) to calculate the selectivity in terms of electrical variables.

(29) $$Selectivity=\frac{N_{max}-N}{N_{max}}$$

where $N_{max}$ is the maximum voltage or current provided by an excited sensor, and *N* is the voltage or current that appears on the same sensor when an adjacent cell is excited.

##### Limit of Detection (LOD)

LOD determines the minimum value of the parameter of interest that the sensor array can measure. Below this minimum value, the sensor array does not provide a perceptible response. Low-LOD sensor arrays are required for applications that measure small-value magnitudes (low stress detection, sensing of low concentrations of VOCs in the air, etc.) [2,7,37,47,57,66,68,83,92,93,99,101,142,143,147,149,150,158,159,160,163,175,185,186,188,191,202,208,223,225,232,234,235,236,237,238,239,240,253,271,286,310,324,326,327,355]. It usually matches the lower value of the span (see Section Sensing Range and Span).

##### Specificity

It is an important figure-of-merit in classification tasks [153,213,214,239,255,307]. It is related to the classification sensitivity (Equation (26)), and is calculated by Equation (30), where TN represents true negatives and FP represents false positives.

(30) $$SP_{C}=\frac{TN}{TN+FP}$$

#### 8.1.2. Performance Metrics: Dynamic Parameters

This subsection presents the dynamic performance metrics of sensor arrays.

##### Frequency Response

Frequency response analysis is performed in systems subjected to varying external stimuli, such as piezoresistive arrays [3,64,67,85,99], capacitive arrays [9,37,61,92,146,150,151,159,160,162,170,171,173,174,177,180,183,196], triboelectric arrays [39,40,41,42,44,45,315,318,319,320,321,322,323,324,325,326,327,328,330,342], piezoelectric sensor arrays [4,5,241,242,245,246,247,250,251,252,254,255,257,258,259,261,265,271,272,275,276,277,278,281,283,285,287,289,290,291,292,294,296,297,298,301,302,303,304,306,308,309,310,311], and bioimpedance sensor arrays [367]. It has also been used in light-based technologies, such as fiber-optic sensor arrays [1,31,32,33,49,332,336,338,339,340,343,347,349] and diode sensor arrays [220].

Frequency response analysis has also been adopted in studies that detect oscillatory motion or vibration, such as those based on IMUs [13], those focused on structural health monitoring [4,170,171,261,272,291,292,296,298,302,335], UXO detection [216], magnetometry [364], or those measuring periodic physiological signals [85,92,150,159,177,246,257,366].

Frequency response analysis is also useful in electrical applications. In this sense, Kundu et al. [220] tested a diode array under different wavelengths. A subset of studies [67,208,209,210,211,213,214,216,308] considered AC voltages and currents, performing cutoff frequency analysis or frequency response analysis.

##### Bandwidth (BW)

The BW indicates the frequency range in which sensor arrays can operate without distortion. This metric is usually accompanied by an analysis of the frequency response Section Frequency Response. Depending on the sensing technology, the BW may provide different information. It is a common metric in fiber-optic sensor arrays, as they are high-frequency systems. In this context, the BW is often used to determine the value of the physical parameter [1,32,332,338,339,341,342]. In piezoelectric technology, the BW determines the frequency range at which the sensor arrays can operate [218,242,258,265,276,277,290,292,303,305,309]. It is especially interesting in sensor arrays that function as resonators [99,180]. It is also useful in capacitive sensor arrays. For example, Wang et al. [174] measured the dynamic strain and provided the operating BW. Wang et al. [9], the BW of different capacitive sensors designed to measure high-frequency voltages in transformers is compared. In inductive sensor arrays [216], the bandwidth was used to provide information about the allowed range of oscillating electromagnetic fields.

Although existing studies usually provide information on the BW of the sensor arrays themselves, several studies also focused on the acquisition system. In fact, it is common to provide the BW of the OA, as it limits the BW of the entire system [115,122,123,124,125,126,127,129,135]. In other cases, the BW of the entire system is limited by the implemented filter [3,276,277,291].

##### Response Time

This metric indicates the time required for a sensor array system to provide stable measurement. Some studies distinguish between response time and recovery time, which is the time it takes for the sensor array to stabilize its output after removing the stimulus [38,39,46,47,84,143,149,155,158,159,161,163,164,185,212,224,248,251,325]. In Table 6 and Table A3 these two values are separated by a slash. In this sense, these are common metrics in piezoresistive [37,46,47,54,56,57,59,68,70,75,82,83,85,86,87,88,93,99,101,107,114,128,245,337], capacitive [38,88,93,142,144,145,149,150,155,156,158,159,160,161,162,164,165,172,174,175,181,182,191,197,202,203,204,219], piezoelectric [2,6,156,246,254,255,258,288,303,310,312,314], or inductive technologies [208,214].

Meanwhile, it is not as relevant for transistor-based [76,221,222], and triboelectric technologies [30,39,40,41,42,43,163,318,326,327,328], since they usually have a low response time. However, transistor-based chemical sensors, such as ISFETs, can be slow [233]. It is desirable to have sensor arrays with a low response time, as this metric limits the maximum sampling rate of the system. In addition, if signals from the sensor arrays are post-processed, the time required for this operation is often considered part of the response time of the entire electronic system.

Finally, the response time has also been provided sporadically in studies on diode and fiber-optic sensor arrays. For example, the diode-based system proposed in the work of Yeom et al. [137] for pressure measurement presented a moderate response time of 70 ms. In a completely different context, the fiber-optic study by Ren et al. [350] measured the response time of an anti-vandalism system. The response time was defined as the time it took for an alarm to sound, rather than focusing on the physics of the array itself. Yang et al. [1] measured the response time of the fiber-optic sensor to a heating stimulus and found it to be less than 0.2 s. A delayed response is associated with the creep effect (see the section entitled Creep).

##### Hysteresis

The hysteresis metric is relevant for sensor arrays that provide different measurements for the same value of the parameter of interest, depending on whether that value was reached with the parameter of interest increasing or decreasing. It is mainly studied in pressure arrays [318]. Hysteresis is usually represented by superimposing the load and unload curves. In relation to sensing technologies, hysteresis usually occurs in capacitive [10,20,61,63,93,142,144,150,161,168,169,172,173,175,176,178,191] and piezoresistive arrays (their internal structure deforms under physical stress) [20,47,51,54,56,59,63,65,69,71,72,76,80,82,93,116].

It has also been analyzed in several inductive, piezoelectric, and transistor-based studies. In this sense, the inductive sensor array proposed by Yeh & Fang [210,211] presented hysteresis associated with the specific polymer used. Meanwhile, in the piezoelectric sensor array proposed by Nagayama et al. [282], hysteresis was evaluated by applying an electric field to the material and measuring the current density across it. In addition, transistor-based arrays recorded low hysteresis values in pressure [76,221,222,368] and temperature applications [95].

Several studies on sensor arrays have focused on reducing hysteresis. For example, Pyo et al. [172] presented a capacitive sensor array without hysteresis, while Zhang et al. [69] applied a Genetic Algorithm Wavelet NN to reduce hysteresis levels, and Jang et al. [322] developed a hysteresis-free piezoelectric array for gesture recognition. Sun et al. [310] compared several works in terms of hysteresis. Finally, in the piezoresistive array presented by Choi et al. [83] hysteresis was significantly reduced by pre-straining the material. It has been related to repeatability (see Section “*Repeatability*”) of the materials [65].

##### Stability

An stable sensor array provides the same output for a constant input. Stability is usually tested by repeating the same experiments with the same sensor array and analyzing the performance between repetitions. For this reason, the number of cycles before abnormal operation is normally indicated. It may be related to the durability of the sensor arrays [315]. This metric is important for sensor arrays subjected to long periods of operation [10,30,34,37,39,40,42,43,44,46,52,56,57,59,61,62,65,68,70,71,72,76,85,86,87,92,94,97,102,114,137,142,144,149,150,156,158,159,160,172,174,175,176,178,185,191,192,203,204,208,212,216,218,221,225,232,233,235,243,245,246,247,248,250,251,253,254,255,258,259,264,272,305,307,310,318,320,323,324,325,326,327,328,329,330,331,337]. Some sensor arrays, designed to be unaffected by certain types of loads, are nonetheless tested under those conditions to confirm their immunity [212,319]. In VOC-sensitive arrays, which have long response times, it can be measured in days [98,236].

In this sense, Li et al. [258] evaluated the stability and reliability of an impact-detecting safety helmet based on piezoelectric sensor arrays. For that, they obtained the Intra-Class Correlation coefficient (ICC). The coefficient of variation (CV) was used in [12,78,88,99,258,284,355] for similar purposes.

##### Drift

Drift is an slow variation of the sensor array output over an extended period of time. It is simply due to the operation of the sensors themselves. For example, several studies quantifying drift in their sensor arrays reported that the sensor array output changed at a rate of 5 mV/h [233]. or 0.01% in 7 days [213]. Thus, it is related to the stability of the sensor arrays (see Section Stability) [176]. Based on the nature of the drift, it is sometimes referred to as “baseline drift”, as it can be represented as a low-frequency trend [3,238,244,257]. For this reason, it is usually removed by filtering [3,240].

Several studies developed sensor arrays that showed very low or negligible drift values [57,158,166,172,176,203,237,238], while other works quantified this effect (see Table 6 and Table A3) [186,198,213,233,307,336,362].

Drift due to environmental reasons require frequent recalibration of the arrays [85]. In fact, temperature is a common source of drift. This effect occurs in several technologies:

- In the case of capacitive technology, any capacitive sensor array is prone to drift in capacitance values due to temperature or humidity [79,167,171,173,269,370].
- In the case of piezoresistive technology, there are several studies considered state-of-the-art that analyze the effects of drift. Zhang et al. [69] studied temperature drift by placing the piezoresistive array inside a temperature chamber. Then it was compensated by a NN. In turn, Mirza et al. [64] pre-heated the sensor array and circuitry to prevent thermal drift. Sensor arrays using OAs (typical of resistive sensor arrays) may experience temperature drift [113,117,119]. This effect is difficult to calibrate and compensate for. Li et al. [79] avoided temperature drift by using a capacitive trans-impedance feedback amplifier (CTIA).
- In the case of Hall effect technology, Luca et al. [362] reported drift in a Hall effect sensor array, which was due to time and temperature.
- In the case of fiber-optic technology, temperature drift is a relevant problem, as fiber-optic arrays are often very sensitive [1,337]. Ren et al. [349] and Liu et al. [347] compensated for this effect by modeling it as an additional phase in the light signals.
- In the case of inductive technology, Yeh & Fang [210,211] reported drift in an inductive sensor array due to mechanical imperfections. Khatoon et al. [213] measured a drift value of less than 0.01% in 7 days.
- In VOC-sensitive piezoelectric resonators, drift can appear as a shift in the original frequency of the sensors after exposure to aggressive sorbates [307]

##### Crosstalk

Crosstalk is an effect that appears in sensor arrays due to the sharing of cables between several sensors. It becomes apparent when the measurement from a specific sensor is mixed with the measurements of adjacent sensors. This is a problem common to all sensor array technologies. Avoiding crosstalk is necessary in most sensor arrays, as it essentially represents inaccuracy. It can also be considered a source of noise, so its elimination increases the SNR [179].

Another type of crosstalk is mechanical. It occurs due to the physical structure of the sensor arrays. If the arrays are not well designed, it may appear that neighboring sensors are excited, when in fact they are not [208,271,285,285]. This limits multi-touch applications [264]. One way to evaluate this type of crosstalk is to press a sensor and observe its influence on adjacent elements of the array [95,241].

Crosstalk has been quantified in several ways: expressed as the electrical variable whose variation is detected [178] as a percentage of error [149,241,279,329], as light power in dB for fiber-optic arrays [342,347], or as an output value of the array [181]. Meanwhile, Yan et al. [315] quantified this effect as near-end crosstalk (NECT), which is the ratio of the voltage in the target sensor over the voltage in the surrounding sensors.

Great effort has been made to reduce crosstalk in all sensing technologies:

- Many studies on resistive/piezoresistive sensor arrays have dealt with crosstalk and proposed new techniques to compensate for it (referred to as “compensated” in Table 6 and Table A3) [8,50,51,59,64,72,75,93,96,97,99,104,105,106,107,109,110,111,112,113,114,115,117,118,119,120,121,122,123,124,125,126,127,128,129,130,131,132,133,134,135,136,138,139,140,141,310,370].
- Similar crosstalk compensation studies exist for capacitive arrays [160,166,178,179,200].
- In triboelectric sensor arrays, grounding/shielding techniques are used to avoid crosstalk [27,324]
- In fiber-optic sensor arrays, TDM or wave-division multiplexing are techniques used to reduce crosstalk [32,33,49,348,349]. They allow several signals to be transported within the same fiber beam. Meanwhile, Park et al. [353] reduced crosstalk between near scintillators placing reflectors between them.
- Transistor-based array do not usually exhibit crosstalk, as they conform active matrices. In this topology, only selected transistors conduct current, which completely eliminates crosstalk [94,116,221,222,370].

Several studies developed sensor arrays that had either no or negligible crosstalk [137,162,172,186,191,210,212,223,224,265,267,271,285,302], and others compensated for it through the physical design of the array (labeled as “eliminated by structure” in Table 6 and Table A3) [10,41,94,160,179,185,200,213,244,246,253,316]. One way to avoid crosstalk through structural modifications in all technologies is to have one line per sensor and never share cables between elements [244,246]. However, this configuration is inefficient in dense arrays.

##### Creep

Creep is associated with the viscoelastic behavior of some sensitive materials [65]. It has to do with the response time of the sensors (see the section entitled Response Time) [88]. It is only mentioned for resistive/piezoresistive arrays. However, it can be interpreted that sensor arrays that exhibit a delayed response suffer from creep. This effect is sometimes related to hysteresis and lack of repeatability due to its complex nature [65,69]. Angeli et al. [66] avoided creep using a thresholding technique.

##### Noise

This subsection groups the set of works that analyze the effect of noise on sensor array measurements. Noise is known to severely affect the resolution of systems [2,181]. This is an important issue in fiber-optic arrays, as they are high-frequency and high-precision systems [31,33,49,269,332,336,339,341,343,347,349,350]. Noise in oscillators can be provided as a shift in the base frequency [269,307].

A common approach found in existing studies was focused on reducing noise [98,151,187,198,257,300,309,338,341,347,360,364,367]. In this regard, studies on sensor arrays presented different noise cancellation strategies:

- In electromagnetically sensitive sensor arrays, adequate shielding can prevent high-frequency noise sources [147,184,189,216,286,315,322].
- Filtering is a common way to reduce noise [84,205,207,254,290,339,359,360].
- The noise of power supplies can be reduced by using coupling capacitors [205] or digital filters [254]. In the work of Wijaya et al. [98], this task was performed through a WT, which eliminated various types of noise.
- Calibration algorithms were also adopted to reduce noise [77,361].

In relation to noise metrics, a great variety was found in existing studies on sensor arrays:

- The influence of noise is usually quantified by the SNR [1,4,8,16,32,33,36,42,47,49,64,69,73,139,163,178,179,181,215,222,228,238,243,252,264,265,291,305,314,342,347]. The SNR in decibels (dB) can be calculated with Equation (31), where $P_{S}$ and $P_{N}$ are the power magnitude of the signal and noise, respectively, and $A_{S}$ and $A_{N}$ are the amplitude of the signal and noise expressed in any magnitude (voltage, pressure, etc.).
  (31) $$SNR=10\log\frac{P_{S}}{P_{N}}=20\log\frac{A_{S}}{A_{N}}$$
  Another way to calculate the SNR is with the mean value of the measurements of the sensor array and their standard deviation (Equation (32)) [8,64]. This is a percentage value. This metric assumes that the standard deviation comes from the noise:
  (32) $$SNR=20\log\frac{\bar{y}}{\sigma }$$
  Proper DAQ electronics design can improve SNR [103].
- Choi et al. [83] performed a logarithmic fit of the applied pressure on a piezoresistive sensor array versus SNR, obtaining an $R^{2}$ = 0.9861.
- The root-mean-square noise has also been used in existing studies [1,253].
- Noise can also be quantified in absolute magnitude values, as in the work of Ren et al. [350], Gagino et al. [99], and Santamato et al. [31].
- Magnetic noise $N_{M}\left(Hz^{1/2}\right)$ was obtained in the study of Qu et al. [309]. It can be calculated as $N_{M}=N/S$, where $N(V/Hz^{1/2})$ is the overall noise and S(V/T) is the sensitivity of the system.
- The EMN is analyzed in several works on Hall effect arrays [364,365].
- The allowable noise can also be quantified as a function of the number of ADC bits in digital systems [139,140]. If noise levels are above 1/2 LSB, the circuit readout can be severely affected.

Additive white Gaussian noise can be simulated in voltage signals, as shown in [273], following Equation (33).

(33) $$V=V_{measured}+E_{P}N_{noise}\sigma \left(V_{measured}\right),$$

where $E_{P}$ is the noise level (equivalent to $A_{N}$ in Equation (31)), $N_{noise}$ is the standard normal distribution with μ=0 and σ=1, and $\sigma (V_{measured})$ is the standard deviation of $V_{measured}$. Several studies added noise to their computational simulations [139,189,273]. Noise can be associated with the repeatability/uncertainty of measurements (see Section “*Repeatability*”) [140].

Finally, a group of studies evaluated the effects of manufacturing mismatch in sensor arrays, which in most cases caused “fixed-pattern-noise” (consistent, spatially repeating noise in sensor arrays caused by fixed variations between individual sensing elements):

- The resistive sensor array presented by Li et al. [79] reported fabrication inaccuracies, which were manifested as a mismatch in the nominal resistances of the RSA. In this regard, Angeli et al. [66] characterized the mismatches in a resistive array by conducting and appropriate experiment.
- Warnakulasuriya et al. [75] used a fixed resistor array model to prevent manufacturing defects and mismatching between the sensels in the array from interfering with the verification of the readout circuit.
- Yang et al. [318] identified impedance mismatch as the main problem for the commercialization of triboelectric sensor arrays.
- Fernandes et al. [178] presented a capacitive array with electrode cracking due to mismatch in the properties of the adhesion and elastomeric layers. In this sense, Nabovati et al. [197] identified the mismatch in the sensor electrodes as the main source of error in a capacitive array. Other authors [9,193] also reported inaccuracies in capacitive array measurements due to sensor mismatches.
- The work of Tabrizi et al. [205] is particularly interesting, as it is one of the few studies on sensor arrays that quantifies the effects of mismatch between several individual sensor elements. In fact, the standard deviation in the output current due to mismatch is quantified between 9.3 and 10 μA.
- Piron et al. [15] discussed time-to-amplitude converters in their review of diode arrays. They indicated that they were prone to noise and transistor mismatches.
- Su et al. [76] identified the mismatch in Young’s modulus as a major challenge in a transistor-based array.
- The study of Weichart et al. [176] focused on thermal expansion coefficient mismatches in a capacitive sensor array. They proposed minimization strategies based on coefficient matching. In this regard, the works of Verma et al. [84], and Kundu et al. [220] also dealt with compensation and minimization of mismatch. Finally, Faria et al. [215] stated that the effects of channel mismatches were minimal in their inductive array measurement system.

##### Flexibility

A flexible sensor array bends or stretches without loss of properties. Flexibility can be expressed quantitatively in several ways: bending or curvature radius [62,95,172,245,251,305,361] or bending angle [241,314]. Different sensing technologies have been used in the design of flexible systems: capacitive [37,38,92,144,145,149,150,156,157,162,169,170,174,182,185,195,218], transistor-based [58,160,221], piezoresistive [8,53,54,55,57,59,65,78,81,82,83,85,87,88,93,113,114,169], piezoelectric [63,93,243,244,245,255,259,271], triboelectric [40,163,315,326,328], diode-based [137], inductive [212], and fiber-optic-based [352]. Studies focusing on flexible sensor arrays typically test flexibility.

Flexibility testing is of particular interest for applications such as wearable devices and e-skins [60,65,94,95,142,154,158,175,176,188,246,250,260,282,286,288], imaging [227], sports [68], unmanned vehicles [3,82] and, in general, in two-dimensional applications where sensor arrays are subjected to stretching [61,62,66,76,159,162,171,178,204,241,247,252,366,367].

Flexibility is not only a performance metric for the sensing element, but also for other ancillary components: printed circuit boards (PCBs) [10,64,65,88,176,257,260,261,262,263,270,274,283,361] and electrodes [7,169,172,195].

#### 8.1.3. Performance Comparison

In this field, it is common to compare the performance of the proposed sensor array with other reference approaches to highlight technical contributions. There are several possible options: comparison with other state-of-the-art studies or methods [1,5,6,7,9,32,36,41,46,47,48,49,55,57,58,62,63,66,68,70,73,76,78,79,83,84,85,89,91,94,95,98,99,100,101,107,108,109,131,137,138,140,141,144,145,147,149,151,155,156,158,163,165,167,171,172,181,184,185,189,191,196,199,204,205,207,208,209,216,218,220,223,228,229,230,232,233,235,239,240,242,247,248,250,251,253,255,257,267,268,269,271,278,279,280,281,282,286,289,291,292,293,297,305,309,310,314,316,325,333,342,346,347,355,359,360], comparison with commercially available alternatives [1,43,55,80,90,97,101,168,179,180,197,216,221,242,244,256,271,272,284,289,307,312,323,336,352,357,362], comparison with *gold-standard* reference devices to test a less expensive option [42,80,97,168,225,270,274,284,285,307,312,345,352,358,366], or comparison with mimicked biological systems [43,52,85,158,222,246]. The Bland–Altmann plot was a common metric when comparing the sensor array with a reference system [225,284,366].

Another group of studies focused on other aspects, such as processing methods or manufacturing processes. On the one hand, several works compared new processing techniques to increase readout accuracy with state-of-the-art methods [33,97,104,106,110,111,113,115,116,117,119,123,126,129,133,135,139,160,176,178,179,192]. On the other hand, the study of Ha et al. [217] presented a transistor manufacturing system, which was compared with the standard CMOS fabrication. In [175], it was observed that micro-array dielectric layers greatly increase the sensitivity of the capacitive pressure sensors.

#### 8.1.4. Visual Evaluation

These articles rely extensively on graphical information to evaluate the performance of their sensor arrays [51,303,356]. Some examples of these graphical tests are input vs. output signals [45,89,157,165,195,202,203,206,207,210,211,219,226,227,249,259,262,263,264,270,274,278,279,281,293,299,312,316,317,321,335,340,343,348], error plots [108,109], heat maps of the distribution of the variable of interest [81,84,215,260,280,289,296,301], or physical design parameters [58,165,200,311,312,322]. Most of the works show graphic evidence of their results, but only those that are primarily based on it are included in this group.

### 8.2. Brief Conclusion of Sensor Array Metrics

**Figure 62:**
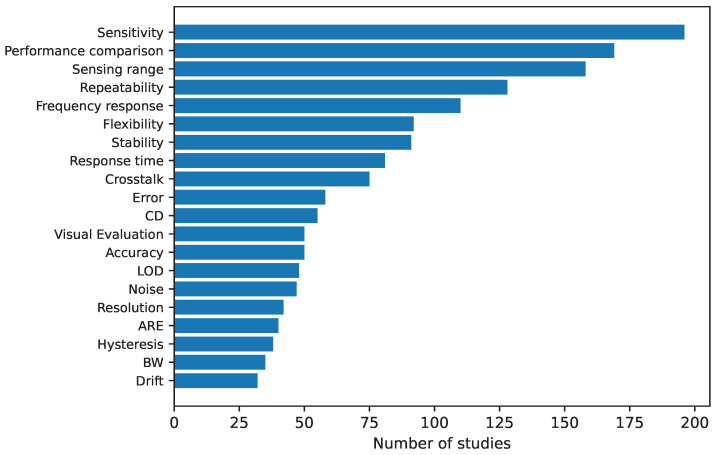
Number of sensor array studies using the different performance parameters.

Figure 62 represents the number of studies that adopt the most frequent validation metrics. Sensitivity, performance comparison, and sensing range are widely used, appearing in 54.3%, 46.8%, and 43.8% of existing studies, respectively. Among the dynamic parameters, the frequency response is the most common, as frequency information is useful for many sensor array applications. The authors in this field also pay attention to the repeatability, flexibility, stability, and response time of sensor arrays, while other physical metrics such as BW, the effect of noise, or hysteresis are not studied so often.

Finally, Table 8 shows some aspects related to sensor array performance metrics to be considered by researchers in this field.

## 9. Discussion and Conclusions

This paper presents a comprehensive systematic review on sensor arrays. For the first time in this field, all technologies have been considered and compared, focusing on all aspects related to sensor array design. Thus, this paper fills an existing gap in the field of sensor arrays. This has led to the identification of the aspects that could be improved.

This review has several limitations. Although a large number of studies (361) have been analyzed in detail, only the Web of Science repository has been taken into account for the searches. This is a prestigious repository that compiles relevant papers in this field, although it may not contain all those published. The inclusion of additional papers could have an impact on the synthesis of the results or on the conclusions drawn. In addition, two of the review authors have screened each study and extracted its relevant information. Although rigorous work has been carried out, the participation of additional researchers in this task could have helped to complement the analysis performed. Finally, the different categories of the review were organized according to the aspects of sensor arrays that may be of interest to researchers in this field. However, the information could also have been structured differently depending on the intended audience.

From the comprehensive analysis conducted, different research challenges were identified. In relation to sensing technologies, resistive, piezoelectric, and capacitive technologies are widely explored in array configurations. However, configuring arrays with inductive or bioimpedance sensors is much more complex. The array configuration remains a challenge for these technologies.

With regard to applications, this review has shown that many sensing technologies can be used for the same application. In other words, there is not a high correlation between application and sensing technology. It is also worth noting the large number of fields in which sensor arrays have been applied. This gives an idea of the opportunity that this technology represents. Due to its great versatility, researchers could expand the use of the array configuration to unexplored applications or future sensing requirements.

With respect to the validation of sensor arrays, most researchers performed simulations or laboratory experiments. However, integrating sensor arrays into usable prototypes remains a challenge. Only a few studies present usable real-world systems with sensor arrays that can be used long-term in unattended environments. Instead, controlled laboratory experiments or computer simulations prevail. Future research should focus on conducting long-term unsupervised evaluations with fully functional prototypes incorporating sensor arrays.

In relation to the characteristics of sensor arrays, most studies provide information on cost and power consumption. These two aspects remain a major issue, leading to the low number of portable sensor array devices. The high number of sensors involved and the complexity of the associated data acquisition systems increase both the cost and power consumption of the prototypes. Therefore, future research should focus on addressing this challenge.

In terms of the software tools employed in sensor array systems, most studies use proprietary solutions, while the adoption of open source tools remains limited. As a result, only a few studies provided the raw data collected during the experiments, limiting the potential for reuse by other interested researchers. Therefore, a challenge for future research is to improve the availability of sensor array data and processing code. This would facilitate performance comparison among studies and repeatability.

In summary, sensor array design is challenging, given the large number of aspects to be considered. The results of this comprehensive review can help researchers identify the most effective solution for each aspect involved in developing new sensor arrays.

## Figures and Tables

**Table 1:** Analysis of sensing technology, sensor dimensions, electrode material, and sensing material. This table includes only the most recent studies. The analysis of the remaining studies is included in in Appendix A (Table A1).

Study	Technology	Sensor Dimensions	Electrode	Sensing Material
[30]	Triboelectric	3 × 3 (3 × 3 cm)	Ag, Cu, Au	9 different materials: PDMS, Ecoflex, Rubber, PVDF, PTFE, PI, PCL, PA66, PU
[31]	Fiber Optic	2 + 1 sensors (2 for strain + 1 for temperature)	FBG	FBG
[32]	Fiber Optic	4 sensors	Fiber Optic	Fiber Optic
[33]	Fiber Optic	4 sensors (10 m long)	Fiber Optic	Fiber Optic
[34]	Transistor	8 sensors	Al	IGZO
[35]	Capacitive	6 × 6	Cu	Dragon Skin^TM^
[36]	Capacitive	4 × 3 (150 mm × 150 mm, 29.8 × 40 × 7 mm electrode area)	Cu	Object to detect
[37]	Capacitive	3 × 3 (electrodes of 2 mm radius)	Ag	Ionic gel
[38]	Capacitive	4 × 8 (1 cm × 2 cm)	Graphene	PDMS
[39]	Triboelectric	32 sensors inside a shoe insole (1 cm × 1 cm per sensor)	CNT	Nylon, PVDF
[40]	Triboelectric	5 × 1 (each sensor in one finger)	Medical hydrogel	Silicone hydrogel, FEP
[41]	Triboelectric	4 sensors (each sensor 40 mm height, 20 mm diameter)	Conductive ink	FEP, PTFE, PLA
[42]	Triboelectric	2 × 2 (1 cm × 1 cm)	Al, Cu	PDMS, Nylon
[43]	Triboelectric	2 × 2 (2 × 2 ${\mathrm{cm}}^{2}$ per sensor)	Al	Silicone, Ferrofluid
[44]	Triboelectric	2 × 2 (120 × 120 mm)	Cu	FEP, EVA, PMMA
[45]	Triboelectric	3 × 3 (95 × 70 mm, 20 × 12 mm electrode)	Cu	PDMS, Water
[46]	Piezoresistive	4 × 4	Cu	Methylcellulose-chitosan Mxene
[47]	Piezoresistive	4 × 4	Ag, PET	MWCNT, PDMS
[48]	Piezoresistive	8 × 8 (5 × 5 ${\mathrm{mm}}^{2}$)	-	Silicon PDMS
[49]	Fiber Optic	4 sensors	Fiber Optic	Fiber Optic

**Table 2:** Summary of advantages and disadvantages of each sensing technology.

	Advantages	Disadvantages
Resistive and piezoresistive sensor arrays	- Arrays can be made of soft, formable materials, suitable for irregular or moving surfaces. - Low cost arrays. - Relatively easy to manufacture. - They have good scalability and are suitable for applications where large areas need to be covered.	- They usually present nonlinearity and hysteresis problems. - Arrays made of low-cost piezoresistive materials often drift with time, temperature or humidity. - They require advanced acquisition circuits to avoid crosstalk between adjacent cells in the array.
Capacitive sensor arrays	- Fast response time of the entire array. - They provide stable and repeatable measurements over long periods. - They are usually low power. - They show low hysteresis and crosstalk	- Calibration and fine-tuning of the array is required. - They require advanced acquisition circuitry to detect small capacitance changes. - In large arrays, there may be interference between adjacent sensors. - Capacitive sensors often show temperature drift.
Inductive sensor arrays	- Fast response time of the entire array. - Contactless. - They have a long service life and are resistant to harsh environments.	- Emerging technology. - Sensitive to electromagnetic interference. - In large arrays, there may be interference between sensing elements.
Diode sensor arrays	- Fast response time of the entire array. - These arrays can be easily incorporated into integrated circuits and miniaturized systems. - Low power consumption.	- They are usually temperature-dependent. - Complex acquisition circuitry to read array measurements. - Array output is not always linear.
Transistor sensor arrays	- Arrays can be made of formable materials, suitable for irregular or moving surfaces. - Measurements can be amplified directly in the sensor array as it is composed of active devices. - High spatial resolution arrays. - Low power consumption when not in active mode.	- Their manufacture process is among the most complex. - Measurements are prone to electrical or environmental interference. - They can be expensive.
Piezoelectric sensor arrays	- Fast response time of the entire array. - Low power consumption due to the potential for self-harvesting. - The arrays can be integrated into curved or irregular surfaces.	- The sensibility of the array depends on the orientation of its sensors. - Complex acquisition circuitry to read array measurements. - Measurements prone to noise interference. - They do not provide an output in the absence of stimuli.
Triboelectric sensor arrays	- Low power consumption due to the potential for self-harvesting. - Arrays can be made of soft, formable materials, suitable for irregular or moving surfaces. - Affordable cost.	- As arrays rely on contact or friction, they may degrade with repeated use. - Difficult to calibrate and array response can vary greatly based on angle or direction of the stimulus. - Measurements prone to noise interference. - They do not provide an output in the absence of stimuli.
fiber-optic sensor arrays	- High measurement sensitivity and accuracy. - Arrays with high immunity to electromagnetic interference. - Arrays are small in size and light in weight.	- They can be expensive. - They require specific interrogation equipment that limits scalability. - For large arrays, installation can be very complex.
Hall effect sensor arrays	- Fast response time of the entire array. - Robust arrays. - They perform will in harsh environments and have a long service life. - Low power consumption. - Small and easy-to-integrate arrays.	- Measurements prone to magnetic interference. - Limited applications, as they require magnetic fields. - Complex acquisition circuitry to read array measurements.
Bioimpedance sensor arrays	- They collect data from multiple zones, which favors the robustness of the measurements. - Arrays can be made of soft, formable materials, suitable for irregular or moving surfaces.	- Complex acquisition circuitry to read array measurements. - Significant power consumption. - They can be expensive.

**Table 3:** Analysis of the type of array (wearable or environmental), application, measured variable, and validation experiment. This table includes only the most recent studies. The analysis of the remaining studies is included in in Appendix B (Table A2).

Study	Wearable or Environmental	Application	Measured Variable	Validation Experiments
[30]	Environmental	HMI	Pressure	Motor Subjects interacting externally with the device
[31]	Environmental	SHM	Strain Temperature	Computational simulation Lab experiment Motor
[32]	Environmental	Blood monitoring Improve readout accuracy	Light	Lab experiment
[33]	Environmental	Improve readout accuracy	Light	Vibration machine Computational simulation
[34]	Environmental	Organic compounds	Concentration	Chemical testing
[35]	Environmental	Electronic skin	Force	Motor Robot
[36]	Environmental	Imaging SHM	Presence	Computational simulation Lab experiment
[37]	Wearable	Heart monitoring Airflow applications Electronic skin	Pressure	Subjects wearing the device Wind machine
[38]	Environmental	Surgery	Pressure	Robot Force gauge Motor Customized object or stamp Computational simulation
[39]	Wearable	Plantar pressure Walking assessment	Pressure	Subjects wearing the device Motor
[40]	Wearable	Gesture recognition	Pressure	Subjects wearing the device Motor Force gauge
[41]	Environmental	Marine applications	Pressure	Computational simulation Motor
[42]	Wearable	Speech detection Swallowing detection	Pressure	Subjects wearing the device Motor
[43]	Environmental	Airflow applications	Pressure	Motor Wind machine Computational simulation
[44]	Environmental	HMI	Pressure	Motor Subjects interacting externally with the device
[45]	Wearable	Walking assessment	Force	Subjects wearing the device Computational simulations
[46]	Wearable	Heart monitoring HMI	Pressure	Motor Customized object or stamp Subjects wearing the device
[47]	Wearable	Heart monitoring Airflow applications Electronic skin HMI Speech detection	Pressure	Subjects wearing the device Subjects interacting externally with the device Customized object or stamp Motor
[48]	Environmental	Force	Force	Computational simulation Force gauge
[49]	Environmental	Improve readout accuracy	Vibration	Computational simulation Vibration machine

**Table 4:** For each type of application (columns), the percentage of studies belonging to each sensing technology (rows) is included (%). The sum of the values in each column gives a value of 100%. Values exceeding 20% are highlighted in bold.

	Interaction	Human Health Monitoring and Biometric	Measurement of Physical Magnitudes	Chemical, Biological and Physical	Energy Generation	Security	Marine and Aerospace	Improve Readout Accurac	Imaging
Resistive, piezoresistive	**28**	**26**	18	**25**	**21**	0	**26**	**68**	0
Capacitive	**34**	**21**	**26**	**25**	**21**	18	11	18	**44**
Inductive	4	0	3	0	0	3	0	3	6
Diode	2	1	1	3	0	0	0	0	13
Transistor	3	1	5	**27**	0	0	0	1	**25**
Triboelectric	13	14	5	2	**36**	0	**21**	0	0
Fiber optic	1	4	14	5	0	12	16	9	13
Hall effect	2	4	2	0	0	6	0	0	0
Piezoelectric	13	**27**	**26**	12	**21**	**61**	**26**	1	0
Bioimpedance	0	2	0	0	0	0	0	0	0

**Table 5:** Summary of advantages and disadvantages of each validation experiment.

	Advantages	Disadvantages
Computational simulation	- It is easy to simulate different testing scenarios. - Testing is often cheaper, as it does not require physical equipment. - Aspects of sensor arrays that are difficult to test in real-world environment can be tested using computational simulation.	- Simulation results may not always represent the real-world behavior of sensor arrays. - Some sensor array tests may require specialized simulation software.
Tests using a mechanical force element	- As it is a common testing method, it is easy to compare performance with other studies. - It is useful for characterizing sensor arrays. - It is widespread in this field.	- Test results may not represent the real-world performance of sensor arrays. - In most cases, they require specialized equipment and expensive machines.
Chemical testing	- It is useful to test some internal properties of sensor arrays. - Tests are performed in controlled environments and under controlled conditions. - They improve the reproducibility of the test.	- It is useful for a limited number of applications. - Test results may not represent the real-world performance of sensor arrays. - Tests require specialized equipment, and the results are sometimes difficult to interpret.
Validation with human subjects	- Sensor arrays are tested directly with target users. - Aspects related to the usability of the sensor arrays and their behavior in the real world can be assessed. - Sensor arrays are tested in a real-world environment and under real-world conditions.	- They require prior approval from an ethics committee, which can slow down the testing process. - A large number of volunteers are needed to obtain significant results. - Volunteers participating in the tests must be representative of end users, which is not always possible.
Lab experiment	- Arrays are tested in a controlled environment. - Researchers have a high degree of control over the tests. - The tests can be adapted to the specific characteristics of each particular sensor array.	- Difficult to replicate by other researchers, as tests are often customized by researchers and based on specific equipment. - Difficult to compare with other sensor array studies, as tests are highly dependent on the laboratory.

**Table 6:** Analysis of the software used for processing, the sensor array characteristics, and the metrics used to evaluate performance. This table includes only the most recent studies. The analysis of the remaining studies is included in Appendix C (Table A3). Parameters labeled as *visually* present the corresponding result only graphically instead of numeric values.

Study	Software for Analysis	Characteristics	Metrics
[30]	MatLab	Sampling frequency (1 kHz)	Accuracy (99%), Stability (120,000), Response time (8 ms), Repeatability (visually), Crosstalk (eliminated by structure)
[31]	MatLab, FEA	Effects of ECs (=temperature sensitivity)	Frequency response, Repeatability (2.7–6.4%), Noise (1.58 μϵ, 2.7–6.4%)), Relative error (individual sensors are in range 6–14%), Sensitivity (strain = [−1.58, 1.68] μϵ/kN, temperature = (1.3–1.5) × $10^{-5}$ °$C^{-1}$). All values are provided for FBGs 1 to 4.
[32]	-	Spatial resolution (10.7 µm), Sampling frequency (166 kHz)	Sensitivity (average = 1 mPa/${\mathrm{Hz}}^{1/2}$, visually), BW (16 MHz), SNR (30.9–32.2 dB), Frequency response, Performance comparison
[33]	-	ADC bits (14), Sampling frequency (100 MHz)	Noise (THD = 0.12%), Frequency response, SNR (20 dB), RMSE (0.07), Performance comparison, Crosstalk (compensated)
[34]	-	Effects of ECs (“no notable alteration when samples were stored in different atmospheric conditions’’)	Stability (50,000 cycles), Correlation coefficient (53.2–99.7%)
[35]	Python, C	Power consumption (0.05 mAh), ADC bits (16)	Sensing range (0.1–3 N), CD (0.9996), RMSE (0.0446)
[36]	COMSOL	-	MSE (<2 × $10^{-3}$), RMSE (<0.2), Correlation coefficient (>0.65), SNR (>62 dB), Performance comparison
[37]	-	-	Sensitivity (5.55 ${\mathrm{kPa}}^{-1}$), Stability (3000 cycles), Flexibility (tensile deformation up to 100%), Sensing range (2.5–16 kPa), LOD (30 Pa), Response time (53 ms), Absolute error (0.2 Pa), Frequency response
[38]	FEA	Spatial resolution (4 ${\mathrm{mm}}^{2}$)	Flexibility, Response time (160 ms), Sensitivity (1.2 ${\mathrm{kPa}}^{-1}$), Repeatability
[39]	-	Power consumption (1.2 µA generated, 42.5 V open circuit)	Sensitivity (19.4–45.1 mV/kPa), Sensing range (40–400 kPa), Accuracy (94.32%), Frequency response, Stability (40,000 cycles), CD (0.95–0.98), Response time (48 ms)
[40]	Python	Cost (“cost effective” materials, 1.5$ the whole module), Power consumption (23 V, 11 nC, 180 nA, generated)	Flexibility, Accuracy (98.5%), Frequency response, Stability (14,000 s), Response time (<30 ms), Repeatability (visually)
[41]	ANSYS, COMSOL	Sampling frequency (120 fps)	Response time (19 ms), Sensitivity (0.2V/${\mathrm{ms}}^{-1}$), Accuracy (81.2%), RMSE (0.02), Frequency response, Performance comparison
[42]	LabView	Power consumption (generated max. power density = 0.48 mW/$m^{2}$), Effects of ECs	Performance comparison, Accuracy (97.06% for speech, 98.04% for swallowing), Sensing range (1–10 kPa), Frequency response, Sensitivity (0.109 V/kPa), Stability (10,000 cycles), SNR (12.42–25.14 dB)
[43]	LabView, MatLab, COMSOL	Effects of ECs (environmental magnetic field = 50 mT), Cost (“cost-effective manufacturing approach”)	Sensitivity (38.84 ${\mathrm{kPa}}^{-1}$), LOD (0.76 Pa), Performance comparison, Repeatability, CD (0.995), Response time (26–29 ms), Stability (6000 cycles)
[44]	Phyphox	Effects of ECs, Power consumption (2.5 to 7 µA generated, 150 V), Cost (“low cost triboelectric sensors’’)	Frequency response, Stability (25,000 cycles), Accuracy (76–100%)
[45]	COMSOL	Power consumption (40.5 mW generated)	Accuracy (>96%), Frequency response, Visual evaluation
[46]	-	-	Sensitivity (2.9 ${\mathrm{kPa}}^{-1}$), Stability (8000 cycles), Response time (119 ms), Performance comparison
[47]	-	Cost (“cost effective”)	Sensitivity (1.74 ${\mathrm{kPa}}^{-1}$), Response time (<100 ms), LOD (5.8 Pa), Hysteresis (3.46%), SNR (37 dB), Performance comparison
[48]	FEA	Cost (low cost due to “MEMS process”)	Sensitivity (0.025 V/N), Performance comparison, Sensing range (0–0.72 N), CD (0.9998)
[49]	FEA	Sampling frequency (500 MS/s), Cost (“increases the system cost and complexity”)	SNR (30 dB ref ${\mathrm{rad}}^{2}$/Hz), BW (500 kHz), Performance comparison, Sensitivity (17.89 dB ref rad/V), Frequency response, Crosstalk (compensated), Noise (std = 0.2837 rad, power spectral density = −98.54 dB)

**Table 7:** Summary of some remarkable aspects about sensor array characteristics.

Large sensor arrays	It is common to find arrays from 2 × 2 and 4 × 4 dimensions. However, it is difficult to find larger arrays, especially for some sensing technologies such as Hall effect arrays. This may be due to limitations of the data acquisition circuitry. Future studies should explore large sensor arrays.
Cost not provided	Most studies do not provide quantitative details on costs. In general, the cost of sensor arrays is not a topic of interest to researchers, as studies focus more on laboratory prototypes than on commercial devices. Future studies should pay more attention to this factor.
Spatial resolution in millimeter scale	The spatial resolution provided in most sensor array studies is at the millimeter scale. However, spatial resolution is highly dependent on the specific application. Studies that present less demanding applications often omit the resolution information in the papers.
High sampling frequency	The sampling frequency is usually high in sensor array characterization, as it is common to use high-performance DAQs. However, in sensor arrays used in real-life applications, low-cost DAQs are also employed. The effects of these differences between characterization and real-life use should be studied.
No homogeneity of power values	Most studies do not provide information on power consumption. This may be because the systems are not considered to be portable. Also, there is no homogeneity in the way power details are provided (some studies include only the array consumption, others include the acquisition system, others consider the idle mode, etc.). More rigorous studies on the power consumption of sensor arrays are needed.
Effects of environmental conditions not deeply studied	Most studies do not provide information on the effects of environmental conditions on sensor arrays. The most quantified effect is temperature. However, other effects such as humidity or pressure are hardly studied. It is also common to provide a qualitative assessment of the effect of environmental conditions. Quantitative approaches are generally lacking.

**Table 8:** Summary of some aspects about sensor array performance metrics.

Difficult to compare performance	There is a wide variety of metrics and measurement conditions. This makes it difficult to compare studies fairly. Future research should improve the comparison of results.
Non-idealities evaluated in a qualitative way	Some studies that take into account sensor array non-idealities (hysteresis, creep, drift, crosstalk, etc.) evaluate them qualitatively, but do not provide a numerical performance metric. More specific evaluations of these non-idealities and a common way of showing their influence them are needed.
Accuracy and error in lab experiments	In general, accuracy is high and error values are low. However, most of the time these values are obtained in laboratory experiments. Rarely is the performance of the sensor arrays evaluated in a real-use environment.
Repeatability, stability and response time generally evaluated	Most studies include quantitative values for repeatability, stability and response time. This demonstrates that researchers are performing integral characterizations of sensor arrays.

## Abbreviations

The following abbreviations are used in this manuscript:

ADC	Analogue-to-Digital Converter
AE	Absolute Error
AFCRS	Array of fiber-optic Cerenkov Radiation Sensor
AI	Artificial Intelligence
ARE	Absolute Relative Error
BAW	Bulk Acoustic Wave
BW	Bandwidth
CACT	Co-planar array capacitive imaging technique
CERN	European Organization for Nuclear Research
CFET	Cantilever FET
CNN	Convolutional Neural Network
CNT	Carbon nanotube
CTIA	Capacitive trans-impedance feedback amplifier
CV	Coefficient of variation
DAQ	Data Acquisition Card
DG-TFT	Dual-Gate TFT
ECs	Environmental conditions
ECT	Electrical capacitance tomography
EISCAP	Electrolyte-insulator-semiconductor capacitors
EMN	Equivalent magnetic noise
ENOB	Effective number of bits
E-Nose	Electronic nose
FBG	Fiber Bragg Grating
FEA	Finite-Element Analysis
FEP	Fluorinated Ethylene Propylene
FET	Field-Effect Transistor
FMPE	Ferromagnetic-piezoelectric
FS	Full scale
FSO	Full scale output
FSR	Force-sensitive resistor
HEMT	High-Mobility Electron Transistor
HMI	Human-Machine Interface
ICC	Intra-Class Correlation Coefficient
IGZO	Indium Gallium Zinc oxide
IMU	Inertial Measurement Unit
IoT	Internet of Things
ISFET	Ion-Sensitive FET
kNN	k-Nearest Neighbor
LMA	Levenberg-Marquardt Algorithm
LOD	Limit of detection
LSB	Least significant bit
LWR	Lamb wave resonator
MAE	Mean Absolute Error
MARE	Mean Absolute Relative Error
MEMS	Micro electro-mechanical system
MG-FET	Moving-Gate FET
MIP	Molecularly imprinted polymer
ML	Machine learning
MRE	Mean Relative Error
MSE	Mean Squared Error
MUSIC	Multiple Signal Classification
MWCNT	Multi-walled CNT
NECT	Near end crosstalk
NEP	Noise equivalent pressure
NI	National Instruments
NIST	National Institute of Standards and Technology of the United States
NRMSE	Normalized root mean squared error
NN	Neural Network
NW	Nanowires
OA	Operational Amplifier
OSA	Obstructive Sleep Apnea
PAAMPSA	poly(2-acrylamido-2-methyl-1-propanesulfonic acid
PAni	polyaniline
PBU	polybutadiene-urethane
PCA	Principal Component Analysis
PCB	Printed Circuit Board
PCL	Polycaprolactone
PEDOT:PSS	poly (3,4-ethylenedioxythiophene): polystyrene sulfonate (PSS)
PEGDA	Poly(ethylene glycol) diacrylate
PEO	polyethylene oxide
PET	polyethylene terephthalate
PDMS	polydimethylsiloxane
PMMA	Polymethyl methacrylate
PMN–PT	Lead magnesium niobate–lead titanate
pMUT	piezoelectric Micromachined Ultrasonic Transducer
PQR	Piezoelectric Quartz Resonator
PSM	Pressure-sensitive mat
PTFE	Polytetrafluoroethylene
PVA	Polyvinyl alcohol
PVDF	polyvinylidene fluoride
PWV	Pulse Wave Velocity
RE	Relative Error
RMSE	Root Mean Squared Error
SAW	Surface Acoustic Wave
SHM	Structural Health Monitoring
SNR	Signal-to-Noise-Ratio
STD	Standard deviation
TDM	Time-division multiplexing
TEG	Triboelectric Generator
TENG	Triboelectric Nano-Generator
TFT	Thin-Film Transistor
UV	Ultraviolet
UXO	Unexploded Ordnance
VOC	Volatile Organic Compound
VOL	Volatile Organic Liquids

## Appendix A

**Table A1:** Analysis of sensing technology, sensor dimensions, electrode material, and sensing material. This table includes the studies not analyzed in Table 1.

Study	Technology	Sensor Dimensions	Electrode	Sensing Material
[183]	Capacitive	5 × 1 (25 × 124 ${\mathrm{mm}}^{2}$, 24 × 24 ${\mathrm{mm}}^{2}$ per sensor)	Cu	Silicone composite
[337]	Fiber Optic	10 × 10 (10 cm of separation)	Commercial sensor	Commercial sensor
[365]	Hall effect	3 × 3 (7 × 10 ${\mathrm{cm}}^{2}$)	-	${\mathrm{Al}}_{2}$$O_{3}$, Ta, Py
[251]	Piezoelectric	3 × 3 (10 × 12 mm per sensor)	Ag	P(VDF-TrFE), ${\mathrm{Fe}}_{3}$$O_{4}$
[363]	Hall effect	6 sensors (elliptical array)	Commercial sensor	Commercial sensor
[103]	Resistive	16 × 16	Custom magnetic sensor	Custom magnetic sensor
[346]	Fiber Optic Piezoelectric	16 elements	Fiber Optic	Fiber Optic
[331]	Triboelectric	7 × 1 (7 piano keys, 2 ${\mathrm{cm}}^{2}$ per sensor)	Al	rGO, Nylon, Ecoflex, Mo$S_{2}$
[102]	Resistive Triboelectric (heterogeneous)	2 × 2 (6 × 3 ${\mathrm{cm}}^{2}$)	Ag	Mxene, Metal oxide
[330]	Triboelectric	3 × 2 (area of a bed)	Cu	PDMS, PVDF
[248]	Piezoelectric	4 × 4 (29 × 29 mm, 5 mm × 5 mm per sensor)	AgNW	P(VDF-TrFE), ZnO
[329]	Triboelectric	15 sensors (3 per finger)	Au, Cu	Vertical graphene, FEP
[93]	Capacitive Resistive	5 × 5	Copper foil, Graphite paint film	Air
[328]	Triboelectric	5 × 3 (20 × 20 ${\mathrm{mm}}^{2}$)	Galinstan	PVA
[242]	Piezoelectric	2 × 5 (2 × 2 mm each sensor)	Ti, Pt	V-doped ZnO
[301]	Piezoelectric	7 × 1 (real-life), 8 nodes (simulation, 10 mm spaced)	PZT	PZT
[107]	Resistive	10 × 10 (simulated), 4 × 4 (real)	Commercial resistor	Commercial resistor
[163]	Triboelectric Capacitive (heterogeneous)	4 × 4 (7 × 7 mm per sensor)	EGaIn	Liquid metal, MXene, Silicone
[255]	Piezoelectric	5 × 1 (8 × 6 cm)	Printed Ag	BaTi$O_{3}$
[319]	Triboelectric	20 × 20 (32 × 32 ${\mathrm{mm}}^{2}$)	Galinstan + Nano iron powders	Nano silica, PTFE, Silicone
[285]	Piezoelectric	3 × 3 (2 mm diameter each sensor)	Cr, Au	PZT
[87]	Piezoresistive	3 × 3 (10 mm × 14 mm)	Cu	Mxene, ZnONW
[149]	Capacitive	16 × 16 (3 cm × 3 cm)	Cu	MWCNT, PDMS
[86]	Piezoresistive	2 × 3 (1 × 1.5 cm, 100 µm electrode size)	ITO	PEO:PAni
[280]	Piezoelectric	24 sensors (circular shape around a bolt)	-	PZT
[85]	Piezoresistive	32 × 32 (0.6 mm pixel spacing)	Ag	Carbon, Graphene
[281]	Piezoelectric	9 sensors (20 × 20 mm each sensor)	-	PMN-PT
[84]	Piezoresistive	5 × 5	CMOS fabrication	CMOS fabrication
[83]	Piezoresistive	10 × 10	AgNW-PBU	PBU-MXene
[82]	Piezoresistive	8 × 8 (370 × 470 mm)	Cu	CNT commercial sensor
[81]	Piezoresistive	8 × 1 (8 conductive strips, 180 cm × 90 cm)	Cu	Velostat^TM^
[80]	Piezoresistive	10 sensors embedded in smart clothing	Cu	PU-MWCNT
[141]	Resistive	16 × 16	Cu	Velostat^TM^, Commercial PSM
[108]	Resistive	40 × 240 (35 × 200 cm)	Cu	Caplink
[101]	Resistive (heterogeneous)	2 × 1 (one sensor detects Zn(II) ion, one sensor detects Cu (II) ion)	Au	Device 1: ${\mathrm{Ni}}_{2}$$O_{3}$ Device 2: ${\mathrm{Cu}}_{0.05}$${\mathrm{Ni}}_{1.95}$$O_{3}$
[79]	Resistive	10 × 10	Cu	-
[138]	Resistive	From 4 × 4 to 32 × 32 (simulated), 8 × 8 (real)	Commercial resistor	Commercial resistor
[340]	Fiber Optic	4 sensors (1 m × 1 m × 1 m)	Fiber Optic	Fiber Optic
[189]	Capacitive	8 electrodes (wrapped around a metallic pipe)	Cu	Object to detect
[193]	Capacitive	2 × 8 × 16 (35 µm × 30 µm)	CMOS fabrication	CMOS fabrication
[186]	Capacitive	6 × 6 (94 × 94 ${\mathrm{mm}}^{2}$, 14 × 14 ${\mathrm{mm}}^{2}$ each taxel, 5 electrodes per sensor)	Cu	Ecoflex gel, conductive fabric
[344]	Fiber Optic	2 × 3	Fabry-Perot	Fabry-Perot
[342]	Fiber Optic	26 FBGs (20 m long)	FBG	FBG
[238]	Transistor	2 × 2 (4 mm × 4 mm)	Ti, Au	CNT, Au, Cu, Ti
[224]	Transistor Triboelectric	5 × 5 (4 cm × 4 cm)	Mo Ag	IGZO
[237]	Transistor	3 electrodes (3 different antibiotics)	MIP	AuNPs-loaded metal-organic framework
[236]	Transistor	5 × 5	Au	PCBM-MAPb$I_{3}$
[52]	Piezoresistive Diode (heterogeneous)	16 × 16, 8 × 8 (16 × 16 mm)	Mo, ITO	IGZO, MWCNT
[240]	Transistor	16 sensors (7 × 7 ${\mathrm{mm}}^{2}$)	Pt, Chitosan, Ag AgCl	PEDOT:PSS
[215]	Inductive	40 sensors (100 × 100 mm, 1 × 0.5 × 0.5 mm per sensor)	Commercial sensor	Commercial sensor
[214]	Inductive	4 sensors (different shapes, wrapped around the elbow)	Cu	Conductive yarn
[212]	Inductive	4 × 4 (cilinders of 5 mm diameter, 6 mm height)	Cu	Ecoflex + Magnetic filler
[194]	Capacitive	6 sensors (concave electrodes around a pipeline)	Cu	Object to detect
[336]	Fiber Optic	8 sensors	Fiber Optic	Fiber Optic
[146]	Capacitive	3 × 3 (10 × 10 mm per sensor)	Printed Ag	PI, PDMS
[245]	Piezoelectric	3 × 3 (3.14 ${\mathrm{mm}}^{2}$ each pixel)	Cr, Au	PZT, PDMS
[243]	Piezoelectric	3 × 1 (2 cm long each sensor)	Cr, Au	PDMS, AlN, AlGaN
[77]	Piezoresistive	30 × 11	-	Velostat^TM^
[100]	Resistive	14 × 1	Commercial sensor	Commercial sensor
[190]	Capacitive	6 (area of the electrode 70 mm × 8 m)	Cu	Object to detect
[358]	Hall effect	4 × 4 (4.5 mm spacing)	Commercial sensor	Commercial sensor
[244]	Piezoelectric	255 sensors (60 × 90 cm, 7 × 32 mm per sensor)	Printed Ag	P(VDF-TrFE), PEDOT:PSS
[144]	Capacitive	6 × 28 (0.36 × 1.82 $m^{2}$, in 7 tiles)	Cu	EVA
[147]	Capacitive	8 × 8 (2.5 × 2.5 mm each taxel)	Cu	Air, PI
[185]	Capacitive	137 electrodes distributed in the palm of a hand	Cu	PU, BaTi$O_{3}$
[184]	Capacitive	4 × 8	-	Silicone
[182]	Capacitive	16 × 16 (70 mm × 70 mm)	Ag ink	Object to detect
[235]	Transistor	2 × 2	Ti, Au	CNT, Si
[229]	Transistor	12 × 12	Au, Ti	Si, Au, Ti
[353]	Fiber Optic	20 × 1	Fiber Optic	Fiber Optic
[169]	Capacitive	4 × 3	AgNW	PDMS
[213]	Inductive	5 × 1	Commercial sensor	Commercial sensor
[199]	Capacitive	16 × 16	CMOS fabrication	Object to detect
[209]	Inductive	2 × 1	CMOS fabrication	CMOS fabrication
[174]	Capacitive	10 × 10	AgNW	PDMS, BT, Zn, Cu
[173]	Capacitive	3 × 3	Cu	Silicone, Ag
[160]	Capacitive	4 × 4	Printed Ag	Printed PDMS, Air
[338]	Fiber Optic	2 × 1	Fiber Optic	Fiber Optic
[198]	Capacitive	480 × 960	CMOS fabrication	Object to detect
[161]	Capacitive	2 × 2	Cu	PET, Silk
[113]	Resistive	From 2 × 2 to 12 × 12	Cu	EGaIn
[137]	Resistive	5 × 5	AgNW	Al, ITO, PDMS, Si
[116]	Resistive	84 × 80 (90 mm × 90 mm)	-	PET, Si
[8]	Resistive	8 threads (crossing 4 vs. 4)	Cu	Silicone
[134]	Resistive	32 × 32	-	-
[356]	Fiber Optic	3 × 1	Fabry-Perot	Fabry-Perot
[112]	Resistive	16 × 8	Cu	Commercial thin-film resistor
[361]	Hall effect	4 × 4	Commercial sensor	Commercial sensor
[139]	Resistive	4 × 4, 8 × 8, 16 × 16	-	-
[269]	Piezoelectric	9 × 9 (1 mm × 1 mm, 50 µm sensor diameter)	Au	Mo, AlN
[305]	Piezoelectric	5 × 1 (1.6 × 2.4 cm)	-	Perovskite rods
[304]	Piezoelectric	8 × 2 (20 cm between columns, 1 column of transmitters, 1 of receivers)	Commercial sensor (PZT)	Commercial sensor (PZT)
[271]	Piezoelectric	6 × 5 (electrode of 2 mm diameter)	Al, Au, Cr	PZT
[218]	Diode	14 × 14	ITO	IGZO
[143]	Capacitive	6 × 6	Au	PDMS, PI
[145]	Capacitive	3 × 3	Cr, Au, Parylene	Porous PDMS NaHC$O_{3}$
[201]	Capacitive	4 × 4 (71 mm height, 0.5 ${\mathrm{cm}}^{2}$ electrode size)	Ag AgCl	Si, ${\mathrm{Ta}}_{2}$$O_{5}$, Si$O_{2}$
[164]	Capacitive	8 × 6	Conductive ink, conductive textiles,	Ecoflex, PDMS
[327]	Triboelectric	4 × 4 (1 × 1 ${\mathrm{cm}}^{2}$)	PEDOT:PSS, glycerol	PMDS, Polycaprolactone
[360]	Hall effect	7 × 4 5 × 3	Commercial sensor	Commercial sensor
[109]	Resistive	From 8 × 1 to 8 × 512 (simulation), 4 × 4 8 × 8 12 × 12 16 × 16 (real)	Commercial resistor	Commercial resistor
[78]	Piezoresistive	4 × 4	Commercial resistor	Commercial resistor
[92]	Capacitive Piezoresistive (heterogeneous)	2 × 1	ITO	CNT, PDMS
[68]	Piezoresistive (heterogeneous)	2 × 1	Ag	MXene
[254]	Piezoelectric	Tennis racket shape	Printed Ag	PVDF
[253]	Piezoelectric	3 × 3, 5 × 5	Au, Ag	(K, Na)Nb$O_{3}$
[257]	Piezoelectric	3 × 1	Cu	PZT
[256]	Piezoelectric	5 × 5	Cu	PZT
[364]	Hall effect	4 × 1	Au	Si, Al, Ta, Py
[314]	Piezoelectric	8 × 5 (300 × 340 ${\mathrm{mm}}^{2}$)	ITO	P(VDF-TrFE)
[247]	Piezoelectric	1 × 3	-	Mo$S_{2}$
[62]	Diode Capacitive Piezoresistive (heterogeneous)	1 × 1 temperature 1 × 1 pressure 1 × 1 UV light 8 × 8 LED display	Au, Al, CNT	CNT, PVDF, Si ZnONW, PAni
[192]	Capacitive	-	-	C0G
[114]	Resistive (heterogeneous)	5 × 4	Commercial sensor	Commercial sensor
[140]	Piezoresistive	6 × 6	-	-
[315]	Triboelectric	16 × 16 3 × 3	Graphene	Kapton, Silicone
[317]	Triboelectric	12 sensors (circular shape)	Al	PTFE
[322]	Triboelectric	6 × 3 0.5 cm per cell	Printed Ag, ITO	PDMS
[318]	Triboelectric	2 × 1, 2 × 1, 4 × 1	Ag	PVDF, PET
[320]	Triboelectric	5 × 5 (11 × 1 cm^2^)	Al	PVDF, PDMS, Nylon
[268]	Piezoelectric	8 × 8	Mo, Al	ITO, Zn, PVDF
[228]	Transistor	256 × 256	Si	ITO
[227]	Transistor	6250 sensors	Au	ITO
[366]	Bioimpedance	2 × 3	Ag	Organic tissue
[65]	Resistive	4 × 4	Cu	rGO
[152]	Capacitive	2 × 2, 5 × 5 (both 9 × 9 cm^2^)	Printed Ag Cu	Resin
[71]	Piezoresistive	10 × 10 (4.5 × 4.5 cm^2^)	Au, Printed Ag	Printed CNT
[94]	Piezoresistive Resistive (heterogeneous)	2 × (7 × 4)	MXene	IGZO, PDSM CNT, MXene
[205]	Capacitive	16 × 16	CMOS fabrication	Object to detect
[311]	Piezoelectric	4 × 1	-	$LiTaO_{3}$
[347]	Fiber Optic	-	Fiber Optic	Fiber Optic
[292]	Piezoelectric	4 × 4	Al	Si, Quartz, Ni
[4]	Piezoelectric	16 × 1 circular shape	Commercial sensor	Commercial sensor
[2]	Piezoelectric	5 × 1	Si	AlN, Graphene oxide, MXene
[288]	Piezoelectric	6 × 1	Ag	BT, PDMS
[354]	Fiber Optic	2 × 1	Fiber Optic	Fiber Optic
[351]	Fiber Optic	-	Fiber Optic	Fiber Optic
[299]	Piezoelectric	6 × 1, 1 × 1	Cu	PZT
[162]	Capacitive	4 × 6 × 6	Cu	PEGDA
[258]	Piezoelectric	5 × 1	Al	PVDF
[1]	Fiber Optic	4 sensors	Fabry-Perot	Fabry-Perot
[334]	Fiber Optic	3 × 1	Fiber Optic	Fiber Optic
[282]	Piezoelectric	3 × 3	Al	PVDF
[355]	Fiber Optic	2 × 1	Fiber Optic	Au, Fiber Optic Polystyrene
[300]	Piezoelectric	4 × 1	Cu	PZT
[70]	Piezoresistive	3 × 3 (1 mm × 1 mm)	Au	PEDOT
[76]	Transistor Piezoresistive	5 × 4	CNT, AgNW	PEDOT, PAAMPSA
[56]	Piezoresistive	4 × 4	Printed Ag, Cu	PDMS, MWCNT
[151]	Capacitive	32 × 32 (4.8 × 2.4 mm)	Standard CMOS	Standard CMOS
[168]	Capacitive	3 × 3 (30 cm × 30 cm)	Fabric, Al	Polyurethane
[97]	Resistive	5 × 5	Pt100	Pt100
[57]	Piezoresistive	3 × 3	Cu, Ag	Sponge Polyaniline
[91]	Piezoresistive	4 × 4 (40 mm each sensor)	Cu, Ni, Au	CNT, PDMS
[51]	Piezoresistive	5 × 5 (11.5 × 11.5 cm^2^)	-	Velostat^TM^
[290]	Piezoelectric	2 × 1	-	-
[191]	Capacitive	8 × 8 (1.5 × 1.5 cm^2^)	PEDOT:PSS, CNT	PVDF, PDMS
[316]	Triboelectric	4 × 1 (one per finger)	Cu	PTFE
[176]	Capacitive	12 × 12 (1 mm × 1 mm)	Ti, Cu	Polyimide
[352]	Fiber Optic	5 × 1	FBG	FBG
[150]	Capacitive	4 × 4 (0.6 cm^2^)	CNE	Ecoflex, PET
[75]	Piezoresistive	100 × 300	Cu	Polymer
[55]	Piezoresistive	5 × 4	Cu, Ag	CNT, Cotton
[297]	Piezoelectric	6 × 1, 1 × 1	Cu	PZT
[200]	Capacitive	2 × 2	Al, Si	Standard CMOS
[287]	Piezoelectric	6 × 4	-	-
[343]	Fiber Optic	3 × 1	Fiber Optic	Fiber Optic
[204]	Capacitive	4 × 4 (4 cm × 4 cm)	Printed CNE	Polyurethane
[350]	Fiber Optic	2 × 2	FBG	FBG
[220]	Diode	3 × 3	Ti, Ni	Si
[246]	Piezoelectric	5 × 5	Ag	PDMS, PVDF
[263]	Piezoelectric	5 × 5	Al	Si, Quartz, Ni
[120]	Resistive	6 × 4	-	-
[178]	Capacitive	6 × 6, 3 × 3 (33 × 33 mm)	Cu	Ecoflex
[225]	Transistor (heterogeneous)	3 × 1	Ag	ZnO-NRs
[88]	Piezoresistive	8 × 8	Copper	CNT, Polyurethane, PDMS
[232]	Transistor	4 × 2	Au, Cr	$MoS_{2}$
[216]	Inductive	3 × 3	Commercial sensor	Commercial sensor
[233]	Transistor	42 × 38	Standard CMOS	Standard CMOS
[133]	Resistive	32 × 32 (7 × 7 mm^2^)	-	-
[362]	Hall effect	3 × 3	Commercial sensor	Commercial sensor
[221]	Transistor	20 × 20 (1 × 1 cm^2^)	ITO	Air, ITO
[307]	Piezoelectric (heterogeneous)	6 × 1	-	Different coatings for piezoelectric sensors: CNT, Polyethilene glycol, Propolis, bromocresol green, Polystyrene
[249]	Piezoelectric	3 × 3	Ag	PVDF
[117]	Resistive	8 × 8	-	-
[10]	Capacitive	Petal shaped array 25 sensors Circle of R = 5 cm	Cu	PDMS
[111]	Resistive	8 × 2	Polymer	-
[156]	Capacitive Piezoelectric	5 × 5 (4 mm^2^)	AgNW	PET
[188]	Capacitive	4 × 3	Cu	Object to detect
[89]	Resistive	3 × 3 (5 cm × 2 cm)	PDMS, Ecoflex	CNT
[167]	Capacitive	3 × 3, 4 × 4, 5 × 5 (1 mm × 1 mm)	Standard CMOS	Standard CMOS
[104]	Resistive	52 × 44 (65 × 55 cm^2^)	-	-
[312]	Piezoelectric	6 sensors (ring shape)	Si	Al, BT
[241]	Piezoelectric	8 × 8 × (6 × 6) 2304 sensors (8 × 8 matrices with 6 × 6 sub matrices)	Au, Ti	Al, PDMS
[273]	Piezoelectric	3 × 1	Cu	PZT
[7]	Resistive	7 sensors (row of 3 over a row of 4)	Printed Ag	PET
[158]	Capacitive	5 × 5, 10 × 10 mm 5 × 1 for gesture recognition	Cu, Al	PVDF
[154]	Capacitive	12 × 12 (5 × 5 mm^2^)	Al, Si, Cu	Air
[283]	Piezoelectric	3 × 3	Au	PZT
[324]	Triboelectric	8 × 8 (38 × 38 cm^2^)	Fabric	Elastic balls
[279]	Piezoelectric	4 × 4	Au, Sn	PZT
[325]	Triboelectric	4 × 4	Cu	Nylon, Kapton, $BaTiO_{3}$
[267]	Piezoelectric	6 × 6	Ti, Au	PMN–PT, PZT
[99]	Piezoresistive	10 × 1	Au	Si
[153]	Capacitive	4 × 1 (heel size)	Cu	-
[250]	Piezoelectric	5 × 4	Printed Ag, PDMS	Printed PVDF PDMS, PZT
[53]	Piezoresistive	3 × 1	Commercial sensor	Commercial sensor
[66]	Resistive	4 × 1 (45 × 45 mm^2^)	Ag	Printed CNT
[302]	Piezoelectric	7 emitters 7 receivers	Cu	PZT
[295]	Piezoelectric	8 sensors	-	-
[121]	Resistive	4 × 4	-	-
[211]	Inductive	2 × 2	CMOS fabrication	CMOS fabrication
[148]	Capacitive	8 × 8 (2025 cm^2^)	Cu	Printed PET
[142]	Capacitive	2 × 2	Au, PET	PDMS
[367]	Bioimpedance	5 × 5 (95 × 100 mm)	Printed Carbon, Ag	Organic tissue
[208]	Inductive	4 × 4 (27 × 27 mm^2^)	Ferrite	PET
[260]	Piezoelectric	5 × 4	Cu	PVDF, PDMS, Ecoflex
[313]	Piezoelectric	-	Cu	PZT
[210]	Inductive	2 × 2	CMOS fabrication	CMOS fabrication
[170]	Capacitive	2 × 2, 10 × 10 (61 × 15 × 15 ${\mathrm{cm}}^{3}$)	Carbon	Ethylene
[179]	Capacitive	Two circles: D = 254 mm 180 and 179 sensors	Cu	Air
[96]	Resistive Capacitive (heterogeneous)	12 × 1 (6 capacitors and 6 resistors)	Commercial sensor	Commercial sensor
[207]	Capacitive	2 × 3	Cu	-
[159]	Capacitive	5 sensors (for gesture recognition), 5 × 5 (for weight measurement)	AgNW, PDMS	Fe, Carbon, PDMS
[63]	Piezoelectric, Triboelectric, Piezoresistive, Capacitive (heterogeneous)	10 × 10	AgNW	CNT, PDMS, PZT, CNT
[195]	Capacitive	2 × 2	Graphene	Ionic gel
[132]	Resistive	32 × 32 (7 × 7 mm^2^)	-	-
[74]	Piezoresistive	2 × 3 (shoe shape)	Flexiforce	FlexiForce
[203]	Capacitive	2 × 6 × 3 (2.5 × 5 mm^2^)	Si, Au, Cr	Air
[110]	Resistive	16 × 16 (32 × 32 cm^2^)	Cu	Velostat^TM^
[72]	Piezoresistive	5 × 4 (3 × 4 cm^2^)	Ag	Printed Carbon, Polymer
[197]	Capacitive	8 × 8 (1 cm × 1 cm)	Standard CMOS	Object to detect
[272]	Piezoelectric	4 × 1	Cu	PZT
[252]	Piezoelectric	2 × 2	Au	PVDF, PDMS
[266]	Piezoelectric	2 × 2	Au	Printed PVDF, PDMS, PtrFE
[277]	Piezoelectric	6 × 1	Commercial sensor	Commercial sensor
[276]	Piezoelectric	6 × 1	Commercial sensor	Commercial sensor
[175]	Capacitive	5 × 5, 4 × 1 (30 × 30 mm^2^)	AgNW	PDMS
[172]	Capacitive	10 × 10 (5 × 5 cm^2^)	Graphene, PET	Air, PDMS
[261]	Piezoelectric	1 × 4, 3 × 3	Cu, Pt, Ti, Ag	PZT
[206]	Capacitive (heterogeneous)	4 gas sensors 8 textile tensile sensors	Commercial sensor	Commercial sensor
[181]	Capacitive	4 × 4 (30 × 30 cm^2^)	FR4	Object to detect
[171]	Capacitive	11 sensors (approx. 15 × 25 cm)	Cu	-
[50]	Piezoresistive	3 × 1 (5 × 25 mm^2^)	Cu	Carbon, Si
[310]	Piezoelectric	4 × 1	Au, Pt, PZT	Graphene, Si, PZT
[262]	Piezoelectric	5 × 5	Al	Si, Quartz, Ni
[118]	Resistive	8 × 2	Polymer	-
[59]	Piezoresistive	6 × 8	Cr, Au	CNT, PDMS
[60]	Piezoresistive	3 × 2 (15 × 20 mm^2^)	Cu	CNT, PDMS
[119]	Resistive	8 × 8	-	-
[73]	Piezoresistive	2 × 2 (10 × 10 mm^2^)	Ag	Si
[341]	Fiber Optic	-	Fiber Optic	Fiber Optic
[177]	Capacitive	2 × 1 (one on the wrist and one on the arm)	Standard CMOS	Air
[219]	Diode	100 × 100, 80 × 80, 50 × 50	Au	Si
[155]	Capacitive	5 × 5 size of a hand	Graphene, AgNW	Polyurethane
[187]	Capacitive	3 × 4	Cu	-
[357]	Hall effect	8 × 8	Commercial sensor	Commercial sensor
[348]	Fiber Optic	4 × 1	Fiber Optic	Fiber Optic
[349]	Fiber Optic	64 sensors	FBG	FBG
[339]	Fiber Optic	8 × 1	Fiber Optic	Fiber Optic
[166]	Capacitive	3 × 3	Cu	-
[136]	Resistive	3 × 3	-	-
[278]	Piezoelectric	3 × 1	-	PVDF, Zr, Ti
[61]	Capacitive Resistive Triboelectric (heterogeneous)	5 × 5 (60 mm × 60 mm)	AgNW	Silicone
[289]	Piezoelectric	2 × (5 × 4)	Commercial sensor	Commercial sensor
[284]	Piezoelectric	9 × 1	-	-
[105]	Piezoresistive	7 × 1	-	-
[106]	Piezoresistive	13 × 5	-	-
[127]	Resistive	8 × 6	-	-
[54]	Piezoresistive	12 × 8	PAM, Tuolene	PEDOT
[321]	Triboelectric	3 × 3 (5 × 5 cm^2^)	ITO	PDMS, Glass
[326]	Triboelectric	PC keyboard	CNT	Silicone, Polyurethane, Air
[58]	Piezoresistive	2 × 2 × 5 (5 sensors in each neuron)	Standard CMOS	Printed Standard CMOS
[286]	Piezoelectric	5 × 1 (1.5 × 53 mm^2^)	PVDF	Polyurethane
[222]	Transistor	12 × 12, 50 × 50 (2.5 × 2.5 cm^2^)	AgNW, Cr, Au	Air, PDMS, Graphene
[217]	Transistor Diode	9 × 1	Custom process (similar to CMOS)	Custom process (similar to CMOS)
[231]	Transistor	2 × 1	-	Air, Si
[226]	Transistor	8 × 1	AlGaN GaN	PMMA
[230]	Transistor	32 × 32	Standard CMOS	Standard CMOS
[6]	Piezoelectric	2 sensors (perpendicular)	Pt	PZT
[115]	Resistive	From 8 sensors to 449 sensors	-	-
[67]	Piezoresistive	3 × 4	FSR	FSR
[5]	Piezoelectric	High number of electrodes orientated at, 0°, 30°, 45°, 60°, and 90°	Al, Pt	Al
[259]	Piezoelectric	3 × 3 (12 × 6 cm^2^)	Cu	PDMS
[264]	Piezoelectric	3 × 3, 6 × 6	Au	ZnONW
[275]	Piezoelectric	4 × 4	Al	PET
[135]	Resistive	4 × 4	-	-
[298]	Piezoelectric	8 × 1	Cu	-
[223]	Transistor	11 × 1	-	$MoS_{3}$, Air
[69]	Piezoresistive	2 × 2 (4 × 4 mm^2^)	Al	Air
[98]	Resistive (heterogeneous)	6 × 1	Commercial sensor	Commercial sensor
[196]	Capacitive	8 × 8 (1 cm × 1 cm)	Standard CMOS	Object to detect
[131]	Resistive	8 × 6	-	-
[9]	Capacitive	1 × 108, one per disc in the transformer windings	-	Air
[3]	Piezoresistive	10 × 1	Ag	PAni
[124]	Resistive	16 × 8	-	-
[165]	Capacitive	450 × 450	-	-
[95]	Resistive	16 × 16	Printed Al, Ag	Printed PEN, Al
[157]	Capacitive	8 × 8	Steel	Fabric
[180]	Capacitive	4 × 1 (10 cm × 4 cm)	Cu	Object to detect
[123]	Resistive	From 8 × 8 to 256 × 256	-	-
[64]	Piezoresistive	4 × 4 (40 × 50 mm^2^)	Au	Printed PEDOT, Kapton
[239]	Transistor	3 × 3	Au, Cr	Graphene
[90]	Piezoresistive	2 × 2	-	PDMS, Si
[125]	Resistive	8 × 6	-	-
[234]	Transistor	2 × 1	Au	CNT
[202]	Capacitive	3 × 2 (6 × 9 mm)	Au, Si	-
[359]	Hall effect	8 × 8	Commercial sensor	Commercial sensor
[265]	Piezoelectric	3 × 2	Al	PVDF
[296]	Piezoelectric	4 sensors	Cu	PZT
[335]	Fiber Optic	80 × 1	FBG	FBG
[309]	Piezoelectric	2 × 1	Au	Si, Quartz, Ni
[345]	Fiber Optic	128 × 1	Fiber Optic	Fiber Optic
[306]	Piezoelectric (heterogeneous)	18 sensors	-	Quartz, CNT
[303]	Piezoelectric	2 × 2	Ag	Ag, PZT
[294]	Piezoelectric	3 × 1, 1 × 1	Cu	PZT
[333]	Fiber Optic	4 × 2, 1 × 4	FBG	FBG
[274]	Piezoelectric	1 × 4, 3 × 3	Cu, Pt, Ti, Ag	PZT
[332]	Fiber Optic	4 × 1	FBG	FBG
[308]	Piezoelectric	2 × 2	Mo	AlN
[291]	Piezoelectric	2 × 1	Cu	PZT
[122]	Resistive	8 × 8	-	-
[293]	Piezoelectric	6 × 1	Cu, Printed Ag	PZT
[270]	Piezoelectric	3 × 3	Ag, Al, Au	PZT, Si
[128]	Resistive	4 × 4	-	-
[323]	Triboelectric	6 × 3 1 cm per cell	Cu, PDMS	PTFE
[130]	Resistive	5 × 3	-	-
[126]	Resistive	From 8 sensors to 449 sensors	-	-
[129]	Resistive	20 × 20	-	-

## Appendix B

**Table A2:** Analysis of the type of array (wearable or environmental), application, measured variable, and validation experiment. This table includes the studies not analyzed in Table 3.

Study	Wearable or Environmental	Application	Measured Variable	Validation Experiments
[183]	Environmental	Pressure	Pressure	Customized object or stamp Motor
[337]	Environmental	Temperature	Temperature	Computational simulation
[365]	Environmental	Magnetic field detection	Position Angular position	Magnetic machine Computational simulation
[251]	Environmental Wearable	Pressure Strain	Pressure Strain	Motor Robot Customized object or stamp Subjects wearing the device
[363]	Environmental	Electric system monitoring	Current	Computational simulation Lab experiment
[103]	Environmental	Magnetic field detection	Magnetic Field	Magnetic machine
[346]	Environmental	Surgery	Position	Motor
[331]	Environmental	HMI Walking assessment	Pressure	Subjects interacting externally with the device Computational simulation
[102]	Environmental	Organic compounds Airflow applications	Concentration Pressure	Wind machine Computational simulation
[330]	Environmental	Skin health	Force	Vibration machine Subjects interacting externally with the device
[248]	Environmental Wearable	Pressure Plantar pressure	Pressure	Motor Force gauge Subjects wearing the device
[329]	Wearable	Gesture recognition Sport activities Heart monitoring Electronic skin	Force	Force gauge Motor Subjects wearing the device
[93]	Environmental	Improve readout accuracy	Force	Force gauge Customized object or stamp
[328]	Wearable	HMI	Pressure	Force gauge Customized object or stamp Subjects interacting externally with the device Motor Robot
[242]	Environmental	Marine applications	Pressure	Motor Lab experiment Computational simulation
[301]	Environmental	SHM	Sound	Computational simulation Lab experiment
[107]	Environmental	Improve readout accuracy	Resistance	Computational simulation
[163]	Wearable	HMI	Pressure	Customized object or stamp Force gauge Computational simulation
[255]	Wearable	Walking assessment	Pressure	Subjects wearing the device
[319]	Environmental	HMI	Pressure	Subjects interacting externally with the device Motor
[285]	Wearable	Heart monitoring	Force	Motor Subjects interacting externally with the device
[87]	Wearable	Heart monitoring Speech detection Sport activities Walking assessment Electronic skin	Pressure	Customized object or stamp Subjects interacting externally with the device Subjects wearing the device Motor
[149]	Environmental	Pressure	Pressure	Customized object or stamp Motor Subjects interacting externally with the device
[86]	Wearable	Posture assessment	Pressure	Customized object or stamp Motor
[280]	Environmental	SHM	Sound	Lab experiment
[85]	Wearable	Electronic skin	Pressure	Customized object or stamp Motor Robot
[281]	Environmental	Electric system monitoring	Vibration	Computational simulation Lab experiment
[84]	Environmental	Electronic skin	Pressure	Computational simulation
[83]	Environmental	Pressure	Pressure	Customized object or stamp Force gauge
[82]	Environmental	Posture assessment	Pressure	Force gauge Subjects interacting externally with the device
[81]	Environmental	Posture assessment	Pressure	Customized object or stamp Subjects interacting externally with the device
[80]	Wearable	Posture assessment	Strain	Force gauge Subjects wearing the device
[141]	Environmental	Improve readout accuracy	Resistance	Computational simulation
[108]	Environmental	Walking assessment	Resistance	Computational simulation Subjects interacting externally with the device
[101]	Environmental	Inorganic compounds	Concentration	Chemical testing
[79]	Environmental	Improve readout accuracy	Pressure	Computational simulation Lab experiment Subjects interacting externally with the device
[138]	Environmental	Improve readout accuracy	Resistance	Computational simulation Lab experiment
[340]	Environmental	Sound	Sound	Acoustic machine
[189]	Environmental	Imaging	Presence	Computational simulation Lab experiment
[193]	Environmental	Organic compounds	Concentration	Chemical testing
[186]	Environmental	Gesture recognition	Force	Motor Subjects interacting externally with the device
[344]	Environmental	Electric system monitoring	Sound	Lab experiment
[342]	Environmental	Sound	Sound	Computational simulation Motor
[238]	Environmental	Organic compounds	Concentration	Chemical testing
[224]	Wearable Environmental	Heart monitoring	Pressure	Customized object or stamp Motor Force gauge Subjects interacting externally with the device
[237]	Environmental	Organic compounds	Concentration	Chemical testing
[236]	Environmental	Organic compounds	Concentration	Chemical testing
[52]	Environmental	Electronic skin	Pressure Temperature	Temperature machine Motor Subjects interacting externally with the device Customized object or stamp
[240]	Environmental	Dopamine	Concentration	Chemical testing
[215]	Environmental	SHM	Impedance	Lab experiment
[214]	Wearable	Gesture recognition	Impedance	Subjects wearing the device Subjects interacting externally with the device
[212]	Environmental	Pressure	Pressure	Subjects interacting externally with the device
[194]	Environmental	Inorganic compounds	Concentration	Computational simulation Lab experiment
[336]	Environmental	Sound	Sound Pressure	Lab experiment
[146]	Environmental	Electronic skin	Force	Subjects interacting externally with the device Motor Robot
[245]	Wearable Environmental	Posture assessment Walking assessment Speech detection	Pressure	Vibration machine Force gauge Subjects interacting externally with the device Subjects wearing the device Daily life objects Customized object or stamp Computational simulation
[243]	Wearable	Eye tracking	Pressure	Subjects wearing the device Motor
[77]	Environmental	Posture assessment	Pressure	Subjects interacting externally with the device
[100]	Environmental	Organic compounds	Concentration	Chemical testing
[190]	Environmental	Imaging Electronic skin	Presence	Robot
[358]	Environmental	Surgery	Position	Motor
[244]	Environmental	Heart monitoring	Pressure	Subjects interacting externally with the device Motor Force gauge
[144]	Environmental	Plantar pressure	Pressure	Subjects interacting externally with the device Motor
[147]	Environmental	Pressure Imaging	Pressure	Computational simulation Motor Customized object or stamp Subjects interacting externally with the device
[185]	Wearable	HMI Electronic skin	Pressure	Subjects interacting externally with the device Computational simulations Daily life objects Customized object or stamp Force gauge
[184]	Environmental	Electronic skin	Force Presence	Robot
[182]	Environmental	HMI	Presence	Subjects interacting externally with the device
[235]	Environmental	Organic compounds Inorganic compounds	Concentration	Chemical testing
[229]	Environmental	Imaging	Light	Computational simulation, Lab experiment, Light machine
[353]	Environmental	Radiation	Light	Lab experiment
[169]	Wearable	HMI	Strain	Force gauge
[213]	Environmental	Gesture recognition	Presence	Subjects interacting externally with the device
[199]	Environmental	DNA	Concentration	Computational simulation Chemical testing
[209]	Environmental	HMI	Force	Force gauge
[174]	Environmental Wearable	Electronic skin	Strain	Subjects wearing the device Subjects interacting externally with the device
[173]	Environmental	Electric system monitoring	Strain Temperature	Computational simulation Temperature machine
[160]	Environmental Wearable	Improve readout accuracy Speech detection	Pressure Sound	Computational simulation Force gauge Subjects wearing the device Subjects interacting externally with the device
[338]	Environmental	Improve readout accuracy	Sound	Lab experiment Computational simulation
[198]	Environmental	Cell concentration	Concentration	Chemical testing
[161]	Wearable	Pressure Skin health	Pressure	Force gauge
[113]	Wearable	Improve readout accuracy	Force	Force gauge
[137]	Wearable	HMI Improve readout accuracy	Pressure	Subjects wearing the device Force gauge
[116]	Environmental	Improve readout accuracy	Pressure	Force gauge Daily life objects
[8]	Wearable	Improve readout accuracy	Strain	Computational simulation Subjects interacting externally with the device
[134]	Environmental	Improve readout accuracy	Resistance	Computational simulation
[356]	Environmental	Organic compounds	Concentration	Motor Chemical testing
[112]	Environmental	Organic compounds Inorganic compounds Improve readout accuracy	Concentration	Lab experiment
[361]	Wearable	Surgery	Position	Robot Subjects wearing the device
[139]	Environmental	Improve readout accuracy	Resistance	Computational simulation
[269]	Environmental	Strain	Strain	Computational simulation
[305]	Environmental	Speech detection HMI	Sound	Computational simulation Loudspeaker Subjects interacting externally with the device
[304]	Environmental	SHM	Sound	Lab experiment
[271]	Environmental	HMI Sound	Pressure	Computational simulation Loudspeaker Subjects wearing the device Subjects interacting externally with the device
[218]	Environmental	Imaging	Light	Light machine Motor
[143]	Wearable	Heart monitoring Respiration monitoring	Pressure	Computational simulation Customized object or stamp Daily life objects Subjects interacting externally with the device Subjects wearing the device
[145]	Environmental	Respiration monitoring Speech detection Sport activities Electronic skin	Pressure	Computational simulation Subjects wearing the device Subjects interacting externally with the device Customized object or stamp Daily life objects
[201]	Environmental	pH Inorganic compounds	Concentration	Chemical testing
[164]	Environmental	Electronic skin	Pressure	Robot Force gauge Customized object or stamp
[327]	Wearable	Walking assessment Electronic skin	Pressure	Motor Customized object or stamp Rotatory table Subjects interacting externally with the device
[360]	Environmental	Robot control	Position	Computational simulation Robot
[109]	Environmental	Improve readout accuracy	Resistance	Computational simulations Lab experiment Customized object or stamp
[78]	Wearable	Posture assessment	Strain	Lab experiment Subjects wearing the device
[92]	Environmental Wearable	Heart monitoring Speech detection	Pressure Sound	Computational simulation Subjects wearing the device Subjects interacting externally with the device
[68]	Environmental	Sport activities Gesture recognition	Pressure Acceleration	Subjects interacting externally with the device Daily life objects
[254]	Environmental	Sport activities	Pressure Position	Force gauge
[253]	Environmental	Pressure Energy generation	Pressure	Force gauge
[257]	Wearable	Heart monitoring	Pressure	Subjects wearing the device
[256]	Environmental	Marine applications	Pressure	Computational simulation Lab experiment
[364]	Environmental	Magnetic field detection	Magnetic Field	Computational simulation
[314]	Environmental	Gesture recognition	Temperature	Computational simulation Subjects interacting externally with the device
[247]	Wearable	Pressure	Pressure	Force gauge
[62]	Wearable	HMI Pressure	Pressure Temperature Light	Subjects wearing the device
[192]	Environmental	Improve readout accuracy	Humidity	Lab experiment
[114]	Environmental Wearable	HMI	Temperature Force Light	Subjects wearing the device
[140]	Environmental	Pressure Improve readout accuracy Plantar pressure	Pressure	Lab experiment Computational simulation
[315]	Environmental Wearable	HMI	Pressure	Subjects interacting externally with the device Computational simulation
[317]	Environmental	Airflow applications Energy generation	Pressure	Wind machine Computational simulation
[322]	Wearable	Electronic skin	Force	Subjects interacting externally with the device Subjects wearing the device
[318]	Environmental	Energy generation	Force Energy	Motor
[320]	Environmental	Sport activities	Force	Motor
[268]	Environmental	HMI	Pressure	Customized object or stamp Lab experiment
[228]	Environmental	Imaging	Light	Lab experiment Light machine
[227]	Environmental	Imaging	Light	Customized object or stamp Lab experiment Light machine
[366]	Wearable	Heart monitoring	Pressure	Subjects wearing the device
[65]	Wearable	Electronic skin Pressure	Pressure	Force gauge
[152]	Environmental	SHM	Force Position	Force gauge
[71]	Environmental Wearable	Electronic skin	Pressure	Vibration machine
[94]	Wearable	Electronic skin	Pressure Temperature	Subjects interacting externally with the device
[205]	Environmental	DNA	Concentration	Computational simulation Chemical testing
[311]	Environmental	Sound	Sound	Computational simulation
[347]	Environmental	Simulation	Noise	Computational simulation Lab experiment
[292]	Environmental	SHM	Sound	Computational simulation Lab experiment
[4]	Environmental	SHM	Sound	Computational simulation Lab experiment
[2]	Environmental	Organic compounds	Concentration	Chemical testing
[288]	Environmental	Respiration monitoring	Pressure	Computational simulation Lab experiment
[354]	Environmental	Imaging	-	-
[351]	Environmental	Aerospace applications	Strain	-
[299]	Environmental	SHM	Sound	Lab experiment
[162]	Wearable	Swallowing detection HMI	Pressure	Force gauge Subjects wearing the device
[258]	Wearable	Security	Strain	Daily life objects
[1]	Environmental	Sound Imaging	Sound	Acoustic machine Motor
[334]	Environmental	Temperature Curvature	Curvature Temperature	Force gauge Computational simulation
[282]	Wearable	Heart monitoring	Pressure	Daily life objects
[355]	Environmental	Cancer detection	Concentration	Chemical testing
[300]	Environmental	SHM	Sound	Lab experiment
[70]	Wearable	Bite monitoring Pressure	Pressure	Subjects wearing the device
[76]	Wearable	Force, Pressure HMI, Electronic skin	Strain Pressure	Robot
[56]	Wearable	Skin health	Pressure	Force gauge Subjects wearing the device
[151]	Environmental	Force Pressure	Pressure	Customized object or stamp Force gauge
[168]	Environmental	Posture assessment	Pressure	Force gauge
[97]	Environmental	Temperature Improve readout accuracy	Temperature	Computational simulation Temperature machine
[57]	Wearable Environmental	HMI	Pressure	Subjects interacting externally with the device Subjects wearing the device
[91]	Environmental	Airflow applications	Pressure Speed	Wind machine
[51]	Wearable Environmental	HMI Improve readout accuracy	Pressure	Force gauge
[290]	-	Improve readout accuracy Speech detection	Vibration	Computational simulation
[191]	Environmental	Electronic skin	Pressure	Force gauge Customized object or stamp
[316]	Wearable	HMI Gesture recognition	Angular position Angular speed	Robot Motor Subjects wearing the device Computational simulation
[176]	Environmental Wearable	HMI, Electronic skin, Improve readout accuracy, Pressure	Pressure	Force gauge
[352]	Environmental	Aerospace applications Curvature Position Angular position	Position	Computational simulation Force gauge
[150]	Wearable	Heart monitoring Sport activities	Pressure	Subjects wearing the device
[75]	Environmental	Improve readout accuracy	Resistance	Computational simulation Customized object or stamp
[55]	Environmental	HMI Curvature	Curvature Angular position Pressure	Daily life objects
[297]	Environmental	SHM Curvature	Sound	Temperature machine
[200]	Environmental	Organic Compounds, Improve readout accuracy, Food quality, DNA	Concentration	Computational simulation
[287]	Environmental	Respiration monitoring	Pressure	Subjects interacting externally with the device
[343]	Environmental	Electric system monitoring	Sound	Lab experiment
[204]	Wearable	Force	Pressure Force	Force gauge Subjects wearing the device
[350]	Environmental	Security	Vibration	Lab experiment
[220]	Wearable	Blood monitoring	Concentration	Chemical testing
[246]	Wearable	Electronic skin Heart monitoring Pressure	Pressure Angular position	Computational simulation Lab experiment Robot Subjects wearing the device
[263]	Environmental	Sport activities	Sound	Daily life objects
[120]	-	Improve readout accuracy	Resistance	Lab experiment Computational simulation
[178]	Environmental	Force Improve readout accuracy Pressure	Pressure Shear Force Angular position	Force gauge
[225]	Environmental	Inorganic compounds	Concentration	Chemical testing
[88]	Environmental	HMI Force	Shear Force	Customized object or stamp
[232]	Environmental	pH	pH	Chemical testing
[216]	Environmental	Imaging	Presence	Lab experiment
[233]	Environmental	Organic compounds Food quality	Concentration	Chemical testing
[133]	Environmental	Improve readout accuracy	Concentration Resistance	Lab experiment Computational simulation
[362]	Environmental	Electric system monitoring	Current	Lab experiment
[221]	Wearable	HMI	Pressure	Force gauge
[307]	Environmental	Respiration monitoring	Concentration	Chemical testing
[249]	Environmental	Electric system monitoring	Temperature Force	Motor
[117]	-	Improve readout accuracy	Resistance	Computational simulation
[10]	Environmental	Electronic skin Pressure	Pressure	Force gauge
[111]	-	Improve readout accuracy Organic compounds	Resistance	Lab experiment Computational simulation
[156]	Wearable	Respiration monitoring	Pressure	Motor Subjects interacting externally with the device
[188]	Environmental	HMI Imaging Pressure	Pressure Presence	Subjects interacting externally with the device
[89]	Wearable	Gesture recognition	Pressure	Customized object or stamp Subjects interacting externally with the device
[167]	Environmental	Force Pressure	Pressure	Force gauge
[104]	Environmental	Walking assessment Plantar pressure Improve readout accuracy	Pressure	Lab experiment Subjects interacting externally with the device
[312]	Environmental	Respiration monitoring Humidity	Humidity	Lab experiment
[241]	Environmental	Force	Force	Motor Force gauge
[273]	Environmental	SHM	Strain	Lab experiment
[7]	Environmental	Organic compounds	Concentration	Chemical testing
[158]	Wearable	Electronic skin Speech detection Swallowing detection	Pressure Angular position	Subjects wearing the device
[154]	Environmental	HMI Electronic skin	Pressure	Force gauge Customized object or stamp
[283]	Wearable	Heart monitoring	Strain	Lab experiment
[324]	Environmental	Plantar pressure Pressure	Pressure	Subjects interacting externally with the device
[279]	Environmental	Force	Force	Computational simulation
[325]	Environmental	Plantar pressure Pressure	Pressure	Customized object or stamp Subjects interacting externally with the device Computational simulation
[267]	Environmental	Material characterization Force	Strain Shear Force	Force gauge Acoustic machine
[99]	Environmental	Improve readout accuracy Organic compounds Inorganic compounds	Concentration	Chemical testing
[153]	Wearable	Walking assessment	Pressure	Subjects wearing the device
[250]	Wearable	Swallowing detection Speech detection	Pressure	Subjects wearing the device Subjects interacting externally with the device
[53]	Wearable	Gesture recognition	Force	Subjects wearing the device
[66]	-	Surgery	Strain	Force gauge
[302]	Environmental	SHM	Sound	Lab experiment
[295]	Environmental	Material characterization	Sound	Loudspeaker
[121]	-	Improve readout accuracy	Resistance	Computational simulation
[211]	Environmental	Force Improve readout accuracy	Force	Force gauge
[148]	Environmental	Gesture recognition Pressure	Pressure	Customized object or stamp Subjects interacting externally with the device
[142]	Wearable	Electronic skin HMI Pressure	Pressure	Force gauge Subjects wearing the device
[367]	Wearable	Skin health	Impedance	Subjects wearing the device
[208]	Wearable	HMI Pressure	Pressure	Force gauge Subjects wearing the device
[260]	Environmental Wearable	Electronic skin Pressure	Pressure	Robot Subjects interacting externally with the device
[313]	Environmental	Energy generation	Energy	-
[210]	Environmental	HMI Improve readout accuracy	Force	Force gauge
[170]	Environmental	SHM	Strain	Force gauge
[179]	Environmental	Angular position Improve readout accuracy	Angular displacement	Rotatory table Force gauge
[96]	Environmental	Improve readout accuracy Energy generation Food quality	Temperature Humidity	Control humidity chamber
[207]	Environmental	Marine applications	Presence	Lab experiment
[159]	Environmental Wearable	Heart monitoring Speech detection Sport activities	Presión	Subjects wearing the device
[63]	Wearable	Energy generation	Force Energy	Force gauge
[195]	Environmental	HMI	Position	Computational simulation Subjects interacting externally with the device
[132]	Environmental	Improve readout accuracy	Concentration Resistance	Lab experiment Computational simulation
[74]	Wearable	Walking assessment	Pressure	Subjects wearing the device
[203]	Environmental	Organic compounds Improve readout accuracy	Concentration	Chemical testing
[110]	Environmental	Improve readout accuracy Plantar pressure	Resistance	Computational simulation
[72]	Environmental	Improve readout accuracy Pressure	Pressure	Force gauge Customized object or stamp
[197]	Environmental	Cell concentration	Concentration	Chemical testing
[272]	Environmental	SHM	Force Position	Force gauge
[252]	Environmental Wearable	Force	Pressure Strain	Lab experiment
[266]	Environmental	Force	Shear Force	Vibration machine
[277]	Wearable	HMI	Pressure	Subjects wearing the device
[276]	Wearable	HMI	Pressure	Subjects wearing the device
[175]	Environmental Wearable	Electronic skin	Pressure	Motor
[172]	Environmental	HMI Pressure	Pressure	Force gauge Computational simulation Control humidity chamber
[261]	Environmental	SHM	Strain	Force gauge Subjects interacting externally with the device
[206]	Environmental	Organic compounds Improve readout accuracy Force	Concentration Force	Chemical testing Force gauge
[181]	Environmental	HMI	Presence	Computational simulation Subjects interacting externally with the device
[171]	Environmental	SHM	Strain	Force gauge
[50]	Environmental	Improve readout accuracy	Pressure	Force gauge
[310]	Environmental	Humidity	Humidity Temperature	Control humidity chamber
[262]	Environmental	Sport activities	Sound	Daily life objects
[118]	-	Improve readout accuracy Organic compounds	Resistance	Lab experiment Computational simulation
[59]	Wearable	HMI	Pressure	Force gauge
[60]	Wearable	Electronic skin	Pressure Temperature Strain	Computational simulation Daily life objects
[119]	-	Improve readout accuracy	Resistance	Computational simulation
[73]	Environmental	HMI	Force	Motor Computational simulation
[341]	Environmental	-	Sound	-
[177]	Wearable	Heart monitoring	Pressure	Computational simulation
[219]	Environmental	Imaging Radiation	Light	Lab experiment
[155]	Wearable	HMI Pressure	Pressure	Subjects interacting externally with the device
[187]	Environmental	Imaging	Presence	Lab experiment
[357]	Environmental	Sport activities	Position	Lab experiment
[348]	Environmental	Improve readout accuracy	Vibration	Vibration machine
[349]	Environmental	Improve readout accuracy	Vibration	Lab experiment Vibration machine
[339]	Environmental	Sound Speech detection	Sound	Acoustic machine
[166]	Wearable	Improve readout accuracy	Pressure	Lab experiment
[136]	-	Improve readout accuracy	Resistance	Computational simulation
[278]	Environmental	Aerospace applications	Vibration	Computational simulation Wind machine
[61]	Environmental	Energy generation Force	Force Strain	Force gauge
[289]	Environmental	Aerospace applications	Pressure	Wind machine
[284]	Environmental	Heart monitoring	Pressure	Subjects interacting externally with the device
[105]	Environmental	Plantar pressure Improve readout accuracy	Pressure	Computational simulation
[106]	-	Improve readout accuracy	Pressure	Computational simulation
[127]	-	Improve readout accuracy	Resistance	Lab experiment Computational simulation
[54]	Environmental	HMI	Force Strain	Force gauge Subjects interacting externally with the device
[321]	Environmental	Energy generation	Force Energy	Lab experiment
[326]	Environmental	HMI Pressure	Pressure	Force gauge Subjects interacting externally with the device Computational simulation
[58]	Environmental	Posture assessment	Pressure	Computational simulation
[286]	Wearable	Swallowing detection	Pressure	Subjects interacting externally with the device
[222]	Environmental	Electronic skin Pressure	Pressure	Customized object or stamp
[217]	Environmental	pH	pH, Temperature, Current	Chemical testing
[231]	Environmental	Improve readout accuracy	Concentration	Computational simulation
[226]	Environmental	Organic compounds DNA	Concentration	Chemical testing
[230]	Environmental	Imaging	Light	Lab experiment
[6]	Environmental	Airflow applications	Pressure	Wind machine Rotatory Table
[115]	-	Improve readout accuracy	Resistance	Computational simulation
[67]	Environmental	Pressure	Pressure	Customized object or stamp
[5]	Environmental	Magnetic field detection	Magnetic Field	Magnetic machine
[259]	Wearable	Plantar pressure	Strain	Force gauge Motor Subjects wearing the device
[264]	Wearable	Electronic skin Pressure	Pressure Temperature	Daily life objects
[275]	Wearable	HMI Material characterization	Force	Force gauge Robot
[135]	-	Improve readout accuracy	Resistance	Computational simulation
[298]	Environmental	SHM	Sound	Lab experiment
[223]	Environmental	Force	Force	Computational simulation
[69]	Environmental	Aerospace applications	Pressure	Temperature machine
[98]	Environmental	Inorganic compounds Organic compounds Cell concentration Food quality	Concentration	Computational simulation
[196]	Environmental	Cell concentration	Concentration	Lab experiment
[131]	-	Improve readout accuracy	Resistance	Lab experiment
[9]	Environmental	Electric system monitoring	Voltage	Lab experiment
[3]	Environmental	Marine applications	Pressure	Motor
[124]	-	Improve readout accuracy	Resistance	Lab experiment Computational simulation
[165]	Environmental	Fingerprint recognition	Pressure	Computational simulation
[95]	Environmental Wearable	Electronic skin	Temperature	Lab experiment
[157]	Environmental	HMI Pressure	Pressure	Force gauge
[180]	Environmental	Imaging	Presence	Lab experiment
[123]	-	Improve readout accuracy	Resistance	Computational simulation
[64]	Environmental	Electronic skin Pressure	Pressure	Force gauge
[239]	Environmental	DNA	Concentration	Lab experiment
[90]	Environmental	HMI	Strain	Computational simulation
[125]	-	Improve readout accuracy	Resistance	Lab experiment Computational simulation
[234]	Environmental	pH	pH	Lab experiment
[202]	Environmental	Respiration monitoring Improve readout accuracy	Concentration	Subjects interacting externally with the device
[359]	Environmental	Robot control Surgery	Position	Robot
[265]	Wearable	Force	Shear Force	Force gauge
[296]	Environmental	SHM	Sound	Computational simulation
[335]	Environmental	Aerospace applications	Angular acceleration, Angular speed, Angular position, Strain	Computational simulation Lab experiment
[309]	Environmental	Heart monitoring Magnetic field detection	Magnetic Field	Magnetic machine
[345]	Environmental	Surgery	Position	-
[306]	Environmental	Organic compounds	Concentration	Chemical testing
[303]	Wearable	Surgery	Sound	Subjects wearing the device Lab experiment
[294]	Environmental	SHM	Sound	Lab experiment
[333]	Environmental	Material characterization	Strain	Force gauge
[274]	Environmental	SHM	Strain	Force gauge Lab experiment
[332]	Environmental	Strain	Strain	Vibration machine
[308]	Environmental	Organic compounds	Concentration	Chemical testing Magnetic machine
[291]	Environmental	SHM	Sound	Lab experiment
[122]	-	Improve readout accuracy	Resistance	Computational simulation
[293]	Environmental	Sound Material characterization	Sound	Loudspeaker
[270]	Environmental	Sport activities	Strain	Subjects interacting externally with the device
[128]	-	Improve readout accuracy	Resistance	-
[323]	Environmental	Plantar pressure	Pressure	Customized object or stamp Force gauge Computational simulation
[130]	-	Improve readout accuracy	Resistance	Lab experiment
[126]	-	Improve readout accuracy	Resistance	Lab experiment Computational simulation
[129]	-	Improve readout accuracy	Resistance	Lab experiment

## Appendix C

**Table A3:** Analysis of the software used for processing, the sensor array characteristics, and the metrics used to evaluate performance. This table includes the studies not analyzed in Table 6. Parameters labeled as *visually* present the corresponding result only graphically instead of numeric values.

Study	Software for Analysis	Characteristics	Metrics
[183]	LabView, Spice	Sampling frequency (1 kHz), Cost (low cost due to unstructured sensor)	Accuracy (97%), MARE (5.2%), Frequency response (10 kHz cutoff freq), Sensing range (up to 8 N), CD (>0.9), MAE (average = 0.94 N, all sensors in range [0.52, 1.11] N)
[337]	CATIA	ADC bits (12), Sampling frequency (10 Hz), Effects of ECs, Energy consumption (1.2 kWh)	Sensing range (−40 to 200 °C), Resolution (0.0625 °C), MAE (0.5 °C), RMSE (0.7 °C), Correlation coefficient (0.95), Stability (ensured by design), Drift (due to ECs), Response time (2 s), Accuracy (95%), Repeatability (std = 2.1 °C)
[365]	-	Spatial resolution (1 ${\mathrm{mm}}^{2}$)	Noise (36 pT/$\sqrt{\mathrm{Hz}}$ at 10 Hz), MSE (2.4615 mm best value), Absolute error ([x, y] = [0.142, 0.042] best values), MARE (<15.6% in position, <13% in orientation)
[251]	ANSYS	Power consumption (power density generated = 39.6 mW/$m^{2}$), Effects of ECs	Frequency response (16 V output at 3 Hz), Response time (45/66 ms), Flexibility (curvature radius 7.085 cm), Sensitivity (740 mV/kPa pressure, 380 mV/kPa stretching, 21.16 mV bending, visually for each individual sensor), Stability (10,000 cycles), Performance comparison, Sensing range (0–20 kPa), Repeatability
[363]	COMSOL, MatLab, LabView	ADC bits (16), Sampling frequency (1 MS/s)	ARE (0.18%), MAE (0.0028–0.2272), MSE (0.0001–0.0771), MARE (0.42–10.35%)
[103]	C# custom software	ADC bits (16), Sampling frequency (48 kHz), cost (”low cost platform”)	Noise (23.6–49.3 mV/$\sqrt{\mathrm{Hz}}$), RE (0.7%), Repeatability (0.02–0.14%), Sensitivity (3.4–13,478 mΩ/LSB), Resolution (3.6 ppm)
[346]	Python	Cost (“low-cost fiber-optic”)	Performance comparison, Repeatability (std = 0.98 mm)
[331]	COMSOL	Power consumption (generated power density = 1.3 W/$m^{2}$)	Stability (25,000 cycles), Sensitivity (7.5 V/Pa), CD (0.99)
[102]	COMSOL	Power consumption (1.2 mW generated in open circuit), Cost (“low-cost materials”)	MRE (<0.7%), MAE (1.31–2.09 ppm), ARE (1.43%), Repeatability (visually), Stability (10,000 s), Sensitivity (visually for each individual sensor)
[330]	Python, Arduino, COMSOL	Power consumption (max. power density generated = 0.853 mW/$m^{2}$)	Frequency response, Stability (0.91 output factor after 75,600 cycles), Sensitivity (11.13–59.11 N/V), Range (30–885 N)
[248]	MatLab	Power consumption (max. output voltage generated = 9.44 V), Spatial resolution (5 mm)	Sensitivity (8.30 mV/kPa), Response time (5 ms), Repeatability (std of sensitivity = 0.09–0.20 mV/kPa), CD (0.991–0.999), Stability (10,000 cycles), Performance comparison
[329]	LabView	Cost (fabrication process), Effects of ECs, Power consumption (450 mW PCB consumption), Sampling frequency (1 kHz)	Accuracy (98.1%), Resolution (0.1N), Sensing range (0–21.5 N), Stability (25,200 cycles), CD (0.96), Repeatability (0.03–0.07 V amplitude), Crosstalk (<3.15%)
[93]	Arduino	ADC bits (10), Sampling frequency (15 kS/s), Cost (graphite paint is cost-effective)	Flexibility, LOD (16.3 N–4290 kPa), Resolution (0.02 N in capacitive mode/0.9 N in resistive mode), Response time (29 ms in capacitive mode/31 ms in resistive mode), Repeatability (1000 cycles), Hysteresis (7.54% in capacitive mode/8.48% in resistive mode), Sensitivity (1.835 pF/N in capacitive mode and 0.465–0.018 V/N in resistive mode)
[328]	-	Cost (“low-cost”), Effects of ECs	Flexibility, Frequency response, Stability (5500 cycles), Response time (100 ms), Accuracy (100%)
[242]	-	Power consumption (visually, generated)	Sensitivity (average = 0.64 mV/Pa, visually for some individual sensor), Repeatability (std of sensitivity of some individual sensors = 0.06 mV/Pa), BW (10–10,000 Hz), Linearity error (0.3%), Performance comparison, Frequency response
[301]	ABAQUS	Sampling frequency (10 MHz)	Visual evaluation, Error (0–2 mm), Frequency response
[107]	Spice	Power consumption (“increased power consumption due to (…) amplifiers’’), Cost (“increased fabrication costs due to (…) amplifiers’’)	Performance comparison, ARE (0.1%), Response time (2.2 µs for Type II)
[163]	Arduino, COMSOL	Spatial resolution (7 mm), Effects of ECs	LOD (0.8 Pa), Response time (6 ms), Accuracy (100% for classification), Sensing range (0–80 kPa capacitive mode, 0–8.78 kPa triboelectric mode), Sensitivity (7.88 ${\mathrm{kPa}}^{-1}$ triboelectric mode, 17% ${\mathrm{kPa}}^{-1}$ capacitive mode), SNR (15.6 dB), CD (0.995), Repeatability (std = 4.4% triboelectric, 3.9% capacitive), Flexibility, Performance comparison
[255]	-	Sampling frequency (100 Hz), Response time (35 ms)	Flexibility, Sensitivity (average = 4.844 mV/kPa, individual sensors in range [4.47, 5.22] mV/kPa), Accuracy (93.65%), Stability (1500 cycles), Frequency response, CD (0.980–0.998), Linearity error (RMSE = 19.20–81.65 mV), Specificity (84.38%), Performance comparison, Repeatability (std of sensitivity of individual sensors in range [0.09, 0.39] mV/kPa)
[319]	LabView	Power consumption (3.9 µW generated), Cost (“low-cost fabrication”), Effects of ECs	Frequency response, Stability (300 cycles), Sensitivity (6.1–6.9 mV/kPa)
[285]	-	Power consumption (visually, generated)	Sensitivity (47.45 mV/N), Frequency response, Performance comparison, Crosstalk (“negligible serial interference’’)
[87]	-	Cost (“no need for costly techniques”),	Flexibility, Sensitivity (236.5 ${\mathrm{kPa}}^{-1}$), Sensing range (0–260 kPa), Response time (100 ms), Stability (10,000 cycles)
[149]	-	Sampling frequency (90 Hz)	Flexibility (stretchability > 30%), Sensing range (265 kPa), Sensitivity (0.15–5.4 ${\mathrm{kPa}}^{-1}$), LOD (2 Pa), Response time (44 ms), Stability (1000 cycles), Accuracy (97.7%), Performance comparison, Crosstalk (8.53%)
[86]	-	Effects of ECs	Sensing range (up to 1 Mpa), Sensitivity (0.279 ${\mathrm{kPa}}^{-1}$), CD (0.959), Response time (0.72), Stability (20 cycles)
[280]	-	Sampling frequency (10 MHz)	Absolute Error (<3.02 mm), Performance comparison, Visual evaluation
[85]	Python	Sampling frequency (50 Hz), Cost (“cost effective fabrication process”), Spatial resolution (1.5 mm)	Resolution (temporal = 7 ms), Flexibility, Accuracy (96.1–99.0%), Performance comparison, Sensitivity (1.661 ${\mathrm{kPa}}^{-1}$), Response time (3 ms), Frequency response (error < 2%), Stability (15,000 cycles), Drift (due to ECs)
[281]	COMSOL	Power consumption (2.341 mW generated)	Performance comparison, Frequency response, Visual evaluation
[84]	COMSOL, Verilog	Sampling frequency (10.2 ms), Power consumption (300 µA, 540 W), Effects of ECs, ADC bits (12), ENOB (11.5)	Sensing range (100 kPa–100 Mpa), Performance comparison, Noise (80 nV/$\sqrt{\mathrm{Hz}}$), Resolution (11 bit, 48 kPa/LSB), Visual evaluation, Sensitivity (12 mV/MPa)
[83]	-	-	Sensitivity (888.79 ${\mathrm{kPa}}^{-1}$), Flexibility, Accuracy (94.08%), CD (0.9861, fitting of SNR vs. pressure), SNR (visually), Performance comparison, LOD (0.4608 Pa–100 kPa), Response time (66/69 ms)
[82]	C++ custom software	Effects of ECs	Linearity error (4.4%), Response time (4 ms), Hysteresis (12.2%), Repeatability (5.2%), Flexibility, Sensitivity (0.0412 ${\mathrm{kPa}}^{-1}$), CD (0.99)
[81]	-	Sampling frequency (10), Cost (“affordable”), ADC bits (10)	Repeatability (11 ± 3 Ω, 219.2 ± 0.5 kΩ), Visual evaluation, Flexibility
[80]	Arduino	Cost (0.06$ CAD per sensor), Power consumption (1 mW), Sampling frequency (100 Hz)	NRMSE (6.31–8.22%), CD (0.767–0.925), Repeatability (std of gauge factor = 1.035, std of resistance of 10 sensors = 165.1 kΩ), Performance comparison, Hysteresis (mecanical = 0.023%, electrical = −0.261%), Sensitivity (gauge factor = 4.369)
[141]	Python	-	MARE (3.57%), Performance comparison
[108]	-	ADC bits (8), Sampling frequency (75 fps), Cost (“cost effective”), Power consumption (“increased power consumption”)	Performance comparison, Visual evaluation
[101]	-	Cost (“cost-efficient’’)	LOD (6 ppb for device 1, 4.6 ppb for device 2), Response time (2.4 for device 1, 2.8 for device 2), Repeatability (variations of 7.2% for device 1, 8.2% for device 2 in 15 days), Relative error (4%), CD (0.91), Performance comparison
[79]	-	Effects of ECs	ARE (0.3%), Linearity error (<1 LSB), Performance comparison, Absolute error (<29.4 Ω), Drift (avoided by using CTIA)
[138]	NI Multisim, Arduino	Sampling frequency, ADC bits (10)	ARE (0.31%), Performance comparison
[340]	-	Effects of ECs (“temperature/humidity variations change cavity length’’)	MAE (0.01–0.030), MARE (1.2–4%), Frequency response, Visual evaluation
[189]	-	-	Noise (shielding), Relative error (<0.72%), Performance comparison, Correlation coefficient (0.74–0.97), Noise (“Gaussian noise is added”)
[193]	-	Cost (“low-cost”), Power consumption (“low-power consumption”), Effects of ECs	Relative error (<21.3% for ethanol, <20.6% for methanol), Repeatability, CD (0.94)
[186]	-	Sampling frequency (43 Hz), Power consumption (20 mW)	Sensing range (1.5–43 kPa), Repeatability (std = 3.5%), Accuracy (88%), LOD (1.5), CD (0.99), Drift (visually), Crosstalk (“no observed’’)
[344]	-	Cost (“this method is cost-effective’’)	Absolute error (7.6–17.8 cm), Sensitivity (visually for one individual sensor)
[342]	-	Sampling frequency (5 MHz), ADC bits (199)	BW (45 kHz), Frequency response, SNR (32 dB), Performance comparison, Sensitivity (7.3 $\times \;10^{-6}$ ${\mathrm{rad}}^{2}$/Hz), Crosstalk (average = 29 dB)
[238]	-	Effects of ECs (optimal temperature operation = 150 °C)	Accuracy (100%), LOD (14.5 $\times \;10^{-9}$) SNR (3 dB, at LOD), Drift (“negligible baseline drift’’)
[224]	-	-	CD (0.996), Sensitivity (14.04–55.37 ${\mathrm{kPa}}^{-1}$, visually for some individual sensors), Response time (64/331 ms), Sensing range (0–15.75 kPa), Crosstalk (“very low’’)
[237]	-	Cost (“MIPs have low cost”)	LOD (1.4fM), Repeatability (std of 3 sensors = 3.9% for ampicillin, 6.1% for kanamycin, 6.2% for amoxicillin, visually), CD (0.991–0.998), Sensing range (1 $\times \;10^{-14}$–1 $\times \;10^{-8}$ M), Drift (“very slight drift’’, std = 0.51%)
[236]	-	-	Sensing range (50–150 ppb), Stability (30 days), LOD (20 ppb), Accuracy (83.6–99.3%)
[52]	-	Spatial resolution (50 pixels/${\mathrm{cm}}^{2}$), Effects of ECs	Sensitivity (pressure = 5.29 ${\mathrm{kPa}}^{-1}$, temperature = 0.55 $\times \;10^{-2}$ °$C^{-1}$), Sensing range (0.25–16 kPa, 20–70 °C), Stability (5000 cycles), Performance comparison
[240]	-	Sampling frequency (0.5 s)	Sensing range (1–100 µM), LOD (1 nM), Selectivity (1 nM–100 µM), Performance comparison, Sensitivity (0.037–0.343/log(M)), Repeatability
[215]	LabView	Spatial resolution (1 mm), Sampling frequency (31.25 kHz), Cost (low-cost commercial coils), ADC bits (14), Power consumption (2.748 W)	SNR (26.5 dB), Sensitivity (visually), Visual evaluation
[214]	MatLab	Cost (cost-effectiveness), ADC bits (28),	Accuracy (test 94%), Repeatability (2.9 $\times \;10^{-4}$, 1.186 $\times \;10^{-2}$ MHz), Classification sensitivity (>96.1%), Specificity (>99.5%), Response time (50 ms), Frequency response
[212]	-	Sampling frequency (50 ms)	CD (0.9953), Sensitivity (4.789 pH/Pa), Flexibility (operation bent over a curved rod), Repeatability, Stability (100 cycles), Response time (visually)
[194]	COMSOL	-	Correlation coefficient (>0.991 between capacitances), MARE (individual values in range 0.39–0.96%), Sensitivity (visually)
[336]	-	Sampling frequency (40 kHz)	Sensitivity (average = −135 dB, visually for each individual sensor), Sensing range (10–2032 Hz), Repeatability (std of sensitivity = 0.4 dB), Correlation coefficient (0.987), Performance comparison, Frequency response, Noise, Drift (phase drift < −1.49 $\times \;10^{-5}$)
[146]	-	Cost (“cost-effective inkjet printing’’), Power consumption (“low power consumption”)	Sensitivity (0.32%/kPa), Frequency response, Repeatability, Crosstalk (“minimal mutual interference’’)
[245]	COMSOL	Spatial resolution (6 mm), Effects of ECs (between 20–70 °C, “good temperature stability”)	Sensitivity (15.08 mV/kPa), Flexibility (bending radius of 4.23 mm), Accuracy (98.18%), Response time (5 ms), Sensing range (0–183.91 kPa), Frequency response, Stability (10,000 cycles)
[243]	COMSOL, MatLab, LabView	Power consumption (“low power consumption’’)	Sensitivity (7mV per 100 Pa, visually for each individual sensor), Stability/Repeatability (10,000 cycles), Flexibility, SNR (>40 dB)
[77]	-	Effects of ECs, Cost (affordable materials)	Sensing range (0–13.3 N), Classification sensitivity (0.9680), Correlation coefficient (−58.7%)
[100]	Arduino	Cost (“low cost sensors”), Sampling frequency (2 S/s)	RMSE ([x, y] = [5.3, 5.2] in), Performance comparison
[190]	Arduino	-	Accuracy (>85%)
[358]	Raspberry Pi	-	Error (visua), Performance comparison, Sensing range (up to 4 mm), Accuracy (0.1 mm)
[244]	-	ADC bits (12), Spatial resolution (<1 µm)	LOD (mN), Flexibility, Hysteresis (“hysteresis free’’), Performance comparison, Repeatability (20 volunteers), Sensitivity (visually for each individual sensor), Crosstalk (eliminated by structure)
[144]	-	Cost (low-cost materials)	Sensitivity (0.175%), Hysteresis (8.25%), Response time (0.15 s), Sensing range (0–80 kPa), Repeatability (5% FSO, visually), RMSE (1.089), CD (0.99), Stability (1000 cycles), Performance comparison, Flexibility
[147]	COMSOL	Sampling frequency (1kHz)	Sensitivity (2.2 ${\mathrm{Mpa}}^{-1}$, visually for some individual sensors), Resolution (255 Pa, 1.9 µm), LOD (15 mm), Noise (shielding), Performance comparison
[185]	COMSOL, Python	Cost (“cost effective”)	Flexibility, Sensitivity (0.141 ${\mathrm{kPa}}^{-1}$), Sensing range (0–190 kPa), Response time (90 ms), Accuracy (95.14%), Stability (7000 s), Repeatability (7000 s), LOD (60 Pa), Performance comparison, Crosstalk (eliminated by structure)
[184]	-	Spatial resolution (2 mm), Sampling frequency (1 kHz), ADC bits (24), Power consumption (“low-power consumption”)	Accuracy (0.01N, 93%), Performance comparison, Noise (shielding), Sensing range (up to 2 N)
[182]	Python	Spatial resolution (32.7 dpi), Sampling frequency (100 Hz)	Flexibility, Accuracy (98%), Response time (1.5 s),
[235]	-	Power consumption (reduced energy consumption at $V_{G}$ = 0), Effects of ECs (RH = 0.4%)	Accuracy (97.51%), LOD (80 ppb), Stability (10 signals, 20 days each), Performance comparison, Sensitivity/Repeatability (“better sensitivity and repeatability’’), Stability (10 signals, MARE = 5.24%)
[229]	ANSYS	Cost (“low-cost processes’’)	Sensing range (1–50 W/$m^{2}$), Sensitivity (1.6 A/W), Performance comparison, Repeatability (visually)
[353]	-	Spatial resolution, Cost (“cost-effective’’)	Resolution (energy resolution = 7.18–10.34% for the closest channel to the hotspot), CD (0.9984), Crosstalk (eliminated by structure), Sensitivity (3.20 ADC number/keV)
[169]	-	-	Flexibility, Sensitivity (150–292.8% resistance under 50% strain), Hysteresis (visually), Repeatability (visually)
[213]	MatLab	Cost (“cost-effective’’)	Accuracy (97.3–98.7%), Sensitivity (>92%), Specificity (>99.7%), Repeatability (std of sensitivity < 0.85%, std of specificity = 0.09%), Drift (<0.01% in 7 days), Crosstalk (eliminated by structure)
[199]	ConventorWare	ADC bits (14), Sampling frequency (100 kHZ)	CD (0.96), Sensitivitiy (151 fF/pH, 94 fF/$\log_{10}$[DNA]), Repeatability (std = 6.3 fF), Performance comparison, Sensing range (10 aM–100 pM)
[209]	-	-	Sensitivity (visually), Performance comparison
[174]	-	-	Flexibility (stretchability = 50%), Resolution (“high-resolution’’), Sensitivity (gauge factor = 0.9), Stability (2100 cycles), Response time (20 ms), BW (100–1M Hz), Frequency response, Repeatability, CD (0.999), Sensing range (up to 50% strain)
[173]	-	Effects of ECs	Resolution (kPa), Frequency response, Hysteresis (visually), Sensing range (up to 10 Mpa), Drift (worse due to parasitic capacitances), Sensitivity (visually for each individual sensor)
[160]	ANSYS, SolidWorks	Cost (“low-cost’’)	Flexibility, Sensitivity (0.035–0.76 ${\mathrm{kPa}}^{-1}$), Response time (50 ms), Sensing range (0–30 kPa), LOD (2 Pa), CD (0.97–1), Performance comparison, Frequency response, Stability (200 cycles), Crosstalk (eliminated by structure)
[338]	-	Sampling frequency	Noise (floor = −100 dB ref rad/$\sqrt{\mathrm{Hz}}$), Sensing range/BW (10–20k Hz), Frequency response, Correlation coefficient (>0.99), ARE (3%)
[198]	Python	Sampling frequency (43 fps)	Sensitivity (6.896 codes/fF), Noise (cancelated), Performance comparison, Drift (1.6 mV in 40 min)
[161]	LabView, Arduino, Python	-	Sensitivity (0.001–0.18 ${\mathrm{kPa}}^{-1}$ for the whole array, visually for each individual sensor), Hysteresis (<16%), Response time (0.8–2.2/1.0–3.1 s), Sensing range (0–390 kPa), Repeatability (std of sensitivity = 0.0005–0.07 ${\mathrm{kPa}}^{-1}$)
[113]	-	Effects of ECs, Power consumption (“low power consumption’’)	Relative error (visually), Flexibility, Sensing range (1–200 Ω), Performance comparison, Crosstalk (compensated), Drift (temperature drift in OAs)
[137]	-	Power consumption	Flexibility, Sensitivity (0.15–0.6 ${\mathrm{Pa}}^{-1}$), Sensing range (0–3.3 kPa), Response time (70 ms), Performance comparison, Repeatability, Stability (6000 cycles), Crosstalk (“sensing without any crosstalk’’)
[116]	-	Sampling frequency (100 Hz), Spatial resolution (1 mm)	Hysteresis (visually) Crosstalk (compensated), Sensing range (0.04–1.4 MPa), Performance comparison, Sensitivity (visually), Crosstalk (eliminated by structure)
[8]	Raspberry Pi	ADC bits (24)	Flexibility, Sensing range (22.08 ± 0.04 kΩ), SNR (230–650), RMSE (2.3–12.7%)
[134]	MatLab, Simulink	ADC bits (8–16)	ARE (<0.3%), Sensing range (1.71–217 kΩ), RMSE (%, visually), Crosstalk (compensated)
[356]	-	-	Visual evaluation, Accuracy (100%), Sensitivity (“high sensitivity’’)
[112]	-	ADC bits (16)	Sensing range (100k–1M Ω), ARE (<2%), Crosstalk (compensated)
[361]	Arduino, Python	Sampling frequency (>90 Hz)	MAE (8.33°), Absolute error (individual sensors in range [2°, 18°]), Flexibility, Sensing range (1.31–3.38 mm), Repeatability (visually), Error (±20°), Noise (cancelated by software), Flexibility (bending angle > 180°)
[139]	Python	ADC bits (16)	MARE (0.018–1.131%), ARE (0.013–0.249%), RMSE (0.127–0.216%), Sensing range (100–300M Ω), Noise (added to the experiments), Performance comparison, Repeatability (10 experiments), Crosstalk (compensated)
[269]	FEA	Power consumption (7.64 peak-to-peak voltage generated, 200–300 mW consumption)	Noise (phase noise = −119 dBc/Hz, noise floor = −151 dBc/Hz), Resolution (0.024 Hz, 15 pϵ), Performance comparison, Sensitivity (1.6 kHz/µm)
[305]	COMSOL	Sampling frequency (250 Hz), Power consumption (max. > 300 mV generated open circuit)	Sensitivity (61.8–150.63 mV/Pa 39.22 ${\mathrm{mVPa}}^{-1}$${\mathrm{cm}}^{2}$. visually for each individual channel), BW (0.1–3 kHz frequency range), SNR (60 dB), Flexibility (curvature radius = 2.5 cm), Performance comparison, Stability (20 min at sound power = 94 dB)
[304]	-	Spatial resolution (1 × 1 ${\mathrm{mm}}^{2}$)	Correlation coefficient (2D map), Frequency response
[271]	COMSOL	Cost (“low-cost strategy”), Spatial resolution (11.11 sensors/${\mathrm{cm}}^{2}$)	Sensitivity (447.82 mV/kPa, visually for individual sensors 1 to 5), Flexibility (“wrist bending motion”), LOD (3.60 Pa), Performance comparison, Frequency response, Repeatability, Crosstalk (“minimal’’)
[218]	-	-	Sensitivity (31.43 mA/W, visually for each individual sensor), Flexibility (tested bent over surfaces), Stability (1000 cycles), CD (0.94), BW (100 Hz), Performance comparison
[143]	FEA	Cost (“low-cost”), Power consumption (“low-power consumption”)	Sensitivity (5.53 ${\mathrm{kPa}}^{-1}$), Sensing range (0–0.444 kPa), Response time (70 ms), LOD (0.24 Pa)
[145]	FEA	-	Flexibility, Sensitivity (0.07 ${\mathrm{kPa}}^{-1}$), Repeatability (1500 cycles), Performance comparison, Sensing range (0–80 kPa), Response time (100/110 ms), CD (0.98)
[201]	Python	Cost (“cost-efficient in preparation”),	Sensitivity (avg = 56.4 mV/pH, 16 individual sensors = [53–58] mV/pH), Repeatability (1.5 mV/pH in sensitivity)
[164]	MatLab	Cost (“inexpensive solution”)	Accuracy (83.3–100%), Response time (0.16–0.89 s), Sensing range (up to 2 MPa)
[327]	-	Cost (“cost effective” fabrication system), Effects of ECs	Stability (10,000 cycles), Frequency response, CD (0.996), Sensitivity (7.78 mV/kPa), Sensing range (40–200 kPa), LOD (0.4 kPa)
[360]	LabView	Cost (visually)	MAE ([x, y] = [43.9, 47.5] µm), Repeatability (std of MAE [x, y] = [27.8, 28.0] µm, visually), Noise (0.15 mT), Sensitivity (visually), Performance comparison, Resolution (0.1 mT), Sensing range (−200 to 200 mT)
[109]	Proteus, C# custom software	ADC bits (12)	ARE (0.3%), Visual evaluation, Performance comparison
[78]	MatLab	Cost (low-cost), ADC bits (12), Sampling frequency (10 Hz),	MARE (0.31% and 0.71%), CV, Repeatability (std = 0.11% and 0.15%), LOD (0.5–1.5 times nominal value), Performance comparison, Hysteresis, Accuracy (99.45% and 99.8%), Flexibility
[92]	COMSOL	Sampling frequency, Power consumption	Sensitivity (gauge factor = 1.2–6.4 in capacitive, 36–644 in resistive), Flexibility, Sensing range (1 $\times \;10^{-4}$–16 kPa, 0–41.25% ϵ), Stability (6000 cycles), Repeatability (“good repeatability”), Frequency response, Resolution (“sufficient resolution”), LOD (0.1 Pa)
[68]	-	Cost	Sensitivity (0.6–5.35 ${\mathrm{kPa}}^{-1}$), Sensing range (1.1–266 kPa), Accuracy (98.2%), CD (0.973–0.977), Stability (5000 cycles), Repeatability (“high repeatability’’), Performance comparison, Response time (300/180 ms), Flexibility, LOD (0.35 g)
[254]	Arduino	Effects of ECs (visually)	Response time (2 ms), Sensitivity (8.9V/1.5 MPa), Frequency response, Repeatability (visually), Noise (removed by filters), Stability (repeated experiments)
[253]	-	Power consumption (visually, generated)	Sensitivity (0.2 V/N, LOD (20 g), Performance comparison, Stability (10,000 cycles), Noise (3RMS = 0.048 V), Crosstalk (eliminated by structure)
[257]	-	ADC bits (12), Sampling frequency (125 Hz)	Accuracy (up to 98.4%), Sensing range (0–206 Pa), Relative error (<5%), Repeatability (visually), CD, Correlation coefficient (>0.2), Sensitivity (16.019 mV/kPa), Frequency response, Performance comparison, Flexibility, BW (0.5–100 Hz), Drift (baseline drift)
[256]	-	Sampling frequency (1000 Hz)	Sensitivity (visually for both single channels and whole array), Sensing range (2.5–20 Hz), Performance comparison, Repeatability (visually)
[364]	COMSOL	ADC bits (24)	Noise (16–470 pT/$\sqrt{\mathrm{Hz}}$), Sensitivity (visually for both single sensor and whole array), Frequency response, Sensing range (100–200 nT)
[314]	Mathematica	Power consumption (840 µV generated)	Resolution (0.5 °C), Sensing range (0–10 cm), Linearity error (<1%), SNR (400%), Flexibility (180° bending degree), Performance comparison, Response time (128 ms)
[247]	Python, Arduino, Raspberry Pi, Smartphone App	Power consumption (visually, generated)	Sensitivity (262 mV/kPa), Sensing range (1–5 kPa), Performance comparison, Flexibility, Frequency response, Stability (test of several days)
[62]	Arduino	Power consumption (50 mW)	Sensitivity (temperature = 0.8%/°C, pressure = 4.5% for 100 kPa, light = 2%/20 µW/${\mathrm{cm}}^{2}$), Repeatability (deviation = 8–10% after bending), Performance comparison, Flexibility (7.5 mm bending radius), Stability (4000 cycles)
[192]	SCADA, WinCC OA	Effects of ECs (“low-temperature dependence’’)	Performance comparison, Sensing range (100–500 pF), Relative error (<2.5%), Sensitivity (visually), Stability (“high stability’’)
[114]	-	Cost (“low-cost’’)	Stability (16 days), ARE (<0.1%), Response time (>60 s), Flexibility, Sensing range (10–10k Ω), Crosstalk (compensated)
[140]	Python	Sampling frequency (10 Hz), ADC bits (12)	Performance comparison, Noise (±1.24 LSB), Repeatability (1000 experiments), Sensing range (1 $\times \;10^{-5}$ to 1 $\times \;10^{-2}$ S), Crosstalk (compensated), ARE (<0.75%)
[315]	COMSOL, LabView	Spatial resolution (8 dpi)	Sensitivity (0.016–0.06 V/kPa), Frequency response, Flexibility (5000 bending cycles at 180°), Stability (6000 cycles), Crosstalk (NECT = 0.18)
[317]	COMSOL	Power consumption (generated power density = 0.76 mW/$m^{2}$)	Visual evaluation, CD (0.991), Relative error (<7.61% in voltage, <12.1% in current)
[322]	-	Power consumption (visually, generated)	Visual evaluation, Hysteresis (“without signal hysteresis’’), Noise (“reduced through grounding’’), Frequency response
[318]	Raspberry Pi	Effects of ECs (“temperature and humidity were controlled’’), Power consumption (generated charge density = 43.4 µC/$m^{2}$)	Sensitivity (0.0032–0.0406 µ${\mathrm{Cm}}^{-2}$${\mathrm{kPa}}^{-1}$ in individual sensors), Sensing range (0–425 kPa), Stability (2000 cycles), Frequency response, Hysteresis (visually), Response time (0.36 s), Repeatability (visually)
[320]	-	Power consumption (960.4 µW generated)	Stability (32,000 cycles), Frequency response
[268]	LabView	Sampling frequency (50 kS/s), ADC bits (16)	Sensitivity (35 mV/kPa), Performance comparison
[228]	-	Spatial resolution (505 ppi), Sampling frequency (34 Hz)	Performance comparison, Sensitivity (visually for one individual pixel), Sensing range (300–1100 nm), SNR (visually)
[227]	-	Spatial resolution (6250 transistors/${\mathrm{cm}}^{2}$)	Visual evaluation, Flexibility, Repeatability (visually)
[366]	Python	Spatial resolution	RMSE (5.0 mmHg), MAE (0.2 mmHg), Correlation coefficient (0.8), Performance comparison, Flexibility, Repeatability (std of correlation coefficient = 0.08 mmHg, std of RMSE = 0.5 mmHg), Frequency response
[65]	-	Cost (“cost-effective’’)	Flexibility, Stability (10 cycles), Sensing range (0–30 kPa), Sensitivity (0.05–0.13 ${\mathrm{kPa}}^{-1}$), Hysteresis (“low’’), Creep (“due to the material’’)
[152]	LabView	-	MARE (6.8–9.1%), Absolute error (1.5 mm), CD (0.999), Sensitivity (Ag electrode = 1.91–3.82 ${\mathrm{kN}}^{-1}$, Cu electrode = 2.77 ${\mathrm{kN}}^{-1}$), Repeatability (std of sensitivity, Ag electrode = 0.01–0.08 ${\mathrm{kN}}^{-1}$, Cu electrode = 0.04 ${\mathrm{kN}}^{-1}$), Sensing range (80 mm)
[71]	Mathematica	Effects of ECs (“showed same signals’’)	Sensitivity (visually), Hysteresis (“negligible’’), Sensing range (0.41–200 kPa), Resolution, Stability (10,000 cycles)
[94]	-	TCR (0.0216 $K^{-1}$), Cost (“low-cost’’)	Sensitivity (temperature = −0.04 to −0.025 °$C^{-1}$, pressure = 13–140 ${\mathrm{kPa}}^{-1}$), Sensing range (temperature = 25–55 °C, pressure = 0–10 kPa), Repeatability/Stability (10,000 cycles), Performance comparison, Flexibility (tested in bended states), Crosstalk (eliminated by structure)
[205]	MatLab	Cost (“cost-effiicient’’), Effects of ECs, Sampling frequency	Sensing range (0–100% concentration), Performance comparison, CD (0.9699–0.9927), Repeatability (std = 9.3–10 µA due to mismatch), Noise (visually)
[311]	COMSOL	Effects of ECs (“are affected by ambient temperature and humidity’’)	Visual evaluation, Frequency response
[347]	-	Cost (“TDM is cost-effective”)	Noise (2 µrad/$\sqrt{\mathrm{Hz}}$), Performance comparison, Frequency response, SNR (visually), Drift (due to ECs), Crosstalk (74–76 dB)
[292]	COMSOL	Cost (“low-cost’’)	Performance comparison, BW/Sensing range (100–700 kHz), Frequency response
[4]	ABAQUS	Sampling frequency (100 MHz), ADC bits (8)	MARE (2.1%), SNR (“high’’), Frequency response
[2]	FEA	Effects of ECs, TCF (−20.4 ppm/°C), Sampling frequency (1 Hz)	Accuracy (87.5–95.8%), Sensitivity (visually), Response time, LOD (25.1–111.4 ppm for ethanol, <7.4 ppm for E-2-hexenal), Selectivity, CD (frequency/temperature = 0.99), Sensing range (100–800 ppm), Noise (“reduced by specific electrodes’’), Repeatability
[288]	LabView	-	Sensitivity (8 pC/N), Sensing range (tidal volume = [0.6–0.71] L, minute ventilation = [7–8.1] $\min^{-1}$), ARE (8.5%), Response time (0.6 s), Flexibility
[354]	-	Cost	-
[351]	-	-	-
[299]	-	Sampling frequency (2 MHz)	Absolute error (distance = 0–2.4 cm, direction = 1°), Visual evaluation
[162]	-	Spatial resolution (1.5 mm)	Flexibility, Sensitivity (single sensor = 28.8%/N, array = 0.065%/kPa), Frequency response, Response time (visually), Repeatability (various cycles), Crosstalk (“the independence is good’’)
[258]	-	Sampling frequency (1 MHz)	ICC (0.868–0.996), Stability/CV (0.130–0.195), CD (0.99), Sensitivity (11.88 pC/N), Frequency response, Response time (11–13/10–12 ms)
[1]	-	Cost (“low-cost interrogation”), Effects of ECs, ADC bits (16), Sampling frequency (5 Hz)	Sensitivity (visually for each individual sensor), SNR (31.27–35.07 dB), Performance comparison, BW (20 MHz), Frequency response, Response time, Noise (NEP = 0.12–0.19 kPa), Drift (wavelength drift)
[334]	MatLab	Cost, Effects of ECs (temperature sensitivity = 0.1854 dB/°C)	Sensitivity (−38.40 to 24.08 dB/$m^{-1}$), CD (0.99), Resolution, Sensing range
[282]	-	Power consumption (visually, generated)	Hysteresis (visually), Performance comparison, Flexibility
[355]	-	Cost (“cost-effective manufacturing process’’)	CD (0.999), LOD (38 fg/mL), Selectivity (visually), Performance comparison, CV (12%), Correlation coefficient (0.999), Repeatability (visually)
[300]	ABAQUS	Cost (“relatively high’’)	RMSE (0.3762), CD (0.9932), Accuracy (87.5–100%)
[70]	-	-	Sensitivity, (0.58 ${\mathrm{kPa}}^{-1}$) Response time (0.36 s), Performance comparison, Stability (5 cycles)
[76]	-	Cost (“low-cost’’)	Sensitivity (164.5 ${\mathrm{kPa}}^{-1}$), Hysteresis (11%), Performance comparison, Stability (<4.7% variation in 1500 cycles), Repeatability (visually), Flexibility, Response time (19/13 ms)
[56]	Smartphone app	-	Response time (0.11/0.08 s), Sensitivity (0.47–1.61 ${\mathrm{kPa}}^{-1}$, visually for various individual sensors), Hysteresis (visually), Accuracy (96.4%), Absolute error (20 Ω), Sensing range (18–50 kPa), Stability/Repeatability (1000 cycles of 5000 s each)
[151]	FEA	Cost, Power consumption (31.8 mW), Effects of ECs, TCS (−0.4 fF/°C), Sampling frequency (1.8 kHz), ADC bits (14)	Sensitivity (5.8 fF/kPa), Performance comparison, Noise (6 Pa), Resolution (6 Pa), Sensing range (0.006–3.5 kPa), Frequency response
[168]	Wilcom Deco Studio, MatLab, Arduino	Sampling frequency (90.9 Hz), ADC bits (24)	Sensing range (2–30 kPa), Hysteresis (visually), Performance comparison, Repeatability (visually)
[97]	NI Multisim	TCR (commercial sensors)	ARE (1.8%), Relative error (visually), Absolute error (visually), Stability (MSE = 1 $\times \;10^{-7}$), Sensing range (26–500 °C), Performance comparison, Sensitivity (commercial sensors), Crosstalk (compensated)
[57]	-	Cost (“low-cost’’)	Sensitivity (0.54 ${\mathrm{kPa}}^{-1}$), Flexibility (80% elasticity), Sensing range (0.1–101 kPa), Response time (60 ms), Stability (5000 cycles), Performance comparison, LOD (102 Pa), Drift (stable for 10 h)
[91]	-	-	Sensitivity (visually for each individual sensor), Relative error (<19.2% in speed), Error (<9° in direction), Sensing range (orientation = [0, 360]°, velocity = [0–10.2] m/s), Performance comparison, Repeatability (visually)
[51]	Arduino	Cost (low-cost materials)	RMSE (2.17–7.49 kPa), Visual evaluation, Hysteresis
[290]	-	-	BW (0.2–4 kHz), Frequency response, Noise (filtered)
[191]	-	Spatial resolution (13 dpi)	Sensitivity (0.43 ${\mathrm{kPa}}^{-1}$), LOD (3.4 Pa), Response time (33/33 ms), Performance comparison, Sensing range (0–50 kPa), Hysteresis (“response with little hysteresis”), Stability (1000 cycles), Crosstalk (“less impact’’)
[316]	LabView, COMSOL	-	Visual evaluation, Sensing range (visually), Performance comparison, Crosstalk (eliminated by structure)
[176]	Python	Effects of ECs (visually)	Sensitivity (0.022–2.6 ${\mathrm{kPa}}^{-1}$), Sensing range (0–25 kPa), Repeatability (std = 3.0–9.5%, visually), Hysteresis (visually), Stability (10,000 cycles), Performance comparison, Flexibility, Resolution (<1 mm), Drift (“stability without drift”)
[352]	MatLab, ANSYS	Effects of ECs (compensated)	ARE (0.03–0.43%), Absolute error (2.8–5.8 $\times \;10^{-3}$ mm), Accuracy (5.8 $\times \;10^{-3}$ mm), Performance comparison, Sensing range, Flexibility
[150]	FEA	Power consumption (“low power consumption’’)	Sensitivity (2.51 ${\mathrm{kPa}}^{-1}$), Hysteresis (visually), LOD (2 Pa), Response time (84/117 ms), Sensing range, Flexibility, Repeatability-Stability (5000 cycles), Frequency response
[75]	SPICE, Python	ADC bits (12), Sampling frequency (1 MSPS), Power consumption, Cost (USD 545.00), Spatial resolution (4 sensels per ${\mathrm{cm}}^{2}$)	ARE (<0.1%), Response time (3 ms), Sensing range (1k–1M Ω), CD (−0.95), Crosstalk (compensated)
[55]	LabView, Arduino, Raspberry Pi	Cost (“cost-effective’’)	Sensitivity (0.067–0.3 ${\mathrm{kPa}}^{-1}$, normalized resistance/curvature = 1.5 cm), Performance comparison, Flexibility
[297]	-	Effects of ECs	Performance comparison, Absolute error (2.4 cm in distance, 4° in direction), Frequency response
[200]	Python	Cost (“low-cost in fabrication”)	Sensitivity (visually), Visual evaluation, Crosstalk (eliminated by structure)
[287]	-	Effects of ECs (tested from 20–50 °C)	Frequency response
[343]	-	Cost (sensor selected “based on cost consideration’’)	Sensitivity (visually for each individual sensor), Sensing range (20–300 kHz), Noise (visually), Frequency response, Visual evaluation
[204]	MatLab	Cost (“low-cost”), Effects of ECs (“insensitive to temperature and humidity”)	Sensitivity (0.015–5.15 $N^{-1}$), Performance comparison, Response time (60/80 ms), Flexibility, Stability (1000 twisting cycles, 5000 loading/unloading cycles)
[350]	-	Effects of ECs (removed)	Accuracy (90%), Repeatability (visually), Noise (3 rad)
[220]	Silvaco, Arduino	Effects of ECs (“studied by other researchers’’)	Sensing range (wavelength = 300–800 nm), Performance comparison, Frequency response, Sensitivity (responsivity = 0.65–0.85 A/W)
[246]	LabView	Cost (“cost-effective for large-scale manufacturing’’), Power consumption (visually, generated)	Sensitivity (7.7 mV/kPa), Response time (10 ms), Flexibility, Frequency response, Stability (80,000 cycles), Repeatability, Crosstalk (eliminated by structure)
[263]	COMSOL	Sampling frequency (100 Hz)	Visual evaluation, Repeatability (visually)
[120]	MatLab, NI Multisim	ADC bits (10–16)	Relative error (visually), Absolute error (visually), Sensing range (500–30k Ω), Crosstalk (compensated)
[178]	MatLab, Arduino	ENOB (21 bits)	MAE (1–5°), ARE (2.6%), Sensitivity (4.6 fF/N for normal forces, 1.4 fF/N for shear forces, individual sensors in range [26.1, 49.8] fF per 30° for angle measurement), Sensing range (0–15 N), SNR (10–15 dB), Performance comparison, Accuracy (1 $\times \;10^{-18}$ F), Repeatability (5 cyles in 4 different angles), Hysteresis (“small amount”), Flexibility, Stability (60 s), Crosstalk (9.1 fF), Resolution (250 mN)
[225]	-	-	Selectivity (visually), Repeatability (std = 2.5–3.5%), Stability (4 weeks, 3 sensors = 2, 2.5, 3%), CD (0.98–1), Sensing range (0.1–100 mM), Sensitivity (3 sensors = 213.875, 261.663, 275.877 µA/mM/${\mathrm{cm}}^{2}$), LOD (0.1, 0.4, 1.0 µM, 3 ions), Performance comparison
[88]	-	Sampling frequency (5 kHz), Power consumption (“low when no load is applied’’)	Sensitivity (visually for each individual sensor), CV (1.21% for forces, 2.05% for voltages), ARE (<5.29%), Response time (62 ms), Sensing range (shear forces [−11.8, 11.8] N, 0.28–2.19 MPa), Flexibility, Creep (“slow response’’), Repeatability (std < 2.05% for single sensor, std = [2.36–5.29]% for array)
[232]	-	-	Sensitivity (visually), LOD (0.10 µL), Performance comparison, Sensing range (2–12 pH) Repeatability/Selectivity (100 experiments), Stability (60 days)
[216]	-	-	Performance comparison, SNR (visually), BW (10 Hz), Linearity error (<3%), Frequency response, Sensitivity (13 nT), Sensing range (20.1 cm), Stability (“good stability’’), Noise (floor = 0.235 nT/${\mathrm{Hz}}^{1/2}$)
[233]	MatLab	Power consumption	Sensitivity (51.8 mV/pCl), Selectivity, Sensing range (1 $\times \;10^{-5}$–0.1 M), CD (0.990), Performance comparison, Stability (3 weeks/7 months), Response time (visually), Drift (5.0 mV/h)
[133]	MatLab, Simulink	Cost (“low-cost’’)	Absolute error (<0.005%), Sensing range (100–400 kΩ), Performance comparison, Crosstalk (compensated)
[362]	LabView	Cost (*£*1.90/sensor), Sampling frequency (100 Hz)	CD (0.9998), Resolution (20 mA), Performance comparison, Drift (75 mA)
[221]	-	Spatial resolution (400 µm)	Sensitivity (4.7 $\times \;10^{-5}$–1.13 $\times \;10^{-3}$ ${\mathrm{kPa}}^{-1}$, visually for all devices in the array), Performance comparison, Hysteresis (“negligible’’), Sensing range (200 Pa–5 MPa), Repeatability (std of sensitivity = 0.27 $\times \;10^{-3}$ ${\mathrm{kPa}}^{-1}$), Response time (50 ms), Stability (5100 cycles), Flexibility, Crosstalk (eliminated by structure)
[307]	The Unscrambler	Cost (cost-effective)	Specificity (96% for ammonia), Classification sensitivity (90%), Selectivity (100% for ammonia), Performance comparison, CD (0.96–0.998), Sensing range (0–0.01%), Repeatability (>90%), Noise (3–4 Hz), Drift (8–17 Hz), Stability (10 sorpotion-desorption cycles)
[249]	-	Spatial resolution (50 µm)	Sensitivity (visually), Visual evaluation
[117]	SPICE	Power consumption (visually)	ARE (0.0018% to 1.39%), Absolute error (50–300 Ω), Repeatability (10,000 tests), Performance comparison, Crosstalk (compensated)
[10]	-	Power consumption (“low power consumption’’)	Accuracy (83–99%), Sensing range (0–30 N), Sensitivity (62.5–76.9%/N), Resolution (0.05 N), Hysteresis (5.25–9.36%), Repeatability (2.36–2.62%), Crosstalk (eliminated by structure)
[111]	SPICE, MatLab	Effects of ECs (crosstalk evaluation in 10–80 °C)	ARE (visually), Sensing range (100–10M Ω, 10–80 °C), MSE (30 kΩ), Sensitivity (visually), Performance comparison, Crosstalk (compensated)
[156]	Arduino	Power consumption (16.93 V/MPa generated), Effects of ECs (operating temperature between 20–60 °C)	Sensitivity (37.54 ${\mathrm{MPa}}^{-1}$, 16.63 V ${\mathrm{Mpa}}^{-1}$), Repeatability (std of sensitivity = 1.488 ${\mathrm{MPa}}^{-1}$), Stability (15 days), Sensing range (0–0.1 MPa), Response time (60/45 ms), Performance comparison, Flexibility
[188]	-	Spatial resolution, Cost (“low-cost hardware”), Sampling frequency (100 fps)	Sensitivity (visually), LOD (3 $\times \;10^{-4}$ pF), Flexibility
[89]	-	-	Visual evaluation, Sensitivity (visually), Performance comparison
[167]	COMSOL	Effects of ECs, TCO (−0.05%/°C), TCS (0.63%/°C)	Sensitivity (5.62 fF/N), Sensing range (0–15 N), Repeatability (visually), Performance comparison, Drift (−3.79 fF/°C), Linearity error (<1%)hsie
[104]	-	Sampling frequency (63 fps)	Performance comparison
[312]	LabView	ADC bits (16)	Response time (visually), Visual evaluation, Sensitivity (visually), Performance comparison
[241]	LabView	-	Sensitivity (142 mV/0.8N at 1.8 Hz), Flexibility (bends 90°), Frequency response, LOD (0.2 N), Crosstalk (33%), Repeatability (950 cycles)
[273]	-	Sampling frequency (250 Hz)	ARE (9.14–10.86% in load speed, 5.5–6.25% load magnitude), Noise (added to the experiments), Sensing range (1/4–3/4 length of the beam)
[7]	LabView, SolidWorks, Arduino	Power consumption (“low power consumption’’)	LOD (36 ppm for methane, 40 ppm for ethanol), CD (0.90–0.96), Sensitivity (visually for each individual sensor and type of gas), Performance comparison, Flexibility
[158]	FEA	Cost (“cost-effectively fabricated”)	Sensitivity (0.103–6.583 ${\mathrm{kPa}}^{-1}$), Sensing range (up to 1 kPa), Response time (48/36 ms), LOD (3 Pa), Repeatability (5 cycles at 10, 100, and 1000 Pa), Performance comparison, Flexibility, Stability (10,000 cycles), Drift (“negligible within 500 cycles’’)
[154]	COMSOL, Python	Spatial resolution (420 µm), Sampling frequency, ADC bits (24)	Sensitivity (0.14–0.23 fF/kPa), Flexibility, Sensing range (0–300 kPa)
[283]	-	-	Sensitivity (0.78–1.88 mV/μϵ, variable between individual sensors), Flexibility, Frequency response
[324]	-	Power consumption (0.5 mA generated)	Sensitivity (5.3–53.7 mV/Pa), Sensing range (0.1–37.5 kPa), CD (0.991–0.996), Stability (30,000 cycles), LOD (0.1 kPa), Frequency response, Crosstalk (eliminated by structure)
[279]	ANSYS	Effects of ECs (visually)	Visual evaluation, Performance comparison, Crosstalk (<20.25%)
[325]	COMSOL	Power consumption (visually, generated)	Performance comparison, Sensitivity (15.6 V/MPa), Sensing range (0–1.1 MPa), Stability (1200 s), Frequency response, Response time (40 ms)
[267]	-	-	Sensitivity (elastic = 23.52 Ohm/Mpa, force = 19.27 Ohm/N, visually for both for the array and one single sensor), Resolution (4.25 kPa, 5.19 mN), Performance comparison, Repeatability (std of measured impedance shift = 2.5%), CD (0.9999), Crosstalk (“low crosstalk noise’’)
[99]	MatLab	Sampling frequency (10 kHz), Effects of ECs (“any change in mass resolution relative to the thermomechanical limit is <1%)	Sensing range (20–80 mm particle diameter), Noise (mass equivalent noise = 20–40 ag), Sensitivity (4.46–5.50 mHz/ag for type 0 sensors, and 15.2–18.6 for type 1 sensors), LOD (47 ag), BW (150 Hz), Frequency response, Response time (65.5 ms), CV (variation in diameter of the device < 8%), Performance comparison, Crosstalk (avoided by physical design), Repeatability (std sensitivity = 16% across 100 devices)
[153]	Python	ADC bits, Sampling frequency	MAE (20.86–27.60 ms), Sensitivity (100%), Specificity (100%), Repeatability (std of errors = 0.02–0.03 ms)
[250]	-	Power consumption (visually, generated)	Sensitivity (0.28 V/kPa for individual sensors, 0.15 V/kPa for the whole array), Sensing range (1–30 kPa), Stability (2000 cycles), Performance comparison, Flexibility, Frequency response
[53]	Weka, Processing, Arduino	ADC bits (12), Power consumption (“low’’), Sampling frequency (1 kHz)	Accuracy (96%), Repeatability (2.93–13.25%), Flexibility
[66]	-	Cost (“low-cost fabrication technique’’)	Sensitivity (gauge factor = 7.2%, visually for each individual sensor), Performance comparison, ARE (0.5%), Repeatability (std gauge factor = 2%, response variability between individual sensors = 0.006–13%), LOD (0.5% nominal strain), Sensing range (up to 2.5% strain). Flexibility, Creep (avoided by threshold)
[302]	-	-	MAE (position = 0.1–1.0 cm, orientation = 3–11°), MARE (6.3%), Frequency response, Crosstalk (“most crosstalk reflections were supressed’’)
[295]	-	-	-
[121]	SPICE	Sampling frequency (1 MHz)	Relative error (visually), Sensing range (100–1M Ω), Crosstalk (compensated)
[211]	-	-	Sensitivity ([x, y] = [2.9, 15.3] nH/N, Drift (due to mechanical imperfections), Visual evaluation, Frequency response, Repeatability (visually)
[148]	MatLab	Cost (“low-cost”), Sampling frequency (50 Hz)	Accuracy (100%)
[142]	-	-	Sensitivity (0.42 ${\mathrm{kPa}}^{-1}$), LOD (1 Pa), Repeatability (std for 4 sensors = 8 $\times \;10^{-4}$–4.9 $\times \;10^{-3}$ kPa, relative std for 4 sensors = 0.84–2.6%, relative std of individual sensors in range 0.8–1.57%), Response time (visually), Hysteresis (“negligible”), Flexibility, Stability (1000 cycles)
[367]	-	-	Flexibility, Frequency response
[208]	MatLab	Effects of ECs (−3.14% output at 100 °C)	Sensitivity (1.60 ${\mathrm{kPa}}^{-1}$), Performance comparison, Response time (111/215 ms), Repeatability (3 cycles at 38.45, 107, and 177.82 Pa), LOD, Resolution (13.61 Pa), Stability (0.3% in 32 h), Sensing range (0–0.18 kPa), Noise (0.08% alteration), Crosstalk (“mechanical interferences’’)
[260]	Solidworks	Sampling frequency (166 Hz)	Flexibility, Visual evaluation
[313]	Arduino	Power consumption (“helps to save energy’’)	-
[210]	-	-	Performance comparison, SNR (visually), BW (10 Hz), Linearity error (<3%), Frequency response, Sensitivity (visually for each individual sensor), Sensing range (20.1 cm), Stability (“good stability’’), Noise (floor = 0.235 nT/${\mathrm{Hz}}^{1/2}$)
[170]	LabView	Cost (“cost-effective”), Sampling frequency (24 Hz)	Resolution (9.6 μϵ, Sensing range (0–0.7% strain), Flexibility, Frequency response, Linearity error (7.5 $\times \;10^{-4}$%)
[179]	-	Cost (“low-cost”)	Error (visually), Absolute error (visually), Sensing range (360°), Performance comparison, Accuracy (±2”), Crosstalk (eliminated by structure)
[96]	-	Power consumption (visually)	ARE (<0.6% for R, <1.2% for C), Sensitivity (commercial sensors), Resolution (relative resolution in range 0.08–0.13% for R, and 0.12–1.8% for C), Repeatability (std = 0.5–1 kΩ)
[207]	MatLab	Sampling frequency (2 samples per second)	Visual evaluation, Performance comparison, Noise
[159]	COMSOL	Sampling frequency (10 kHz), Cost (“cost-effective fabrication process”)	Flexibility, Sensing range (0–200 kPa), LOD (2 Pa), CD (0.981), Repeatability, Response time (100 ms), Stability (10,000 s), Frequency response, Sensitivity (0.02–0.25 ${\mathrm{kPa}}^{-1}$)
[63]	-	Power consumption (generated power density = 422 mW/$m^{2}$)	Sensitivity (average = 0.627 pF/N, visually for some individual sensors, max = 0.19 pF per 0.8 N), Hysteresis (5.12%), Repeatability (std of sensitivity = 3.4%, std of initial capacitances = 29.5 fF), Performance comparison, Flexibility, Sensing range (up to 200 kPa)
[195]	COMSOL	-	Visual evaluation, Flexibility, Error (visually)
[132]	MatLab, Simulink	ADC bits (8–16)	RMSE (<0.06%), Crosstalk (compensated), Sensing range (10 kΩ ± 10 kΩ)
[74]	-	-	ARE (0.19–3%), RMSE (8.337), CD (0.9976), Sensing range (50–100 lbs)
[203]	SPICE, Smartphone app	Effects of ECs	Selectivity, Response time (visually), Accuracy (visually for each individual sensor), Sensitivity (visually for each individual sensor), Stability (Allan deviation = 2.2 Hz), Visual evaluation (all metrics are shown in Figures), Drift (“no significant drift was observed”)
[110]	Python	Sampling frequency (10 Hz)	ARE (3.5–10.6%), MARE (1.2 $\times \;10^{-10}$–24.3%), Repeatability (std MARE = 0.47 $\times \;10^{-10}$–2.8%), Performance comparison
[72]	-	-	Sensitivity (0.31 ${\mathrm{kPa}}^{-1}$), Hysteresis (“hysteresis-less’), Sensing range (up to 22.1 kPa), Stability (“repeated tests at 30 kPa’’), Resolution (0.03 g)
[197]	LabView	Power consumption (<700 µW)	Performance comparison, Sensitivity (350 mV/fF), Response time (visually)
[272]	-	-	ARE (<15%), Performance comparison, Stability (10 groups of tests), Frequency response
[252]	-	ADC bits (16)	Sensitivity (individual sensors in range 200–700 pC/N, average = 25.5 pC/N), RMSE (individual sensors in range 2.2–62 pC), Sensing range (0.03–0.3%), Repeatability (std of sensitivity of individual sensors in range 1.1–2.2 pC/N, average = 1.5 pC/N), SNR, Flexibility, Frequency response.
[266]	COMSOL, LabView	Sampling frequency (200 Hz)	Sensitivity ([x, y, z] = [0.37, 0.41, 0.34] V/N, 0.0641 V ${\mathrm{mm}}^{-1}$, visually for each individual sensor)
[277]	-	Sampling frequency (4.9 kHz), ADC bits (10)	Accuracy (97%), BW (1–150 Hz), Frequency response
[276]	-	Sampling frequency (4.9 kHz), ADC bits (10)	Accuracy (97%), BW (1–150 Hz), Frequency response
[175]	-	-	Sensitivity (2.04 ${\mathrm{kPa}}^{-1}$), Flexibility, Sensing range, LOD (<7 Pa), Response time (<100 ms), Stability (1000 cycles), Hysteresis (visually), Performance comparison, Repeatability (std of sensitivity = 0.08–0.16 ${\mathrm{kPa}}^{-1}$)
[172]	SolidWorks, ANSYS	Effects of ECs	Sensitivity (6.55% ${\mathrm{kPa}}^{-1}$), Performance comparison, Response time (70 ms), Flexibility (500 cycles at 8 cm bending radius), Repeatability (2500 cycles), Sensing range (0–30 kPa), Hysteresis (“without hysteresis”), Crosstalk (“crosstalk-free”), Stability (2500 cycles), Drift (“without significant drift”)
[261]	-	Power consumption (visually, generated)	Sensitivity (4.07–4.91 mV/μϵ), Repeatability (variation in sensitivity = 1 mV/μϵ), Flexibility, Frequency response
[206]	Custom Software, C	ADC bits (16)	Visual evaluation, Sensitivity (visually for each individual sensor)
[181]	COMSOL	Cost (“low cost”), Sampling frequency (2 kHz per channel), ADC bits (12)	Sensitivity (3.53 V/pF), SNR (26.6–57 dB), Response time (4 µs), Resolution (3.1 fF), Sensing range (0–45 cm), Performance comparison, Linearity error (6.31%), Crosstalk (0.5 V)
[171]	-	Cost (“cost-effective’’)	Performance comparison, Flexibility, Frequency response, Repeatability (150,000 cycles), Drift (due to ECs)
[50]	C	-	Absolute Error (<30 kPa), Sensitivity (2.6125 ${\mathrm{Mpa}}^{-1}$), Repeatability (7.5%), Sensing range (0–0.4 Mpa), Linearity error (<0.15%)
[310]	COMSOL	TCF (−36.3 ppm/°C, −77.9 ppm/°C)	Sensitivity (719 Hz/% RH), Sensing range (10–90% RH), Resolution (0.4% RH), LOD, Repeatability (variation < 3.2%), Hysteresis (<4%), Response time (78/54 s), Performance comparison, CD (0.999), Frequency response, Stability (4 days for 1 month)
[262]	COMSOL	-	Visual evaluation, Repeatability (visually)
[118]	SPICE, MatLab	Effects of ECs (“requires calibration’’)	MARE (<1%), Sensitivity (visually), Sensing range (100–10M Ω), Crosstalk (compensated), Repeatability (10 repeats)
[59]	-	Cost (“low-cost’’), Effects of ECs (<10% change in [−20, 50] °C range)	Sensitivity (5.41–16.9 ${\mathrm{kPa}}^{-1}$ the whole array, visually for some individual sensors), Sensing range (0.1–1.3 kPa), Response time (29.7/47 ms), Flexibility, Hysteresis (“moderate hysteresis’’), Stability (1000 cycles), Repeatability (visually), Crosstalk (compensated)
[60]	COMSOL	TCR (=sensitivity)	Sensitivity (−0.105 MΩ/°C), CD (0.964), Repeatability (3 experiments)
[119]	SPICE	-	ARE (0.00–2.12%), Performance comparison, Sensing range (100–10k Ω), Drift (near-zero OA temperature drift), Crosstalk (compensated)
[73]	ANSYS	Spatial resolution (2 mm), Power consumption (30 mW), Sampling frequency (1 kHz)	Sensitivity (four sensors are in range 0.11–0.31 mV/mN·V), Performance comparison, SNR (39.2), Sensing range (0.01–0.25 N)
[341]	-	Effects of ECs, Power consumption, Sampling frequency	Noise, BW, Frequency response, Sensitivity, Resolution, Accuracy
[177]	FEA	Effects of ECs (“eliminated by the differential approach”)	Sensing range (10–100 kPa), Frequency response (visually)
[219]	-	Spatial resolution (<1 µm)	Visual evaluation
[155]	ADINA	Cost, Sampling frequency	Sensitivity (strain gauge factor = 0.1 (real), 0.56 (simulation)), Performance comparison, Response time (1.3/0.5 s), Sensing range
[187]	Custom software	-	Noise (<1%), Sensitivity (visually), Sensing range (30–90 mm)
[357]	Arduino	Cost (“low cost’’)	Performance comparison, Accuracy (86–96%)
[348]	Quartus	Cost (“hardware cost is extremely low”), Sampling frequency (200 MHz)	Sensitivity (940 rad/g), CD (0.997), Visual evaluation, Sensing range (0.002–0.02 g)
[349]	-	Sampling frequency (visually)	Frequency response, Noise (visually), Resolution (4 $\times \;10^{-4}$ rad/$\sqrt{\mathrm{Hz}}$), Crosstalk (<−60 dB), Drift (due by ECs)
[339]	LabView	Cost, Sampling frequency (50 kHz)	Error (visually), Sensitivity (depends on the frequency), Noise, BW/Sensing range (200–2000 Hz), Frequency response
[166]	Arduino	Effects of ECs (“is not affected by temperature’’)	Sensing range (0.211–0.306 kg/${\mathrm{cm}}^{2}$), Resolution (individual sensors in range 0.211–0.306 kg/${\mathrm{cm}}^{2}$ per 3 kg/${\mathrm{cm}}^{2}$), Sensitivity (circuit = 5 fF, individual sensors in range 51–71 fF per 3 kg/${\mathrm{cm}}^{2}$), ARE (individual sensors in range 2.0–11.8%), Drift (“low drift”), Crosstalk (compensated)
[136]	SPICE, MatLab	Cost (“integration of more devices leads to high cost’’)	Relative error (−0.0047% to 0.354%), ARE (20.56%), Sensing range (200–100k Ω), Crosstalk (compensated)
[278]	MatLab, ANSYS	-	Performance comparison, Frequency response, Visual evaluation
[61]	-	Power consumption (201 µW generated)	Sensitivity (0.24–4.72%/N, visually for individual threads), Hysteresis (4.6%), Flexibility, Stability (8,000 cycles), Frequency response, Sensing range (0–6 N), Repeatability (std = 30–40 fF)
[289]	LabView	Sampling frequency (200 kHz)	Performance comparison, Frequency response, Visual evaluation
[284]	SAS, MatLab	Cost (“low-cost solution’’)	Correlation coefficient (0.92), MAE (0.24 m/s), CV (8.26%), Repeatability (2×std of MAE = 1.91 m/s), Performance comparison (Bland-Altmann)
[105]	MatLab, Proteus	ADC bits (22), Sampling frequency (60 SPS)	MARE (average < 5%, measured individual values in range [5.2 $\times \;10^{-4}$, 17.8]%), Resolution (5 decimal point)
[106]	MatLab, Proteus	ADC bits (16), Sampling frequency (250 kSPS)	MARE (average < 5%, measured individual values in range 0.06–199.6%), Performance comparison
[127]	NI Multisim, MatLab	ADC bits (16), Power consumption (“high’’)	Relative error (<3.9%), Absolute error (<7%), Sensing range (1–100kΩ), BW (5.5 MHz), Crosstalk (compensated)
[54]	MatLab	-	Repeatability (“good’’), Hysteresis (“small hysteresis’’), Response time (0.25–0.35 ms), Resolution (0.01 N), Flexibility (strechtability = 15%)
[321]	LabView	Sampling frequency, Power consumption (4.3–6.6 µA generated)	Frequency response, Visual evaluation
[326]	COMSOL, Arduino	Power consumption (0.3 µW generated)	Sensitivity (1.073–1.52 mV/Pa), CD (0.94–0.993), Response time (50/50 ms), Stability (3000), Flexibility (stretch nearly 150%), Selectivity (0.9–0.977), Frequency response, LOD (250 Pa)
[58]	-	Power consumption (low-power CMOS), Cost (cost/area = Low, cost/transistor = High), Spatial resolution (1.03 sensors/${\mathrm{cm}}^{2}$)	Performance comparison, Flexibility, Visual evaluation
[286]	-	Spatial resolution (0.2–0.3 mm), Sampling frequency (1 kHz)	Performance comparison, Sensing range (13.5 mm), Repeatability (in various subjects), Noise (shielding), Flexibility
[222]	LabView	Spatial resolution (1 mm)	Sensitivity (9.43 $\times \;10^{-6}$ to 2.05 $\times \;10^{-4}$ ${\mathrm{kPa}}^{-1}$), Hysteresis (“negligible’’), Repeatability (2500 FETs), Response time (31/49 ms), SNR (1068), Sensing range (250 Pa–3 Mpa), Performance comparison, Crosstalk (eliminated by structure)
[217]	-	Effects of ECs (2 temperature monitors are used)	Performance comparison
[231]	Sentaurus, SPICE	Cost (“low-cost production’’)	Sensitivity (visually), Resolution (visually)
[226]	-	Effects of ECs, Cost (“low-cost’’)	Visual evaluation, Sensitivity (visually)
[230]	SPICE, Verilog, Spectre, NIST ASTAR	-	Performance comparison, Sensitivity (visually)
[6]	-	-	Sensitivity (visually for each individual sensor), Response time (visually for each individual sensor), Repeatability (“high’’), Performance comparison
[115]	NI Multisim	-	Relative error (−12.39% to 5.01%), Performance comparison, Sensing range (0.1–30 Ω), BW (5.5 MHz), Crosstalk (compensated)
[67]	-	-	ARE (<0.27%), Frequency response, Sensing range (10.8–11.8V)
[5]	-	Effects of ECs, TCF	Sensitivity (−28 Hz/µT), Performance comparison, Frequency response, Sensing range (0–90 Oe)
[259]	-	Power consumption (1–7 V, 50–150 nA generated)	Stability (3 days at 4 Hz), Flexibility, Frequency response, Visual evaluation
[264]	-	Power consumption (10 nW generated)	Visual evaluation, Sensitivity (visually), Crosstalk (“limits multi-touch applications’’), Stability (visually)
[275]	LabView	-	Accuracy (99.93%), Repeatability (std = 1.31%), CD (0.996), Frequency response
[135]	SPICE	Cost (balance cost-reading speed)	ARE (visually), Performance comparison, BW (4 MHz), Sensing range (1–100 kΩ), Crosstalk (compensated)
[298]	-	Sampling frequency (1 MHz)	ARE (0.20%), Absolute error (0.0787), Frequency response
[223]	ANSYS	-	Sensitivity (array = 0.107 mA/nN, single sensor = 18.18 µA/µm), LOD (340 nN), Performance comparison, Crosstalk (“low cross-axis sensitivity’’), Linearity error (0.25% FS), ARE (<2%)
[69]	ANSYS, MatLab, C	TCS (individual sensors in range [−4.1 $\times \;10^{-6}$, −1.41 $\times \;10^{-6}$] $K^{-1}$), Power consumption (max = 5 $\times \;10^{-3}$ mW/µ$m^{2}$)	Relative error (overall error in range = ±0.252%), Sensitivity (individual sensors in range 0.020–0.052 mV/V/kPa), SNR (visually), Repeatability (error = 0.152%), Hysteresis (error = 0.183%), Sensing range (0–100 kPa), Linearity error (0.8129–0.833 mV/V/kPa), Creep (to do with hysteresis and repeatability), Drift (temperature drift)
[98]	MatLab, Arduino	Cost (“low-cost’’)	RMSE (individual sensors in range 3.544–300.4 ppm), CD (individual sensors in range 0.9907–0.9997), Correlation coefficient (0.56–0.91), Selectivity (in sensors datasheets), Noise (individual sensors in range 1.02–14.41%), Repeatability (3 days), Performance comparison
[196]	LabView	Cost, Sampling frequency (450 kHz), Power consumption	Sensitivity (350 mV/fF), Performance comparison, Sensing range (10–10k beads/ml), Resolution (5 af), Frequency response
[131]	-	Power consumption (“reduced power consumption’’), ADC bits (14)	ARE (0.0018% to 1.39%), Absolute error (50–300 Ω), Repeatability (10,000 tests), Performance comparison, Crosstalk (compensated)
[9]	Ansoft	-	ARE (<3%), Performance comparison, Frequency response, BW (0.64–120 Hz), Sensing range (0.64 Hz–76 MHz)
[3]	LabView, MatLab	Cost (<10$ per sensor)	Absolute error (10 Pa), RMSE (32.80–37.54 Pa), NRMSE (8.89–10.17%), Flexibility, Sensitivity (5 Pa, visually for each individual sensor), BW (0.2–3 Hz), Sensing range (50–500 Pa), Frequency response, Repeatability (several plungings), CD (0.8752–0.8881), Drift (“low-frequency DC drift’’)
[124]	NI Multisim	ADC bits (12)	Relative error (−0.162% to 0.077%), BW (0.6 MHz), Sensing range (0.1–100 Ω)
[165]	-	-	Visual evaluation, Performance comparison
[95]	LabView	TCR (0.044)	Sensitivity (0.044 $K^{-1}$), Performance comparison, Resolution (0.4 °C), Hysteresis (“very little hysteresis’’), Flexibility, Sensing range (20–100 °C), Repeatability (256 devices analyzed), Stability/Flexibility (3.2 mm bending radius)
[157]	LabView	-	Visual evaluation, Sensitivity (visually), Flexibility
[180]	COMSOL	-	Sensing range (visually), BW (0.25–0.85 GHz), Frequency response, Resolution (<1 mm), Performance comparison, Sensitivity (0.5 dB/mm)
[123]	NI Multisim	-	Relative error (−9% to 9%), Performance comparison, Sensing range, BW (0.6 MHz), Crosstalk (compensated)
[64]	LabView	ADC bits (10), Effects of ECs (175 mV/°C, 7.88 mV/% RH)	Sensitivity (visually), Frequency response, Resolution (0.15 psi), Repeatability (std = 12.4 mV), Sensing range (0–10 psi), Flexibility, SNR (individual sensors in range 13.07–20.6), Crosstalk (4.2–6.76%), Drift (temperature drift)
[239]	CasaXPS	Cost (“cost-effective strategy’’)	Performance comparison, Sensitivity (visually), LOD (1 nM), Repeatability (24 devices), Specificity
[90]	COMSOL	-	Sensitivity (visually), Performance comparison
[125]	NI Multisim	Power consumption (visually)	Relative error (−0.06% to 2.10% simulation, −40% to 4% prototype), BW (5.5 MHz), Sensing range (500–90k Ω), Crosstalk (compensated)
[234]	-	Cost (“cost-effective platform’’)	Sensing range (linear in 7–9 pH), Sensitivity (15%/pH unit for one sensor, visually for the array), Selectivity, LOD (50 µM), Repeatability (visually)
[202]	Python, Arduino	Power consumption (10 µW), Sampling frequency (1 s)	Response time, Visual evaluation
[359]	LabView	Sampling frequency (200–1k Hz)	Sensitivity (9 mV/G), MAE (2.1 mm in position, 6.7 in rotation), Performance comparison, Noise (15 mV), Repeatability (std or MAEs = 0.8 mm in position, 4.3° in rotation), Sensing range (5 cm)
[265]	LabView	-	MARE (average = 10.68%, each individual sensor in range [2.27, 20]%), Sensitivity ([x, y, z] = [14.93, 14.92, 6.62] pC/N), Sensing range (0–1.5 N), Noise (visually), Frequency response, Repeatability (std MARE = 6.84%), BW (5–400 Hz), Crosstalk (“relatively small’’), Linearity error ([x, y, z] = [2.45, 2.37, 1.74]% FS)
[296]	ANSYS, MatLab	-	Visual evaluation, Frequency response
[335]	MatLab, ANSYS	Sampling frequency (>1000 Hz)	Visual evaluation, Frequency response
[309]	-	-	Resolution (0.58–0.75 nT), Noise (floor = 0.13–0.17 nT/${\mathrm{Hz}}^{1/2}$), Sensitivity (nT), BW (184.8–191.5 Hz), Frequency response, Performance comparison
[345]	-	-	Performance comparison
[306]	The Unscrambler	Sampling frequency (1 Hz)	Frequency response, Sensitivity (for each individual sorbent and depending on the test substance, in range [0.16, 300]), Accuracy (visually)
[303]	-	-	Frequency response, Visual evaluation
[294]	-	-	RMSE/Linearity error (0.1001), MARE (9.2%), Frequency response, Repeatability (visually)
[333]	-	Sampling frequency (200 Hz), Effects of ECs (sensitivity = 6.67 $\times \;10^{-6}$ °$C^{-1}$)	Sensitivity (0.78 $\times \;10^{-6}$ μϵ), Resolution (1 μϵ), Performance comparison
[274]	-	-	Visual evaluation, Sensitivity (0.18 mV/μϵ)
[332]	-	TCS (compensated), Cost (“low-cost multiplexing”), Sampling frequency (100 samples/s)	ARE (<0.001%), Sensitivity, Resolution (<0.4 nϵ), Noise (0.4 nϵ/$\sqrt{\mathrm{Hz}}$, BW (0.01–50 Hz), Frequency response, Sensing range (up to 24.9 μϵ), Drift (compensated), Repeatability (uncertainty level = 0.04%)
[308]	MatLab	TCF (−23.09 ppm/K, −23.15 ppm/K)	Sensitivity (2.3–2.6 kHz/nH), Frequency response
[291]	-	Sampling frequency (0.14 μs), Cost (“low-cost PZTs’’)	SNR (41.12–47.97 dB), Performance comparison, Frequency response, BW (5–250 kHz)
[122]	MatLab, NI Multisim	-	Relative error (visually), Sensing range (studied in different ranges), BW (OA gain-bandwidth = 0.6 MHz)
[293]	ANSYS	Cost (“serial production can reduce the costs’’), Sampling frequency (sampling period = 4.883 µs)	Visual evaluation, Performance comparison
[270]	-	-	Sensitivity (0.16 mV/μϵ), Visual evaluation, Performance comparison
[128]	SPICE	Power consumption (“severely increased’’), Cost (“severely increased’’)	ARE (0.1%), Response time (13 µs), Sensing range (100–1M Ω), Crosstalk (compensated)
[323]	COMSOL	Power consumption (2.45 µW generated)	Repeatability (visually), Stability (5000 cycles), Performance comparison, Frequency response
[130]	-	ENOB (10.14), ADC bits (14)	ARE (<0.066%), Absolute error (<20 Ω), Resolution (1.70–6.3 Ω), Repeatability (8,000 measurements), Crosstalk (compensated), Sensing range (200–7350 Ω)
[126]	NI Multisim	ADC bits (12)	Relative error (<12%), Performance comparison, BW (18 MHz), Sensing range (1–128 Ω), Crosstalk (compensated)
[129]	SPICE	Sampling frequency (200 frame/s)	ARE (0.089–0.69%), BW (125 MHz), Sensing range (100k–1000M Ω), Performance comparison, Crosstalk (compensated)
